# A prospective evaluation of AI-augmented epidemiology to forecast COVID-19 in the USA and Japan

**DOI:** 10.1038/s41746-021-00511-7

**Published:** 2021-10-08

**Authors:** Sercan Ö. Arık, Joel Shor, Rajarishi Sinha, Jinsung Yoon, Joseph R. Ledsam, Long T. Le, Michael W. Dusenberry, Nathanael C. Yoder, Kris Popendorf, Arkady Epshteyn, Johan Euphrosine, Elli Kanal, Isaac Jones, Chun-Liang Li, Beth Luan, Joe Mckenna, Vikas Menon, Shashank Singh, Mimi Sun, Ashwin Sura Ravi, Leyou Zhang, Dario Sava, Kane Cunningham, Hiroki Kayama, Thomas Tsai, Daisuke Yoneoka, Shuhei Nomura, Hiroaki Miyata, Tomas Pfister

**Affiliations:** 1 Google Cloud AI, 1170 Bordeaux Dr, Sunnyvale, CA USA; 2 Google, Japan, Shibuya, 3-Chrome-21-3, Tokyo, Japan; 3 Google Health, 1600 Amphitheatre Parkway, Mountain View, CA USA; 4 Harvard School of Public Health, 677 Huntington Ave, Boston, MA USA; 5 Department of Health Policy and Management, School of Medicine, Keio University, 35 Shinanomachi, Shinjuku-ku, Tokyo Japan; 6 Division of Biostatistics and Bioinformatics, Graduate School of Public Health, St Luke’s International University, 3-6-2 Tsukiji, Chuo-ku, Tokyo, Japan; 7 Department of Global Health Policy, Graduate School of Medicine, The University of Tokyo, 7-3-1, Hongo, Bunkyo-ku, Tokyo, Japan; 8 Department of Healthcare Quality Assessment, Graduate School of Medicine, The University of Tokyo, 7-3-1, Hongo, Bunkyo-ku, Tokyo, Japan

**Keywords:** Epidemiology, Risk factors

## Abstract

The COVID-19 pandemic has highlighted the global need for reliable models of disease spread. We propose an AI-augmented forecast modeling framework that provides daily predictions of the expected number of confirmed COVID-19 deaths, cases, and hospitalizations during the following 4 weeks. We present an international, prospective evaluation of our models’ performance across all states and counties in the USA and prefectures in Japan. Nationally, incident mean absolute percentage error (MAPE) for predicting COVID-19 associated deaths during prospective deployment remained consistently <8% (US) and <29% (Japan), while cumulative MAPE remained <2% (US) and <10% (Japan). We show that our models perform well even during periods of considerable change in population behavior, and are robust to demographic differences across different geographic locations. We further demonstrate that our framework provides meaningful explanatory insights with the models accurately adapting to local and national policy interventions. Our framework enables counterfactual simulations, which indicate continuing Non-Pharmaceutical Interventions alongside vaccinations is essential for faster recovery from the pandemic, delaying the application of interventions has a detrimental effect, and allow exploration of the consequences of different vaccination strategies. The COVID-19 pandemic remains a global emergency. In the face of substantial challenges ahead, the approach presented here has the potential to inform critical decisions.

## Introduction

Predicting the spread of infectious diseases is an essential component of public health management. Forecasts have contributed to resource allocation and control measures in past epi- and pandemics such as influenza^1^ and Ebola^2^. Most recently, such models have shown promise during the COVID-19 pandemic^3,4^ by helping ease the devastating public health and economic crisis^5–9^. However, forecasting models must overcome multiple challenges. Existing datasets contain substantial noise due to inconsistencies in reporting and the fact that many cases are asymptomatic or undocumented^10,11^, and the causal impact of features within the available data is unknown. The nature of the data and the fundamental dynamics changes over time as the progression of the disease influences public policy^12^ and individuals’ behaviors^13^ and vice versa. Beyond overcoming these, forecasting models must be explainable for decision-makers to be able to interpret the results in a meaningful way^14^.

Recent work has demonstrated promising results with retrospective evaluations^3,4,15–19^. On the other hand, to understand the value of such models and their potential utility to policy decisions, a prospective evaluation is essential. Further, the utility of the forecasts needs to be rigorously validated, which is of crucial importance if such forecasts are to play a role in vaccination strategies given the wide variation in vaccine distribution, effectiveness, and uptake^20,21^.

To address these challenges, we introduced an AI-augmented epidemiology framework to forecast the expected burden of COVID-19 4 weeks into the future, along with a rigorous framework for training and validation^22^, and made the forecasts publicly available.

We run a prospective observational cohort study to validate the framework in the United States of America (USA) and Japan, two countries with substantial differences in healthcare systems, demographics, and the policy response to COVID-19. We demonstrate the efficacy of the framework by deriving new epidemiological findings, evaluating the predicted effect of changes in policy and behavior, and exploring settings in which the framework is being used such as hospital resource allocation and guiding state-wide social distancing policies.

## Results

### An AI-augmented approach to epidemiology

**Fig. 1:**
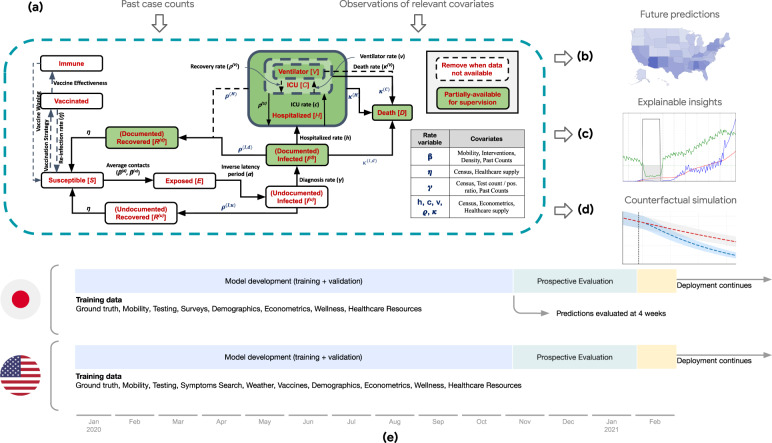
Proposed framework and timeline for model development and prospective evaluation. **a** Our proposed AI-augmented epidemiology framework for COVID-19 forecasting is an extension to the standard Susceptible-Exposed-Infectious-Removed (SEIR) model^23,48^. We model compartments for undocumented cases explicitly as they can dominate COVID-19 spread, and introduce compartments for hospital resource usage as they are crucial to forecasts for COVID-19 healthcare planning. Learnable encoders infer the rates at which individuals move through different compartments, trained on static and time-varying public data, to model the changing disease dynamics over time and extract the predictive signals from relevant data. The models are trained daily on all available data up to the day each prediction is made (see “Methods”). **b** Public dashboard that shows generated 28-day forecasts at county- and state level for the USA. A dashboard was similarly created in Japan at the prefecture level. **c** Predictions for the effective R number and force of infection that come from the compartmental nature of the model, as well as feature importances for the rates from the variable encoder architectures. **d** Simulations of counterfactual scenarios can be used to estimate the potential impact of vaccines or policy measures. **e** Prospective evaluation of the forecasts— on each prediction date, 28-day forecasts are released publicly, and the evaluation of the accuracy is performed at the end of the 28-day horizon.

Our framework is an extension to the Susceptible-Exposed-Infectious-Removed (SEIR) model, where a population is assigned to and may flow between compartments representing disease states^23^ (Fig. 1a). Our models are optimized for the prediction of COVID-19-associated deaths and are trained on static and time-series data.

Our framework makes use of a number of key technical approaches. We use a larger number of static and time-varying features compared to prior work, powered by a systematic end-to-end learning approach with robust training mechanisms. We use a novel sequence-to-sequence modeling approach to minimize amplified growth of errors into the future due to robustness issues. Instead of depending on human-engineered functions or distribution priors to capture the impact of features, we use learnable time-varying functions to relate features to compartmental variables, which are trained in an end-to-end way. We include hospitalization compartments and demonstrate efficient training to forecast hospitalization compartments despite data sparsity. Our framework yields forecasts for a large number of locations. In our framework, a single model can provide forecasts for more than 3000 US counties with very distinct dynamics and characteristics; and indeed benefits from this transferring information across locations. Beyond point forecasts, our framework yields prediction intervals using quantile regression, and we show these are well-calibrated to capture uncertainty. We also report counterfactual simulations, and demonstrate that the counterfactual outcomes capture the relationship between the inputs and outcomes. Finally, the feature encoders in our framework can suggest the most important factors driving compartmental transitions. For additional technical details on the framework please see “Methods”.

### A prospective evaluation of forecasting accuracy

**Fig. 2:**
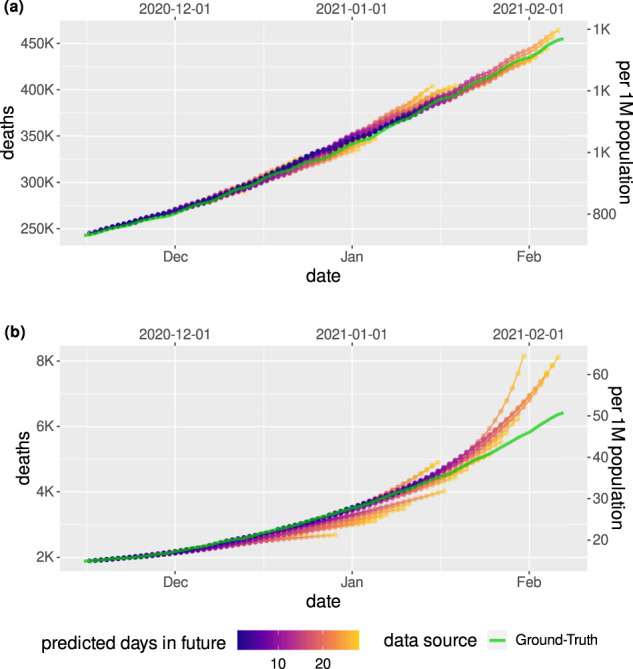
Prospective forecasts for the US and Japan models. Ground truth cumulative deaths counts (cyan lines) are shown alongside the forecasts for each day. Each daily forecast contains a predicted increase in cases for each day during the prediction window of 4 weeks (shown as colored dots, where shading shifting to yellow indicates days further from the date of prediction in the forecasting horizon, up to 4 weeks). Predictions of deaths are shown for (**a**) the USA, and (**b**) Japan.

To evaluate our framework, we conducted a prospective observational study over 8 weeks in the USA and Japan. Predictions were made daily, each looking 4 weeks into the future (Fig. 2). We predicted the number of COVID-19 associated deaths and confirmed cases. For the USA, the available data also allowed us to predict hospitalizations, intensive care (ICU) admissions, and admissions requiring mechanical ventilation. We report both incident and cumulative mean average percentage error (MAPE) averaged across locations, as well as aggregate average percentage error (AAPE) summed over locations, in the USA and Japan (for results on absolute error, see Supplementary Discussion). Incident metrics only consider the cases that occurred during the 28-day prediction window and are helpful in the context of resource allocation. Cumulative metrics additionally take into account the total existing number of cases and can provide a broader view in the context of the pandemic thus far.

During the prospective period, across the USA as a whole, the framework achieved an AAPE of 1.4% (95% CI [1.1%, 1.6%]) for cumulative deaths and 7.1% (95% CI [5.8%, 8.5%]) for incident deaths. The framework predicted cumulative confirmed cases, incident confirmed cases, hospitalizations, intensive care unit (ICU) admissions, and admissions requiring mechanical ventilation with AAPEs of 9.20% (95% CI [8.3%, 10.2%]), 18.6% (95% CI [14.6%, 22.6%]), 59.0% [41.3%, 76.7%], 66.1% [40.2%, 92.0%], and 51.7% ([37.0%, 66.5%]), respectively. For the USA, we also provide state- and county-level predictions. When evaluating at state level and averaging across all locations, the framework achieves MAPE for cumulative deaths and confirmed cases of 5.4% [5.1%, 5.6%] and 9.2% [8.2%, 10.1%], and incident deaths and confirmed cases of 24.2% [22.9%, 25.6%] and 37.8% [34.4%, 41.2%], respectively. At county level, MAPE for cumulative deaths and cumulative confirmed cases were 25.1% [23.1%, 27.0%] and 12.8% [11.5%, 14.1%], respectively. Predictions of cumulative deaths achieved an average percentage error <10% or average error <100 for 43/51 states and 2585/3006 counties, and for cumulative confirmed cases 34/51 states and 1647/3006 counties (Supplementary Tables 1 and 2).

We compare our framework with alternatives for the US (there are no public prefecture models in Japan to compare to). For statistical significance, we employ a two-sided Diebold–Mariano (DM) test (Supplementary Table 15). For cumulative deaths, the DM statistics on mean AE (MAE) are negative for all comparisons other than “Karlen-pypm”— our framework has a lower MAE in 32 out of the 33 models—and the difference with “Karlen-pypm” is statistically insignificant. Our model’s forecasts are statistically significantly different for 13/33 comparisons, and in all of these cases, our model’s MAE is lower. Using MAPE of cumulative deaths, “COVIDhub-ensemble”—a combined forecast that includes our framework’s predictions—has a negative DM statistic and a slightly lower MAPE, but this difference is not statistically significant (Supplementary Table 16). For the MAPE of incident deaths over the 4 weeks are compared, three comparisons have a negative DM statistic but none of those differences is statistically significant (Supplementary Table 17). Our model did, however, have significantly lower MAPE than five other models for incident deaths. For hospitalization predictions (Supplementary Tables 21 and 22), our model is the most accurate of the nine models, with a statistically significant difference for eight out of nine of the comparisons for MAE. For confirmed cases, the ranking of our model is lower and more variable. There are no statistically significant differences for MAE or MAPE for either cumulative or incident cases. We attribute this to the low data quality for confirmed cases, which has been widely reported^24–30^, where inconsistencies in testing and reporting limit the forecastability of cases compared to hospitalizations and deaths, which have more standardized reporting. Because of this lower-quality data, we also put a lower weight on errors in confirmed cases than deaths in our multitask learning objective (see “Methods”) compared to deaths. We note that our framework can be readjusted if the desired use case places more weight on confirmed cases prediction (see “Machine-learning methods”). In addition to lower-quality data, death and hospitalizations are lagging indicators, the prediction of which may benefit more from our proposed approach than confirmed cases do.

Beyond predicting point forecasts, we also compare the quality of prediction intervals using the weighted interval score (WIS)^31,32^, which is the discretized version of continuous ranking probability. Our models are in the top five and often the top model (Fig. 3, as well as Supplementary Figs. 3–11).

**Fig. 3:**
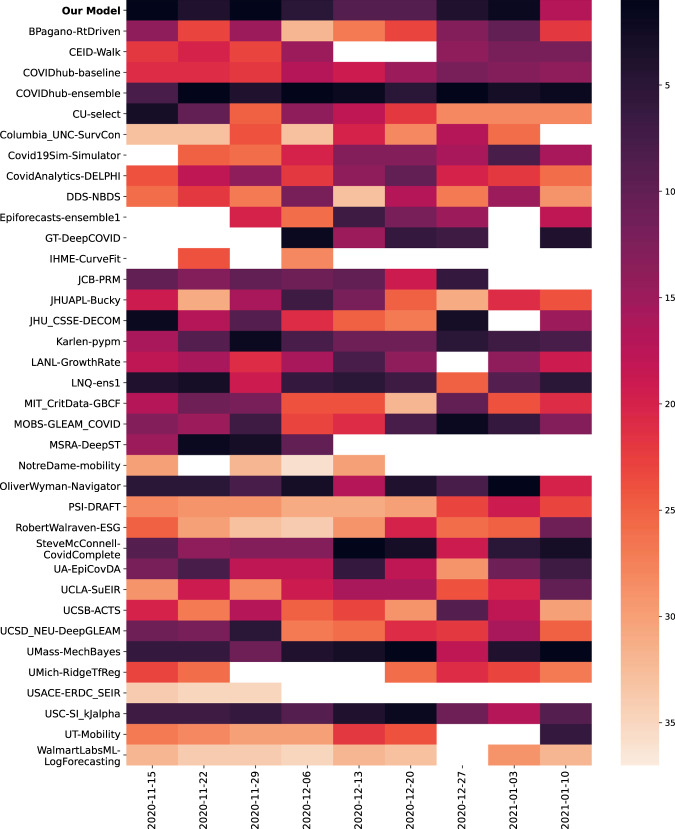
Model rankings for incident death MAPE. Model rankings for incident death MAPE in the prospective evaluation period. The darker the color, the higher the ranking of the model is for the corresponding prediction date.

We also validate our model performance in Japan, reporting AAPEs for cumulative deaths and confirmed cases over the next 4-week period, 9.8% (95% CI [7.4%, 12.2%]) and 9.1% (95% CI [5.7%, 12.5%], respectively. The corresponding incident deaths and confirmed cases were 28.8% (95% CI [22.3%, 35.3%]) and 26.7% (95% CI [16.0%, 37.4%]. Data were not available on hospitalizations, ICU admissions, and admissions requiring mechanical ventilation.

**Fig. 4:**
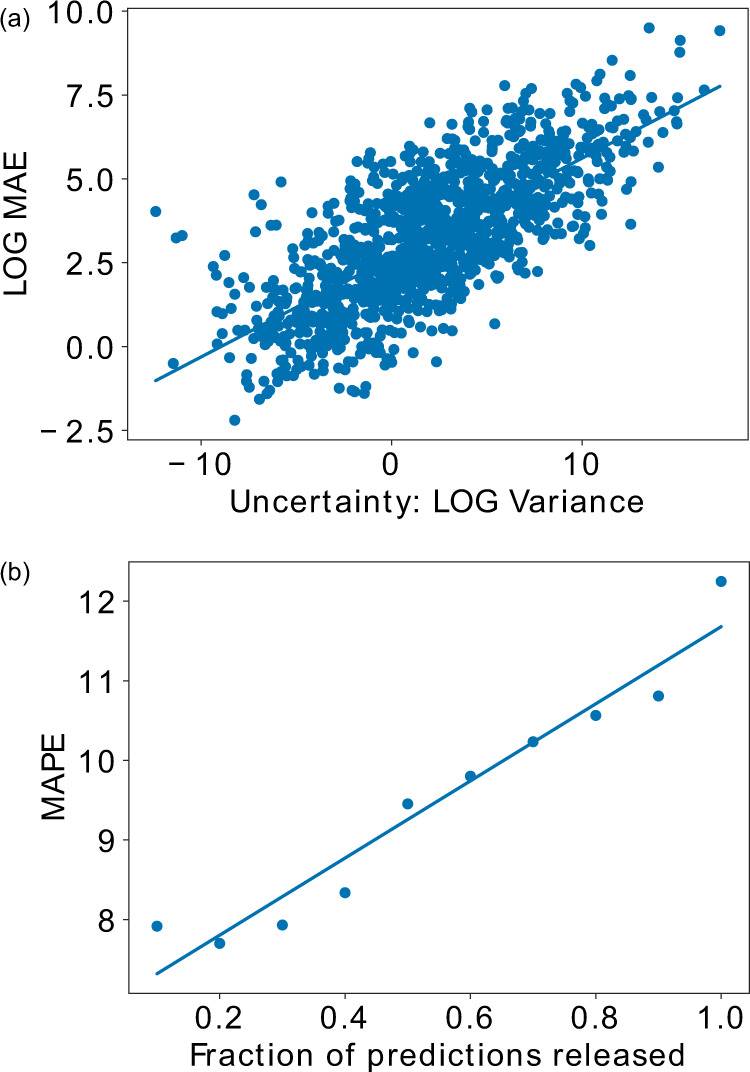
Model uncertainty. **a** Model disagreement due to model uncertainty, measured as average prediction variance across the top *k* = 5 models, versus the MAE performance, both plotted in log space. From this, we see that higher model disagreement correlates with worse metric performance. For the best fit line, *R*^2^ = 0.539, 4.39*x* + 3.37. **b** A rejection diagram showing the percentage of dates on which a prediction is made, after thresholding on model disagreement due to model uncertainty, versus the MAPE performance on those dates. From this, we can see that better average metric performance (on the days for which a forecast is released) can be achieved by withholding forecasts on days with higher model disagreement. Thus, we find the reliability of the forecasting system can be improved through model uncertainty thresholding. For the best fit line, *R*^2^ = 0.941, *f*(*x*) = 2.18*x* + 9.50.

The number of prefectures with an APE <10% or AE <10 for cumulative deaths and confirmed cases were 38/47 and 14/47, respectively (Supplementary Tables 3 and 4). As our models are well-calibrated, model uncertainty correlates with 28-day forecast accuracy (Fig. 4 and Supplementary Discussion). Calibrated uncertainty enables us to further improve the average prediction accuracy by flagging the uncertain predictions to be improved by human experts or other decision-making systems. For instance, we observe a 25% reduction in MAPE for cumulative deaths in Japan by only considering the most confident 50% of predictions (Fig. 4).

**Fig. 5:**
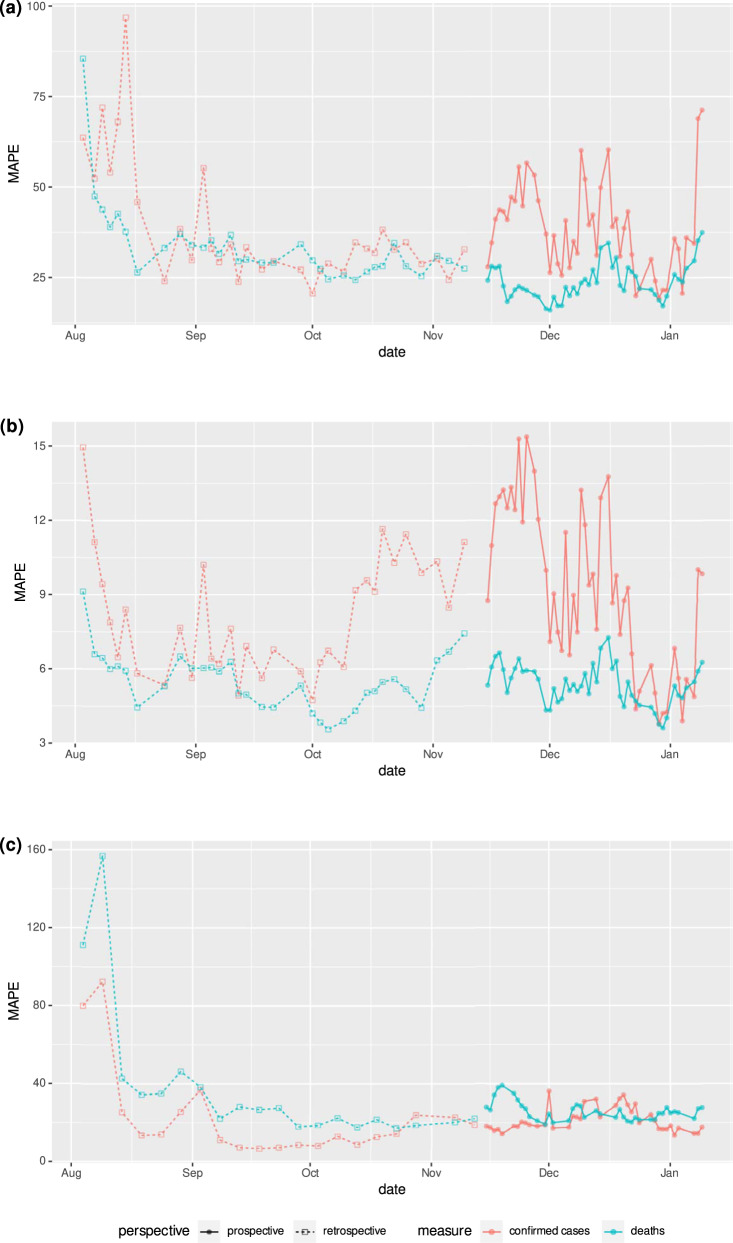
Retrospective and prospective 28-day MAPE over time. Performance over time is shown for the (**a**) state-level US models using 4-week incidental predictions (**b**) state-level US models using cumulative predictions (**c**) prefecture-level Japan model using cumulative predictions. Japan’s 4-week incidental deaths and cases were too low to meaningfully report. Metrics shown are the “mean absolute percentage error” for predicted deaths and predicted confirmed cases compared to ground truth. Retrospective performance during model development periods for confirmed cases (orange) and deaths (light blue) are shown alongside performance reported during the prospective study for cases (dark blue) and deaths (green).

In addition to evaluating our framework prospectively, we also show retrospective evaluations for dates before the prospective study began. Retrospective performance was calculated by training the model using data up to a particular prediction date, and evaluating 4 weeks after the prediction date. The framework uses the most recent version that includes any corrections made to previous data and there is no leakage of data from the future dates into model training. Our comparison shows that the MAPE during the prospective period was at most 1.3% above the MAPE for the retrospective period for both cumulative deaths and cumulative confirmed cases in both the USA and Japan (Fig. 5).

We chose a 28-day prediction window to balance the timescale useful for public health decisions to be made and the rapidly changing responses to the pandemic. It also allows us to make accurate comparisons with the models at COVID-19 Forecast Hub (see “Supplementary Discussion”). However, different settings may benefit from other prediction horizons (see Supplementary Fig. 2).

COVID-19 disproportionately affects certain demographic subpopulations^33–37^. We investigate the differences in performance across locations with greater proportions of key demographic groups. While statistically significant relationships between MAPE and several demographic variables were found, only small correlations remained for most subgroups after accounting for confounding variables, suggesting that the errors are not associated with the demographic variables of race, gender, population density, or income (see the section on fairness analysis in Supplementary Note 6).

### Ablation studies

Ablation studies demonstrate the most significant framework components that contribute to performance. Robust sequential learning with partial teacher forcing is critical for overall accuracy (yielding 34.9% ± 9.4% death and 9.6% ± 6.2% improvement in MAPE for cumulative confirmed cases). We also demonstrate the benefit of learning static and time-varying features, instead of using constant rates (yielding 1.6% ± 2.9% death and 2.2% ± 3.7% confirmed MAPE improvement). Information-sharing across locations can help generalize dynamics from other locations that have experienced similar pandemic phases in modeling confirmed cases (yielding 0.7% ± 0.8% MAPE improvement). Finally, we model hospitalization compartments, improving death forecasts (yielding 1.0% ± 1.1% MAPE improvement). See “Supplementary Discussion” for more detail.

### Using the framework to understand the COVID-19 pandemic

Compartment models allow predictions of how connected compartments change over time, potentially providing information on disease dynamics including estimates for the effective reproductive number (*R*_*eff*_), and the force of infection (*F*^(*u*)^, the rate at which susceptible individuals acquire the disease). Figure 6 demonstrates this for Texas, USA and Aichi, Japan, respectively (for all other locations, see Supplementary Figs. 36–46.

**Fig. 6:**
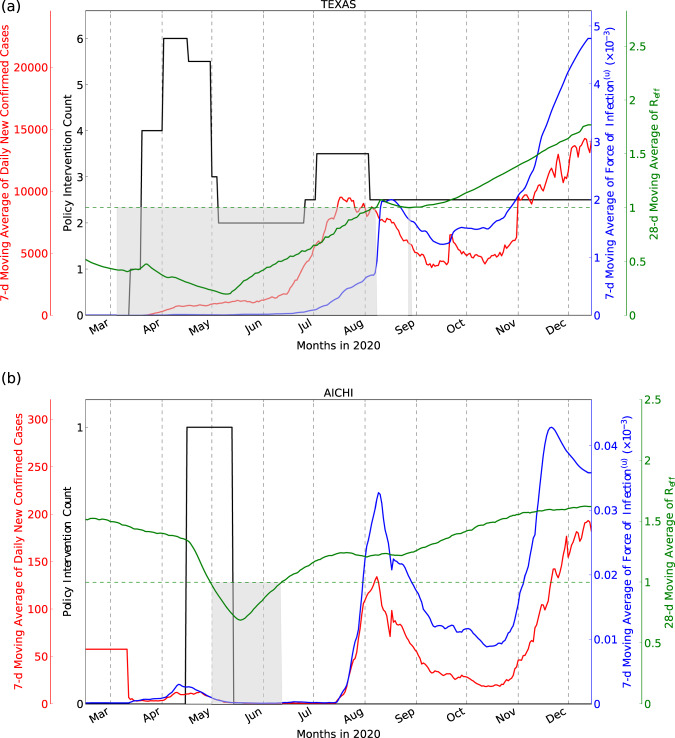
Model outputs. New daily confirmed cases, number of NPIs, *F*^(*u*)^ and *R*_*eff*_ for Texas, USA (**a**) and Aichi, Japan (**b**), chosen to represent a location with high and low COVID-19 associated deaths, respectively. Seven-day moving average of the daily confirmed case counts and number of Non-Pharmaceutical Interventions (NPIs) are plotted on the left *y* axis, and the 7-day moving average of *F*^(*u*)^ (see Eq. (11)) and the 28-day moving average of *R*_*eff*_ (see Eq. (13)) are plotted on the right *y* axis. For *R*_*eff*_ < 1 (shaded gray regions below the horizontal dotted line), dynamics are tending toward the Disease-Free Equilibrium (DFE)^118^. These areas often overlap with the dates when multiple NPIs are imposed.

We observe that nonpharmaceutical interventions (NPIs, such as mask mandates and mobility restrictions^38^) in both locations were associated with a change in *R*_*eff*_, yielding low *F*^(*u*)^ and confirmed cases. The relaxation of NPIs in Texas, and their complete removal in Aichi, were associated with cases and *F*^(*u*)^ increasing. The gradual rise in the average undocumented contact rate (shown via *F*^(*u*)^), results in the gradual increase in *R*_*eff*_, which yields increasing case counts. This may also indicate that it could be beneficial to keep the NPIs in place even after *R*_*eff*_ < 1 while additionally observing *F*^(*u*)^.

In addition, the effect of individual features on the transition rates (modeled by encoders, see “Methods”) provides insights on the relative contributions of each feature (Table 1). Internet-search trends, survey results for COVID-like symptoms, and weather trends were most strongly associated with fitted contact rates. The encoder weights may also be helpful in comparing NPIs: of the seven considered for the USA, closing schools rank higher than others, suggesting its relative contribution to reducing COVID-19 spread may be greater.

**Table 1:** Model feature importance.

USA model		Japan model	
Time-series features	Median rank	Time-series features	Median rank
NPI schools	1	Mobility changes: residences	1
NPI bar/restaurants	2	Std error of % of survey responders reporting CLI (unweighted)	2
Snowfall (mm)	3	Estimated *R*_*eff*_	3
Mobility index	4	Confirmed cases	4
Cases/total tests	5	Cases mean-to-sum ratio	5
NPI non-essential business	6	State of emergency	6
Mobility samples	7	Mobility changes: transit	7
Average temperature (°C)	8	Std error of % of survey responders reporting CLI (weighted)	8
Confirmed deaths	9	% of survey responders reporting CLI (weighted)	9
Cases mean-to-sum ratio	10	Mobility changes: workplaces	10
**Static features**	**Median rank**	**Static features**	**Median rank**
Ratio of the population over 60	1	Average BMI of males	1
Per capita income	2	Number of H1N1 cases in 2010	2
Mean air quality index	3	Number of new ICU beds	3
Population density	4	Number of clinic beds/100 k population	4
Number of households	5	Number of doctors/100 k population	5

Top ten time-series and top five static feature importance ranks for the average undocumented contact rate in the USA and Japan models.

### Simulating the effects of interventions

Our framework can be used to predict the effect of interventions including NPIs and vaccinations. Overriding NPI features provide forecasts that simulate NPI implementation, and a “vaccinated” compartment transitioning from “susceptible” allows modeling of vaccination strategies, including dosing, effectiveness, and availability. We evaluate counterfactual accuracy by treating past NPIs as counterfactual outcomes, finding MAPE improvements when using observed features as counterfactual scenarios in all but one date tested for cumulative cases and deaths (Supplementary Table 27), as well as evaluating on simulated data (“Counterfactual” section in Supplementary Discussion). We also show uncertainty estimates for counterfactual predictions, allowing less confident estimates to be treated with appropriate caution (Supplementary Figs. 22–35).

**Fig. 7:**
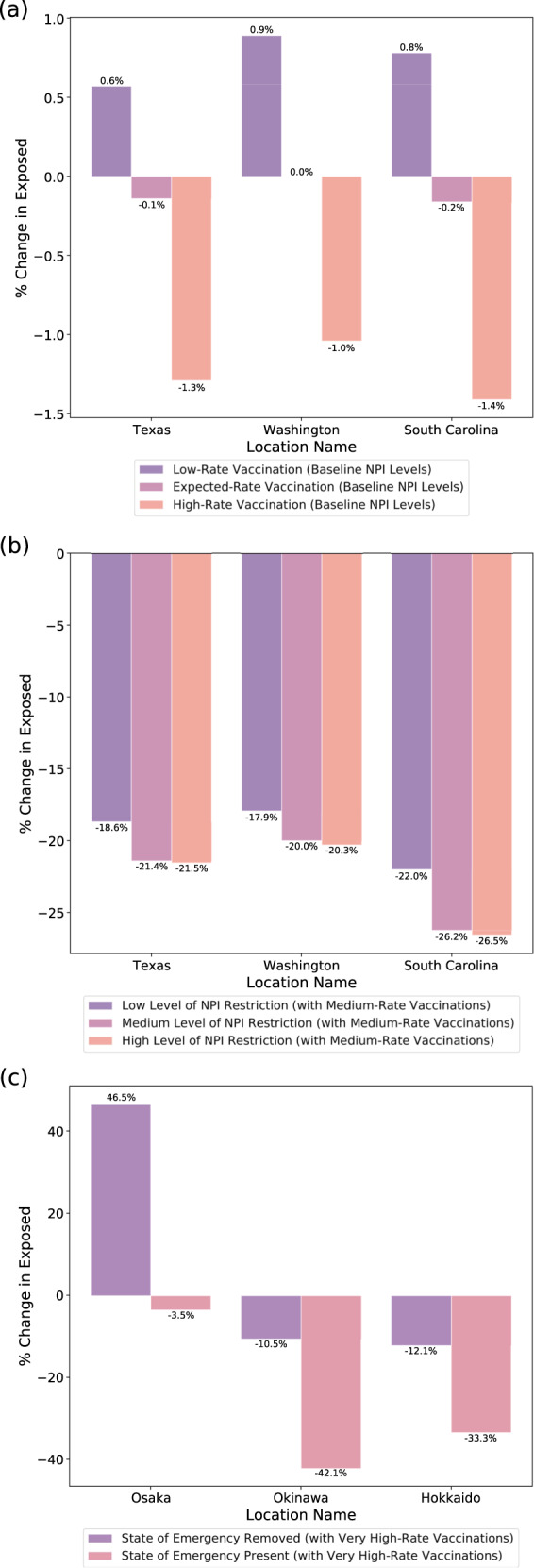
Counterfactual analysis on the count of predicted exposed individuals for different vaccination rates in tandem with NPIs, for the prediction date of March 1, 2021. **a** As shown for the three US states, when vaccination rates (low: 0.2 % population/day, medium: 0.5% population/day, high: 1.0% population/day) are increased compared to the expected baseline, which is obtained from the past 4 weeks’ trend, there is around 1% extra reduction in the predicted exposed. Here, the predicted baseline exposed individual counts are 69,700, 67,600, and 63,700 for Texas, Washington, and South Carolina, respectively. **b** For these US states, when NPI levels are increased while keeping the vaccination rate 0.5% population/day, we observe a significant reduction in the number of predicted exposed, >17% across the three states. The majority of the benefit is coming from the low-level NPI, due to the school closures being the NPI with the largest impact according to the fitted model. **c** In Japan, we show counterfactual analysis assuming a very high vaccination rate (2% population/day), and considering the cases of applying or removing the State of Emergency. Here, the baseline exposed individual counts are 5800, 3800, and 3300 for Osaka, Okinawa, and Hokkaido, respectively. Applying the state of emergency is observed to be effective in reducing the predicted exposed cases. When the State of Emergency is removed in Osaka, despite the high vaccination rate, the predicted exposed cases are observed to go up significantly. Note that in all cases, because of the uncertainty in counterfactual outcome is high—the 95% confidence intervals for baseline and counterfactual outcomes often overlap (see Supplementary Discussion). This suggests that although the statistical significance on the directionality of the change would be high, the statistical significance on the exact amount of change would not be as high. Thus, it is important to stress that if used, the forecasts should be used alongside other information and with the support of epidemiology experts. The percentage change of the exposed individual counts on March 29, 2021 against the forecasted features baseline is shown in both cases.

With counterfactual analysis, consistent with the findings from feature importance, we find that school closures are associated with the highest reduction in predicted exposed counts among all NPIs. The joint application of multiple NPIs (based on the Rand levels^39^) is observed to be much more effective than each individually (Supplementary Tables 28, 29, and 37). We also find that a 7-day delay in applying all NPIs reduced predicted cases by 45% for Japan (Supplementary Table 30). Maintaining NPIs during vaccination reduces predicted cases and deaths by 9% and 9.3%. When increasing the vaccination rate to 1% of the population per day, we observe 65.5% and 16.5% reduction in susceptible and exposed counts for the US, but only a 0.8% drop in predicted cases. We do note that the overall benefit of vaccinations is more visible over longer time horizons, beyond 4 weeks. For reduction of exposed counts in the short-term, keeping particular NPIs (e.g., school closures) in place in tandem with vaccination is beneficial. The observations are also similar for Japan—keeping the State of Emergency in tandem with a high vaccination rate seems highly beneficial (Fig. 7c and Supplementary Table 35). Further results on applying and evaluating counterfactual analysis are available in Supplementary.

### Use cases and the impact of our framework

Our forecasts are released publicly, and thus are available to a wide range of organizations to whom the information may aid decision-making ^40^. While a robust analysis of the impact our forecasts have had is outside the scope of this paper, we conducted a structured survey of those using our forecasts to better understand how they are being used in practice. We found the forecasts, when used alongside other sources of information, were considered helpful across a broad set of areas. Uses included national resource allocation in healthcare and business settings, and implementing social distancing measures at a state-wide level. The full results of this survey, including a series of detailed case studies, are provided in Supplementary Table 56.

## Discussion

We present and evaluate prospectively an AI-augmented calibration procedure for compartmental models in epidemiology that forecasts at a state-, county-, and prefecture level, and provide insights relevant to current and future public health decisions. Coupled with the ability to forecast at a local level (state or county in the USA, prefecture in Japan), our framework creates the opportunity for forecasts to play a greater role in public health.

The forecasts are publicly available^40^, and have been adopted alongside other information by a number of public and private organizations, alongside playing an educational role as a public reminder of the risks of COVID-19. Early case studies are positive, finding that both public and private organizations found the forecasts beneficial to a diverse range of decisions including implementing state-wide social distancing policy measures and national business decisions and healthcare resource allocation. Predictions were used alongside other available information; the forecasts are not intended to be used alone for decision-making. Despite these encouraging anecdotal reports, future quantitative studies are needed to investigate the impact of the forecasts to outcomes. Our framework also provides insight into testing resources. As our compartmental model yields the counts for undocumented and documented infected cases separately, it can suggest locations where undocumented infections are rapidly increasing, and where increasing testing may be beneficial.

Our framework provides insight into the potential consequences of public health decisions around NPIs and vaccination with counterfactual analysis. Via modifications to the proposed compartmental models, it is possible to simulate the efficacy for different vaccine regimens as new vaccines and strategies become available, helping the framework remain relevant as the pandemic evolves. This is important as our understanding of the real-world effectiveness of COVID-19 vaccines and the properties of COVID-19 variants are growing with time. The counterfactual results reinforce the risk of prematurely relaxing NPIs, the importance of prioritizing vaccinations in larger geographies, and to exercise caution even when *R*_*eff*_ is 1, until the force of infection is also reduced.

The survey conducted on organizations using the framework included academic and government organizations that had actively used this counterfactual analysis capability in their decision-making. Though feedback was positive, we note the uncertainty in counterfactual outcomes (which was communicated with these users) is high, and that the model outputs were used alongside other sources of information.

While the performance of the models was overall good, important variations were seen between the USA and Japan, and between different geographic locations. There are several reasons this may be the case. First, cases in Japan are skewed towards a small number of prefectures: 77.7% of Japan prefectures during prospective window had fewer than 30 deaths over the 28-day period, compared to only 0.4% for the USA. This means the model training is dominated by a small number of locations, and that evaluation using MAPE, and incident MAPE in particular, is more strongly influenced by locations with very few cases or deaths when averaging across all of Japans prefectures.

Second, there was less data to learn from due to fewer COVID-19 cases, and Japan ICU and hospitalization data were unavailable for modeling. Data quality, especially for confirmed cases, was not always consistent, including errors such as reporting delays and incorrect data^24–30^. We partially account for this with our preprocessing mechanisms (see “Methods”) and by placing higher weight on confirmed deaths, which are considered to be more accurate than confirmed case counts^10,11^. However underreporting still presents challenges, as is suggested by an analysis of model *R*_*eff*_ (Supplementary Figs. 36–46).

For some states, there is a lag between peaks of *R*_*eff*_ and peaks of daily new confirmed cases, which may reflect increased testing following periods of more rapid but under-reported disease spreading. Finally, our models were optimized for predicting COVID-19-associated cumulative deaths. It is possible that performance for case prediction could be improved if the models were instead optimized for cases instead.

One potential solution to differences in performance is thresholding based on model uncertainty. Because our framework produces well-calibrated predictions, by withholding predictions when the model is uncertain, we can improve the accuracy of the remaining predictions. As each prediction provides an estimate for 4 weeks ahead, any negative impacts of withholding predictions may be relatively small.

While this publication focuses on COVID-19, our approach has value beyond the current pandemic. The underlying principles are not specific to one condition, and evidence of this is seen by the fact that performance did not substantially change during early January 2021 when new variants of SARS-CoV-2 began to emerge in both the USA and Japan. Considering future pandemics our counterfactual analysis supports existing literature on the importance of early interventions^41^, and may also be useful in forward planning. Post-COVID counterfactual analyses may help better understand the relative values of different NPIs, which can be extended to novel and existing epi- and pandemics. Our results also underline the importance of making high-quality data openly available^42^. For future planning, there must be coordinated efforts to make data available before it is needed.

Our work builds on a body of work in epidemiology^43–47^, compartmental models^23,48–54^, and machine learning^55–59^. Recent work has modeled the impact of NPIs such as travel restrictions^60^ in the US^61^ and Europe^62^ with judiciously designed functional forms. By modeling static and time-varying features in compartmental modeling, learning their associations from data in an end-to-end way, our model improves performance while bringing explainable insights^22^. Conversely, several recent publications have attempted direct modeling from features^63–65^. In the absence of high-quality and large-scale historical data, such black-box methods underperform. The inductive bias coming from compartmental modeling has an epidemiological basis and helps the model to fit the data better. Our work differentiates from these, providing a systematic framework to ingest static and time-varying features into compartmental modeling for multi-horizon forecasting with mechanisms to inject scientific priors into the aspects that make the most sense. Our framework’s performance compares favorably with alternatives (Supplementary Tables 15–22, Fig. 3, and Supplementary Figs. 10 and 11) while also providing explainability through feature encoders and counterfactual analysis, and is generalizable to higher geographic granularity and other countries. The ablation studies reported in the Supplements suggest the largest contributing factors to model performance are partial teacher forcing and the integration of learning static and time-varying features.

Our framework has several limitations. It does not differentiate between groups with different levels of risk. For vaccine modeling, differentiating the risk to priority groups (healthcare workers or the elderly population) could aid planning, but the available data do not allow this. Our models treat all locations (i.e. US states/counties and Japan prefectures) in the same way. If an application favors higher accuracy for particular locations rather than the entire country, the loss function can be tailored to overweight particular terms.

It is difficult to evaluate the accuracy of hypothetical counterfactual simulations due to the lack of ground truth. Our approach of evaluating past events and simulated data constitute only partial solutions (see “Counterfactual” section of Supplementary Discussion) as prospective evaluations of counterfactual outcomes present feasibility challenges. Rigorous experimental testing would be required to draw stronger conclusions about counterfactual accuracy. In addition to data quality, the granularity of data sources may also influence performance. One example is mobility data, where to preserve privacy only aggregated data are available. More detailed data including times of day, greater geographic granularity, or demographic factors that may influence the spread of disease could improve performance. Though we find that performance differences across locations reflect variation in case counts rather than systemic biases, the data granularity prevented evaluating subgroup performance at an individual level and biases may still be present. In addition, the uncertainty in counterfactual outcomes is high—the 95% confidence intervals for baseline and counterfactual outcomes often overlap (see Supplementary Discussion). This suggests that although the statistical significance on the directionality of the change would be high, the statistical significance on the exact amount of change would not be as high. Thus, it is important to stress that if used, the forecasts should be used alongside other information and with the support of epidemiology experts.

The COVID-19 pandemic created substantial challenges for governments, businesses, and individuals. In such an event, it is critical to inform decisions with the most accurate, up-to-date information available. We show that a generalizable, explainable AI-augmented epidemiological approach can provide accurate forecasts of the number of confirmed COVID-19 cases, deaths, and hospitalizations during the following 4 weeks, and evidence of its performance in the USA and Japan. Through our approach, we demonstrate that accurate future forecasts of case count are not only possible but are an essential and growing part of public health.

## Methods

### Study design

We conduct a nationwide prospective observational study across the USA and Japan. The USA models were trained from January 22 to November 13, 2020, and the Japan model from January 15 to November 13, 2020. The study concluded on January 9, 2021 in both countries. Each daily forecast in this period was evaluated after 4 weeks had passed. Models were retrained daily prior to each daily forecast. All counties in the USA and all prefectures in Japan were included in the study. The entire populations of both countries were reflected in the public data; US territories were excluded. The study ran for 8 weeks, providing 56 daily forecasts to evaluate. This number was chosen based on a sample size of 43 forecasts being required to detect a 10% difference between predictions of confirmed cases and the observed values at 90% power. Of the 56 forecasts, 7 and 12 were unavailable for the USA and Japan respectively due to errors with the data sources or software bugs preventing a forecast from being produced.

### Data sources and preprocessing: USA model

In this section, we describe the datasets used for our proposed framework in the US. The next section discusses Japan. The preprocessing techniques are largely the same for both countries, while the data sources are country-specific.

The ground truth data for the compartments supervise the forecasting model via training objective functions. We also use “static” (i.e. those with values that do not vary with time) and “time-varying” (i.e. those with values that vary with time) variables as inputs, to extract information from.

The progression of COVID-19 is influenced by a multitude of static variables, including relevant properties of the population, health, environmental, hospital resources, demographics, and socioeconomic indicators. Time-varying variables such as population mobility, hospital resource usage, and public policy decisions can also be important. While variables with predictive signals would be beneficial for more accurate forecasting, indiscriminately incorporating irrelevant variables may hurt performance as it may cause overfitting, if the model fits the relationships to spurious patterns that do not generalize to the future. Therefore, from multiple datasets, we choose variables that may have high predictive signals for the particular transitions in the proposed compartmental model. Those variables are used as feature inputs to the encoders which determine the transition rates. Below, we describe which features we particularly use for the USA and Japan models (also shown in Table 2).

**Table 2:** Features used by the USA and Japan models.

Feature	Transition rates the feature is used for
USA model	
Per capita income	*β*^(*d*)^, *β*^(*u*)^, *η*, *γ*, *ρ*^(*I*, *d*)^, *ρ*^(*I*, *u*)^, *ρ*^(*H*)^, *ρ*^(*C*)^, *ρ*^(*V*)^, *h*, *c*, *v*, *κ*^(*I*, *d*)^, *κ*^*H*^, *κ*^*C*^, *κ*^*V*^
Population density	*β*^(*d*)^, *β*^(*u*)^, *η*, *γ*, *ρ*^(*I*, *d*)^
Households on food stamps	*η*, *ρ*^(*I*, *d*)^, *ρ*^(*I*, *u*)^, *ρ*^(*H*)^, *ρ*^(*C*)^, *ρ*^(*V*)^, *h*, *c*, *v*, *κ*^(*I*, *d*)^, *κ*^*H*^, *κ*^*C*^, *κ*^*V*^
Population	All
Number of households	*β*^(*d*)^, *β*^(*u*)^, *η*, *γ*, *ρ*^(*I*, *d*)^, *ρ*^(*I*, *u*)^, *ρ*^(*H*)^, *ρ*^(*C*)^, *ρ*^(*V*)^, *h*, *c*, *v*, *κ*^(*I*, *d*)^, *κ*^*H*^, *κ*^*C*^, *κ*^*V*^
Population ratio above age 60	*β*^(*d*)^, *β*^(*u*)^, *η*, *γ*, *ρ*^(*I*, *d*)^, *ρ*^(*I*, *u*)^, *ρ*^(*H*)^, *ρ*^(*C*)^, *ρ*^(*V*)^, *h*, *c*, *v*, *κ*^(*I*, *d*)^, *κ*^*H*^, *κ*^*C*^, *κ*^*V*^
Hospital rating scale	*η*, *γ*, *ρ*^(*I*, *d*)^, *ρ*^(*I*, *u*)^, *ρ*^(*H*)^, *ρ*^(*C*)^, *ρ*^(*V*)^, *h*, *c*, *v*, *κ*^(*I*, *d*)^, *κ*^*H*^, *κ*^*C*^, *κ*^*V*^
Available types of hospitals	*η*, *ρ*^(*I*, *d*)^, *ρ*^(*I*, *u*)^, *ρ*^(*H*)^, *ρ*^(*C*)^, *ρ*^(*V*)^, *h*, *c*, *v*, *κ*^(*I*, *d*)^, *κ*^*H*^, *κ*^*C*^, *κ*^*V*^
Hospital patient experience rating	*η*, *ρ*^(*I*, *d*)^, *ρ*^(*I*, *u*)^, *ρ*^(*H*)^, *ρ*^(*C*)^, *ρ*^(*V*)^, *h*, *c*, *v*, *κ*^(*I*, *d*)^, *κ*^*H*^, *κ*^*C*^, *κ*^*V*^
Air quality measures	*β*^(*d*)^, *β*^(*u*)^, *η*, *κ*^(*I*, *d*)^; also for state only: *h*, *c*, *v*, *κ*^*H*^, *κ*^*C*^, *κ*^*V*^ and for county only: *γ*, *ρ*^(*I*, *d*)^, *ρ*^(*I*, *u*)^
Mobility indices	*β*^(*d*)^, *β*^(*u*)^
Weather (state only)	*β*^(*d*)^, *β*^(*u*)^, *γ*, *h*, *ρ*^(*I*, *d*)^, *ρ*^(*I*, *u*)^
Google symptoms search (state only)	*γ*, *h*
Nonpharmaceutical interventions (state only)	*β*^(*d*)^, *β*^(*u*)^
Total tests (state only)	*γ*, *h*
Antigen/antibody tests (state only)	*β*^(*d*)^, *β*^(*u*)^, *γ*, *h*, *ρ*^(*I*, *d*)^, *ρ*^(*I*, *u*)^
Day of the week	*β*^(*d*)^, *β*^(*u*)^, *γ*, *h*
Confirmed per total tests	*β*^(*d*)^, *β*^(*u*)^, *γ*, *h*
Lagged confirmed cases	*β*^(*d*)^, *β*^(*u*)^, *γ*, *h*
Lagged deaths	*β*^(*d*)^, *β*^(*u*)^, *γ*, *h*
Japan model	
Per capita GDP	*β*^(*d*)^, *β*^(*u*)^, *γ*, *ρ*^(*I*, *d*)^, *ρ*^(*I*, *u*)^, *ρ*^(*H*)^, *h*, *κ*^(*I*, *d*)^, *κ*^*H*^
Population density	*β*^(*d*)^, *β*^(*u*)^, *γ*, *ρ*^(*I*, *d*)^, *ρ*^(*I*, *u*)^, *ρ*^(*H*)^, *h*, *κ*^(*I*, *d*)^, *κ*^*H*^
Age distribution	*β*^(*d*)^, *β*^(*u*)^, *γ*, *ρ*^(*I*, *d*)^, *ρ*^(*I*, *u*)^, *ρ*^(*H*)^, *h*, *κ*^(*I*, *d*)^, *κ*^*H*^
Population	All
Healthcare resources (doctors, hospital beds, clinic beds, ICU beds)	*γ*, *ρ*^(*I*, *d*)^, *ρ*^(*I*, *u*)^, *ρ*^(*H*)^, *h*, *κ*^(*I*, *d*)^, *κ*^*H*^
Wellness (past H1N1 infection, BMI, smokers, alcohol consumption)	*β*^(*d*)^, *β*^(*u*)^, *η*, *ρ*^(*I*, *d*)^, *ρ*^(*I*, *u*)^, *ρ*^(*H*)^, *h*, *κ*^(*I*, *d*)^, *κ*^*H*^
Google mobility indices	*β*^(*d*)^, *β*^(*u*)^
State of emergency	*β*^(*d*)^, *β*^(*u*)^
Total tests	*γ*
Symptoms survey results	*β*^(*d*)^, *β*^(*u*)^, *γ*, *ρ*^(*I*, *d*)^, *ρ*^(*I*, *u*)^
Day of week	*γ*, *ρ*^(*I*, *d*)^, *ρ*^(*H*)^, *h*, *κ*^(*I*, *d*)^, *κ*^*H*^
Confirmed mean-to-sum ratio	*β*^(*d*)^, *β*^(*u*)^, *γ*, *η*, *ρ*^(*I*, *d*)^, *ρ*^(*I*, *u*)^, *ρ*^(*H*)^, *h*, *κ*^(*I*, *d*)^, *κ*^*H*^
Deaths mean-to-sum ratio	*β*^(*d*)^, *β*^(*u*)^, *γ*, *η*, *ρ*^(*I*, *d*)^, *ρ*^(*I*, *u*)^, *ρ*^(*H*)^, *h*, *κ*^(*I*, *d*)^, *κ*^*H*^
Discharges	*ρ*^(*H*)^, *h*, *κ*^*H*^
Lagged confirmed cases	*β*^(*d*)^, *β*^(*u*)^, *γ*, *h*
Lagged deaths	*β*^(*d*)^, *β*^(*u*)^, *γ*, *h*

#### Ground truth for compartments

For confirmed and death cases, JHU^66^ is used in our work, similar to other models^45^. They obtain the raw data from the state and county health departments. Because of the rapid progression of the pandemic, past data have often been restated, or the data collection protocols have been changed. We always use the latest version of the data available prior to training or evaluation time. Ground truth data for the hospitalization compartments, including the number of people who are in ICUs or on ventilators are obtained from the COVID Tracking Project^67^.

#### Mobility

Human mobility within a region, for work and personal reasons, may have an effect on the average contact rates^68^. We use time-varying mobility indices provided by Descartes labs at both state- and county-level resolutions^69^. Descartes labs aggregate the movement data of individual cellphone users within a region over a 24-h period. The index is equal to the ratio of the median of the distribution of distance traveled is divided by the “normal” value of the median of the distribution during the period from February 17 to March 7, 2020. These time-series features are encoded to reflect the average contact rates (*β*^(*d*)^, *β*^(*u*)^), at both the state- and county level of geographic resolution.

#### Nonpharmaceutical interventions

Public policy decisions restricting certain classes of population movement or interaction can have a beneficial effect on restricting the progression of the disease^61^, at the state level of geographic resolution. The interventions are presented in six binary-valued time series, indicating when an intervention has been activated in one of six categories–school closures, restrictions on bars and restaurants, movement restrictions, mass gathering restrictions, essential businesses declaration, and emergency declaration^70^. This time-series feature is encoded into the average contact rates (*β*^(*d*)^, *β*^(*u*)^).

#### Demographics

The age of the individual may have a significant outcome on the severity of the disease and the mortality. The Kaiser Family Foundation reports the number of individuals over the age of 60 in US counties (c19hcc-info-ext-data:c19hcc_info_public.Kaiser_Health_demographics_by_Counties_States). We encode the effect of this static feature into the average contact rate (*β*^(*d*)^, *β*^(*u*)^), the diagnosis (*γ*), re-infected (*η*), recovery (*ρ*^(*I*, *d*)^, *ρ*^(*I*, *u*)^, *ρ*^(*H*)^, *ρ*^(*C*)^, *ρ*^(*V*)^) and death rates (*κ*^(*I*, *d*)^, *κ*^*H*^, *κ*^*C*^, *κ*^*V*^), at both the state- and county level of geographic resolution.

#### Historical air quality

Historical ambient air quality in a region can have an effect on the disease spread^71^. We use the BigQuery public dataset that comes from the US Environmental Protection Agency (EPA) that documents historical air quality indices at the county level (bigquery-public-data:epa_historical_air_quality.pm10_daily_summary). This static feature is encoded into the recovery rates (*η*), recovery (*ρ*^(*I*, *d*)^, *ρ*^(*I*, *u*)^, *ρ*^(*H*)^, *ρ*^(*C*)^, *ρ*^(*V*)^) and death rates (*κ*^(*I*, *d*)^, *κ*^*H*^, *κ*^*C*^, *κ*^*V*^), at both the state- and county level of geographic resolution.

#### Socioeconomic indicators

An individual’s economic status, as well as the proximity to other individuals in a region, may have an effect on the rates of infection, hospitalization, and recovery. The proximity can be due to high population density in urban areas, or due to economic compulsions. The USA census—available from census.gov and on BigQuery Public Datasets^72^—reports state- and county-level static data on population, population density, per capita income, poverty levels, households on public assistance (bigquery-public-data:census_bureau_acs.county_2018_5yr and bigquery-public-data:census_bureau_acs.county_2018_1yr). All of these measures affect transitions into the exposed and infected compartments (*β*^(*d*)^, *β*^(*u*)^), as well as the recovery rates (*ρ*^(*I*, *d*)^, *ρ*^(*I*, *u*)^, *ρ*^(*H*)^, *ρ*^(*C*)^, *ρ*^(*V*)^) and death rates (*κ*^(*I*, *d*)^, *κ*^*H*^, *κ*^*C*^, *κ*^*V*^), at both the state- and county level of geographic resolution. In addition, for the state-level model, it also influences the hospitalization rate *h*, ICU rate *c,* and ventilator rate *v*.

#### Hospital resource availability

When an epidemic like COVID-19 strikes a community with such rapid progression, local hospital resources can quickly become overwhelmed^73^. To model the impact, we use the BigQuery public dataset that comes from the Center for Medicare and Medicaid Services, a federal agency within the United States Department of Health and Human Services (bigquery-public-data:cms_medicare.hospital_general_info). These static features are encoded into the diagnosis rate (*γ*), recovery rates (*ρ*^(*I*, *d*)^, *ρ*^(*I*, *u*)^, *ρ*^(*H*)^, *ρ*^(*C*)^, *ρ*^(*V*)^), re-infected rate (*η*) and death rate (*κ*^(*I*, *d*)^, *κ*^*H*^, *κ*^*C*^, *κ*^*V*^), at both the state- and county level of geographic resolution.

#### Symptoms search

Google provides aggregated search data related to specific disease symptoms^74^ for USA states. From these symptoms, we select seven^75^ as features—cough, chills, anosmia, infection, chest pain, fever, and shortness of breath. They are encoded into the diagnosis rate (*γ*) and the hospitalization rate *h*.

#### Weather

The Open Covid Dataset^42^ provides weather features for USA states and counties and Japanese prefectures. These include daily average temperature, rainfall, and snowfall. These are encoded into the contact rates (*β*^(*d*)^, *β*^(*u*)^), the diagnosis rate (*γ*), the hospitalization rate *h*, and selected recovery rates (*ρ*^(*I*, *d*)^, *ρ*^(*I*, *u*)^).

#### Antigen and antibody test counts

Counts for antigen and antibody tests (both positive and negative outcomes) come from the Covid Tracking Project^67^. These time-series features are encoded into the contact rates (*β*^(*d*)^, *β*^(*u*)^), the diagnosis rate (*γ*), the hospitalization rate *h*, and selected recovery rates (*ρ*^(*I*, *d*)^, *ρ*^(*I*, *u*)^).

#### Day of week

The day of the week feature accounts for the cadence of data updates during the week. This feature is used for the average contact rates (*β*^(*d*)^, *β*^(*u*)^), the diagnosis rate (*γ*), and the hospitalization rate *h*.

#### Confirmed cases and deaths

Past confirmed case counts and deaths can have an effect on the current values of these quantities. We include these as time-series features. These are encoded into the average contact rates (*β*^(*d*)^, *β*^(*u*)^), the diagnosis rate (*γ*), and the hospitalization rate *h*.

### Data sources and preprocessing: Japan model

#### Ground truth for compartments

We obtain the ground truth for confirmed cases, deaths, and discharges for Japanese prefectures from the Open Covid Dataset^42^.

#### Mobility

We use six publicly available Google Mobility Reports^76,77^ time series, corresponding to retail and recreation, groceries and pharmacies, parks, transit stations, workplaces, and residential. Each time series is an index reference to a baseline value of 100 from before the pandemic. The number of unique visitors per day to places in each of the six categories is the raw measure. The raw measure is anonymized by adding Laplace noise. For each of the six measures, the reference is constructed by computing the median of the measure for each day of the week in the 5-week range from January 3, 2020 through February 6, 2020. The ratio between the raw measure and the reference is expressed as a percentage and provided as the Google mobility time series. Negative values indicate a decrease in that category of mobility and vice versa. These are encoded into the contact rates (*β*^(*d*)^, *β*^(*u*)^).

#### State of emergency

The state of emergency is a set of Covid-related restrictions^78^ that are applied by the Japanese Government on a per-prefecture basis. Local- and prefecture-level authorities in Japan have wide leeway in the interpretation of the NPI^79^. We manually map the NPI to a binary-valued time series. This is encoded into the contact rates (*β*^(*d*)^, *β*^(*u*)^).

#### Symptoms survey

The Facebook Symptoms Survey dataset^80^ is a dataset of survey responses regarding Covid-like illness, which could have predictive power for the COVID-19 spread and impact. We incorporate features from this dataset encoding them into the contact rates (*β*^(*d*)^, *β*^(*u*)^), diagnosis rate *γ*, and selected recovery rates (*ρ*^(*I*, *d*)^, *ρ*^(*I*, *u*)^).

#### Demographics

We use various prefecture-level demographic features including population, population density^81^, and age distributions^82^ from the 2005 census. These are encoded as continuous variables into the contact rates (*β*^(*d*)^, *β*^(*u*)^), diagnosis rate *γ*, recovery rates (*ρ*^(*I*, *d*)^, *ρ*^(*I*, *u*)^, *ρ*^(*H*)^), hospitalization rate *h*, and selected death rates (*κ*^(*I*, *d*)^, *κ*^*H*^).

#### Socioeconomic indicators

We use prefecture-level per capita GDP from 2000^83^ as an socioeconomic feature. It is encoded into the contact rates (*β*^(*d*)^, *β*^(*u*)^), diagnosis rate *γ*, recovery rates (*ρ*^(*I*, *d*)^, *ρ*^(*I*, *u*)^, *ρ*^(*H*)^), hospitalization rate *h*, and selected death rates (*κ*^(*I*, *d*)^, *κ*^*H*^).

#### Healthcare resources

We incorporate healthcare resource features like the number of doctors, hospital, ICU^84^, and clinic beds^85^, both as raw and as per capita values. These features are encoded into the diagnosis rate *γ*, recovery rates (*ρ*^(*I*, *d*)^, *ρ*^(*I*, *u*)^, *ρ*^(*H*)^), hospitalization rate *h*, and selected death rates (*κ*^(*I*, *d*)^, *κ*^*H*^).

#### Wellness

General health-related features measured before the pandemic, like BMI^86^, alcohol consumption^87^, past H1N1 illness^88^, and smoking habits^89^. These features are encoded into the contact rates (*β*^(*d*)^, *β*^(*u*)^), reinfection rate *η*, recovery rates (*ρ*^(*I*, *d*)^, *ρ*^(*I*, *u*)^, *ρ*^(*H*)^), hospitalization rate *h*, and selected death rates (*κ*^(*I*, *d*)^, *κ*^*H*^).

#### Day of week

The day of the week feature accounts for the cadence of data updates during the week. It is encoded into the diagnosis rate *γ*, selected recovery rates (*ρ*^(*I*, *d*)^, *ρ*^(*H*)^), the hospitalization rate *h*, and selected death rates (*κ*^(*I*, *d*)^, *κ*^*H*^).

#### Confirmed cases and deaths

As for the USA model, the past confirmed cases and deaths can have an effect on their current values. So we include them and their derivative features (mean-to-sum ratios) into both rates.

### Data sources and preprocessing: missing data

For both USA and Japan models, the data sources were provided in real time and were at risk of missing data. To address this for time-varying features, we first apply forward-filling for the future values, and then backward-filling wherever applicable. For static features, we apply median imputation. After imputation, categorical features are mapped to integer labels, and then all features are normalized to be in [0, 1], considering statistics across all locations and timesteps since the beginning of training, January 22, 2020.

### Proposed compartmental model

We adapt the standard SEIR model with some major changes, as shown in Supplementary Fig. 1.

- Undocumented infected and recovered compartments: Recent studies suggest that the majority of the infected people are not detected and they dominate disease progression^15,16,51^ (as the documented ones are either self-isolated or hospitalized). An undocumented infected individual is modeled as being able to spread the disease, until being documented or recovered without being undocumented.
- Hospitalized, ICU, and ventilator compartments**:** We introduce compartments for the people who are hospitalized, in the ICU, or on a ventilator, due to the practical utility to model these^73^ and there are partially available observed data to be used for supervision.
- Partial immunity: To date, there is no scientific consensus on what fraction of recovered cases demonstrates immunity to future infection. Due to reports of reinfection^90^, we model the rate of reinfection from recovered compartments (though our model infers low reinfection rates).
- No undocumented deaths: We assume the published COVID-19 death counts are coming from documented cases, not undocumented.
- Population invariance: We assume that the entire population is invariant, i.e. births and non-COVID-19 deaths are negligible in comparison to the entire population.
- Vaccination: To consider the expected consequences of vaccination strategies, following^91^, we introduce a new “Vaccinated” compartment, which has a transition from the “Susceptible”. Approved COVID-19 vaccines have partial effectiveness^92^. In other words, only a subset of the “Vaccinated” people would actually transition into the “Immune” compartment, while some portion would become susceptible again because of the limited immunity. The two key variables for vaccination strategy—vaccine effectiveness and the number of vaccinated per day—can be adjusted for each location separately. Note that approved vaccines may be injected in one or two doses^92^, and we consider multidose regimes as the number of vaccinated individuals is provided for both first and second doses, and because our framework models the partial immunity between the first and second doses.

The modeled compartments are shown in Table 3. For a compartment *X*, *X*_*i*_[*t*] denotes the number of individuals in that compartment at location *i* and time *t*. We assume a fixed sampling interval of 1 day. *N*[*t*] denotes the total population. Figure 1 describes transition rate variables used to relate the compartments, via the equations (we omit the index *i* for concision):

1 $$\begin{array}{ll}S[t]-S[t\ -\ 1]\ \ =\ -\ ({\beta }^{(d)}{I}^{(d)}[t\ -\ 1]+{\beta }^{(u)}{I}^{(u)}[t\ -\ 1])\frac{S[t\ -\ 1]}{N[t\ -\ 1]}\\\qquad\qquad\qquad\qquad\quad +\ \eta ({R}^{(d)}[t\ -\ 1]\ +\ {R}^{(u)}[t\ -\ 1])-Y[t-1],\end{array}$$

2 $$E[t]-E[t\ -\ 1]\ \ =({\beta }^{(d)}{I}^{(d)}[t\ -\ 1]+{\beta }^{(u)}{I}^{(u)}[t\ -\ 1])\frac{S[t\ -\ 1]}{N[t\ -\ 1]}-\alpha E[t\ -\ 1],$$

3 $${I}^{(u)}[t]-{I}^{(u)}[t\ -\ 1]\ \ =\alpha E[t\ -\ 1]-({\rho }^{(I,u)}+\gamma ){I}^{(u)}[t\ -\ 1],$$

4 $${I}^{(d)}[t]-{I}^{(d)}[t\ -\ 1]\ \ =\gamma {I}^{(u)}[t\ -\ 1]-({\rho }^{(I,d)}+{\kappa }^{(I,d)}+h){I}^{(d)}[t\ -\ 1],$$

5 $${R}^{(u)}[t]-{R}^{(u)}[t\ -\ 1]\ \ ={\rho }^{(I,u)}{I}^{(u)}[t\ -\ 1]-\eta {R}^{(u)}[t\ -\ 1],$$

6 $$\begin{array}{ll}{R}^{(d)}[t]-{R}^{(d)}[t\ -\ 1]\ \ ={\rho }^{(I,d)}{I}^{(d)}[t\ -\ 1]+{\rho }^{(H)}(H[t\ -\ 1]-C[t\ -\ 1])\\\qquad\quad-\eta {R}^{(d)}[t\ -\ 1],\end{array}$$

7 $$\begin{array}{l}H[t]-H[t\ -\ 1]\ \ =h{I}^{(d)}[t\ -\ 1]-({\kappa }^{(H)}+{\rho }^{(H)})(H[t\ -\ 1]-C[t\ -\ 1])\\\qquad-{\kappa }^{(C)}(C[t\ -\ 1]-V[t\ -\ 1])-{\kappa }^{(V)}V[t\ -\ 1],\end{array}$$

8 $$\begin{array}{l}C[t]-C[t\ -\ 1]\ \ =c(H[t\ -\ 1]-C[t\ -\ 1])-({\kappa }^{(C)}+{\rho }^{(C)}+v)\\\qquad(C[t\ -\ 1]-V[t\ -\ 1])-{\kappa }^{(V)}V[t\ -\ 1],\end{array}$$

9 $$V[t]-V[t\ -\ 1]\ \ =v(C[t\ -\ 1]-V[t\ -\ 1])-({\kappa }^{(V)}+{\rho }^{(V)})V[t\ -\ 1],$$

10 $$\begin{array}{ll}D[t]-D[t\ -\ 1]\ \ ={\kappa }^{(V)}V[t\ -\ 1]+{\kappa }^{(C)}(C[t\ -\ 1]-V[t\ -\ 1])\\\qquad+{\kappa }^{(H)}\ \ (H[t\ \ -\ \ 1]-C[t\ -\ 1])+{\kappa }^{(I,d)}{I}^{(d)}[t\ -\ 1],\end{array}$$

**Table 3:** Modeled compartments.

Compartment	Description	Compartment	Description
*S*	Susceptible	*R*^(*u*)^	Recovered undocumented
*E*	Exposed	*H*	Hospitalized
*I*^(*d*)^	Infected documented	*C*	In intensive care unit (ICU)
*I*^(*u*)^	Infected undocumented	*V*	On ventilator
*R*^(*d*)^	Recovered documented	*D*	Death
*Z*^(1)^	First-dose vaccinated	*Z*^(1)^	Second-dose vaccinated
*Y*	Immune with vaccination	*L*	Re-susceptible after vaccination

The transition rate variables that define the relationship between the compartments are obtained with machine-learning models that input the corresponding features, as explained in the section “Machine-learning methods”.

#### Force of infection

The force of infection is defined as the measure of the rate at which susceptible individuals become infected^93^—for undocumented infected, formulated as:

11 $${F}^{(u)}={\beta }^{(u)}* {I}^{(u)}/N,$$

and documented infected, formulated as:

12 $${F}^{(d)}={\beta }^{(d)}* {I}^{(d)}/N.$$

#### Effective reproductive number

Using the Next-Generation Matrix method^94^ on the proposed compartmental model, the effective reproductive number can be derived as^22^:

13 $${R}_{{eff}}=\frac{{\beta }^{(d)}\gamma +{\beta }^{(u)}({\rho }^{(I,d)}+{\kappa }^{(I,d)}+h)}{(\gamma +{\rho }^{(I,u)})\cdot ({\rho }^{(I,d)}+{\kappa }^{(I,d)}+h)}.$$

#### Integration of vaccination

We consider two-dose vaccination strategy^95^ and define the first-dose effectiveness function as:

14 $${\pi }^{(1)}[\tau ]=\min ({\pi }_{max}^{(1)},{\pi }_{max}^{(1)}\cdot \tau /{T}_{\pi }^{(1)}),$$

and the second-dose effectiveness function as:

15 $${\pi }^{(2)}[\tau ]=\min ({\pi }_{max}^{(2)},{\pi }_{max}^{(1)}+({\pi }_{max}^{(2)}-{\pi }_{max}^{(1)})\cdot \tau /{T}_{\pi }^{(2)}),$$

where ${\pi }_{max}^{(1)}$ and ${\pi }_{max}^{(2)}$ are the maximum effectiveness values of the first and second vaccines, and ${T}_{\pi }^{(1)}$ and ${T}_{\pi }^{(2)}$ are the time periods defined for effectiveness ramp-up. We use ${\pi }_{max}^{(1)}=0.921$, ${\pi }_{max}^{(2)}=0.945$, ${T}_{\pi }^{(1)}={T}_{\pi }^{(2)}=14$ days^95^. Given the cumulative counts for first-dose-vaccinated *Z*^(1)^ (also including the s econd-dose-vaccinated) and second-dose-vaccinated *Z*^(2)^, we obtain the count for immune with vaccination as:

16 $$\begin{array}{ll}Y[t]=&\mathop{\sum }\limits_{\tau =0}^{{T}_{\pi }^{(1)}-1}({\pi }^{(1)}[\tau ]\cdot \left(\right.{Z}^{(1)}[t-\tau ]-{Z}^{(1)}[t-\tau -1])+{\pi }_{max}^{(1)}\cdot {Z}^{(1)}[t-{T}_{\pi }^{(1)}]+.\\ &\mathop{\sum }\limits_{\tau =0}^{{T}_{\pi }^{(2)}-1}\left(\right.({\pi }^{(2)}[\tau ]-{\pi }_{max}^{(1)})\cdot ({Z}^{(2)}[t-\tau ]-{Z}^{(2)}[t-\tau -1])\\& +({\pi }^{(2)}-{\pi }_{max}^{(1)})\cdot {Z}^{(2)}[t-{T}_{\pi }^{(2)}]-L[t-1],\end{array}$$

where *L*[*t*] is re-susceptible after vaccination due to the lost immunity and obtained as:

17 $$L[t]=L[t-1]+Y[t]/{T}_{L},$$

where *T*_*L*_ denotes the timescale for losing immunity. We use *T*_*L*_ = 180 days^95^. Note that the impact of *L*[*t*] is often negligible as the forecasting horizon of our framework is much shorter.

### Machine-learning methods

#### Time-varying modeling of variables

Instead of using static rate variables across time to model compartment transitions, there should be time-varying functions that map them from known observations. For example, if mobility decreases over time, the *S* → *E* transition should reflect that. Consequently, we propose replacing all static rate variables with learnable functions that output their value from the related static and time-varying features at each location and timestep. We note that the learnable encoding of variables still preserves the inductive bias of the compartmental modeling framework while increasing the model capacity via learnable encoders.

#### Interpretable encoder architecture

In addition to making accurate forecasts, it is valuable to understand how each feature affects the model. Such explanations greatly help users from the healthcare and public sector to understand the disease dynamics better, and also help model developers to ensure the model is learning appropriate dynamics via sanity checks with known scientific studies. To this end, we adopt a generalized additive model^96^ for each variable *v*_*i*_ from Table 2 based on features X(*v*_*i*_, *t*) at different time *t*. The features we consider include (i) the set of static features ${{{\mathscr{S}}}}$, such as population density, and (ii) ${\{f[t-j]\}}_{f\in {{{{\mathscr{F}}}}}_{i},j = 1,\ldots ,k}$ the set of time-varying features ${{{{\mathscr{F}}}}}_{i}$ with the observation from *t* − 1 to *t* − *k*, such as mobility. Omitting individual feature interactions and applying additive aggregation, we obtain

18 $${v}_{i}[t]={v}_{i,L}+({v}_{i,U}-{v}_{i,L})\cdot \sigma \left(c+{b}_{i}+{{{{\bf{w}}}}}^{\top }\,{{\mbox{X}}}\,({v}_{i},t)\right),$$

where *v*_*i*,*L*_ and *v*_*i*,*U*_ are the lower and upper bounds of *v*_*i*_ for all *t*, *c* is the global bias, *b*_*i*_ is the location-dependent bias. **w** is the trainable parameter, and *σ*() is the sigmoid function to limit the range to [*v*_*i*,*L*_, *v*_*i*,*U*_], which is important to stabilize training and avoid overfitting. We use *v*_*i*,*L*_ = 0 for all variables, *v*_*i*,*U*_ = 1 for *β*, 0.2 for *α*, 0.001 for *η*, and 0.1 for others. We note that although Eq. (18) denotes a linear decomposition for *v*_*i*_[*t*] at each timestep, the overall behavior is still highly nonlinear due to the relationships between compartments.

#### Feature forecasting

The challenge of using Eq. (18) for future forecasting is that some time-varying features are not available for the entire forecasting horizon. Assume we have the observations of features and compartments until *T*, and we want to forecast from *T* + 1 to *T* + *τ*. To forecast *v*_*i*_[*T* + *τ*], we need the time-varying features *f*[*T* + *τ* − *k*: *T* + *τ* − 1] for $f\in {{{{\mathscr{F}}}}}_{i}$, but some of them are not observed when *τ* > *k*. To solve this issue, we propose to forecast *f*[*T* + *τ* − *k*: *T* + *τ* − 1] based on their own past observations until *T*, which is a standard one-dimensional time-series forecasting for a given feature *f* at a given location. To this end, we employ a forecasting model based on XGBoost^97^ classification or regression (based on the number of categories for the feature) with time-series input features, including the lagged features of the past 7 days plus the 2 weeks ago, and mean/max in the windows of sizes of 3, 5, 7, 14, and 21 days. We note that treating feature forecasting as a univariate time-series modeling problem in this way, has limitations. There are indeed codependencies between different features (e.g., current school closure intervention affects the future value of the mobility), and also the values for the compartments (e.g., increase in the number of deaths would affect the future value of the mobility). Utilizing these has the potential to capture more information, however, given the small amount of past data, overfitting is a concern, and this way of limiting the feature forecasting model capacity and relying on implicit modeling of such dependencies, can prevent overfitting to spurious patterns from other features, and generalize better. For most features, our proposed XGBoost-based forecasting model seems to yield highly accurate forecasts, and also the impact of more accurate feature forecasting on compartmental forecasts becomes marginal beyond some point.

#### Information-sharing across locations

Some aspects of the disease dynamics are location-dependent while others are not. In addition, data availability varies across all *L* locations—there may be limited observations to learn the impact of a feature. A model able to learn both location-dependent and independent dynamics is desirable. Our encoders in Eq. (18) partially capture location-shared dynamics via shared **w** and the global bias *c*. To allow the model to capture remaining location-dependent dynamics, we introduce the local bias *b*_*i*_. A challenge is that the model could ignore the features by encoding all information into *b*_*i*_ during training. This could hurt generalization as there would not be any information-sharing on how static features affect the outputs across locations. Thus, we introduce a regularization term *L*_*ls*_ = *λ*_*ls*_∑_*i*_∣*b*_*i*_∣^2^ to encourage the model to leverage features and *c* for information-sharing instead of relying on *b*_*i*_. Without *L*_*ls*_, we observe that the model would use the local bias more than the encoded features, and suffers from poorer generalization.

#### Learning from partially available observations

Fitting would have been easy with observations for all compartments, however, we only have access to some. For instance, *I*^(*d*)^ is not given in the ground truth of USA data but we instead have, *Q*, the total number of confirmed cases, that we use to supervise *I*^(*d*)^ + *R*^(*d*)^ + *H* + *D*. Note that *R*^(*ud*)^, *I*^(*ud*)^, *S*, *E* are not given as well. Formally, we assume the availability of the observations *Y*[*T*_*s*_: *T*], for *Y* ∈ {*Q*, *H*, *C*, *V*, *D*, *R*^(*d*)^}, and consider forecasting the next *τ* days, $\hat{Y}[T+1:T\ +\ \tau ]$. Note that we use the notation *S*_*i*_[*T*_*s*_: *T*] to denote all timesteps between *T*_*s*_ (inclusive) and *T* (inclusive).

#### Fitting objective

There is no direct supervision for training encoders, while they should be learned in an end-to-end way via the aforementioned partially available observations. We propose the following objective for range [*T*_*s*_, *T*_*e*_]:

19 $$\begin{array}{l}{L}_{fit}[{T}_{s}\ :\ {T}_{e}]=\mathop{\sum}\limits_{Y\in \{Q,H,C,V,D,{R}^{(d)}\}}\ {{{{\boldsymbol{\lambda }}}}}_{Y}\mathop{\sum }\limits_{t={T}_{s}}^{{T}_{e}-\tau }\mathop{\sum }\limits_{i=1}^{\tau }\frac{{\mathbb{I}}(Y[t\ +\ i])}{{\sum }_{j}{\mathbb{I}}(Y[j])}\\\cdot Y[j]\cdot q(t\ +\ i\ -\ {T}_{s};z)\cdot L(Y[t\ +\ i],\hat{Y}[t\ +\ i]).\end{array}$$

${\mathbb{I}}(\cdot )\in \{0,1\}$ indicates the availability of the *Y* to allow the training to focus only on available observations. *L*(, ) is the loss between the ground truth and the predicted values (e.g., *ℓ*_2_ or quantile loss), and **λ**_*Y*_ are the important weights to balance compartments due to its robustness (e.g., *D* is much more robust than others). Lastly, $q(t;z)=\exp (t\cdot z)$ is a time-weighting function (when *z* = 0, there is no time weighting) to allow the fitting to favor more recent observations and *z* is a hyperparameter. During training, we randomly sample *T*_*e*_ from [*T*_*s*_, *T* − *τ* − 1] and for fine-tuning, we set *T*_*e*_ as *T*.

#### Constraints and regularization

Given the limited dataset size, overfitting is a concern for training high-capacity encoders. In addition to limiting the model capacity with the epidemiological inductive bias, we further apply regularization to improve generalization to unseen future data. An effective regularization is constraining the effective reproduction number *R*_*eff*_ (see Eq. (13)). There is rich literature in epidemiology on *R*_*eff*_ to give us good priors on the range of the number should be. For a reproduction number *R*_*eff*_[*t*] at time *t*, we consider the regularization

$${L}_{{R}_{{eff}}}[{T}_{s}:T]=\mathop{\sum }\nolimits_{t = {T}_{s}}^{T}\exp \left({({R}_{{eff}}[t]-R)}_{+}\right),$$

where *R* is a prespecified soft upper bound. The regularization favors the model with *R*_*eff*_ in a reasonable range in addition to good absolute forecasting numbers. In our experiments, we use *R* = 5. Without this regularization term, we have observed epidemiologically unreasonable *R*_*eff*_ values (mostly in the form of sharp peaks), especially in the early days of the pandemic when the amount of training data is small. We have empirically observed that this regularization term helps smoothing such peaks, makes the training behavior a bit more robust, and eventually yields slight improvements in forecasting accuracy. Also, we integrate the prior knowledge of disease dynamics via directional penalty regularization: (1) if the mobility increases, the average contact rates (*β*^(*d*)^, *β*^(*ud*)^) will increase, (2) as the NPIs or state of emergency (SoE) introduced, the average contact rates (*β*^(*d*)^, *β*^(*ud*)^) will decrease. The directional penalty regularization is denoted as

$${L}_{dir}={\sum }_{i\in {{\mbox{Mobility}}}}\max (-{w}_{i},0)+{\sum }_{j\in {{\mbox{NPIs or SoE}}}}\max ({w}_{j},0),$$

Last, ignoring the perturbation of a small local window, the trend of the forecast should be usually smooth. One commonly used smoothness constraint is penalizing the first-order difference, velocity, which is defined as *v*_*Y*_[*t*] = (*Y*[*t*] − *Y*[*t* − *k*])/*k*. The first-order constraint encourages *v*_*Y*_[*t*] ≈ *v*_*Y*_[*t* − 1], which causes linear forecasting, and cannot capture the rapidly growing cases. Instead, we relax the smoothness to be on the second-order difference, acceleration, which is defined as *a*_*Y*_[*t*] = *v*_*Y*_[*t*] − *v*_*Y*_[*t* − 1]. The regularization is

$${L}_{acc}[{T}_{s}\ :\ T]=\mathop{\sum}\limits_{Y\in \{Q,D\}}\mathop{\sum }\limits_{t={T}_{s}+1}^{T}{({a}_{Y}[t]-{a}_{Y}[t-1])}^{2}.$$

The final objective function is

20 $$\begin{array}{l}{{{\mathscr{L}}}}({T}_{s},T)={L}_{fit}[{T}_{s}:T]\ +\ {\lambda }_{ls}\cdot {L}_{ls}\ +\ {\lambda }_{{R}_{{eff}}}\cdot {L}_{{R}_{{eff}}}[{T}_{s}:T]\ \\+\ {\lambda }_{dir}\cdot {L}_{dir}\ +\ {\lambda }_{acc}\cdot {L}_{acc}[{T}_{s}:T],\end{array}$$

where *L*_*ls*_ = ∑_*i*_∣*b*_*i*_∣^2^.

#### Partial teacher forcing

The compartmental model generates the future propagated values from the current timestep. During training, we have access to the observed values for *Y* ∈ {*Q*, *H*, *C*, *V*, *D*, *R*^(*d*)^} at every timestep, which we could condition the propagated values on, commonly known as teacher forcing^59^ to mitigate error propagation. At inference time, however, ground truth beyond the current timestep *t* is unavailable, hence the predictions should be conditioned on the future estimates. Using solely ground truth to condition propagation would create a train-test mismatch. In the same vein of past research to mix the ground truth and predicted data to condition the projections on^98^, we propose partial teacher forcing, simply conditioning $(1-\nu {\mathbb{I}}\{Y[t]\})Y[t]\ +\ \nu {\mathbb{I}}\{Y[t]\}$) $\hat{Y}[t]$, where ${\mathbb{I}}\{Y[t]\}\in \{0,1\}$ indicates whether the ground truth *Y*[*t*] exists and *ν* ∈ [0, 1]. In the first stage of training, we use teacher forcing with *ν* ∈ [0, 1], which is a hyperparameter. For fine-tuning, we use *ν* = 1 to unroll the last *τ* steps to mimic the real forecasting scenario.

#### Model fitting and selection

The training pseudocode is presented in Algorithm 1. We split the observed data into training and validation, where the validation size is *τ*. *τ* should be smaller or equal to the forecasting horizon at inference. Although having it equal minimizes the train-test mismatch, it uses more recent samples for model selection instead of training, thus, as the optimal value, we choose it to be half of the forecasting horizon. We use the training data for optimization of the trainable degrees of freedom, collectively represented as **θ**, while the validation data is used for early stopping and model selection. Once the model is selected, we fix the hyperparameters and run fine-tuning on joint training and validation data, to not waste valuable recent information by using it only for model selection. For optimization, we use RMSProp as it is empirically observed to yield lower losses compared to other algorithms and providing the best generalization performance. We implement Algorithm 1 in TensorFlow at state- and county levels, using *ℓ*_2_ loss for point forecasts. We employ^99^ for hyperparameter tuning (including all the loss coefficients, learning rate, and initial conditions) with the objective of optimizing for the best validation loss, with 400 trials and we use *F* = 100 fine-tuning iterations. We choose the compartment weights *λ*^*D*^ = *λ*^*Q*^ = 0.1, *λ*^*H*^ = 0.01 and ${\lambda }^{{R}^{(d)}}={\lambda }^{C}={\lambda }^{V}=0.001$. We observe our results to be not highly sensitive to these hyperparameters. At county granularity, we do not have published data for *C* and *V*, so, we remove them along with their connected variables.

#### Quantile regression

Besides point forecasts, prediction intervals could be helpful for healthcare and public policy planners, to consider a range of possible scenarios. To obtain prediction intervals, we adapt the quantile regression method, following the state-of-the-art multi-horizon forecasting approaches for well-calibrated uncertainity^58,100^. Our framework allows the capability of modeling prediction interval forecasts, for which we replacing the L2 loss with weighted interval loss (WIS)^31^ in Eq. (19) and mapping the scalar propagated values to the vector of quantile estimates. For this mapping, we use the features $Y[t]/\hat{Y}[t]$ and ${\mathbb{I}}\{Y[t]\}$ for *T* − *τ* ≤ *t* ≤ *T* − 1. We obtain the quantiles applying a linear kernel on these features, followed by ReLU and cumulative summation (to guarantee monotonicity of quantiles) and lastly normalization (to match the median to the input scalar point forecast from the proposed framework). In our framework, we output the *α*-quantile *Q*_*α*_[*t*] at time *t*, where *α* ∈ [0.01, 0.05, 0.1, …, 0.95, 0.99]. WIS loss is a discretization of continuous ranking probability score^31^.

### Counterfactual analysis

Counterfactual analysis into the forecasting horizon involves replacing the forecasted values for selected NPIs, mobility features or vaccination rates with their counterfactual counterparts. Replacement or overriding happens in the forecasting horizon. For a detailed exposition, see Supplementary Discussion.

**Algorithm 1** Detailed pseudocode for model training



### Evaluations

#### Metrics

We use two kinds of metrics: the first computes metrics per geographic region (county, state, prefecture) then aggregates via averaging. The second aggregates predictions across geography then compute metrics at a country level. These two metrics allow quantification of accuracy at the location granularity of interest, as well as detection of any machine-learning biases, such as systematic underprediction or overprediction. We define per location absolute error metric as $A{E}_{i}(T,\tau )=| \hat{{D}_{i}}[T+\tau ]-{D}_{i}[T+\tau ]|$ and absolute percentage error metric a $AP{E}_{i}(T,\tau )=100\cdot I\{{D}_{i}[T+\tau ] > 0\}(| \hat{{D}_{i}}[T+\tau ]/{D}_{i}[T+\tau ]| -1)$, where $\hat{{D}_{i}}[t]$ denote the predicted variable at time *t* for location *i*, and *D*_*i*_[*t*] is the corresponding ground truth. *I*{} is an indicator function that we use to eliminate 0 counts from division (this occurs for a small subset of Japanese prefectures and US counties in earlier days for the deaths). As the average absolute error metrics across all locations, we consider the average of per location error metrics: $MAE(T,\tau )=\frac{1}{L}\mathop{\sum }\nolimits_{i = 1}^{L}A{E}_{i}(T,\tau )$ and $MAPE(T,\tau )=\frac{\mathop{\sum }\nolimits_{i = 1}^{L}AP{E}_{i}(T,\tau )}{\mathop{\sum }\nolimits_{i = 1}^{L}I\{{D}_{i}[T+\tau ] > 0\}}$. At the country level, we first aggregate counts and predictions and define aggregated absolute errors as $AAE(T,\tau )=| \mathop{\sum }\nolimits_{i = 1}^{L}\hat{{D}_{i}}[T+\tau ]-\mathop{\sum }\nolimits_{i = 1}^{L}{D}_{i}[T+\tau ]|$ and $AAPE(T,\tau )=| \mathop{\sum }\nolimits_{i = 1}^{L}\hat{{D}_{i}}[T+\tau ]/\mathop{\sum }\nolimits_{i = 1}^{L}{D}_{i}[T+\tau ]-1|$.

#### Data versions for evaluations

There are significant restatements of the past observed counts in the data. For prospective evaluations, we use the data at the end of the *τ* day forecasting horizon. To mimic the prospective evaluations as much as possible with the retrospective evaluations, we use the reported numbers on the prediction date for training (although later we know the restated past ground truth), and the reported numbers *τ* days after prediction date for evaluation.

#### Performance comparisons

To account for the correlations between timesteps when considering the accuracy of fitting to a time series, the two-sided DM test^101^ is used to compare our models’ forecasts to those of other models from “covid19-forecast-hub” (https://covid19forecasthub.org/). The 4-week-ahead forecasts are compared using MAE and MAPE after they had been averaged across all the locations for the dates when both of the models produced forecasts. This is reported for cumulative and incident deaths, cumulative and incident cases, and the number of people admitted to the hospital. The *P* values from the tests are adjusted using the Holm–Bonferroni method^102^ to account for the multiple comparisons and KPSS tests^103^ are run on the differences to examine stationarity over time.

#### Subgroup analysis

To account for potential confounders and biases, a subset of demographic variables are chosen for further investigation. Age, sex, income, population density, and ethnicity are investigated for both the USA and Japanese models. These variables are chosen based on known biases in how COVID-19 has affected different demographics^104–108^, as well as how they may affect healthcare access^109^. To investigate these relationships differences changes in the MAPE of the forecasts were compared to the demographics from each geographical region (counties for the USA and prefectures for Japan). An initial assessment is done by grouping the counties into quartiles of the demographic variable of interest and calculating the MAPE across the groups. Kendall’s Tau^110^ is used to quantify the relationship between the variable of interest and the MAPE for each geographical region. Because of the presence of confounding variables and potential multicollinearity between the variables of interest, partial correlation is performed using all of the other variables as features.

#### Uncertainty analysis

For model reliability when used by human experts, we also investigate using epistemic model uncertainty as a confidence metric of forecasts. Well-calibrated estimates of uncertainty are important for being able to make more reliable predictions^111–115^. To this extent, we investigate the relationship between epistemic model uncertainty and the accuracy of forecasts by simulating the scenario of deciding whether or not to withhold each day’s 28-day forecast based on model disagreement, with the goal of demonstrating an optional feature that could allow the system to identify and withhold the predictions that are likely to be the most erroneous.

For each day in the retrospective period, we train an ensemble of *k* = 5 models and use their disagreement as a metric of uncertainty^111^. Producing 28-day forecasts from each model, we consider two values for each location: (1) the metric performance of the single best model over the 28-day forecast (in MAE or MAPE for cumulative values), and (2) the variance in predictions across the *k* models for each day, averaged over the 28-day period. In Fig. 4, we plot the average prediction variance versus the MAE metric performance on predicted confirmed cases, for all release dates and all prefectures in Japan. We can see that higher model disagreement correlates with worse metric performance.

For each location, we then collect the set of average predicted variances for all release dates and compute ten quantiles at the [10%, 20%, …, 90%, 100%] levels. We then decide on which forecast dates to withhold predictions by thresholding the average predicted variance based on the value at each quantile, yielding ten groups of release dates per location. We average the metric performance across the dates in each group and average over locations. This yields average performance at ten quantiles of uncertainty values, which we visualize in the form of a rejection diagram^116,117^ in Fig. 4.

From this, we can see that withholding forecasts on days with higher average prediction variance can lead to better average metric performance over the remaining forecasts.

Overall, we find that on average, the reliability of our framework could be improved through the proposed method of model uncertainty quantification by allowing the system to identify and withhold predictions that are likely to be the most erroneous at prediction time without access to ground truth labels (additional discussion in Supplementary Note: Uncertainty Analysis). Importantly, this is an optional feature, with prediction withholding being just one example of an action that the system could take upon identifying an unreliable prediction. Other actions could include bringing an expert human into the loop to further analyze the predictions for that day or simply adding a note to the released predictions to indicate that they may be less reliable.

#### Uncertainty in counterfactual predictions

As with the forecast uncertainty, the prediction of counterfactuals is subject to disagreement between independently trained models. We describe the process to obtain the uncertainty in the counterfactuals and some observations in the Supplementary Discussion.

### Reporting summary

Further information on research design is available in the Nature Research Reporting Summary linked to this article.

## Supplementary information

Reporting Summary

Supplementary Information

## Data Availability

The data used for the training, validation, and test sets are publicly available. All data were collected entirely from openly available sources. The dashboard showing our forecasts can be accessed from https://g.co/covidforecast. The following websites can be used to access the time-series data used in this study for the US model: https://github.com/CSSEGISandData/COVID-19 (state and county confirmed cases, deaths); https://github.com/descarteslabs/DL-COVID-19 (state and county mobility, mobility index and mobility samples); https://covidtracking.com/ (state hospitalized currently, state hospitalized cumulative counts, state hospitalized increase, state in ICU currently, state in ICU cumulative, state on ventilator currently, state total test results); https://coronadatascraper.com/#home (county hospitalized cumulative counts); http://goo.gle/covid19symptomdataset (state and county symptoms search tracker); https://dsd.c19hcc.org/ (state NPI information). The static data for the US can be found at https://data.cms.gov/provider-data/dataset/xubh-q36u (state and county hospital resources); https://www.census.gov/programs-surveys/acs (state and county population and demographics data); https://www.epa.gov/ (state and county air quality data). For the Japan model time-series data, the following datasets were used: https://cloud.google.com/blog/products/data-analytics/publicly-available-covid-19-data-for-analytics (confirmed cases and deaths); https://covidmap.umd.edu/api.html (symptoms data); https://github.com/google-research/open-covid-19-data/blob/master/data/exports/google_mobility_reports/Regions/2020_JP_Region_Mobility_Report.csv (mobility data); https://crisis.ecmonet.jp/(ventilator use); https://github.com/graphy-covidjp/graphy-covidjp.github.io (vaccine data). The static data for Japan can be found at https://stats-japan.com/t/kiji/13400 (demographics); https://www.mhlw.go.jp/english/database/db-hh/2-2.html (healthcare resources and wellness data); https://www.mhlw.go.jp/toukei/list/20-21.html (living conditions); https://stats.oecd.org/ (socioeconomic indicators); http://idsc.nih.go.jp/idwr/sokuho/index.html (H1N1 data); https://www.mhlw.go.jp/bunya/kenkou/eiyou/h24-houkoku.html (nutrition data); https://japan.kantei.go.jp/ongoingtopics/index.html (state of emergency data).

## Code availability

We make use of several open-source libraries to conduct our experiments, namely the machine-learning framework TensorFlow (https://github.com/tensorflow/tensorflow). While the entire system relies on proprietary libraries and we are unable to publicly release the entire codebase, we make available code for key pieces of the model details, training, and evaluation at https://github.com/google-research/google-research/tree/master/covid_epidemiology.
