# Supplementary material for: A prospective evaluation of AI-augmented epidemiology to forecast COVID-19 in the USA and Japan
Source: NPJ Digit Med. 2021 Oct 8;4:146. doi: 10.1038/s41746-021-00511-7 (PMC8501040; doi:10.1038/s41746-021-00511-7)
Supplement: Supplementary file 1 — Reporting Summary [file 41746_2021_511_MOESM1_ESM.pdf]

nature research

Corresponding author(s): Sercan Arik, Joseph Ledsam

Last updated by author(s): Aug 12, 2021

Reporting Summary

Nature Research wishes to improve the reproducibility of the work that we publish. This form provides structure for consistency and transparency in reporting. For further information on Nature Research policies, see our Editorial Policies and the Editorial Policy Checklist.

Statistics

For all statistical analyses, confirm that the following items are present in the figure legend, table legend, main text, or Methods section.

n/a	Confirmed	
☐	☒	The exact sample size (*n*) for each experimental group/condition, given as a discrete number and unit of measurement
☐	☒	A statement on whether measurements were taken from distinct samples or whether the same sample was measured repeatedly
☐	☒	The statistical test(s) used AND whether they are one- or two-sided<br>*Only common tests should be described solely by name; describe more complex techniques in the Methods section.*
☐	☒	A description of all covariates tested
☐	☒	A description of any assumptions or corrections, such as tests of normality and adjustment for multiple comparisons
☐	☒	A full description of the statistical parameters including central tendency (e.g. means) or other basic estimates (e.g. regression coefficient) AND variation (e.g. standard deviation) or associated estimates of uncertainty (e.g. confidence intervals)
☐	☒	For null hypothesis testing, the test statistic (e.g. *F*, *t*, *r*) with confidence intervals, effect sizes, degrees of freedom and *P* value noted<br>*Give P values as exact values whenever suitable.*
☒	☐	For Bayesian analysis, information on the choice of priors and Markov chain Monte Carlo settings
☒	☐	For hierarchical and complex designs, identification of the appropriate level for tests and full reporting of outcomes
☐	☒	Estimates of effect sizes (e.g. Cohen's *d*, Pearson's *r*), indicating how they were calculated

Our web collection on statistics for biologists contains articles on many of the points above.

Software and code

Policy information about availability of computer code

Data collection	Data was collected from a wide range of public, openly available sources. A detailed description of each source, and where it can be downloaded, is provided in the methods.
Data analysis	The networks used the TensorFlow library with custom extensions. Analysis was performed with custom code written in Python 2.7. Please see the manuscript methods section for more detail.

For manuscripts utilizing custom algorithms or software that are central to the research but not yet described in published literature, software must be made available to editors and reviewers. We strongly encourage code deposition in a community repository (e.g. GitHub). See the Nature Research guidelines for submitting code & software for further information.

Data

Policy information about availability of data

All manuscripts must include a data availability statement. This statement should provide the following information, where applicable:

- Accession codes, unique identifiers, or web links for publicly available datasets
- A list of figures that have associated raw data
- A description of any restrictions on data availability

The data used for the training, validation and test sets is publicly available. All data was collected entirely from openly available sources. The access information for all sources is provided in the methods section. The dashboard showing our forecasts can be accessed from https://g.co/covidforecast.

Field-specific reporting

Please select the one below that is the best fit for your research. If you are not sure, read the appropriate sections before making your selection.

☒ Life sciences ☐ Behavioural & social sciences ☐ Ecological, evolutionary & environmental sciences

For a reference copy of the document with all sections, see nature.com/documents/nr-reporting-summary-flat.pdf

Life sciences study design

All studies must disclose on these points even when the disclosure is negative.

Sample size	The USA models were trained from January 22nd to November 13th 2020, and the Japan model from January 15th to November 13th 2020. The study concluded on January 9th in both countries. Each daily forecast in this period was evaluated after 28 days had passed. Models were retrained daily prior to each daily forecast. All counties in the USA and all prefectures in Japan were included in the study. The study ran for 8 weeks, providing 56 daily forecasts to evaluate. This number was chosen based on a sample size of 43 forecasts being required to detect a 10% difference between predictions of confirmed cases and the observed values at 90% power.
Data exclusions	US Territories were excluded from analysis due to a lack of available data with which to train the models. There were no other exclusions.
Replication	In this study, the model provided future forecasts on 56 days prospectively, and 147 days retrospectively before this. The performance is reported in Figure 3 of the manuscript.
Randomization	This study evaluates the prospective performance of a forecasting model for COVID-19 associated deaths and cases. The model was trained retrospectively and applied to all available data during the prospective evaluation. Performance was compared between retrospective and prospective evaluations. Randomization between these two periods was no feasible or appropriate, and there were not two groups during the prospective period to randomize between.
Blinding	Data collection was performed independently of the authors. All authors were blinded to any allocations or decisions made during data collection.

Reporting for specific materials, systems and methods

We require information from authors about some types of materials, experimental systems and methods used in many studies. Here, indicate whether each material, system or method listed is relevant to your study. If you are not sure if a list item applies to your research, read the appropriate section before selecting a response.

Materials & experimental systems

n/a	Involved in the study	
☒	☐	Antibodies
☒	☐	Eukaryotic cell lines
☒	☐	Palaeontology and archaeology
☒	☐	Animals and other organisms
☒	☐	Human research participants
☒	☐	Clinical data
☒	☐	Dual use research of concern

Methods

n/a	Involved in the study	
☒	☐	ChIP-seq
☒	☐	Flow cytometry
☒	☐	MRI-based neuroimaging
