# Supplementary material for: A prospective evaluation of AI-augmented epidemiology to forecast COVID-19 in the USA and Japan
Source: NPJ Digit Med. 2021 Oct 8;4:146. doi: 10.1038/s41746-021-00511-7 (PMC8501040; doi:10.1038/s41746-021-00511-7)
Supplement: Supplementary file 2 — Supplementary Information [file 41746_2021_511_MOESM2_ESM.pdf]

Supplementary Information
A prospective evaluation of AI-enabled epidemiology to forecast COVID-19 in the USA and Japan

The aim of this supplementary information is to provide further information to support the claims made in the Article "*A prospective evaluation of AI-enabled epidemiology to forecast COVID-19 in the USA and Japan*". It is the hope of the authors that by providing these supplementary results and associated discussion that the conclusions of the letter are strengthened, along with the reproducibility of the work.

We present the following supplementary material:

- Supplementary Note 1 provides detail on the proposed model and the changes implemented during the prospective period.
- Supplementary Note 2 provides supplementary methods and evaluation details, including full reporting of performance for individual locations and model comparison with baselines.
- Supplementary Note 3 provides additional results from counterfactual simulation experiments in the USA and Japan.
- Supplementary Note 4 provides model explainability graphs for each US State and Japanese prefecture, and detailed results on feature ranking from the encoders.
- Supplementary Note 5 provides results from ablation studies.
- Supplementary Note 6 provides the full results of the model fairness analysis.
- Supplementary Note 7 provides an analysis of and justification for using model uncertainty to reject low-confidence forecasts.
- Supplementary Note 8 provides anecdotal case studies from organisations who used the model in practice during the prospective study.

1 Supplementary Note 1: Proposed compartmental model

Figure 1 overviews our compartmental model. The transitions between the compartments are formulated in the Methods section.

Major model changes during the prospective evaluation period

The prospective trial began after 28 day predictions were publicly launched in the US and Japan. As the models were public and being used by policy makers, some elements of the model were altered during the trial to ensure the model was stable and accurate. This maintenance work was essential to address the changing requirements of the model, such as changing data sources and to improve stability.

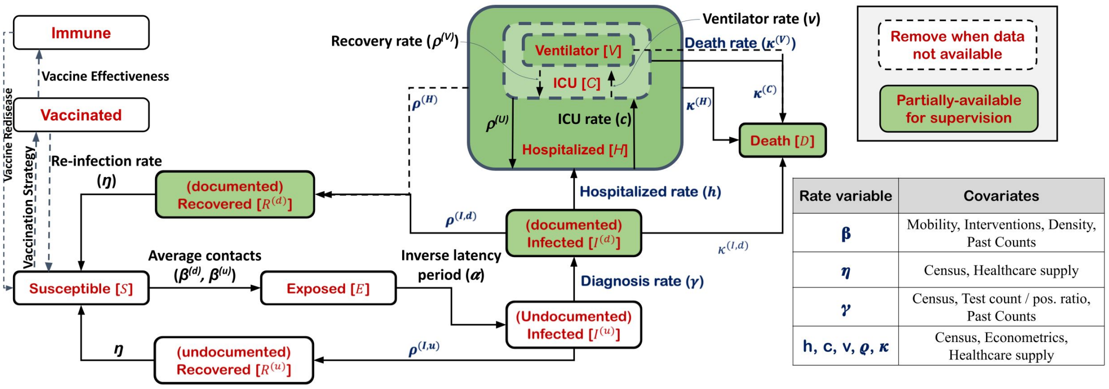

Rate variable	Covariates
β	Mobility, Interventions, Density, Past Counts
η	Census, Healthcare supply
γ	Census, Test count / pos. ratio, Past Counts
h, c, v, ϱ, κ	Census, Econometrics, Healthcare supply

Supplementary Figure 1 | Framework architecture and modeled compartments. Modeled compartments and the corresponding features are show, with the legend on the right. Each rectangle represents a compartment, while each connection between rectangles represents a transition between compartments. The rates are named in the legend. Rectangles shaded in green represent compartments that are partially supervised.

The changes reflect the real world experience of deploying and maintaining an AI model, especially in a rapidly-changing pandemic environment. All changes only influence performance after they were implemented; the prospective performance we report for each date is based on the prediction of our model made on that date.

- **11/11/2020:** 28 day forecast model was launched in the USA and Japan.
- **11/20/2020:** Testing related features were converted to per capita quantitites from raw counts.
- **12/06/2020:** (i) Additional features for antibody/antigen counts and weather data were added. (ii) The earlier version of the model was using the predicted features instead of actual ones during training, it was switched to actual ones so that the encoder weights can learn how to better work with the true features.
- **12/09/2020:** (i) Number of iterations for quantile regression was increased. (ii) The partial teacher forcing coefficient for the Japan model was reduced for undocumented infected as it was overpredicting the new confirmed cases due to overpredicted diagnosis rate.
- **12/23/2020:** (i) Directional penalty regularization was added for mobility and NPI features for US State model. (ii) The cutoff for R_{eff} regularization was converted to a tunable hyperparameter. (iii) Search symptoms data was integrated as a feature. (iv) Quantile regression training loss coefficients were re-optimized to get better validation WIS values. (v) Random sampling of training range was introduced (See Algorithm 1) to reduce train-test mismatch.
- **12/31/2020:** (i) Quantile normalization was switched from mean to median, to ensure the mid-quantile better matches the point forecasts.

- **01/08/2021:** (i) Directional penalty regularization was added for US County and Japan models. (ii) Quantile regression training loss coefficients were reoptimized to get better validation WIS values for the Japan model.
- **02/26/2021:** Two-dose vaccine modeling was introduced reflecting the most recent experimental results[1] (see Methods) to be used in baseline forecasts and counterfactual analysis.

2 Supplementary Note 2: Performance Evaluations

Performance across different time windows

Supplementary Figure 2 shows the model performance in MAPE across different time windows, demonstrating prediction over alternative periods to the 28 days we focus on is possible.

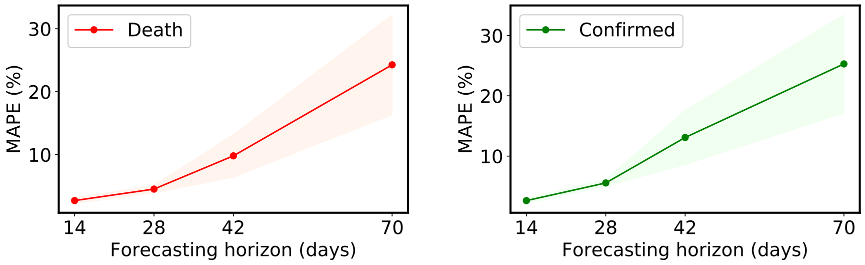

Supplementary Figure 2 | MAPE (%) vs. forecasting horizon, averaged across different prediction dates with the 95 % confidence intervals (shaded), for death (left) and confirmed (right). For each forecasting horizon, we retrain the model after changing the corresponding τ value in Algorithm 1. Forecasting different horizons may improve utility, as having insights farther into the future can allow better policy and health planning. On the other hand, as there is increasing uncertainty into the future and the value of observed features decay over time, it is expected that the model accuracy should get worse. We choose 28-day horizon considering the trade-off between the accuracy and utility. For forecast values beyond 4 weeks, we often observe significant degradation in accuracy.

Prospective performance for all US states and Japanese prefectures

We provide the 4-week predictive performance in APE (see Methods) of both the USA and Japan model at multiple time points during the prospective trial. Performance is shown for forecasts of cumulative deaths (Supplementary Table 1) and cases (supplementary Table 2)) for all 51 US states. For Japan, performance is shown for cumulative deaths (Supplementary Table 3) and cases (supplementary Table 4) all 47 Japan prefectures.

Supplementary Table 1 | MAPE (%) for 4-week forecasts of cumulative deaths for all the US states on different prediction dates.

Forecast Date ⟹<br>State Name ⇓	2020-11-15	2020-11-22	2020-11-29	2020-12-06	2020-12-13	2020-12-20	2020-12-27	2021-01-03	2021-01-10
Alabama	4.6	2.1	9.2	3.6	8.8	9.5	7.6	17.8	23.0
Alaska	1.6	37.4	38.8	22.0	25.8	27.1	1.7	9.0	5.0
Arizona	0.5	4.9	6.3	5.1	6.1	6.7	7.9	8.1	3.1
Arkansas	2.1	1.2	5.0	11.7	1.9	6.0	1.3	5.3	10.7
California	1.7	6.0	8.1	5.4	5.5	4.1	10.5	8.4	0.8
Colorado	8.2	5.1	3.8	3.1	8.2	16.1	7.3	7.1	12.5
Connecticut	0.0	2.1	1.4	2.0	3.8	2.1	0.4	1.9	5.3
Delaware	1.7	5.8	6.8	4.7	6.1	8.8	8.3	1.4	3.3
District Of Columbia	0.9	4.4	2.6	1.0	1.9	0.7	0.1	0.8	1.1
Florida	6.5	1.0	0.8	2.6	3.6	0.6	1.4	0.4	2.4
Georgia	15.8	0.7	1.1	3.0	4.7	0.1	4.8	8.5	9.3
Hawaii	7.7	7.7	9.9	5.0	7.6	1.0	5.9	18.5	9.2
Idaho	1.3	3.3	0.6	3.3	12.5	13.4	7.3	2.6	7.9
Illinois	3.3	2.1	1.6	4.8	2.0	0.2	1.8	2.4	6.5
Indiana	0.8	3.7	0.5	1.3	0.3	0.7	0.1	5.4	2.6
Iowa	4.7	4.2	13.2	15.3	5.5	3.0	1.9	4.0	4.1
Kansas	16.8	14.0	10.2	8.6	8.4	2.3	1.4	9.6	14.2
Kentucky	0.0	2.9	1.8	1.2	1.8	1.1	1.8	7.9	6.8
Louisiana	2.5	1.5	0.9	2.5	1.7	0.0	2.1	0.5	4.4
Maine	1.1	6.8	7.1	10.7	20.0	0.4	8.5	4.7	13.6
Maryland	4.6	2.8	1.4	0.2	1.2	2.4	1.9	1.3	4.8
Massachusetts	2.2	1.3	0.6	1.2	2.8	3.8	2.0	1.2	4.4
Michigan	4.1	0.1	3.7	3.3	3.8	1.7	1.2	1.7	1.8
Minnesota	8.3	9.9	1.6	5.2	2.7	2.5	2.4	0.2	3.7
Mississippi	2.9	2.7	0.6	2.7	0.7	1.4	3.5	1.8	0.5
Missouri	2.5	7.7	14.8	8.6	7.9	0.2	8.4	3.3	1.5
Montana	10.3	10.4	8.6	1.8	3.2	8.0	10.2	10.4	2.6
Nebraska	5.8	12.1	0.1	2.9	19.5	7.5	0.3	4.6	10.5
Nevada	5.0	5.2	3.6	2.2	4.5	1.0	3.8	7.7	2.1
New Hampshire	1.2	3.7	1.3	4.7	0.6	7.2	1.8	7.6	9.8
New Jersey	0.2	1.0	0.1	0.4	0.4	0.8	1.7	0.8	3.1
New Mexico	3.8	11.1	18.9	8.0	1.6	0.9	2.8	0.1	4.4
New York	3.2	2.1	2.5	2.9	2.6	2.1	0.8	2.7	2.9
North Carolina	0.5	1.8	1.0	1.1	2.4	0.0	3.1	3.9	1.1
North Dakota	15.0	4.4	2.0	6.8	6.0	4.3	4.8	2.2	1.3
Ohio	3.3	5.0	5.2	5.6	4.4	0.9	3.5	1.8	1.5
Oklahoma	3.5	4.9	0.3	1.7	0.2	3.4	8.9	7.0	2.3
Oregon	13.1	12.3	3.3	4.4	7.1	9.9	10.6	5.2	1.0
Pennsylvania	9.9	3.4	4.1	2.2	0.4	0.4	4.6	3.4	0.4
Rhode Island	3.5	3.3	1.4	1.4	7.2	10.6	10.0	1.0	12.4
South Carolina	3.5	0.9	2.5	1.9	0.3	2.3	4.0	3.7	1.5
South Dakota	10.1	1.4	1.9	3.8	7.5	0.7	2.8	1.3	0.4
Tennessee	1.0	1.5	5.3	6.7	1.6	4.8	1.5	6.3	1.7
Texas	2.2	0.5	1.2	1.5	3.7	4.8	4.8	5.4	1.3
Utah	12.1	16.6	22.4	29.0	28.4	2.0	5.4	7.3	6.1
Vermont	3.7	11.1	17.4	11.8	6.1	13.9	4.5	12.9	41.0
Virginia	5.5	1.0	1.1	3.2	1.1	1.4	0.1	2.4	1.8
Washington	11.1	8.7	9.2	11.1	3.4	8.9	11.5	2.4	12.9
West Virginia	6.3	10.8	2.0	0.0	2.8	2.6	2.1	4.0	11.7
Wisconsin	12.8	7.4	5.0	3.8	1.4	2.3	1.1	4.5	0.5
Wyoming	20.0	25.4	14.1	5.0	7.6	11.4	21.6	9.7	6.5

Supplementary Table 2 | MAPE (%) for 4-week forecasts of cumulative confirmed cases for all the US states on different prediction dates.

Forecast Date ⟹<br>State Name ⇓	2020-11-15	2020-11-22	2020-11-29	2020-12-06	2020-12-13	2020-12-20	2020-12-27	2021-01-03	2021-01-10
Alabama	10.4	8.3	5.9	6.5	5.2	14.9	2.9	0.8	8.0
Alaska	1.5	12.7	18.5	12.9	23.7	14.8	2.7	9.9	8.4
Arizona	14.6	6.7	4.4	3.7	4.9	5.3	11.8	6.5	20.7
Arkansas	5.4	2.1	2.3	4.9	1.7	3.3	4.7	0.1	13.8
California	22.1	17.1	16.6	5.9	22.5	31.0	8.8	2.5	7.7
Colorado	7.6	30.7	26.2	9.1	17.0	10.0	1.6	3.8	13.4
Connecticut	11.2	0.2	0.4	1.1	4.0	14.0	32.4	19.0	37.5
Delaware	14.3	7.5	1.4	2.8	4.7	15.6	5.9	2.9	16.5
District Of Columbia	14.2	7.2	1.8	7.4	4.2	11.4	5.7	6.5	2.5
Florida	12.8	0.5	0.3	1.6	1.4	2.4	2.0	5.6	22.1
Georgia	4.9	13.2	10.7	8.9	2.1	0.1	4.5	0.3	3.7
Hawaii	3.0	9.1	9.1	2.2	1.7	1.1	4.7	3.8	14.9
Idaho	5.5	7.7	5.7	0.5	10.4	9.6	18.0	5.9	11.7
Illinois	1.3	32.3	14.3	0.9	3.2	6.5	4.2	0.9	12.4
Indiana	5.0	18.5	11.5	0.1	10.5	11.5	2.4	3.8	14.9
Iowa	12.8	33.9	13.5	1.8	1.3	0.1	3.0	2.7	11.1
Kansas	10.4	7.2	14.9	7.9	2.9	4.4	13.9	13.5	24.0
Kentucky	10.9	8.0	10.0	5.1	13.5	3.4	5.8	5.7	17.8
Louisiana	13.7	6.1	0.9	13.7	6.4	2.9	8.0	5.1	14.3
Maine	1.3	6.4	18.5	8.7	22.0	13.5	0.7	12.3	38.7
Maryland	9.3	2.3	3.8	2.9	2.5	1.3	4.9	5.6	12.5
Massachusetts	22.1	5.6	3.1	9.7	1.3	25.4	1.4	3.1	8.5
Michigan	9.9	37.9	35.6	19.3	2.5	6.9	8.0	6.9	10.9
Minnesota	1.8	41.9	39.7	12.6	10.4	6.9	0.9	2.1	8.0
Mississippi	10.3	4.0	7.1	2.8	8.2	4.1	4.5	0.6	11.6
Missouri	7.0	14.7	4.6	2.2	3.5	8.1	2.1	4.4	12.6
Montana	0.8	24.3	21.4	0.9	6.7	3.6	4.9	3.4	9.2
Nebraska	5.4	25.1	19.9	9.6	3.9	3.6	3.0	3.7	12.3
Nevada	11.2	1.2	8.2	5.0	10.3	12.6	1.6	4.3	11.6
New Hampshire	9.6	1.6	3.9	9.5	5.3	27.8	16.2	9.0	13.1
New Jersey	1.5	7.6	6.0	4.2	13.3	2.6	10.1	9.7	6.4
New Mexico	9.7	33.9	36.5	12.0	8.7	4.4	2.4	3.2	14.6
New York	21.2	4.8	5.5	1.6	15.1	9.1	8.6	3.1	4.8
North Carolina	10.6	6.6	4.3	1.1	7.3	4.5	6.6	2.5	9.7
North Dakota	3.9	24.8	14.5	3.4	3.3	2.6	1.1	2.8	3.6
Ohio	8.9	12.9	17.8	3.8	20.7	21.5	1.6	7.1	13.0
Oklahoma	6.7	9.9	10.3	3.3	0.8	4.8	9.8	5.0	10.2
Oregon	1.1	11.1	15.8	24.8	15.3	13.1	1.4	2.5	13.2
Pennsylvania	13.2	4.9	9.6	8.1	0.4	13.3	2.3	4.9	11.6
Rhode Island	18.7	0.4	1.9	2.3	10.0	20.4	22.8	8.5	26.4
South Carolina	7.3	6.4	8.8	3.5	3.6	3.0	5.4	3.9	2.8
South Dakota	4.0	17.9	11.8	2.2	3.3	0.4	2.1	6.0	5.7
Tennessee	10.5	2.3	11.4	16.3	7.2	12.2	4.0	2.9	9.6
Texas	6.7	2.6	5.0	0.8	7.8	0.8	5.1	4.6	0.8
Utah	2.3	4.4	15.4	22.0	22.9	1.3	7.9	2.1	19.7
Vermont	18.6	5.2	1.6	21.2	0.9	24.4	13.3	11.0	24.0
Virginia	11.9	6.1	0.9	2.2	0.5	3.5	1.1	0.8	11.3
Washington	1.8	3.1	11.1	12.6	13.7	4.6	11.1	27.5	19.9
West Virginia	5.3	11.7	10.3	1.0	9.0	11.2	2.4	21.8	27.8
Wisconsin	7.3	26.1	13.6	2.5	0.9	6.3	1.4	1.0	9.3
Wyoming	5.2	37.5	28.6	6.1	5.3	6.8	2.1	2.1	12.0

**Supplementary Table 3 | ** MAPE (%) for 4-week forecasts of cumulative deaths for all the Japan prefectures on different prediction dates.

Forecast Date ⟹<br>Prefecture Name ⇓	2020-11-15	2020-11-22	2020-11-30	2020-12-06	2020-12-13	2020-12-20	2020-12-27	2021-01-03	2021-01-10
Aichi	14.2	9.2	12.6	19.3	25.8	27.2	11.0	1.4	3.9
Akita	26.1	67.2	46.2	19.0	24.0	13.2	117.2	115.3	37.4
Aomori	25.7	20.7	7.6	3.7	4.4	11.9	3.2	9.8	4.3
Chiba	2.1	1.2	11.0	12.9	4.1	2.8	12.0	14.7	8.2
Ehime	7.9	24.6	3.0	36.6	26.0	14.8	16.0	10.7	48.4
Fukui	6.2	13.7	9.8	4.3	5.9	7.1	9.4	31.6	29.9
Fukuoka	0.1	2.8	4.6	6.3	12.5	15.4	9.0	17.4	14.5
Fukushima	7.1	8.0	51.5	51.9	52.3	49.8	0.3	2.8	13.4
Gifu	9.9	13.1	31.7	39.7	40.9	45.2	21.3	33.5	39.2
Gunma	12.0	7.7	10.7	31.4	25.6	31.4	13.3	2.9	35.0
Hiroshima	3.4	19.3	17.8	66.6	37.1	38.6	5.2	27.2	16.0
Hokkaido	20.3	22.0	19.8	17.7	3.8	6.5	5.8	9.7	11.1
Hyogo	0.1	8.8	26.9	42.6	34.0	15.6	10.5	17.7	25.4
Ibaraki	9.6	17.4	1.1	13.6	8.2	0.5	0.3	15.0	6.8
Ishikawa	1.8	1.6	4.6	4.9	0.9	2.4	0.6	2.0	8.6
Iwate	54.4	61.9	70.3	74.5	39.5	6.7	13.9	0.9	8.8
Kagawa	8.0	11.8	4.2	40.5	5.4	30.7	48.1	65.9	45.7
Kagoshima	43.8	17.0	13.2	0.6	39.6	11.9	10.3	1.7	6.4
Kanagawa	0.6	3.8	11.1	11.1	6.3	6.0	2.9	17.2	8.7
Kochi	4.1	12.9	25.7	21.8	82.5	36.0	15.4	16.2	0.3
Kumamoto	18.9	37.2	7.7	8.9	39.4	52.0	35.6	32.3	39.4
Kyoto	8.4	17.6	12.1	12.1	26.0	24.0	7.0	20.5	15.8
Mie	13.2	3.5	5.1	18.2	24.5	20.9	17.3	14.0	49.0
Miyagi	57.8	63.5	3.7	13.6	20.1	13.5	14.3	23.2	14.4
Miyazaki	46.2	36.0	23.7	9.8	39.4	47.9	33.8	53.8	265.5
Nagano	117.7	76.5	20.6	44.8	34.5	31.1	25.1	29.2	39.7
Nagasaki	10.0	17.5	35.1	16.4	45.6	66.7	55.8	36.6	27.7
Nara	49.2	20.5	21.7	19.7	15.2	15.5	14.4	11.5	11.2
Niigata	–	88.5	13.3	1.5	211.6	23.4	3.4	17.9	40.4
Oita	10.2	43.1	41.8	39.3	15.2	39.3	46.4	42.8	12.1
Okayama	100.3	140.1	46.7	10.3	5.8	26.6	122.9	7.6	37.5
Okinawa	1.2	0.7	3.2	5.4	1.6	4.6	4.0	8.9	13.8
Osaka	19.1	16.5	0.7	19.0	18.9	24.4	7.2	4.2	25.6
Saga	31.8	26.9	22.9	11.7	44.7	17.9	28.3	20.9	38.0
Saitama	7.7	12.4	0.6	2.6	0.7	1.5	1.7	12.2	15.4
Shiga	23.1	21.5	1.9	4.7	23.3	32.1	20.4	10.5	1.1
Shizuoka	58.4	12.5	39.3	0.5	17.9	23.6	17.7	7.5	5.5
Tochigi	50.7	151.7	7.6	28.7	12.8	53.3	46.1	36.0	30.7
Tokushima	7.4	9.8	4.7	2.9	1.5	0.8	22.4	33.8	23.8
Tokyo	3.4	3.0	10.1	9.4	15.6	10.1	10.1	207.6	16.0
Tottori	–	–	–	–	13.4	63.5	43.9	19.6	102.4
Toyama	1.8	7.8	5.2	5.0	3.1	3.1	5.0	5.1	34.5
Wakayama	26.3	33.1	51.8	92.8	11.0	21.3	29.0	21.8	11.2
Yamagata	78.9	7.7	34.3	47.0	36.8	51.7	45.9	4.0	35.3
Yamaguchi	234.8	386.7	68.0	1.5	49.1	15.0	9.6	59.5	74.6
Yamanashi	3.6	4.3	3.3	4.1	3.7	0.9	7.1	9.9	11.1

Supplementary Table 4 | MAPE (%) for 4-week forecasts of cumulative confirmed cases for all the Japan prefectures on different prediction dates.

Forecsst Date ⟹ Prefecture Name ⇓	2020-11-15	2020-11-23	2020-11-30	2020-12-06	2020-12-13	2020-12-20	2020-12-27	2021-01-03	2021-01-10
Aichi	16.8	3.3	7.1	7.4	4.0	0.2	2.2	2.9	7.5
Akita	12.8	5.0	5.9	25.0	42.3	51.4	32.8	20.8	12.0
Aomori	7.2	11.4	1.5	2.1	31.8	38.5	22.4	3.4	7.4
Chiba	7.9	3.9	9.3	18.2	18.3	15.6	16.7	4.1	30.5
Ehime	48.1	44.3	21.6	4.2	42.3	53.0	43.2	36.2	10.1
Fukui	7.1	16.1	11.6	0.9	15.8	23.9	20.3	17.9	9.7
Fukuoka	8.6	10.1	13.5	10.1	11.5	12.9	3.1	30.2	0.9
Fukushima	6.6	5.7	26.0	35.0	42.8	45.4	19.8	15.5	2.4
Gifu	28.6	25.4	25.6	10.3	45.4	47.9	33.7	3.4	21.0
Gunma	29.9	22.1	13.9	7.6	35.0	38.6	21.3	23.9	3.7
Hiroshima	41.3	53.7	53.2	57.1	55.8	26.7	63.1	5.5	18.8
Hokkaido	30.7	87.5	51.2	11.1	24.2	3.5	7.4	1.8	8.7
Hyogo	24.9	5.6	47.6	7.5	5.8	1.8	9.9	12.3	12.2
Ibaraki	25.1	22.8	12.1	11.2	32.5	41.9	34.0	23.9	4.4
Ishikawa	3.6	1.1	3.9	8.8	23.6	25.7	11.9	5.1	0.1
Iwate	35.0	14.9	5.9	27.4	21.2	25.7	4.9	0.3	8.0
Kagawa	29.6	6.8	28.7	18.6	51.1	59.8	29.6	36.0	9.2
Kagoshima	6.5	14.6	22.0	29.4	24.5	31.1	25.7	23.9	10.8
Kanagawa	6.8	4.8	4.8	23.4	20.3	17.4	6.2	6.2	24.2
Kochi	54.3	64.8	70.5	20.9	46.6	32.0	12.4	5.9	1.2
Kumamoto	1.4	3.6	26.6	31.4	45.5	50.1	10.3	3.6	28.4
Kyoto	16.3	15.7	14.3	31.2	43.2	44.1	35.0	0.0	2.1
Mie	25.9	2.4	8.6	0.8	25.8	33.8	25.5	28.8	0.9
Miyagi	8.0	11.2	23.2	24.7	37.1	37.9	20.2	10.4	0.4
Miyazaki	28.2	5.3	13.9	10.6	49.4	53.9	46.1	51.1	47.2
Nagano	19.0	9.4	2.7	8.2	34.2	45.3	36.2	30.3	11.7
Nagasaki	8.0	17.1	21.0	55.7	64.6	68.8	58.1	30.7	98.8
Nara	19.3	9.8	16.3	10.0	33.0	34.4	16.6	6.5	0.3
Niigata	25.3	15.0	11.3	25.3	34.4	41.0	28.6	10.5	2.6
Oita	64.1	32.7	12.0	37.3	33.2	36.5	33.6	20.2	7.2
Okayama	5.3	41.7	8.1	36.4	55.0	42.5	24.1	18.0	22.1
Okinawa	2.9	1.6	4.5	7.7	16.2	20.8	15.7	13.6	4.8
Osaka	0.7	42.4	50.6	8.7	10.3	3.4	13.4	13.6	8.6
Saga	14.1	10.6	12.9	14.3	32.3	44.6	42.8	39.6	10.8
Saitama	9.3	5.1	10.3	3.5	1.9	2.3	5.8	1.1	18.1
Shiga	0.8	9.1	11.2	25.2	42.0	45.8	19.8	4.1	1.3
Shimane	6.5	9.8	14.2	7.4	24.7	21.7	26.6	0.5	4.1
Shizuoka	50.1	12.3	65.3	19.7	33.1	36.7	3.9	19.0	8.1
Tochigi	26.4	13.5	27.8	27.2	62.0	66.2	51.5	16.1	50.9
Tokushima	9.0	24.4	8.9	3.2	16.2	33.0	41.2	42.4	27.6
Tokyo	1.3	17.2	3.3	4.6	9.4	17.1	4.7	67.1	46.8
Tottori	0.1	2.0	14.0	12.2	58.6	60.2	27.1	11.1	9.1
Toyama	1.7	10.8	2.8	10.7	29.7	36.6	27.8	21.6	12.6
Wakayama	12.3	4.0	14.0	32.8	24.5	31.7	31.2	22.3	1.7
Yamagata	49.0	49.3	36.5	11.9	34.6	31.2	5.1	3.9	2.2
Yamaguchi	1.7	44.2	4.1	20.9	35.5	42.4	37.4	28.4	22.7
Yamanashi	19.8	16.4	16.0	14.3	34.2	42.6	33.4	18.8	1.1

Prospective comparisons to other models in the USA

We perform performance comparisons to the models submitted to "covid19-forecast-hub" (https://covid19forecasthub.org/). The repository accepts submissions for 4 targets (i) N-week ahead cumulative death, (ii) N-week ahead incremental death, (iii) N-week ahead incremental confirmed cases and (iv) N-day ahead incremental hospitalized. The submissions are up to 4 weeks of forecasting horizon. For each prediction date, we download the corresponding uploaded forecasts, and at the end of the horizon of 4 weeks, we perform the evaluations based on the ground truth on that day.

Tables 5 - 14 show the performance of our model and other model across different prediction dates. The weekly model rankings with respect to the cumulative death WIS, the MAE and WIS of the increase in hospitalized cases, the cumulative confirmed cases MAE and the cumulative death MAE and MAPE are shown in Figures 3, 4, 6, 7, 10 and 11 respectively. Overall, we observe that our model is consistently among the top models.

The comparison models are based on various epidemiological, statistical and machine learning approaches. The descriptions of the models are provided at "covid19-forecast-hub" (https://covid19forecasthub.org/), and summarized below:

- **BPagano-RtDriven**: An SIR model for deaths and cases.
- **CEID-Walk**: A random walk model without drift, using the last few observations of a target time series to estimate the variance in step size of the random walk.
- **COVIDhub-baseline**: Extrapolation with the condition that the median prediction at all future horizons is equal to the most recent observed incidence.
- **COVIDhub-ensemble**: An ensemble, or model average, of submitted forecasts to the COVID-19 Forecast Hub.
- **Covid19Sim-Simulator**: An SEIR model with time-varying inputs, as well as state-specific estimates effective reproductive numbers.
- **CU-select**: A metapopulation SEIR model for projecting future COVID-19 incidence and deaths, using the scenario believed to be most plausible given the current setting.
- **Columbia_UNC-SurvCon**: A survival-convolution model with piece-wise transmission rates that incorporates latent incubation period and provides time-varying effective reproductive number.
- **CovidAnalytics-DELPHI**: An SEIR model augmented with underdetection and interventions, with the projections accounting for reopening and assuming interventions would be re-enacted if cases continue to climb.
- **DDS-NBDS**: Jointly modeling deaths and cases using a negative binomial distribution based nonparametric Bayesian generalized linear dynamical system.
- **Epiforecasts-ensemble1**: An ensemble of three different models based on forecasts for Rt, deaths only and a using deaths and cases.

- **GT-DeepCOVID**: A data-driven deep learning model learns the dependence of hospitalization and mortality rate on various detailed syndromic, demographic, mobility and clinical data (such as increase in positive Covid tests etc) from historical data.
- **IHME-CurveFit**: Uses non-linear mixed effects curve-fitting with the assumption that current interventions continue.
- **JCB-PRM**: The Political Realities Model (PRM) is built on observations of macro-level societal and political responses to COVID measured only in terms of infections and deaths.
- **JHUAPL-Bucky**: A spatial compartment model using public mobility data with estimated local parameters (case reporting rates, doubling times, etc).
- **JHU_UNC_GAS-StatMechPool**: This model jointly trains over locations, fitting to both mechanistic and statistical models, that enforces similar places having similar mechanistic coefficients.
- **Karlen-pypm**: A population modeling framework using discrete-time difference equations with long periods of constant transmission rate.
- **LANL-GrowthRate**: A statistical dynamical growth model accounting for population susceptibility, unconditional on particular intervention strategies.
- **LNQ-ens1**: An ensemble of boosted tree and neural net models with engineered features.
- **MITCovAlliance-SIR**: An SIR model trained on public heath regions with parameters modeled as functions of static demographic and time-varying mobility features.
- **MOBS-GLEAM_COVID**: A metapopulation, age structured compartmental model with the assumption that current interventions continue, modeling human mobility on a network.
- **MSRA-DeepST**: A deep spatio-temporal network with knowledge based SEIR as a regularier under the assumption of spatio-temporal process in pandemic of different regions.
- **NotreDame-mobility**: An ensemble of nine models (each is a deterministic, SEIR-like model) that are identical except that they are driven by different mobility indices.
- **OliverWyman-Navigator**: A compartmental model with non-stationary transition rates that incorporate: (1) the modeled effects of physical distancing and government actions; (2) evolving testing practices in terms of timeliness and scale; (3) the link to tracking and contact tracing, and (4) other region-specific characteristics.
- **PSI-DRAFT**: A stochastic/deterministic, single-population SEIR model that stratifies by both age distribution and disease severity and includes generic intervention fitting.
- **PI_UW-Mob_Collision**: A mobility-informed simplified SIR model motivated by collision theory.

- **RobertWalraven-ESG**: A model based on fitting multiple skewed Gaussian distribution peaks.
- **SteveMcConnell-CovidComplete**: A near-term fatality prediction model that uses estimated fatality trends, trends in positive virus tests and total virus tests, and age-related demographics.
- **UA-EpiCovDA**: An SIR mechanistic model with parameters fitted using a variational data assimilation method.
- **UCLA-SuEIR**: An SEIR model considering both untested and unreported cases, considering reopening and assumes susceptible population will increase after the reopen.
- **UCSB-ACTS**: A data-driven machine learning model makes predictions by referring to other regions with similar growth patterns and assuming the similar development will take place in the current region.
- **UCSD_NEU-DeepGLEAM**: A forecasting model that combines the signal of a discrete stochastic epidemic computational model with a deep learning spatiotemporal forecasting framework.
- **UMass-MechBayes**: A Bayesian compartmental model with observations on cumulative case counts and deaths, fitted independently to each state, including observation noise and a case detection rate.
- **UMich-RidgeTfReg**: A ridge regression (penalized Ordinary Least Squares regression) model to make predictions about future confirmed case and mortality trends.
- **USC-SI_kJalpha**: A heterogeneous infection rate model with human mobility for epidemic modeling, adapting to changing trends and provide predictions of confirmed cases and deaths.
- **UT-Mobility**: A Bayesian multilevel negative binomial regression model that uses the social distancing patterns from anonymized mobile-phone GPS traces.
- **WalmartLabsML-LogForecasting**: A logistic growth prophet forecasting model fit using case counts and deaths as features.

Supplementary Table 5 | 4-week ahead MAE on different prediction dates for cumulative deaths across the US states.

Model	11-15	11-22	11-29	12-06	12-13	12-20	12-27	01-03	01-10
**Our Model**	242.2	189.3	218.8	242.6	251.6	215.1	306.5	359.9	332.6
BPagano-RtDriven	278	303.8	279.6	430.2	377.9	312.7	276.2	508.5	567.3
CEID-Walk	420.4	390.1	501.5	324.3	–	–	486.4	495.2	407.9
COVIDhub-baseline	420.3	386.8	503.9	322.7	379.1	374.4	496.2	513.7	392.2
COVIDhub-ensemble	268.8	195.4	324.6	225.7	239.5	210.1	359	453.4	321.3
CU-select	229.5	284.7	505	310.4	366.2	425	753	832	747.7
Columbia_UNC-SurvCon	697.3	536.5	392.2	695.3	410.3	615.9	422	730	–
Covid19Sim-Simulator	–	430.9	427.8	315.1	377.9	325.5	346.4	385	581
CovidAnalytics-DELPHI	481.9	420.1	399.4	393.5	435.3	292.5	577.7	654.2	852.5
DDS-NBDS	459.2	418.1	614.6	362.3	939.4	447.3	625.5	585.1	702.7
Epiforecasts-ensemble1	–	–	516.1	242.2	269	249.2	434.5	–	371
GT-DeepCOVID	–	–	–	229.5	313.2	228.7	385.7	–	323.2
IHME-CurveFit	–	312.8	–	338	–	–	–	–	–
JCB-PRM	240.9	261.9	280.2	313.1	337.3	339.7	277.7	–	–
JHUAPL-Bucky	351.4	423.2	325.4	264.6	302.4	543.3	780.8	687.1	743.3
JHU_CSSE-DECOM	242.2	257.4	291.3	352.8	404	410	394.7	–	341.3
Karlen-pypm	257.1	242.3	242.4	256.5	253.5	188.7	269.9	347.2	293.5
LANL-GrowthRate	308.2	322.4	471.1	304.4	286.6	446.9	–	533.2	457.8
LNQ-ens1	198.7	158	432.1	231.4	234.2	211	582.5	466.9	334.2
MIT_CritData-GBCF	424.4	301	302.2	310.1	333.7	378.7	400.6	522.1	400.9
MOBS-GLEAM_COVID	308	281.5	352.8	361.4	457.3	267.1	322.2	394.1	339.2
MSRA-DeepST	340.4	210.6	312.2	284.4	–	–	–	–	–
NotreDame-mobility	517.8	–	555.2	622.4	848.9	–	–	–	–
OliverWyman-Navigator	256	228.6	290.8	310.9	369.9	214.2	356.2	314	461.6
PSI-DRAFT	524.1	576.7	613.5	590.7	938.7	621.4	584.9	583.4	760.7
RobertWalraven-ESG	419.5	438.7	727.5	628.7	518.5	372.5	742.3	698.1	389
SteveMcConnell-CovidComplete	248.5	271.5	304.7	289.8	229.3	225.3	520.6	412.4	389.1
UA-EpiCovDA	292	298.8	492.2	298.6	272.7	482.2	900.8	562.1	307.3
UCLA-SuEIR	561.2	359.8	558.1	326	412.7	405.6	696.2	575	423.1
UCSB-ACTS	436	426.2	339.3	425.6	419	426.5	341.6	489.2	603.2
UCSD_NEU-DeepGLEAM	294.7	273.5	349.1	387.5	485.1	286.5	380.4	396.1	358.9
UMass-MechBayes	237.1	207	363	203.1	211.2	193.9	538.9	427	312.8
UMich-RidgeTfReg	443.5	471.6	–	–	–	544.3	625.4	542.9	724.2
USACE-ERDC_SEIR	1079.8	1278.2	1350.1	–	–	–	–	–	–
USC-SI_kJalpha	233	215.5	258.3	246.2	207.3	208.2	328.2	546.9	399.9
UT-Mobility	416.3	440.6	486.4	473.5	371.7	392.8	–	–	452.9
WalmartLabsML-LogForecasting	758	898.3	1001.2	1022.3	1087.2	1177.7	–	1249.9	1201.1

Supplementary Table 6 | 4-week ahead MAPE on different prediction dates for cumulative deaths across the US states.

Model	11-15	11-22	11-29	12-06	12-13	12-20	12-27	01-03	01-10
**Our Model**	5.4	6	5.6	5.1	5.5	4.5	4.5	4.9	5.9
BPagano-RtDriven	8.1	9.2	7.4	11.4	10.6	7.2	5	5.9	7.1
CEID-Walk	10.4	8.7	9.4	5.3	–	–	6.2	6.8	5.6
COVIDhub-baseline	10.5	8.8	9.3	5.6	7	5.3	5.9	6.8	5.8
COVIDhub-ensemble	7	5.3	6	3.9	3.9	3.8	4.3	5.2	4.5
CU-select	7	7.1	11	6.8	7	7	10.3	11.3	9.6
Columbia_UNC-SurvCon	19	14.4	9.4	12.7	7.1	8.7	7	8.9	–
Covid19Sim-Simulator	–	10.8	11.7	6.7	6.4	5.5	6.4	5.8	6.9
CovidAnalytics-DELPHI	11.3	7	6.6	6.4	6.2	4.7	7.2	7.5	9.1
DDS-NBDS	11.8	9.1	11.5	6.1	17.5	6.1	9.3	7.2	7.9
Epiforecasts-ensemble1	–	–	8.9	6.7	5.2	4.9	5.9	–	5.9
GT-DeepCOVID	–	–	–	4.8	6.4	4.3	5.4	–	4.8
IHME-CurveFit	–	8.5	–	9.8	–	–	–	–	–
JCB-PRM	7.2	7.4	6.7	5.8	5.8	6.6	5.2	–	–
JHUAPL-Bucky	8.3	10.6	8.4	5.1	6.2	8.1	10.8	7.7	8.3
JHU_CSSE-DECOM	6.1	8.6	6.7	6.9	9.6	8.2	4.1	–	5.5
Karlen-pypm	8.6	7	5.8	5.2	6	5.2	5.2	5.7	5.7
LANL-GrowthRate	9.9	8.3	9.7	7	5.8	5.8	–	6.9	6.3
LNQ-ens1	7.3	5.9	9.4	5.1	5.3	4.4	8.6	6.2	4.8
MIT_CritData-GBCF	9	7.1	7.7	7.2	8.3	9.5	5.4	7.5	6.7
MOBS-GLEAM_COVID	8.2	7.5	6.7	7.3	7.9	4.4	4.4	5.3	5.9
MSRA-DeepST	7.9	5.5	6.4	5.4	–	–	–	–	–
NotreDame-mobility	15.3	–	12.8	13.9	12.4	–	–	–	–
OliverWyman-Navigator	6.9	6.4	6.8	4.8	6.8	3.8	5.3	4.8	6.6
PSI-DRAFT	12.7	12	10.6	9.3	15.3	8.7	7.7	6.9	7.4
RobertWalraven-ESG	9.9	12.3	18.2	13	11.2	6.5	9.5	8.6	5.8
SteveMcConnell-CovidComplete	7.8	7.8	7	5.4	3.9	3.6	6.9	5.4	5
UA-EpiCovDA	7.7	6.6	9.1	6.9	5.1	6.6	11	6.8	5.7
UCLA-SuEIR	14.8	8.5	11.3	6.6	6.9	5.9	8.8	7.3	6
UCSB-ACTS	10.1	11.8	9	8.3	7.9	8.6	5.1	6.7	9.3
UCSD_NEU-DeepGLEAM	7.5	7.1	6.2	8.9	10.4	6.6	8.2	6.5	7.5
UMass-MechBayes	7.1	6.2	7.5	4.7	4.3	3.1	6.9	5.2	3.9
UMich-RidgeTfReg	11.7	11.8	–	–	–	7.6	7.6	7.4	7.9
USACE-ERDC_SEIR	18.5	19.8	19.5	–	–	–	–	–	–
USC-SI_kJalpha	7.6	6.6	6.7	4.9	4.8	3.6	5.5	7.2	5.6
UT-Mobility	12.4	12.3	11.8	11.3	8.5	7.7	–	–	5.8
WalmartLabsML-LogForecasting	19.1	20	19.7	17.7	16	15.4	–	13.6	12.2

Supplementary Table 7 | 4-week ahead MAPE on different prediction dates for incident deaths across the US states.

Model	11-15	11-22	11-29	12-06	12-13	12-20	12-27	01-03	01-10
**Our Model**	24.4	22.4	21.4	21.6	25.3	21.3	20.8	23	35.3
BPagano-RtDriven	33.9	35.2	31.3	51.7	47.1	35	26.3	30	42.9
CEID-Walk	42.6	34	38.1	26.7	–	–	27.2	31.6	33.2
COVIDhub-baseline	42.5	34.4	38.1	27.5	33	26.2	25.9	31.8	33.7
COVIDhub-ensemble	30.1	19.9	23.9	17.8	19.1	18.6	19.1	23.2	26.2
CU-select	28.8	26.6	41.8	26.6	32.4	32.7	48	54.7	47.8
Columbia_UNC-SurvCon	76.5	60.4	38.8	51.8	34.5	41.2	31.4	42.1	–
Covid19Sim-Simulator	–	42.2	42.7	28.7	28.5	25.2	29.3	28.4	34.3
CovidAnalytics-DELPHI	45.2	32.8	30	29.7	28.8	22.3	34.6	36.8	46
DDS-NBDS	47	35	43.6	24.7	78.5	29.2	47.1	32.6	48
Epiforecasts-ensemble1	–	–	36.6	36.1	25.1	24.9	28.4	–	36.2
GT-DeepCOVID	–	–	–	19.4	29.3	19.5	23	–	27.5
IHME-CurveFit	–	35.3	–	40	–	–	–	–	–
JCB-PRM	30.6	28.1	26.8	24.4	25.9	30.9	23	–	–
JHUAPL-Bucky	39.2	49.3	33	22	26.5	37.1	54	35.8	43.5
JHU_CSSE-DECOM	26.3	31.8	25.8	29.2	44.4	41	20.2	–	33.9
Karlen-pypm	35.6	25.4	22.9	22.1	26.4	23	22.8	26.1	31.2
LANL-GrowthRate	38.8	30.6	37.2	26.7	25.2	25.9	–	32.3	37.8
LNQ-ens1	29.2	21.6	35.7	21.8	24	20	39.2	28.7	28.8
MIT_CritData-GBCF	36.6	26.6	28.5	31.3	39	47.7	25.3	38.6	40.3
MOBS-GLEAM_COVID	33.8	29	25.2	31.3	35.9	21.1	19.5	26.1	33.5
MSRA-DeepST	34.4	20.6	23.8	23.2	–	–	–	–	–
NotreDame-mobility	63.3	–	54.5	76.3	63	–	–	–	–
OliverWyman-Navigator	29.6	23.6	25.7	20.9	32.1	17.9	24.6	22.2	39.5
PSI-DRAFT	56.2	48.3	44.5	47.8	67.6	43.7	37.3	33.9	43.4
RobertWalraven-ESG	45.8	48.3	71.4	60.7	53.2	31.1	42.7	40.3	32.6
SteveMcConnell-CovidComplete	30.6	28.8	29.6	24.8	19	17.4	31.8	24.3	26.9
UA-EpiCovDA	31.4	25.1	34.6	28.1	24.1	29.3	49.8	31	30.8
UCLA-SuEIR	57.5	33	43.7	28.6	30.9	26.7	38.8	34.8	32.5
UCSB-ACTS	41.5	43.4	33.5	34.5	37.2	42.9	24.8	32.9	57.4
UCSD_NEU-DeepGLEAM	31.1	27.6	24	37.4	46.9	31.7	36.1	32.7	44.4
UMass-MechBayes	29.7	23.8	28.1	21	20.2	14.6	31.6	23.7	22.4
UMich-RidgeTfReg	43.8	43	–	–	–	38.6	35.9	37.7	47.2
USACE-ERDC_SEIR	103.1	101.9	97.9	–	–	–	–	–	–
USC-SI_kJalpha	29.9	25	24.7	22.6	23.1	16.8	25.5	32.7	32.3
UT-Mobility	48.8	46.1	46.3	45.7	36	35.5	–	–	30.4
WalmartLabsML-LogForecasting	76	77.5	77.9	73.9	70.2	70.7	–	63.8	61

Supplementary Table 8 | 4-week ahead WIS on different prediction dates for cumulative deaths across the US states.

**Model**	**11-15**	**11-22**	**11-29**	**12-06**	**12-13**	**12-20**	**12-27**	**01-03**	**01-10**
**Our model**	188.3	119.4	137.4	156.7	151.4	149.2	221.8	287.5	246.1
BPagano-RtDriven	206.8	210.9	198.7	304.8	266.8	218.5	200.1	332.1	373.7
CEID-Walk	339.5	293.3	377.8	234.4	–	–	357.2	366.7	263.1
COVIDhub-baseline	304.9	270.0	363.8	223.4	280.5	263.0	381.5	398.5	269.7
COVIDhub-ensemble	180.9	130.8	214.8	163.9	156.8	132.5	236.0	329.6	210.9
CU-select	150.1	195.7	388.9	208.8	248.2	295.4	655.5	714.7	642.8
Columbia_UNC-SurvCon	580.5	438.3	331.4	–	–	–	345.2	667.2	–
Covid19Sim-Simulator	–	393.4	384.1	288.3	349.0	298.8	317.5	360.1	555.3
CovidAnalytics-DELPHI	433.2	366.3	334.7	327.3	359.0	229.7	473.7	551.1	742.2
DDS-NBDS	360.1	311.1	500.2	275.6	483.3	288.7	384.3	445.0	349.3
Epiforecasts-ensemble1	–	–	358.9	189.3	5258.5	172.9	280.6	–	262.7
GT-DeepCOVID	–	–	–	178.0	261.0	173.4	332.9	–	272.7
IHME-CurveFit	–	212.4	–	216.0	–	–	–	–	–
JCB-PRM	177.6	186.6	197.2	208.2	203.5	218.8	183.3	–	–
JHUAPL-Bucky	249.5	298.5	236.6	190.4	214.8	352.7	521.7	614.7	637.1
JHU_CSSE-DECOM	190.2	191.8	219.6	262.2	285.7	302.9	306.2	–	241.9
Karlen-pypm	165.7	170.0	162.3	160.8	176.9	136.6	181.3	221.9	199.7
LANL-GrowthRate	201.5	208.7	327.6	194.6	191.4	281.5	–	377.0	319.2
LNQ-ens1	138.4	139.8	242.0	174.1	183.0	171.0	297.3	270.5	252.2
MIT_CritData-GBCF	–	–	–	368.6	352.6	367.9	579.2	712.1	513.5
MOBS-GLEAM_COVID	219.5	175.2	253.8	254.8	296.7	168.7	243.2	307.0	254.3
MSRA-DeepST	323.3	201.4	294.7	219.5	–	–	–	–	–
NotreDame-mobility	497.3	–	534.3	594.8	823.7	–	–	–	–
OliverWyman-Navigator	193.8	161.2	214.8	238.6	269.3	147.1	222.9	207.9	297.9
PSI-DRAFT	474.9	521.0	570.0	531.0	812.5	534.2	484.4	510.4	663.7
RobertWalraven-ESG	356.7	380.6	516.6	585.1	410.1	361.7	534.6	505.4	480.9
SteveMcConnell-CovidComplete	159.7	197.5	199.6	230.0	227.3	229.8	268.1	253.0	297.2
UA-EpiCovDA	236.8	207.8	298.1	240.8	206.2	417.3	820.2	535.8	278.4
UCLA-SuEIR	539.4	318.1	515.8	287.7	373.8	364.9	649.2	523.0	370.6
UCSB-ACTS	301.5	300.9	238.4	298.4	278.3	285.4	235.1	329.2	376.1
UCSD_NEU-DeepGLEAM	209.5	194.3	264.8	288.0	378.7	206.7	262.7	292.5	252.3
UMass-MechBayes	169.1	159.4	255.9	158.9	161.0	143.0	359.7	279.8	216.3
UMich-RidgeTfReg	436.0	463.2	–	–	–	534.2	615.3	532.4	711.8
USACE-ERDC_SEIR	1045.2	1238.9	1312.7	–	–	–	–	–	–
USC-SI_kJalpha	151.2	138.4	179.1	159.1	143.9	151.8	213.2	391.6	267.3
UT-Mobility	310.5	294.5	345.5	317.4	249.8	270.5	–	–	290.2
WalmartLabsML-LogForecasting	667.5	797.9	874.1	876.3	916.5	989.1	–	1064.9	1018.3

Supplementary Table 9 | 4-week ahead MAE on different prediction dates for cumulative cases across US states.

Model	11-15	11-22	11-29	12-06	12-13	12-20	12-27	01-03	01-10
**Our Model**	32053.4	42754.7	40504	21474.3	38310.2	45864.2	28127.9	22410.9	57046
BPagano-RtDriven	46502.7	47753.3	35789.3	37879.8	33760	36767.5	37741.4	25705.1	40548.5
CEID-Walk	30310.4	32858.6	35872.2	29242.3	–	–	22179.2	16194	45916.9
COVIDhub-baseline	30186.1	32688.6	35194	29530.5	27115.7	19689.5	21590.9	15804.5	45831.3
COVIDhub-ensemble	29291.9	29328.1	29999.2	23426.3	18883.6	19599.5	22122.7	13059.5	45476.5
CU-select	22185.1	24381.8	33547.4	28237	20688.7	18125.6	30703.1	85884.9	134493
Covid19Sim-Simulator	–	32174.8	35998.7	32512.8	23420.4	22177.8	23391.2	17421.4	44462
CovidAnalytics-DELPHI	82754.5	71214.7	64714.4	59964.2	44217.4	40829.7	36745.5	33436	86958.2
DDS-NBDS	113852	84996.1	36806.9	62098	36791.1	41596.5	599623	62666.2	155824
JCB-PRM	28825.6	44413.1	49676	43186.5	39003	50171.3	39588.7	–	–
JHUAPL-Bucky	76184.9	70077.5	31703.4	30754.8	49608.7	51071.9	52437.5	21992.8	50275.4
JHU_CSSE-DECOM	33493.1	47062.1	34593.4	35246.7	24389.1	19124.5	20825.6	–	–
JHU_UNC_GAS-StatMechPool	39695.8	–	–	34528.7	33894	25865	38167.2	19100.3	25647.2
Karlen-pypm	60896.6	65833.9	40988.6	37408.5	55336.1	53411.9	73077	25990.3	39715.5
LANL-GrowthRate	42174.5	28576.7	27922.2	17279.5	26617.2	34812.7	–	16757.3	48362.5
LNQ-ens1	29175.9	23637.3	31136.6	20736	19698.9	21867.1	33130.9	14730.3	59385.2
MOBS-GLEAM_COVID	–	–	–	–	–	–	–	–	18681.7
MSRA-DeepST	30740.6	38163.4	31998.4	32767.3	–	–	–	–	–
RobertWalraven-ESG	36836.8	32084.2	43150.7	36997.7	21797.6	23309.9	38841.3	18920.6	42033.6
TTU-squider	57551.1	62060.6	83003	50172.8	37273.9	30865.3	32628.1	35749.1	35148.6
UCLA-SuEIR	47681.5	35547.5	40870.7	36870.3	31336.7	24932.6	28068.2	23891.6	42193.1
UCSB-ACTS	33140.8	63182.9	88967.8	69733.6	57322.9	38127.6	71747.6	59871	49562.5
UMich-RidgeTfReg	89348	52521.7	–	–	–	59061.2	32540.8	35190.5	64749.4
USACE-ERDC_SEIR	22132.4	36251.4	53714.1	–	–	–	–	–	–
USC-SI_kJalpha	41908.5	32088.8	34601.3	22082.6	29471.4	24764.7	27827.5	19520.7	37291.5

Supplementary Table 10 | 4-week ahead MAPE on different prediction dates for cumulative cases across US states.

Model	11-15	11-22	11-29	12-06	12-13	12-20	12-27	01-03	01-10
**Our Model**	8.8	12.4	11.3	6.6	7.6	8.8	6.2	5.6	13.4
BPagano-RtDriven	15.5	21.4	11.2	13.8	10.4	7	6.3	4.8	8
CEID-Walk	9.7	9.1	8.9	7.2	–	–	4	3.1	8.3
COVIDhub-baseline	9.7	8.9	8.7	7.2	6.2	4	3.9	3.1	8.4
COVIDhub-ensemble	9.1	9.3	7.7	6.7	5.4	3	3.7	2.4	7.9
CU-select	8.1	7.7	7.9	7.5	5.1	3.6	7.6	17.2	21.7
Covid19Sim-Simulator	–	9.4	9.6	8.2	6.6	4.1	3.3	2.5	7.3
CovidAnalytics-DELPHI	28.1	30.3	21.8	17.7	12.4	6.4	7.6	7	13.7
DDS-NBDS	97.9	30.1	12.2	24.3	11.9	14.8	210.9	13	29
JCB-PRM	10.7	14.2	15.4	13.1	11.5	10.1	6.2	–	–
JHUAPL-Bucky	35.9	31.5	10.9	8.9	10.5	7.7	8.5	4.8	11.1
JHU_CSSE-DECOM	13.1	17.2	9.7	8.7	7.1	4.4	4.3	–	–
JHU_UNC_GAS-StatMechPool	12.9	–	–	8.8	7.8	4.7	8.1	4.4	5.4
Karlen-pypm	27.7	28.8	13	13.6	14.6	9.4	9.1	5.9	6.9
LANL-GrowthRate	13.9	10.7	7.1	5.1	5.4	4.3	–	3.6	8.6
LNQ-ens1	10.1	7.7	7.2	6.5	4.8	3.5	6.9	3.2	10.6
MOBS-GLEAM_COVID	–	–	–	–	–	–	–	–	5.1
MSRA-DeepST	10.4	13.6	8.1	8.2	–	–	–	–	–
RobertWalraven-ESG	13.9	10.6	13.3	9.1	6.5	4.4	8.1	4.1	7.3
TTU-squider	19.4	18.8	21.6	11.8	12	9.4	8.2	6.7	6.4
UCLA-SuEIR	15	9.4	9.7	9.7	7.5	5.5	5.5	4.6	7.1
UCSB-ACTS	11.6	20.1	28.4	22.7	17.6	10.2	12.7	9.6	8.1
UMich-RidgeTfReg	33.9	20.7	–	–	–	8.3	5.6	5.4	10.2
USACE-ERDC_SEIR	11	10.7	13.9	–	–	–	–	–	–
USC-SI_kJalpha	15	11.3	8.7	6.6	7.2	3.9	4.1	3.7	6.3

Supplementary Table 11 | 4-week ahead MAPE on different prediction dates for incident cases across US states.

Model	11-15	11-22	11-29	12-06	12-13	12-20	12-27	01-03	01-10
**Our Model**	27.9	43.5	43.1	26.6	32.9	38	28.6	31.8	93.9
BPagano-RtDriven	47.7	70.4	40.9	57	49.3	32.8	30.4	30	63.4
CEID-Walk	29.8	30.7	31.7	31.8	–	–	17.6	17.9	61.6
COVIDhub-baseline	29.8	29.8	31.2	32	32	20.8	16.6	17.8	62.3
COVIDhub-ensemble	28.2	31.1	27	29.2	24.7	13.1	16.5	12.3	56.8
CU-select	24.4	24.4	25.8	29.8	21.6	15.6	36.6	94.5	148.7
Covid19Sim-Simulator	–	30.7	33.2	35.9	33.1	20.7	14.4	12.5	52.5
CovidAnalytics-DELPHI	81	90.3	74.6	69.8	50.5	29.2	37.1	41.5	106.7
DDS-NBDS	248.7	97.4	43.5	96.9	48.8	61.8	857.3	66.9	184.5
JCB-PRM	33.4	50.1	61	57	52.4	47.2	28.2	–	–
JHUAPL-Bucky	95.6	100.1	38.5	35	41.6	32.7	41.3	25.3	66.5
JHU_CSSE-DECOM	40.3	58.1	38.1	38.4	36.1	21.3	20.8	–	–
JHU_UNC_GAS-StatMechPool	40.5	–	–	36.6	32.3	20.7	41.9	25.4	37.6
Karlen-pypm	78.5	88.1	42.9	49.4	54.5	34.6	36.8	27.8	40.2
LANL-GrowthRate	40.3	33.2	24.8	20.9	21.4	18.9	–	19.7	58
LNQ-ens1	30.6	26.1	24.3	27.6	22.9	15	30.9	16.8	77.1
MOBS-GLEAM_COVID	–	–	–	–	–	–	–	–	33.7
MSRA-DeepST	31.9	45.9	30.4	35.9	–	–	–	–	–
RobertWalraven-ESG	41.1	34.1	47.8	38.1	29.1	20	38.3	22.5	49.3
TTU-squider	56.2	58.9	76.7	45.4	49.7	39.6	37.7	35.3	39.4
UCLA-SuEIR	45.3	31.5	34.7	41.4	35.3	25.9	28	27.3	55.2
UCSB-ACTS	36.5	72.1	116.3	108.5	92.9	57.2	62.4	51.9	53.6
UMich-RidgeTfReg	91.5	61.8	–	–	–	37.3	25.3	31.7	73.1
USACE-ERDC_SEIR	31.3	33.9	47.9	–	–	–	–	–	–
USC-SI_kJalpha	44.7	37.3	29.7	26.3	33.2	16.2	18.1	17.9	42.3

Supplementary Table 12 | 4-week ahead MAE on different prediction dates for hospitalized increased for US states.

Model	11-15	11-22	11-29	12-06	12-13	12-20	12-27	01-03	01-10
**Our Model**	74.8	85.8	82.3	107.5	136.4	110.6	140.1	85.6	55.2
COVIDhub-ensemble	–	–	–	279.3	368.5	269.1	281.2	244.4	323.4
CU-select	105.9	101.9	94.8	145.7	309.3	227.8	209.9	566.0	671.5
Covid19Sim-Simulator	–	247.2	265.0	419.3	429.3	323.9	376.6	354.0	369.3
IHME-CurveFit	–	106.0	–	140.5	–	–	–	–	–
JHUAPL-Bucky	264.5	842.5	401.3	373.3	518.6	237.9	239.5	236.0	277.1
Karlen-pypm	313.2	481.7	352.0	425.4	492.4	379.9	324.3	264.0	302.3
LANL-GrowthRate	303.0	257.7	231.2	300.1	365.1	295.8	–	298.0	373.8
MOBS-GLEAM_COVID	–	–	–	–	–	–	257.8	190.8	235.8
UCSB-ACTS	–	–	–	274.2	332.8	301.4	342.3	237.5	283.5
USACE-ERDC_SEIR	97.8	84.4	62.5	–	–	–	–	–	–
USC-SI_kJalpha	–	322.6	218.0	292.3	413.0	346.3	362.9	231.9	356.1

Supplementary Table 13 | 4-week ahead MAPE on different prediction dates for hospitalized increased for US states.

Model	11-15	11-22	11-29	12-06	12-13	12-20	12-27	01-03	01-10
**Our Model**	131.0	185.0	189.6	104.5	117.2	124.1	65.4	70.7	71.3
COVIDhub-ensemble	–	–	–	224.0	254.3	186.1	135.4	173.8	286.3
CU-select	80.9	97.3	219.9	109.2	237.8	117.7	69.2	488.5	806.8
Covid19Sim-Simulator	–	284.8	645.3	390.9	385.9	253.3	243.3	255.5	346.3
IHME-CurveFit	–	79.1	–	147.1	–	–	–	–	–
JHUAPL-Bucky	325.7	944.3	738.9	248.2	215.6	169.5	100.1	200.4	282.6
Karlen-pypm	298.4	835.6	532.8	289.0	215.1	324.8	109.3	139.4	226.7
LANL-GrowthRate	334.1	374.0	477.2	264.3	267.9	189.8	–	234.8	440.8
MOBS-GLEAM_COVID	–	–	–	–	–	–	141.1	153.8	267.1
UCSB-ACTS	–	–	–	294.7	382.0	247.5	188.5	148.7	272.1
USACE-ERDC_SEIR	128.0	71.0	106.8	–	–	–	–	–	–
USC-SI_kJalpha	–	459.4	458.5	250.3	330.6	245.6	172.8	144.4	309.6

Supplementary Table 14 | 4-week ahead WIS on different prediction dates for hospitalized increased for US states.

Model	11-15	11-22	11-29	12-06	12-13	12-20	12-27	01-03	01-10
**Our Model**	55.6	54.3	51.6	66.6	107.4	73.4	115.9	56.3	35.3
COVIDhub-ensemble	–	–	–	223.2	307.7	213.9	238.4	196.2	262.5
CU-select	88.7	81.8	79.5	121.9	268.9	194.3	180.5	441.5	556.7
Covid19Sim-Simulator	–	234.8	248.6	408.3	419.2	314.0	366.7	346.3	359.1
IHME-CurveFit	–	82.5	–	110.4	–	–	–	–	–
JHUAPL-Bucky	214.7	667.4	340.0	313.4	445.2	197.1	205.6	196.2	235.6
Karlen-pypm	210.7	333.5	241.6	294.0	351.1	263.4	237.2	178.8	209.1
LANL-GrowthRate	231.7	195.8	175.5	229.3	284.8	219.4	–	216.2	278.5
MOBS-GLEAM_COVID	–	–	–	–	–	–	221.2	150.4	182.6
UCSB-ACTS	–	–	–	223.3	276.6	243.0	282.4	179.6	226.7
USACE-ERDC_SEIR	95.8	82.8	61.3	–	–	–	–	–	–
USC-SI_kJalpha	–	285.2	195.7	266.6	384.4	319.5	321.6	206.1	317.1

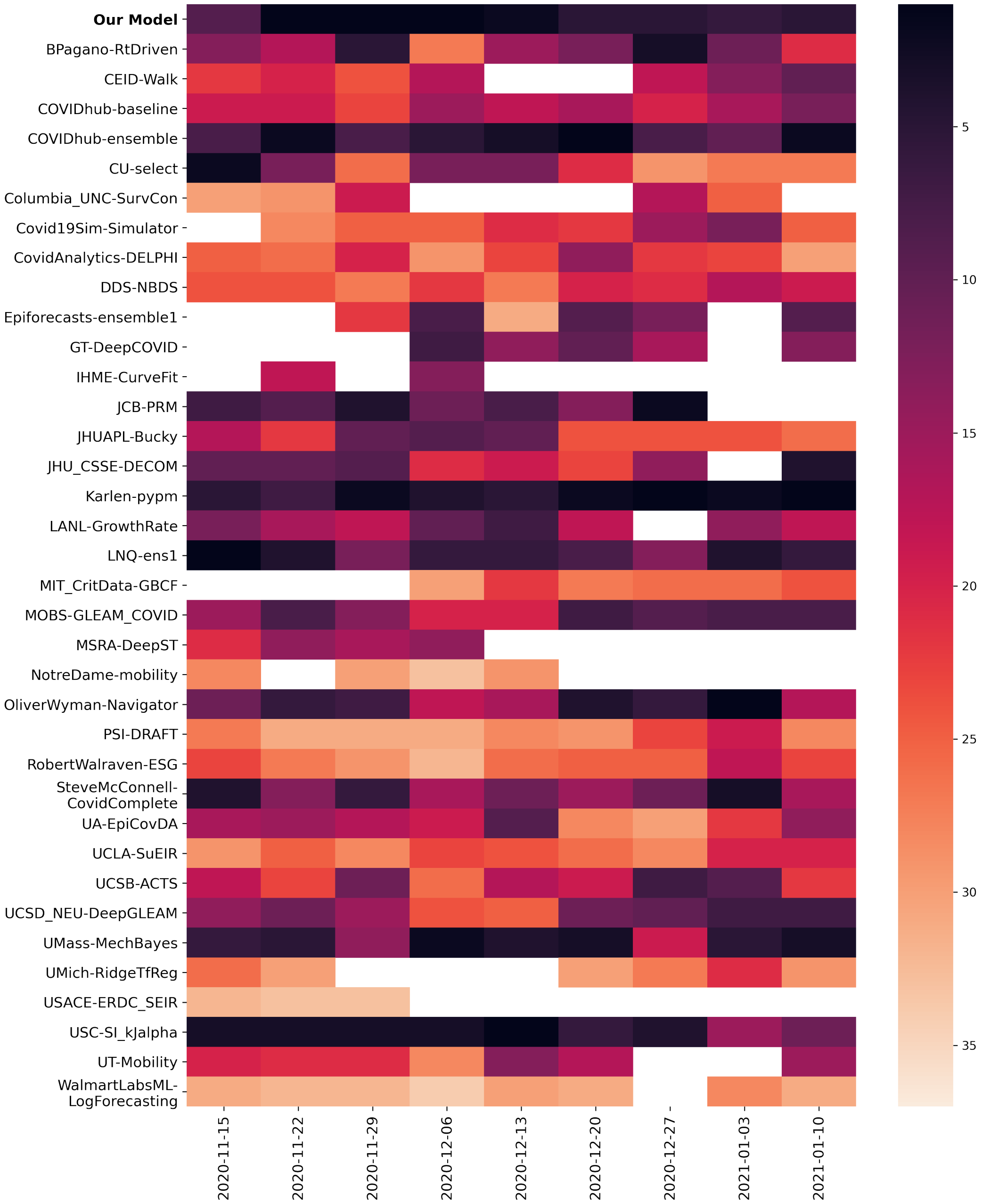

Supplementary Figure 3 | Normalized ranking for death WIS. The darker the color, the higher the ranking of the model is for the corresponding prediction date.

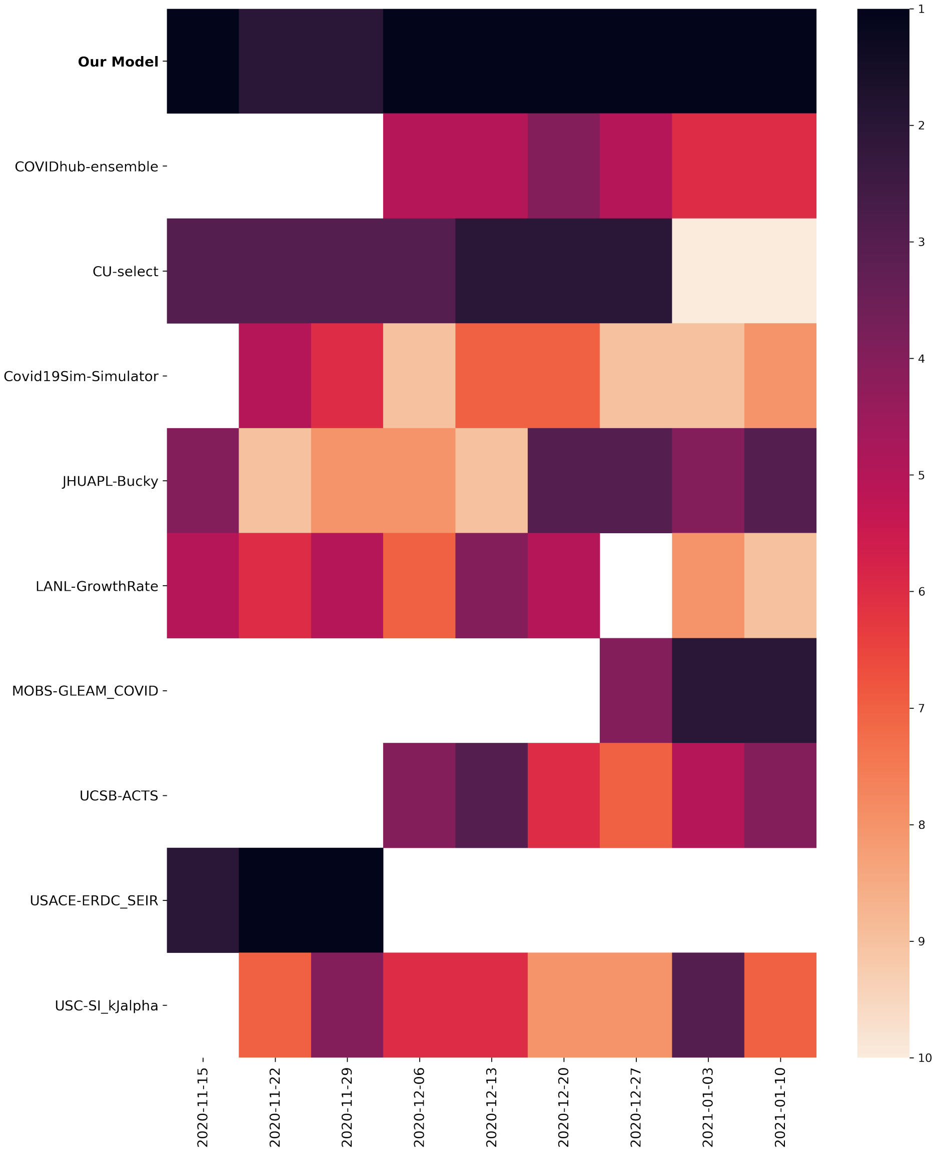

Supplementary Figure 4 | Normalized ranking for hospitalized increase MAE. The darker the color, the higher the ranking of the model is for the corresponding prediction date.

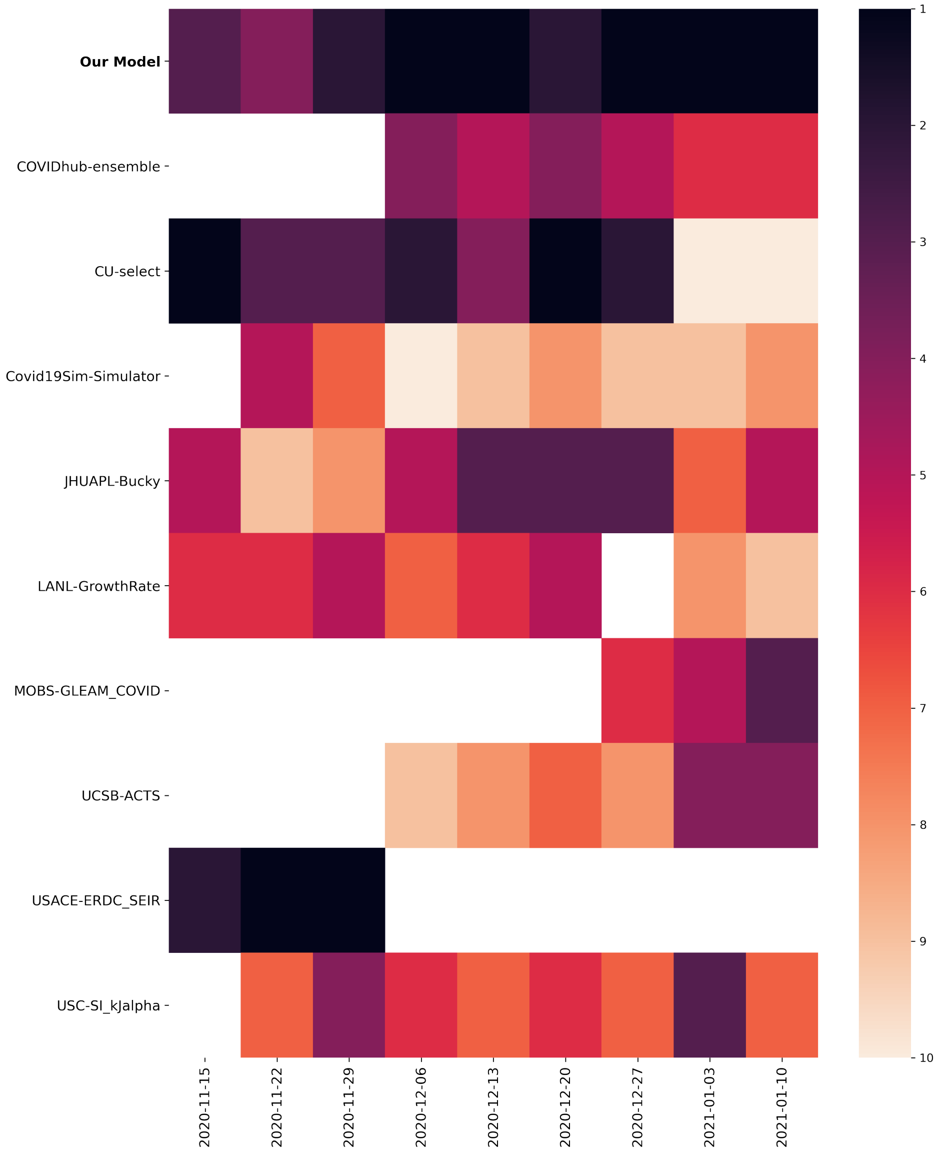

**Supplementary Figure 5 | ** Normalized ranking for hospitalized increase MAPE. The darker the color, the higher the ranking of the model is for the corresponding prediction date.

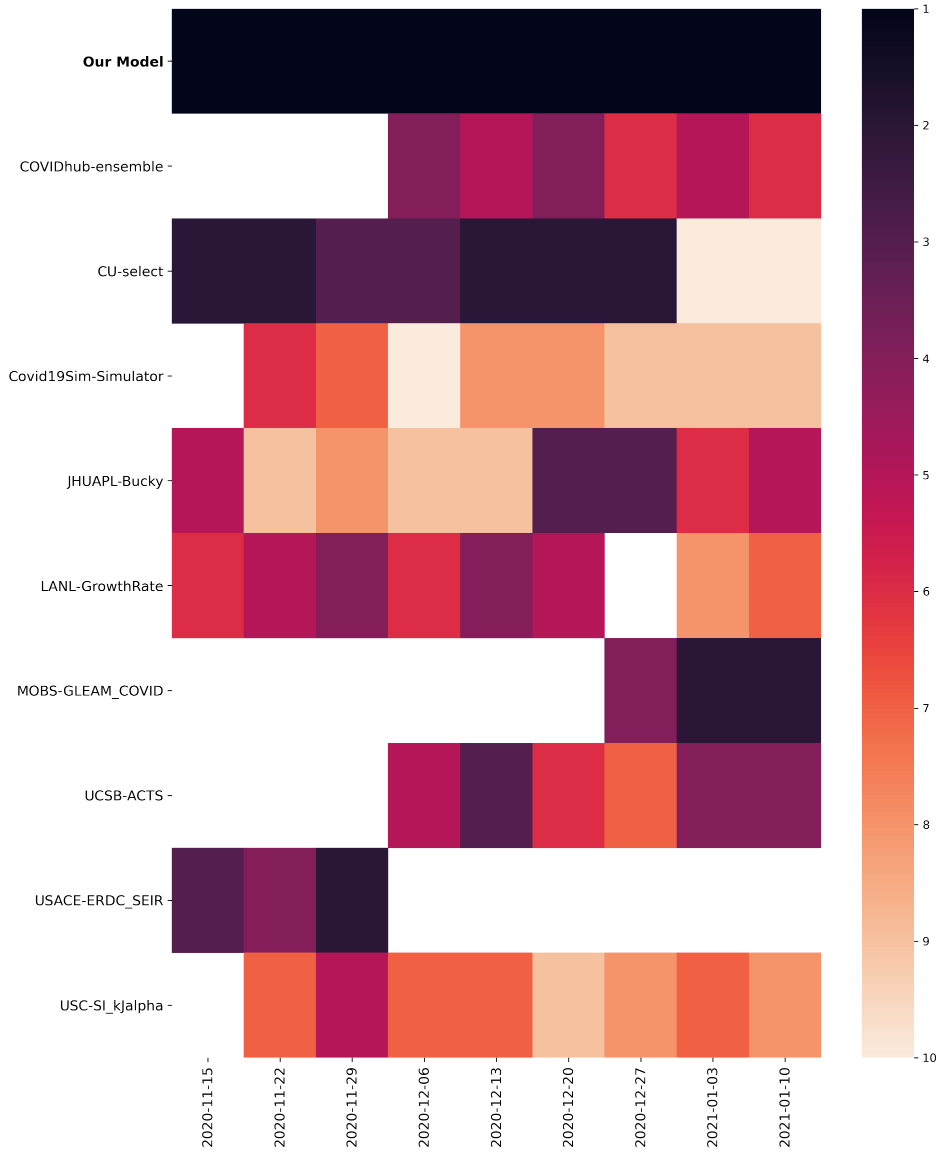

Supplementary Figure 6 | Normalized ranking for hospitalized increase WIS. The darker the color, the higher the ranking of the model is for the corresponding prediction date.

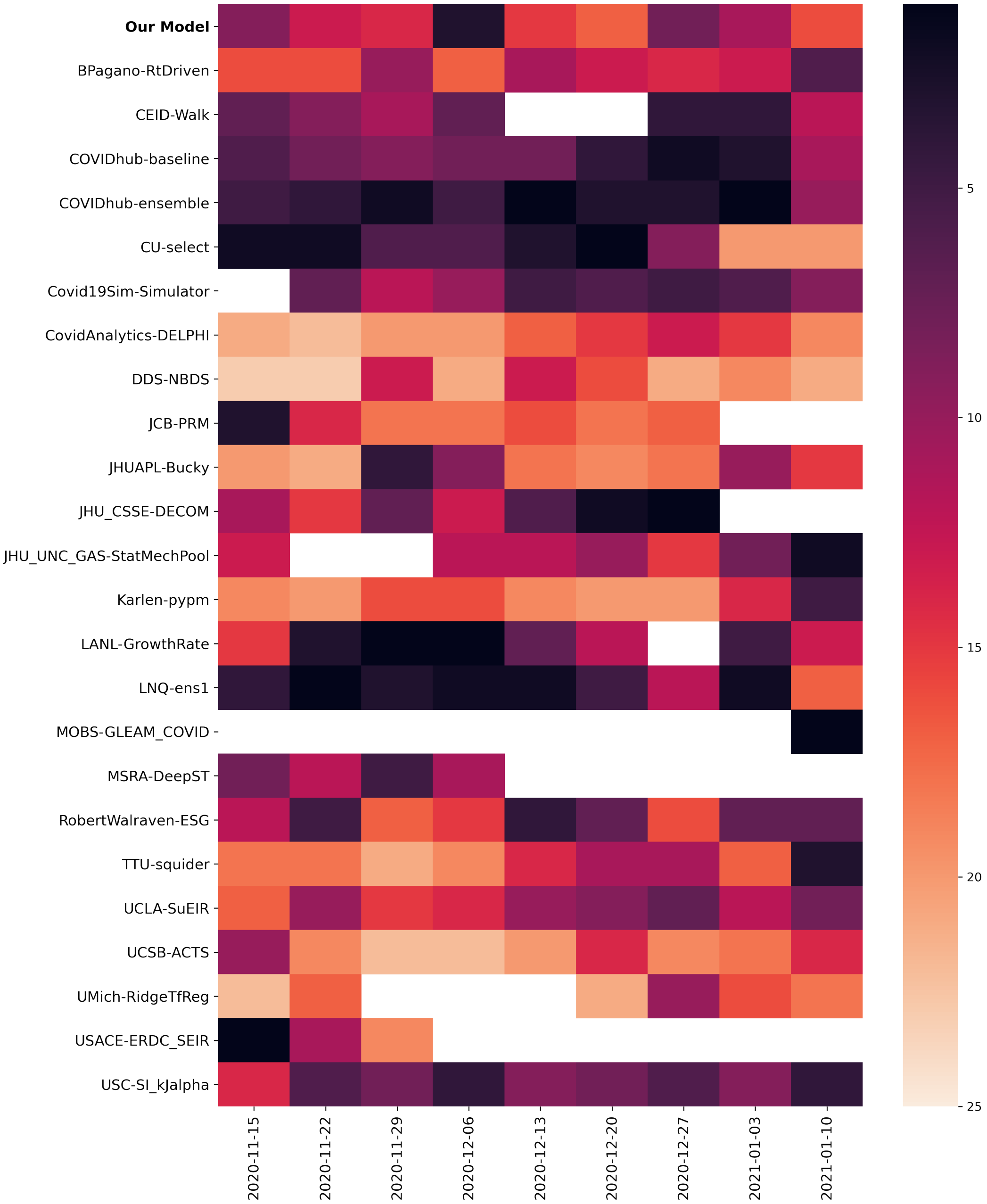

Supplementary Figure 7 | Model ranking for cumulative cases MAE. The darker the color, the higher the ranking of the model is for the corresponding prediction date.

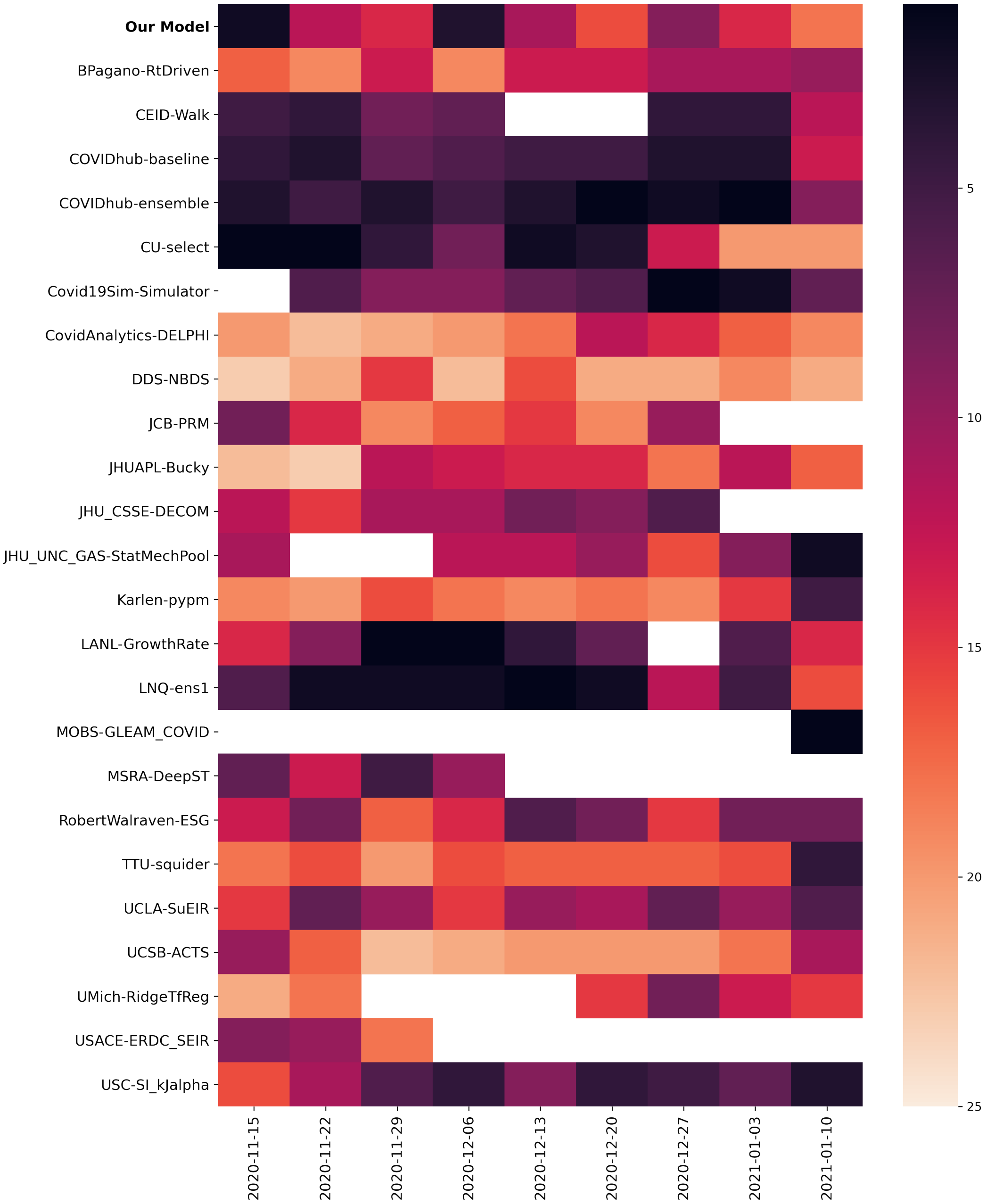

Supplementary Figure 8 | Model rankings for cumulative cases MAPE. The darker the color, the higher the ranking of the model is for the corresponding prediction date.

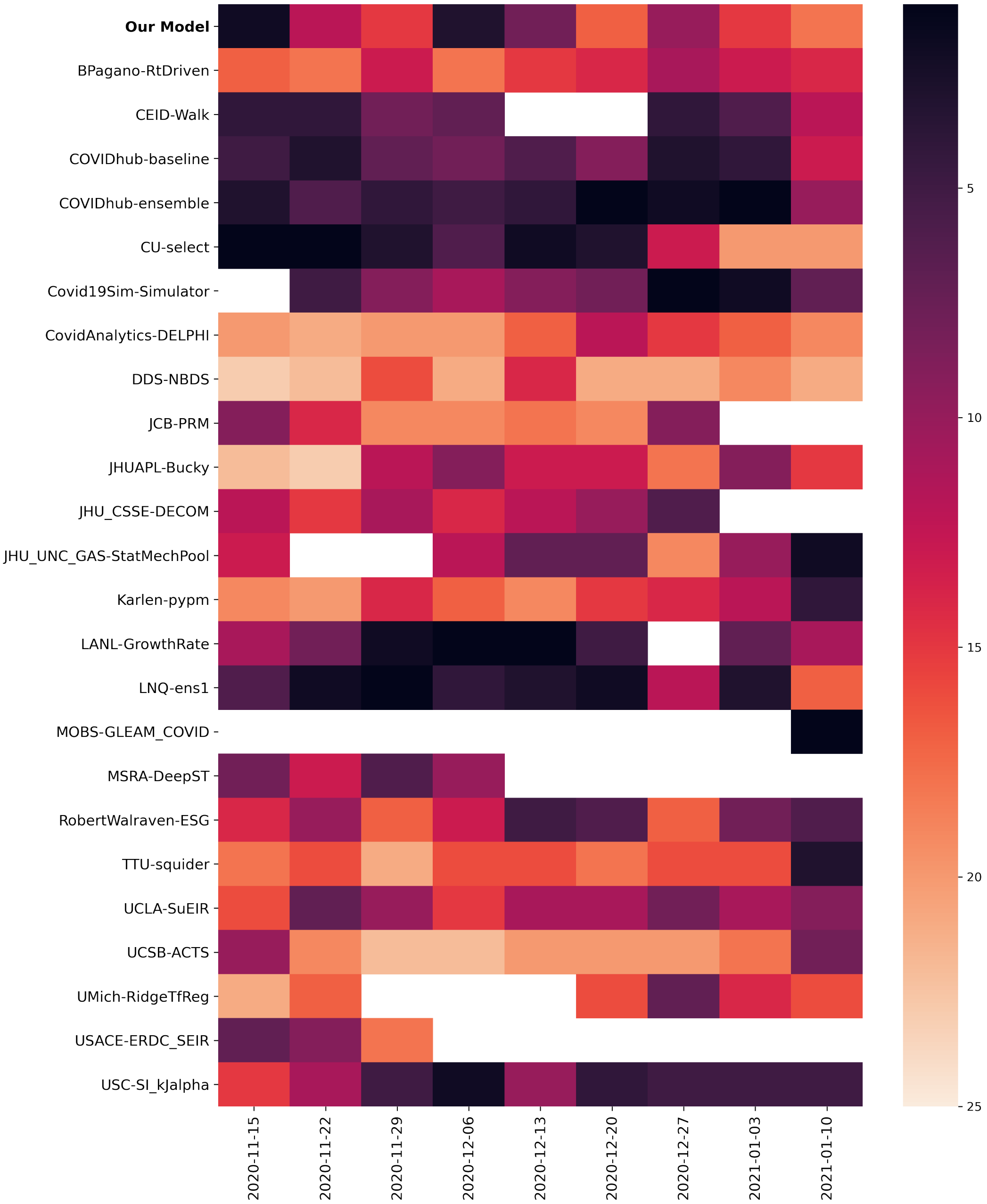

Supplementary Figure 9 | Model rankings for incident cases MAPE. The darker the color, the higher the ranking of the model is for the corresponding prediction date.

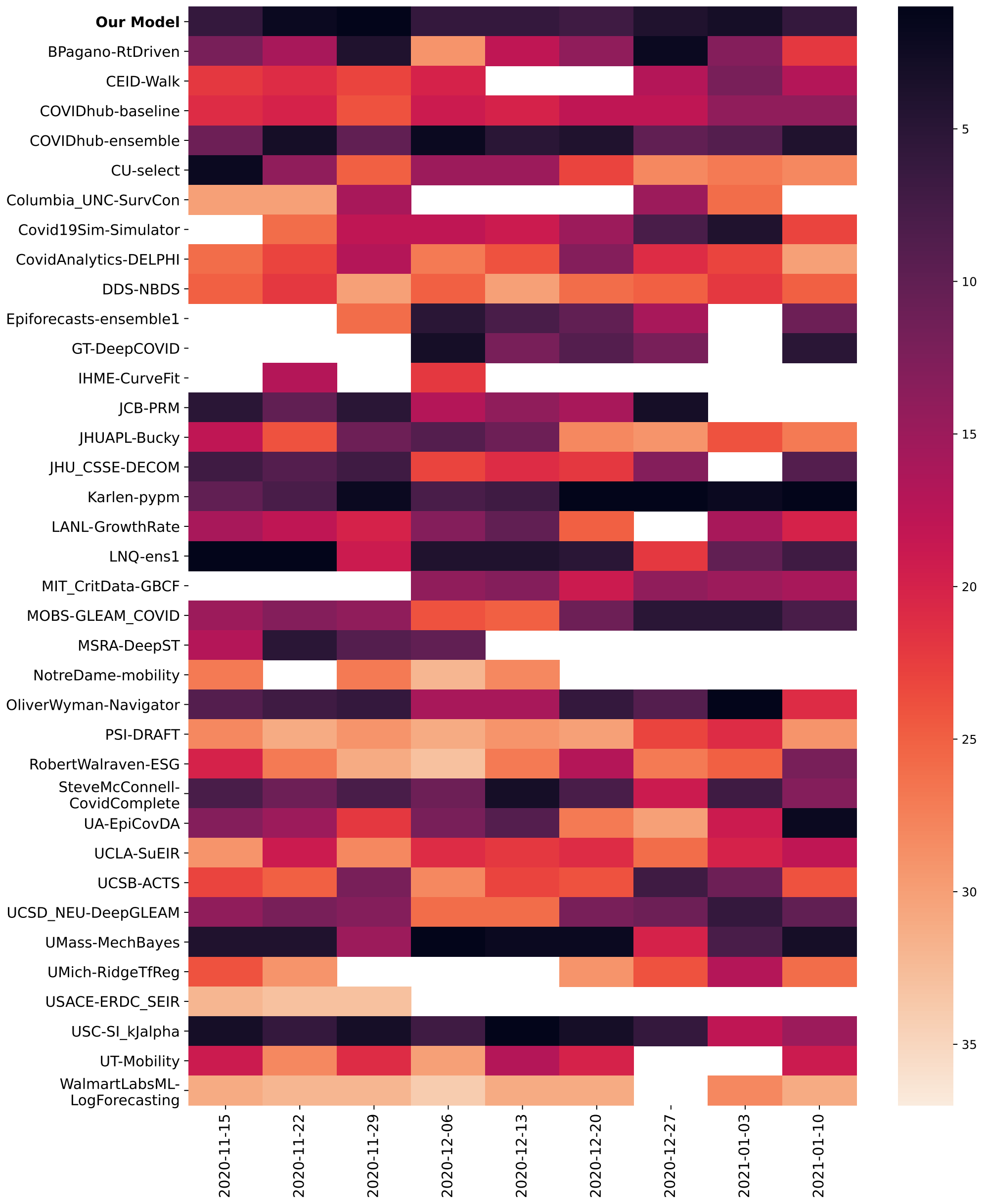

Supplementary Figure 10 | Model rankings for cumulative death MAE in the prospective evaluation period. The darker the color, the higher the ranking of the model is for the corresponding prediction date.

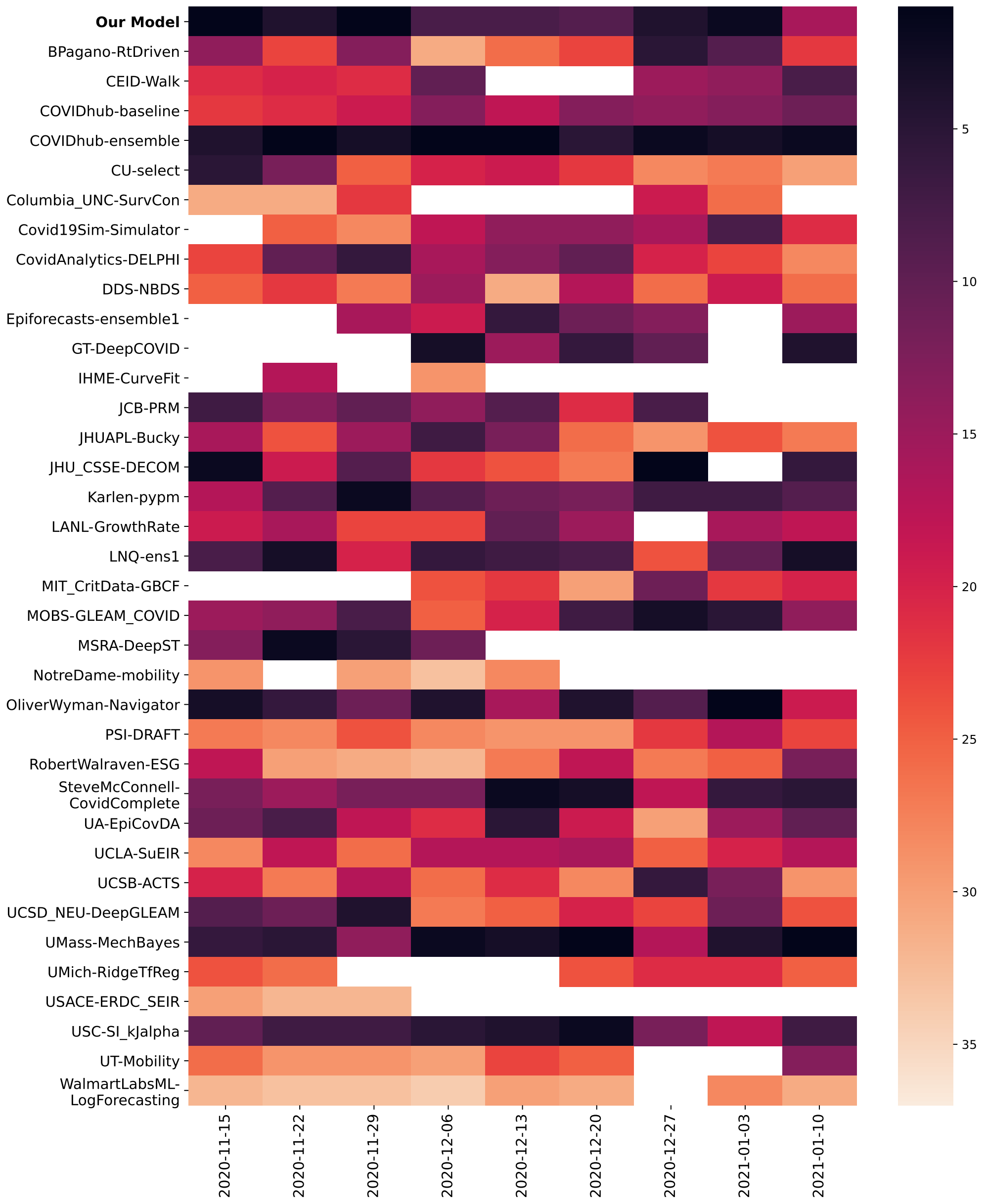

Supplementary Figure 11 | Model rankings for cumulative death MAPE in the prospective evaluation period. The darker the color, the higher the ranking of the model is for the corresponding prediction date.

Statistical comparison of forecasting accuracy

We compare our models' US state forecasts to those of alternative models' by applying the two-sided Diebold-Mariano (DM) test with the small sample size correction [2] to the MAE or MAPE averaged across all the locations for the dates when for both of the models produced forecasts. The DM test was used to compare the forecasts, rather than other statistical tests, because it accounts for the correlation of forecast errors across time [3]. Only models with more than 4 comparisons were included in the results. The p-values from the tests were adjusted using the Holm–Bonferroni method [4] to account for the multiple comparisons in each test. KPSS tests [5] were run on the differences in the loss metrics and rejections of the null hypothesis of stationary about either a constant level or a trend at the 0.05 level are also noted. For each of these tests, a positive DM statistic suggests that our models' forecasts were more accurate (i.e. had a smaller loss) than the competitor's forecasts while a negative statistic suggests that the inverse is true.

Tables 15 and 16 show the DM test results for the four week ahead forecasts of cumulative deaths in the US for MAE and MAPE respectively. When MAE is considered, only one of the DM statistics is negative. In other words, the only model's forecasts with a lower MAE than ours are the forecasts from the 'Karlen-pypm' model and that small difference is not statistically significant (p-value of 1 after adjustment and 0.977 before). Our forecasts had a significantly lower MAE, when adjusted p-value threshold of 0.05 is considered, in 13 of the 33 comparisons. When MAPE is compared the 'COVIDhub-ensemble' forecasts have a negative DM statistic, suggesting that they had a slightly lower MAPE, but this difference is not statistically significant with a p-value greater than 0.25 before p-value adjustment and 1 after. The better relative results of our model when evaluated with MAE vs. MAPE may be due to the fact that the loss function we used to train the model (see Methods), as it is not normalized with the absolute quantities. Neither model that scored better than our model using one metric had a DM statistic in the top 5 in the other metric, while our model was able to achieve consistently high results irrespective of the specific error metric.

These findings are similar for the predictions of the number of incident deaths over the next four weeks and the comparisons using MAPE is shown in Table 17. Note that the MAE comparisons are the same as for cumulative cases. In addition to the 'COVIDhub-ensemble' forecasts, the 'GT-DeepCOVID' and 'UMass-MechBayes' forecasts also had a lower MAPE than ours when predicting incident deaths over the next four weeks. However, the difference was not statistically significant for any of the models (the smallest p-value before adjustment was 0.327). Although our model was optimized for cumulative metrics, its forecasts were among the top models for the number of incident deaths as well.

When other top models are considered, we observe that they are often either in 'black-box' (non-explainable) ensemble model form (such as 'COVIDhub-ensemble' and 'LNQ-ens1'), or complex variants of compartmental models, improved with more advanced variable fitting, appropriate prior assumptions and sometimes additional features similar to our model (e.g. 'USC-SI_kJalpha', 'Karlen-pypm', 'UMass-MechBayes'). Our explainable AI-augmented compartmental model, with its competitive accuracy, constitutes an appreciable alternative.

Tables 18 and 19 show the DM test results for the four week ahead forecasts of US state confirmed cases for MAE or MAPE, respectively. Compared to our models' performance on

Supplementary Table 15 | Diebold-Mariano test results for 4-week ahead predictions of cumulative deaths for US states using MAE as the loss. Each row represents an alternative forecasting model compared with our model. A positive DM statistic suggests that our models' forecasts were more accurate (i.e. had a smaller loss) than the competitor's forecasts, while a negative statistic suggests that the inverse is true.

Model	Comparisons	DM Statistic	p-value	Adj. p-value	KPSS Rejected
Karlen-pypm	9	-0.030	0.977	1	level
UMass-MechBayes	9	1.185	0.27	1	
USC-SI_kJalpha	9	1.217	0.258	1	
LNQ-ens1	9	1.396	0.2	1	
GT-DeepCOVID	5	1.406	0.233	1	level, trend
COVIDhub-ensemble	9	1.696	0.128	0.933	
CU-select	9	1.759	0.117	0.933	
epiforecasts-ensemble1	6	1.864	0.121	0.933	
JHUAPL-Bucky	9	1.960	0.086	0.835	
MOBS-GLEAM_COVID	9	1.976	0.084	0.835	level
SteveMcConnell-CovidComplete	9	2.600	0.032	0.405	
OliverWyman-Navigator	9	2.650	0.029	0.405	level
UA-EpiCovDA	9	2.698	0.027	0.405	
UCSD_NEU-DeepGLEAM	9	2.744	0.025	0.405	
JHU_CSSE-DECOM	8	2.790	0.027	0.405	
Covid19Sim-Simulator	8	3.000	0.02	0.359	
CEID-Walk	7	3.098	0.021	0.36	level
JCB-PRM	7	3.239	0.018	0.336	level
CovidAnalytics-DELPHI	9	3.544	0.008	0.151	level
BPagano-RtDriven	9	4.071	0.004	0.075	
TTU-squider	9	4.509	0.002	0.046	
LANL-GrowthRate	8	4.765	0.002	0.046	
UT-Mobility	7	5.336	0.002	0.042	
DDS-NBDS	9	5.639	< 0.001	0.012	level, trend
RobertWalraven-ESG	9	5.933	< 0.001	0.009	
UCLA-SuEIR	9	5.990	< 0.001	0.009	
WalmartLabsML-LogForecasting	8	6.745	< 0.001	0.007	
UCSB-ACTS	9	7.364	< 0.001	0.002	
MIT_CritData-GBCF	9	7.559	< 0.001	0.002	
PSI-DRAFT	9	8.564	< 0.001	0.001	
COVIDhub-baseline	9	11.701	< 0.001	< 0.001	
UMich-RidgeTfReg	6	18.463	< 0.001	< 0.001	level, trend
Columbia_UNC-SurvCon	8	18.553	< 0.001	< 0.001	level

cumulative deaths (see the previous section) our models' forecasts are relatively less accurate for confirmed cases in comparison to alternative models. The decreased accuracy is also apparent in the MAPE of forecasts of the number of additional confirmed cases over the next four weeks as shown in Table 20. This relative decrease in accuracy is largely due to our choice of overemphasizing on predicting deaths more accurately when the multi-objective optimization trade-off is considered (see Methods). As the data quality of the confirmed case counts is lower than the death counts (due to its dependence on the testing rates and the reporting differences across locations), we choose to tune our model to fit better to the death counts than the confirmed case counts. We indeed empirically demonstrate that by increasing the confirmed case count coefficient, we can improve confirmed case forecasting accuracy, albeit a decrease in death forecasting accuracy.

In general, relatively large amounts of variability in performance is observed among the models

Supplementary Table 16 | Diebold-Mariano test results for 4-week ahead predictions of cumulative deaths for US states using MAPE as the loss. Each row represents an alternative forecasting model compared with our model. A positive DM statistic suggests that our models' forecasts were more accurate (i.e. had a smaller loss) than the competitor's forecasts, while a negative statistic suggests that the inverse is true.

Model	Comparisons	DM Statistic	p-value	Adj. p-value	KPSS Rejected
COVIDhub-ensemble	9	-1.210	0.261	1	
GT-DeepCOVID	5	0.098	0.926	1	level, trend
UMass-MechBayes	9	0.337	0.745	1	
SteveMcConnell-CovidComplete	9	1.144	0.286	1	
epiforecasts-ensemble1	6	1.244	0.269	1	
CEID-Walk	7	1.363	0.222	1	level
MOBS-GLEAM_COVID	9	1.393	0.201	1	
Covid19Sim-Simulator	8	1.621	0.149	1	
LNQ-ens1	9	1.713	0.125	1	
LANL-GrowthRate	8	1.729	0.127	1	level, trend
COVIDhub-baseline	9	1.801	0.109	1	level
UT-Mobility	7	1.829	0.117	1	level
Columbia_UNC-SurvCon	8	1.862	0.105	1	level, trend
UMich-RidgeTfReg	6	1.897	0.116	1	
USC-SI_kJalpha	9	1.973	0.084	1	
Karlen-pypm	9	2.131	0.066	1	
UCLA-SuEIR	9	2.212	0.058	1	
RobertWalraven-ESG	9	2.317	0.049	0.934	
CovidAnalytics-DELPHI	9	2.634	0.03	0.6	level
TTU-squider	9	2.705	0.027	0.564	level
UA-EpiCovDA	9	2.832	0.022	0.486	
BPagano-RtDriven	9	2.888	0.02	0.466	
PSI-DRAFT	9	3.267	0.011	0.274	
UCSB-ACTS	9	3.423	0.009	0.226	level
DDS-NBDS	9	3.737	0.006	0.149	level, trend
CU-select	9	3.868	0.005	0.128	
WalmartLabsML-LogForecasting	8	4.280	0.004	0.102	level
JHUAPL-Bucky	9	4.555	0.002	0.054	
UCSD_NEU-DeepGLEAM	9	4.804	0.001	0.04	
MIT_CritData-GBCF	9	5.030	0.001	0.031	
OliverWyman-Navigator	9	9.693	< 0.001	< 0.001	level
JCB-PRM	7	10.311	< 0.001	0.002	level
JHU_CSSE-DECOM	8	11.103	< 0.001	< 0.001	

across the prospective time range. In fact, none of the forecasts is better than our forecasts in a statistically significant way for a significance level of 0.1 for MAE or MAPE based on the adjusted p-values. Similar to cumulative death benchmarking, we observe that the two 'black-box' ensemble models, 'COVIDhub-ensemble' and 'LNQ-ens1', are among the top ones. All in all, such ensemble models constitute great alternatives when only the goal of forecasting accuracy is considered, yet their lack of explainability is a significant concern for epidemiological use. Among the other top models, 'Covid19Sim-Simulator' is an SEIR-variant that uses time-varying features, similar to ours. We note that 'Covid19Sim-Simulator' is also one of the top models for the cumulative death forecasts, supporting the importance of data-driven machine-learned compartmental modeling. Interestingly, 'COVIDhub-baseline', a simple trend following model, that does not have any complex machine learning components or that does not make use of any

**Supplementary Table 17 | ** Diebold-Mariano test results for predictions of incident deaths over the next 4 weeks for US states using MAPE as the loss. Each row represents an alternative forecasting model compared with our model. A positive DM statistic suggests that our models' forecasts were more accurate (i.e. had a smaller loss) than the competitor's forecasts, while a negative statistic suggests that the inverse is true.

Model	Comparisons	DM Statistic	p-value	Adj. p-value	KPSS Rejected
COVIDhub-ensemble	9	-1.044	0.327	1	
GT-DeepCOVID	5	-0.553	0.61	1	level, trend
UMass-MechBayes	9	-0.043	0.967	1	
SteveMcConnell-CovidComplete	9	1.151	0.283	1	
Karlen-pypm	9	1.215	0.259	1	
USC-SI_kJalpha	9	1.248	0.247	1	
epiforecasts-ensemble1	6	1.264	0.262	1	
MOBS-GLEAM_COVID	9	1.326	0.221	1	
LNQ-ens1	9	1.382	0.204	1	
CEID-Walk	7	1.443	0.199	1	level, trend
Covid19Sim-Simulator	8	1.591	0.156	1	
UT-Mobility	7	1.702	0.14	1	level
COVIDhub-baseline	9	1.921	0.091	1	level, trend
OliverWyman-Navigator	9	2.029	0.077	1	level
Columbia_UNC-SurvCon	8	2.152	0.068	1	level
LANL-GrowthRate	8	2.234	0.061	1	
RobertWalraven-ESG	9	2.305	0.05	0.901	
UA-EpiCovDA	9	2.381	0.044	0.845	
UCLA-SuEIR	9	2.464	0.039	0.781	
JHU_CSSE-DECOM	8	2.591	0.036	0.754	
CU-select	9	2.661	0.029	0.632	
PSI-DRAFT	9	3.622	0.007	0.156	
DDS-NBDS	9	3.793	0.005	0.137	level
CovidAnalytics-DELPHI	9	3.795	0.005	0.137	
JHUAPL-Bucky	9	3.863	0.005	0.129	
UCSD_NEU-DeepGLEAM	9	4.025	0.004	0.107	
BPagano-RtDriven	9	4.266	0.003	0.079	
UMich-RidgeTfReg	6	4.706	0.005	0.137	level
WalmartLabsML-LogForecasting	8	5.627	0.001	0.024	
TTU-squider	9	6.179	< 0.001	0.008	
JCB-PRM	7	8.297	< 0.001	0.005	level, trend
MIT_CritData-GBCF	9	8.576	< 0.001	0.001	
UCSB-ACTS	9	11.971	< 0.001	< 0.001	level

data, is one of the top models for cumulative case forecasting, suggesting that in the prospective evaluation period, the confirmed case increasing trends have similar patterns between consecutive weeks, and additional predictability is small. For death forecasting, 'COVIDhub-baseline' is one of the poorest models though. Vice versa, one of the top models for death forecasting, 'Karlen-pypm', is one of the worst for confirmed case forecasting.

Tables 21 and 22 show the DM tests between our model and alternative models, for 4-week ahead forecasts of the increase in hospitalized cases in US states, using the average MAE and MAPE respectively. This prediction is particularly important as it can be used for to plan and allocate hospital resources, and to prevent the overwhelming of the hospital systems. Our models' errors are smaller than any of the alternative models using either MAE or MAPE and this difference is statistically significant for all but one of the models using MAE and 75% of

Supplementary Table 18 | Diebold-Mariano test results for 4-week ahead predictions of cumulative confirmed cases for US states using MAE as the loss. Each row represents an alternative forecasting model compared with our model. A positive DM statistic suggests that our models' forecasts were more accurate (i.e. had a smaller loss) than the competitor's forecasts, while a negative statistic suggests that the inverse is true.

Model	Comparisons	DM Statistic	p-value	Adj. p-value	KPSS Rejected
COVIDhub-ensemble	9	-4.068	0.004	0.075	
COVIDhub-baseline	9	-2.829	0.022	0.421	
LNQ-ens1	9	-2.611	0.031	0.528	level
Covid19Sim-Simulator	8	-2.271	0.057	0.919	
LANL-GrowthRate	8	-2.084	0.076	1.000	
CEID-Walk	7	-1.915	0.104	1.000	
USC-SI_kJalpha	9	-1.473	0.179	1.000	level
RobertWalraven-ESG	9	-1.109	0.3	1.000	
JHU_CSSE-DECOM	7	-0.578	0.584	1.000	level
JHU_UNC_GAS-StatMechPool	7	-0.408	0.697	1.000	
UCLA-SuEIR	9	-0.320	0.757	1.000	
BPagano-RtDriven	9	0.277	0.789	1.000	level
CU-select	9	0.361	0.728	1.000	
TTU-squider	9	0.763	0.468	1.000	
UMich-RidgeTfReg	6	1.353	0.234	1.000	
DDS-NBDS	9	1.521	0.167	1.000	level
JHUAPL-Bucky	9	1.670	0.134	1.000	
CovidAnalytics-DELPHI	9	1.785	0.112	1.000	
JCB-PRM	7	2.069	0.084	1.000	
Karlen-pypm	9	2.655	0.029	0.523	
UCSB-ACTS	9	2.922	0.019	0.385	

the models using MAPE when an adjusted p-value threshold of less than 0.05 is considered. 'CU-select' and 'JHUAPL-Bucky', SEIR-variant models that also utilize interventions are among the other top ones for the hospitalization predictions.

In addition to the DM tests, we also compared the model rankings for each week directly which can be seen in the table below.

Supplementary Table 19 | Diebold-Mariano test results for 4-week ahead predictions of cumulative confirmed cases for US states using MAPE as the loss. Each row represents an alternative forecasting model compared with our model. A positive DM statistic suggests that our models' forecasts were more accurate (i.e. had a smaller loss) than the competitor's forecasts, while a negative statistic suggests that the inverse is true.

Model	Comparisons	DM Statistic	p-value	Adj. p-value	KPSS Rejected
LNQ-ens1	9	-3.047	0.016	0.318	level
COVIDhub-ensemble	9	-2.308	0.05	0.947	level
CEID-Walk	7	-2.169	0.073	1.000	
COVIDhub-baseline	9	-2.091	0.07	1.000	level
Covid19Sim-Simulator	8	-1.688	0.135	1.000	
LANL-GrowthRate	8	-1.030	0.337	1.000	
USC-SI_kJalpha	9	-0.661	0.527	1.000	level
UCLA-SuEIR	9	-0.386	0.71	1.000	level
JHU_UNC_GAS-StatMechPool	7	-0.225	0.829	1.000	
RobertWalraven-ESG	9	-0.172	0.868	1.000	level
JHU_CSSE-DECOM	7	0.136	0.897	1.000	level
CU-select	9	0.184	0.859	1.000	
UMich-RidgeTfReg	6	0.489	0.646	1.000	level
BPagano-RtDriven	9	0.520	0.617	1.000	level, trend
JHUAPL-Bucky	9	0.785	0.455	1.000	
TTU-squider	9	0.846	0.422	1.000	level, trend
Karlen-pypm	9	0.893	0.398	1.000	level
CovidAnalytics-DELPHI	9	1.007	0.343	1.000	level
UCSB-ACTS	9	1.542	0.162	1.000	
JCB-PRM	7	3.446	0.014	0.288	
DDS-NBDS	9	3.600	0.007	0.154	

Supplementary Table 20 | Diebold-Mariano test results for predictions of the incident confirmed cases in the next four weeks for US states using MAPE as the loss. Each row represents an alternative forecasting model compared with our model. A positive DM statistic suggests that our models' forecasts were more accurate (i.e. had a smaller loss) than the competitor's forecasts, while a negative statistic suggests that the inverse is true.

Model	Comparisons	DM Statistic	p-value	Adj. p-value	KPSS Rejected
LNQ-ens1	9	-2.869	0.021	0.438	level, trend
COVIDhub-ensemble	9	-1.670	0.134	1.000	level
CEID-Walk	7	-1.663	0.147	1.000	
COVIDhub-baseline	9	-1.487	0.175	1.000	
LANL-GrowthRate	8	-1.402	0.204	1.000	
Covid19Sim-Simulator	8	-1.164	0.283	1.000	
USC-SI_kJalpha	9	-0.907	0.391	1.000	level
UCLA-SuEIR	9	-0.523	0.615	1.000	
RobertWalraven-ESG	9	-0.491	0.636	1.000	
JHU_UNC_GAS-StatMechPool	7	-0.381	0.716	1.000	
JHU_CSSE-DECOM	7	0.182	0.861	1.000	level
CU-select	9	0.301	0.771	1.000	
UMich-RidgeTfReg	6	0.335	0.752	1.000	level
Karlen-pypm	9	0.431	0.678	1.000	level
BPagano-RtDriven	9	0.439	0.672	1.000	level, trend
TTU-squider	9	0.453	0.663	1.000	level
JHUAPL-Bucky	9	0.595	0.568	1.000	
CovidAnalytics-DELPHI	9	1.341	0.217	1.000	
UCSB-ACTS	9	1.388	0.202	1.000	
DDS-NBDS	9	2.142	0.065	1.000	
JCB-PRM	7	3.209	0.018	0.405	

Supplementary Table 21 | Diebold-Mariano test results for 4-week ahead predictions of the increase in hospitalized cases for US states using the average MAE as the loss. Each row represents an alternative forecasting model compared with our model. A positive DM statistic suggests that our models' forecasts were more accurate (i.e. had a smaller loss) than the competitor's forecasts, while a negative statistic suggests that the inverse is true.

Model	Comparisons	DM Statistic	p-value	Adj. p-value	KPSS Rejected
CU-select	9	1.209	0.261	0.523	level
JHUAPL-Bucky	9	3.710	0.006	0.018	level
USC-SI_kJalpha	8	7.994	< 0.001	< 0.001	level
LANL-GrowthRate	8	9.525	< 0.001	< 0.001	
Covid19Sim-Simulator	8	9.655	< 0.001	< 0.001	
COVIDhub-ensemble	6	11.704	< 0.001	< 0.001	
UCSB-ACTS	6	14.107	< 0.001	< 0.001	level
Karlen-pypm	9	22.950	< 0.001	< 0.001	

Supplementary Table 22 | Diebold-Mariano test results for 4-week ahead predictions of the increase in hospitalized cases for US states using the average MAPE as the loss. Each row represents an alternative forecasting model compared with our model. A positive DM statistic suggests that our models' forecasts were more accurate (i.e. had a smaller loss) than the competitor's forecasts, while a negative statistic suggests that the inverse is true.

Model	Comparisons	DM Statistic	p-value	Adj. p-value	KPSS Rejected
CU-select	9	0.964	0.363	0.363	
JHUAPL-Bucky	9	2.652	0.029	0.074	level
Karlen-pypm	9	5.220	0.001	0.003	level
LANL-GrowthRate	8	7.583	< 0.001	0.001	
COVIDhub-ensemble	6	8.555	< 0.001	0.002	
USC-SI_kJalpha	8	11.025	< 0.001	< 0.001	level
Covid19Sim-Simulator	8	13.871	< 0.001	< 0.001	
UCSB-ACTS	6	50.968	< 0.001	< 0.001	

Supplementary Table 23 | Forecast performance rankings with other available COVID-19 forecasting models. Rankings are shown for US State results MAPE, MAE and WIS across the prospective time interval for cumulative and incident deaths, cumulative and incident cases and the number of people admitted to the hospital. Metrics that were not available or could not be compared across any models are listed as N/A and the total number of forecasts in each comparison is shown in the number of models column. Our forecasts are compared with available models for the US; no such models were available for Japan.

		MAPE Rank	MAE Rank	WIS Rank	Number of Models
Cumulative Deaths	2020-11-15	1	6	9	34
	2020-11-22	4	2	1	35
	2020-11-29	1	1	1	35
	2020-12-06	8	6	1	36
	2020-12-13	8	6	2	33
	2020-12-20	9	7	5	33
	2020-12-27	4	4	5	31
	2021-01-03	2	3	6	29
	2021-01-10	16	6	5	32
Incident Deaths	2020-11-15	1	6	N/A	34
	2020-11-22	4	2	N/A	35
	2020-11-29	1	1	N/A	35
	2020-12-06	5	6	N/A	36
	2020-12-13	9	6	N/A	33
	2020-12-20	9	7	N/A	33
	2020-12-27	4	4	N/A	31
	2021-01-03	2	3	N/A	29
	2021-01-10	17	6	N/A	32
Cumulative Cases	2020-11-15	2	9	N/A	23
	2020-11-22	12	13	N/A	23
	2020-11-29	14	14	N/A	22
	2020-12-06	3	3	N/A	22
	2020-12-13	11	15	N/A	20
	2020-12-20	16	17	N/A	21
	2020-12-27	9	8	N/A	21
	2021-01-03	14	11	N/A	20
	2021-01-10	18	16	N/A	21
Incident Cases	2020-11-15	2	9	N/A	23
	2020-11-22	12	13	N/A	23
	2020-11-29	15	14	N/A	22
	2020-12-06	3	3	N/A	22
	2020-12-13	8	15	N/A	20
	2020-12-20	17	17	N/A	21
	2020-12-27	10	8	N/A	21
	2021-01-03	15	11	N/A	20
	2021-01-10	18	16	N/A	21
Hospitalized Inc.	2020-11-15	3	1	1	6
	2020-11-22	4	2	1	9
	2020-11-29	2	2	1	8
	2020-12-06	1	1	1	10
	2020-12-13	1	1	1	9
	2020-12-20	2	1	1	9
	2020-12-27	1	1	1	9
	2021-01-03	1	1	1	10
	2021-01-10	1	1	1	10

3 Supplementary Note 3: Counterfactual analysis

Figure 12 overviews the training and prediction with the proposed framework for counterfactual analysis. On each prediction date, using observed values for the case counts and features, a forecasting model is fitted. This forecasting model learns how to relate particular features to the compartmental outcomes, such as how a particular NPI would affect the number of exposed people. The forecasting model also needs the future values of the features, as it generates the predictions for all the time steps in the forecasting horizon conditioned on the values of the features in the forecasting horizon. For this purpose, we forecast the future values of the features, as explained in Methods. Using these forecasted values, we obtain 'baseline compartmental forecasts', in other words, the most likely outcomes given the past observations. For counterfactual analysis, the forecasted features are overridden. Essentially, we are trying to answer the question of what would happen if a particular feature changes in this particular way during the forecasting horizon? The overriding may be employed for any of the forecasted features.

Evaluation of Counterfactual Analysis

Evaluations of counterfactual scenarios are difficult, as we do not know the underlying ground truth associated with counterfactual scenarios. To demonstrate the reliability of our framework when it is used to analyze the impacts of NPIs, we rely on two evaluation methods. Note that the impact of vaccinations are determined by the way we modify the compartmental modeling, rather than counterfactual analysis.

Using simulated data: Since outcomes of counterfactual interventions cannot be observed in the real world, our first evaluation approach is based on a simulator, that is capable of simulating outcomes for both factual and counterfactual scenarios. Our simulator uses features (such as NPIs) as inputs, and outputs confirmed cases and deaths. Using this simulator, data for both training and evaluation can be synthesized, and the forecasting model can be trained on the simulated training data. Then, the trained forecasting model can be used to generate counterfactual forecasts, and since we have access to the simulator that can also synthesize such counterfactual scenarios, we are able to directly quantify the counterfactual scenario generation reliability by comparing it to the simulated counterfactual outcomes. Figure 13 overviews the entire process of counterfactual evaluation using simulated data.

Evaluations on simulated data would be helpful in detecting either model problems (e.g., poor estimation of model weights) or data problems (e.g., school closures at a certain stage of COVID-19 did not happen sufficient number of times for the model to accurately learn their effect). As the simulator we use the proposed compartmental model, with fitted encoder weights and initial conditions. While fitting to observed data, we disable teacher forcing (see Methods) so that data-driven conditioning can be learned because ground truth data is not available for counterfactual inference, so there is nothing to condition on. To validate that the model without teacher forcing still fits the observed ground truth data reasonably well, we compare the original ground truth cumulative confirmed cases and deaths with the simulated data and found that MAPE was less than 30% for all forecasting dates.

Ideally, the error of our counterfactual estimates should not be worse than the error of baseline

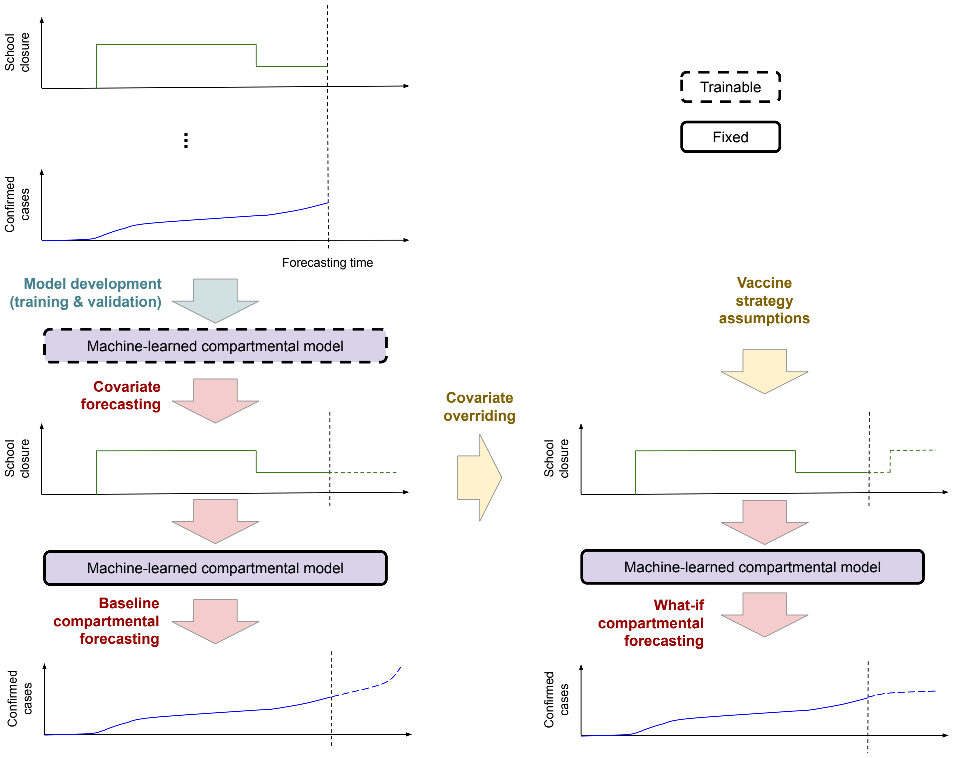

Supplementary Figure 12 | Overview of model development, baseline forecasting and what-if forecasting for counterfactual analysis. Using the past-observed values, the proposed machine-learned compartmental model is fitted. For forecasting, the future values of features are needed with the proposed compartmental model, for which they are separately forecasted. These feature forecasts can be used for "baseline compartmental forecasts", in other words, expected scenarios based on the current observations. Alternatively, they can be overriding the counterfactual assumptions on them, including the vaccine strategy related assumptions that are assumed to be non-existent for the baseline case. Eventually, with these overridden features, counterfactual scenarios for compartmental forecasts can be obtained.

factual estimates. To capture this, we propose the evaluation metric of the ratio between the 'factual MAPE' (MAPE between factual prediction and factual ground truth generated by the simulator) and the 'counterfactual MAPE' (MAPE between counterfactual prediction and counterfactual ground truth generated by the simulator). We evaluate this on 6 different counterfactual scenarios. Table 24 shows that for most scenarios and forecasting dates, the differences between factual and counterfactual MAPEs are less than 5%.

The proposed metric of the MAE ratio between factual and counterfactual has the limitation that if the intervention has a small effect on the progression of the disease, it would be nearly-optimal, close to 1, regardless of the quality of the counterfactual outcomes. To address, we also introduce an additional metric which measures how closely the forecasting model tracks the change in the simulated data due to the intervention, defined as the correlation between the prediction difference (counterfactual prediction - factual prediction) and the ground truth

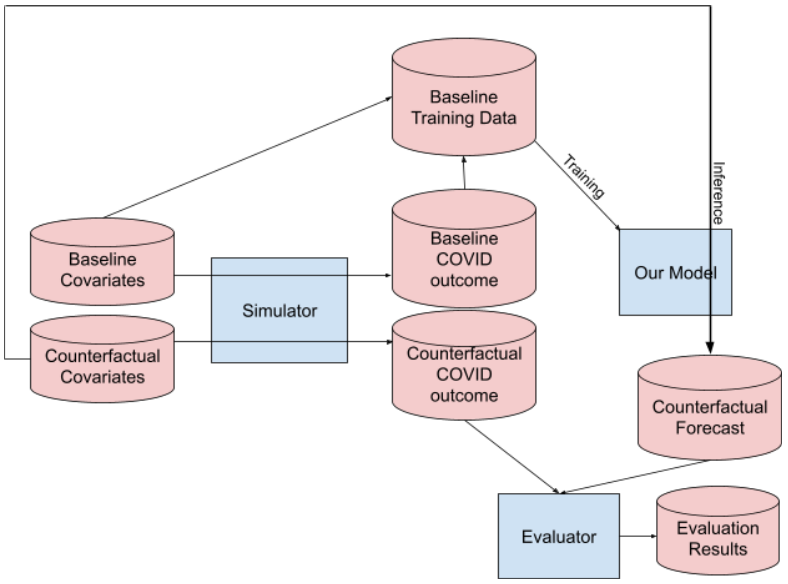

Supplementary Figure 13 | Block diagram of counterfactual evaluation using simulated data. First, a forecasting model is trained to be used as the simulator. With baseline features and counterfactual features as inputs to this simulator, baseline and counterfactual simulated data are generated by the simulator. Baseline simulated data is used to train the forecasting model. Using counterfactual features, forecasting model generates counterfactual forecasts, and these forecasts are evaluated against the simulated counterfactual ground truth.

Supplementary Table 24 | Ratios between factual MAPE and counterfactual MAPE in 6 different scenarios for the US model.

Forecasting date	9/7		9/28		10/19	
Compartments	Confirmed	Deaths	Confirmed	Deaths	Confirmed	Deaths
Mobility Increase (x10)	1.014	1.011	1.002	1.000	1.091	0.998
Mobility Decrease (90%)	0.998	0.999	1.000	1.000	0.997	1.000
School Closure	1.017	1.018	0.999	1.000	1.000	1.000
School Re-open	0.948	1.009	1.025	1.000	1.248	0.995
Restaurant Closure	1.000	1.000	0.974	0.996	1.342	0.998
Restaurant Re-open	1.001	1.001	1.074	1.009	1.054	1.001

difference generated by the simulator (counterfactual ground truth - factual ground truth) across different locations. We evaluate this metric on various what-if scenarios and forecasting dates. Table 25 shows that in most scenarios and forecasting dates, the Pearson correlations between prediction differences are higher than 0.6 which represents strong correlations. In some what-if scenarios, the correlations are even higher than 0.9 such as School re-opening. Note that the correlations can be different across different forecasting dates because the factual scenarios can be different at different forecasting dates and the model can fit them differently.

Using matched pairs: Matching is a technique sometimes used in epidemiological studies to

**Supplementary Table 25 | ** Pearson correlations between (counterfactual - factual prediction) and (counterfactual - factual ground truth) in 6 different scenarios.

Forecasting date	9/7		9/28		10/19	
Compartments	Confirmed	Deaths	Confirmed	Deaths	Confirmed	Deaths
Mobility Increase (10x)	0.667	0.578	0.693	0.716	0.692	0.627
Mobility Decrease (0.9x)	0.666	0.598	0.701	0.723	0.691	0.632
School Closure	0.459	0.813	0.888	0.797	0.664	0.558
School Re-open	0.526	0.610	0.943	0.531	0.948	0.924
Restaurant Closure	0.789	0.699	0.442	0.703	0.678	0.602
Restaurant Re-open	0.377	0.517	0.723	0.801	0.848	0.849

deduce causal effects from observational data[6, 7, 8, 9]. Matching method as an evaluation tool for our counterfactual analysis involves finding pairs of locations that are similar demographically and are in similar stages of the spread of COVID-19, but implement different NPI policies. The idea of matching is to eliminate the confounders - hidden variables other than the policies themselves that might be affecting the outcome.

On the US state-level data, we use an algorithm to find matched pairs of states based on similarity of confirmed cases, deaths, and mobility in the past 28 days, as well as similarity of their demographics (total population, population density, and income per capita). We looked for twins that implemented the same NPI policies in the past 28 days, but then their policies diverged. The most compelling matched pair of states we found were North Dakota and South Dakota. The two states have similar demographic metrics (see Table 26). They also had the same policies and COVID-19 metrics prior to 11/13/2020. After 11/13, North Dakota mandated mask wearing and closed restaurants, while South Dakota remained open (some counties in South Dakota introduced their own mask wearing mandates, but the state did not).

Supplementary Table 26 | Demographic metrics for North Dakota and South Dakota.

	North Dakota	South Dakota
Population	762062	884659
Population Density	4.265 ppl./sq. mi.	4.506 ppl./sq. mi.
Income Per Capita	$36002.7	$30873.1

Figure 14 shows confirmed confirmed cases, deaths, and hospitalization in North Dakota and South Dakota in the 28 days prior and 28 days following the introduction of lockdown policies in North Dakota. While 28 days is insufficient to see the full impact of these policies, we can see confirmed cases in North Dakota increasing slower than in South Dakota after the stricter policies are mandated, and the same trend is visible in the plots of deaths and hospitalized cases.

Once a matched pair of states is found, it can be used to evaluate the validity of our counterfactual predictions. More specifically, we can pretend for the purposes of evaluation that South Dakota applied North Dakota's stricter policies, while North Dakota applied South Dakota's less strict policies.

Figure 15 shows the counterfactual outcomes when North Dakota overrides its forecasted NPIs with the actual NPIs of South Dakota. North Dakota's forecasted confirmed cases, deaths, and

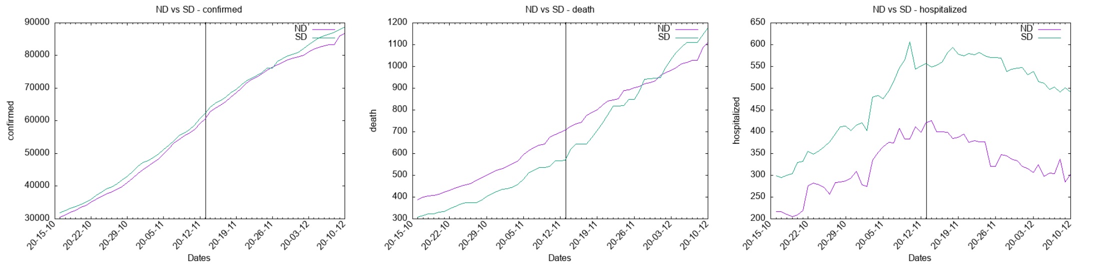

Supplementary Figure 14 | Confirmed cases, death, and hospitalizations in North Dakota (ND) vs. South Dakota (SD). The vertical line is the date on which ND introduced stricter lockdown policies.

hospitalizations increase, mirroring the higher increase in predicted confirmed cases, deaths, and hospitalizations we observe in South Dakota. Figure 16 shows the reversed counterfactual outcomes – if we replace the South Dakota's forecasted NPIs with North Dakota's stricter NPIs, the forecasts of confirmed cases, deaths, and hospitalizations are lowered for South Dakota, mirroring more closely the trend in the ground truth values of North Dakota. Overall, this matched-pair study improves the confidence on the reliability of our counterfactual analysis framework.

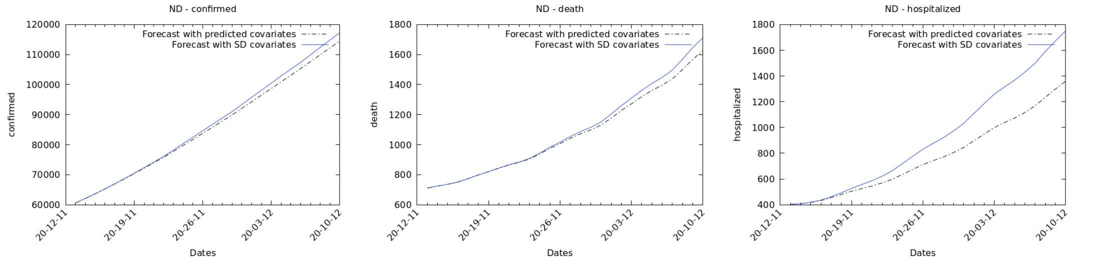

Supplementary Figure 15 | Confirmed cases, death, and hospitalizations for ND with SD overrides

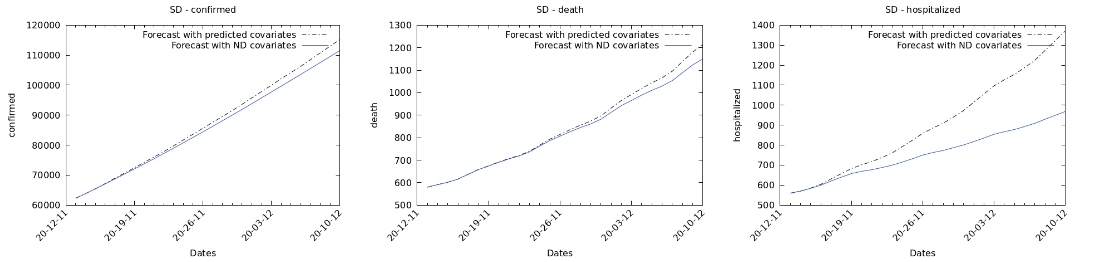

Supplementary Figure 16 | Confirmed cases, death, and hospitalizations for SD with ND overrides

Using past scenarios: A common way to apply counterfactuals in practice is to impart additional information about the future to the model. We may know that a large event scheduled for next week would dramatically increase mobility in a region, but the forecasting model has no way of predicting that this large event would occur. For example, the motorcycle rally on August 7th in Sturgis, SD in the USA caused a mobility increase not predicted by the baseline forecast

generated on August 5th. This baseline forecast should predict cases and deaths less accurately than a counterfactual scenario on August 5th with (a more accurate) increased mobility. We could have told the model about the increased mobility as a counterfactual and ask it to adjust its forecast accordingly. We propose to evaluate the model by overriding the forecasted future features (such as NPIs and mobility) to make its predictions with the actual observed values of these features. This technique is particularly effective when unpredictable events happen and the observed values deviate far from the forecasts.

To identify past scenarios where the models' forecasted features deviated from the actual values, we train the feature forecasting component of our model on an expanding time window and analyze the average prediction errors. Figure 18 (left) illustrates the feature forecasting performance measured by MAE across different training window sizes; the feature forecasting performance improves with increasing training dataset size. We pick 6 forecasting dates for evaluation in the range from 2020-04 to 2020-10: 2 dates from 2020-05 with inaccurate feature forecasting, 1 date from 2020-07 with moderately accurate feature forecasting, and 3 more recent dates with accurate feature forecasting. We then evaluated how well our framework performed when forecasting deaths and confirmed cases in the future window of 28 days for each one of those scenarios.

We then correlated the improvements in the accuracy of our models' forecast of deaths and confirmed cases due to using the correct overrides with the accuracy of our feature forecasts. We measured the improvement in the deaths/confirmed cases forecast accuracy with the ratio between the MAPE of forecasts based on actual features and MAPE of forecasts based on predicted features (lower MAPE ratio corressponds to more improvement in accuracy). We show the Pearson correlation coefficient of the MAPE ratio and the MAE of feature forecasts in Figure 18 (right) and Table 27. We expect that the counterfactual overrides based on the true feature values would help the model more when the original feature forecasts it based its predictions on were far from the true values, as observed in Figure 18. Note the negative correlation between the MAPE ratio and the feature MAE, indicating that higher feature MAE (worse feature predictions), results in greater improvement in the accuracy of predicted deaths and confirmed cases (i.e. lower MAPE ratio) when the ground truth feature values are applied as the counterfactual overrides. The Pearson correlation coefficients between MAPE ratio and feature MAE are -0.998 and -0.934 for death and confirmed cases predictions, respectively.

Figure 17 exemplifies forecasts with actual features and predicted features for three states (CA, IL, PA) for future 28 days in 2020-05. Forecasts move much closer to the ground truth when we override the forecasted features with the actual observed values.

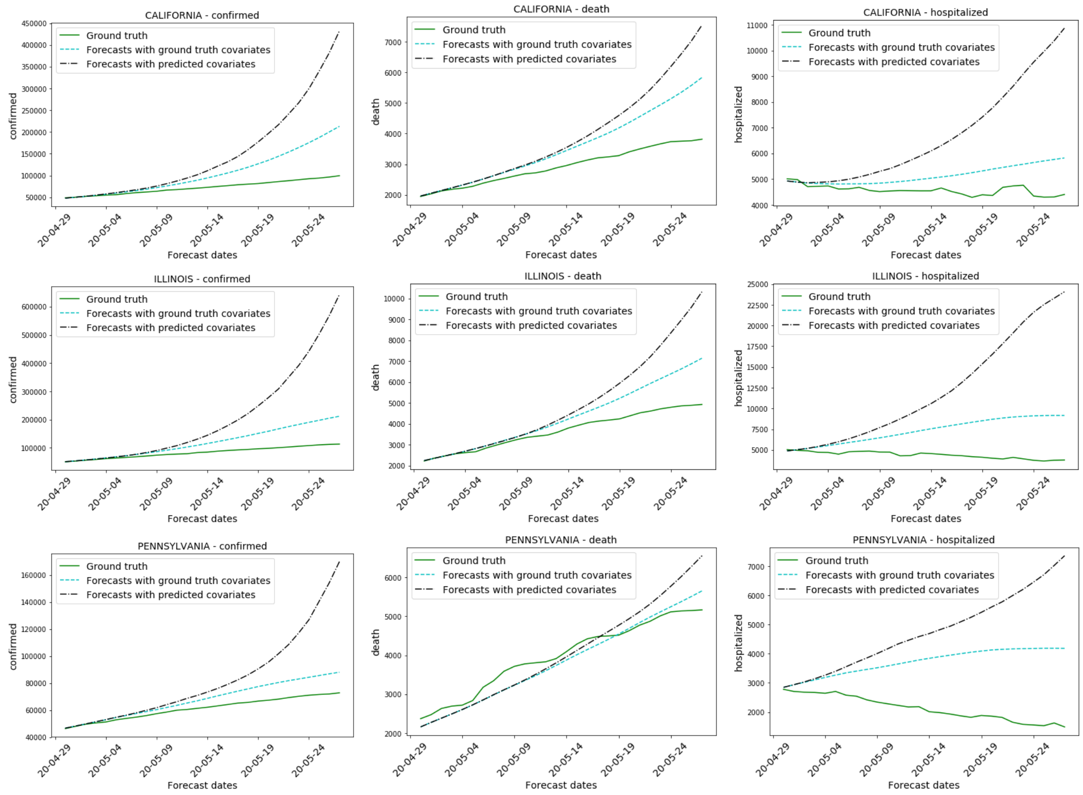

Supplementary Figure 17 | Confirmed cases, death, and hospitalization forecasts with actual features and predicted features in comparison to ground truth. Each row illustrates the forecasting results in California, Illinois, and Pennsylvania, respectively.

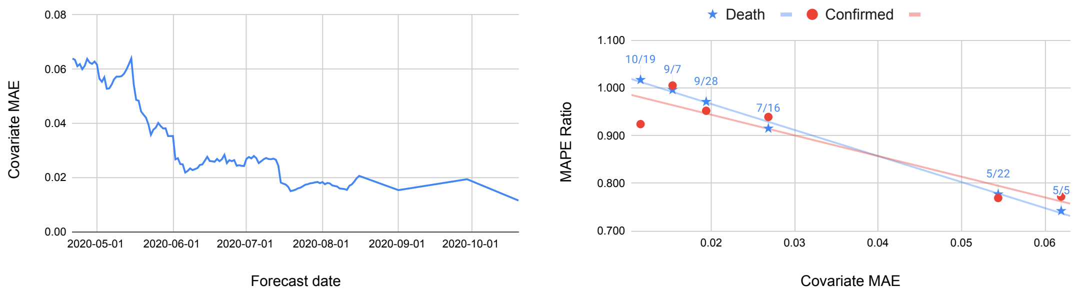

Supplementary Figure 18 | (left) feature forecasting MAE, (right) feature forecasting MAE vs. what-if metric in terms of the death and confirmed MAPE ratio of inference with ground truth features over inference with forecasted features.

Supplementary Table 27 | Forecasting performance improvements with historical ground truth feature. MAE/MAPE ratio = MAE/MAPE with ground truth feature / MAE with forecasted feature for the US model.

Forecasting date	MAE Ratio		MAPE ratio		MAE of feature forecasts
	Confirmed	Deaths	Confirmed	Deaths	
5/5	0.539	0.759	0.743	0.772	0.062
5/22	0.646	0.734	0.777	0.769	0.054
7/16	0.877	0.957	0.915	0.939	0.026
9/7	0.899	0.994	0.996	1.005	0.015
9/28	0.968	0.956	0.971	0.952	0.019
10/19	0.996	0.970	1.017	0.924	0.011

Counterfactual Analysis results

Using the counterfactual analysis methods described, we can observe the predicted differences in case numbers for different individual NPIs (Table 29), as well as multiple NPIs combined at once (Table 28). In these tables, we find that school closures are associated with the highest reduction in predicted exposed counts among all NPIs, and that joint application of multiple NPIs are observed to be much more effective than each individually.

The effect of delaying NPIs can be substantial. We find that a 7-day delay in applying all NPIs (compared to applying on the first day of forecasting horizon) is associated with a predicted increase of 32438 cases over 28 days in US. In Japan, a 7-day delay for medium mobility restrictions is associated with a predicted 45% reduction in confirmed cases. The effect size remains consistent when both weaker and stronger NPIs were considered Table 30.

Supplementary Table 28 | Counterfactual analysis for combined mobility restrictions in Japan In a combined scenario, we decrease a certain percentage (strong: 90%, medium: 60%, weak: 30%) of the mobility for Park, Work, Transit, Grocery, and Retail together. We also increase a certain percentage (strong: 300%, medium: 200%, weak; 100%) of the mobility for residential, to reflect the altered behavioral patterns associated with mobility restrictions. The numbers represent the increase in confirmed cases predicted to occur over the next 4 weeks.

	Strong	Medium	Weak
Overall	-97115 (-16.6%)	-73997 (-12.8%)	-42115 (-7.4%)
Tokyo	-27514 (-24.4%)	-20947 (-18.6%)	-11893 (-10.6%)
Chiba	-22453 (-43.1%)	-16975 (-32.6%)	-9511 (-18.3%)
Kanagawa	-10210 (-24.0%)	-7859 (-18.5%)	-4543 (-10.7%)
Fukuoka	-10953 (-37.3%)	-8249 (-28.1%)	-4619 (-15.7%)
Osaka	-5976 (-19.2%)	-4550 (-14.6%)	-2599 (-8.4%)
Saitama	-4204 (-22.1%)	-3248 (-17.1%)	-1890 (-10.0%)
Hyogo	-2901 (-24.3%)	-2248 (-18.8%)	-1316 (-11.0%)
Kyoto	-3011 (-30.4%)	-2265 (-22.9%)	-1268 (-12.8%)

Supplementary Table 29 | Counterfactual analysis for individual mobility restrictions in Japan The change in case numbers associated with instituting strong measures to reduce mobility in Japan. We decrease 90% of the mobility to areas allocated to Park, Work, Transit, Grocery, and Retail in the mobility data separately. We also separately increase the mobility to residential by 300% to reflect the altered behavioral patterns associated with mobility restrictions. The numbers represent the increase in confirmed cases predicted to occur over the next 4 weeks.

	Park	Work	Transit	Grocery	Retail	Residential
Overall	-5443 (-1.0%)	-18943 (-3.4%)	-8013 (-1.5%)	-18894 (-3.5%)	-20833 (-3.7%)	-56311 (-9.7%)
Tokyo	-1610 (-1.4%)	-5328 (-4.7%)	-2215 (-2.0%)	-5195 (-4.6%)	-5859 (-5.2%)	-16018 (-14.2%)
Chiba	-1098 (-2.1%)	-4186 (-8.0%)	-1750 (-3.4%)	-4093 (-7.9%)	-4598 (-8.8%)	-13076 (25.1%)
Kanagawa	-606 (-1.4%)	-2116 (-5.0%)	-890 (-2.1%)	-2090 (-4.9%)	-2329 (-5.5%)	-5927 (-13.9%)
Fukuoka	-606 (2.1%)	-2057 (-7.0%)	-893 (-3.0%)	-2127 (7.2%)	-2281 (-7.8%)	-6097 (-20.8%)
Osaka	-329 (-1.1%)	-1202 (-3.9%)	-487 (-1.6%)	-1204 (-3.9%)	-1282 (-4.1%)	-3440 (-11.1%)
Saitama	-245 (-1.3%)	-834 (-4.4%)	-379 (-2.0%)	-904 (-4.8%)	-970 (-5.1%)	-2475 (-13.0%)
Hyogo	-170 (-1.4%)	-595 (-5.0%)	-262 (-2.2%)	-612 (-5.1%)	-685 (-5.7%)	-1724 (-14.4%)
Kyoto	-165 (-1.7%)	-576 (-5.8%)	-241 (-2.4%)	-583 (-5.9%)	-609 (-6.2%)	-1687 (-17.0%)

Supplementary Table 30 | Counterfactual analysis on the predicted consequences of delays in applying NPIs. A comparison of predicted confirmed case reductions for NPIs applied immediately compared with if a delay of seven days is introduced. National forecasts are shown alongside those for the prefectures with the most confirmed cases. The 'Combined Medium' scenario is a 60% decrease in the Google Mobility values for Park, Work, Transit, Grocery, and Retail mobility and a 20% increase in Google Mobility values for Residential mobility. For the 'Combined Strong' scenario, the corresponding changes are a 90% decrease and a 30% increase, respectively.

Location	Combined Medium	Combined Medium Delayed 7-days	1 Combined Strong	Combined Strong Delayed 7-days
All Japan	-36521	-20203 (+45.0%)	-41829	-22891 (+45.2%)
Hokkaido	-279	-157 (+44.0%)	323	181 (+44.0%)
Saitama	-2072	-1118 (+46.0%)	-2375	-1274 (+46.4%)
Osaka	-908	-506 (+44.2%)	-1033	-579 (+44.0%)
Tokyo	-23066	-12990 +(43.6%)	-26157	-14569 (+44.3%)
Gunma	-59	-36 (+39.0%)	-69	-42 (+39.0%)

Counterfactual Analysis on Applying Vaccinations Drives with NPIs

Vaccination drives are ongoing around the globe. In the US, states have begun vaccinations for certain classes of occupations deemed vulnerable or crucial to controlling the pandemic. Here we run counterfactual analysis on the effect of vaccination, in combination with removing or reapplying the interventions–the State of Emergency in Japan and the 7 individual NPIs in the US, on the progression of the disease.

For the US, we consider:

1. The three vaccination rates from Supplementary Table 32: 0.1% (low), 0.5% (medium), and 1% (high) of the population per day.

2. The Rand corporation study on NPIs and their health and economic impacts[10] defined 5 levels of NPIs. From those levels, we focus on Levels 1, 3 and 5 for our study. The Levels and their corresponding definitions are described in Supplementary Table 31.

For Japan, we consider:

1. Four vaccination scenarios from Supplementary Table 32—vaccination rates of 0.1% (low), 0.5% (medium), 1% (high) and 2% (very high) of the population per day—compared to the baseline (expected trend-rate in the US and 0% in Japan).

2. Two Non-Pharmaceutical Interventions (NPI) scenarios—the State of Emergency being in force for all prefectures, and being withdrawn in all prefectures.

The application of one or more NPIs has a range of impacts and is subject to socioeconomic and compliance costs. Using high-frequency data such as electricity consumption and nitrogen dioxide emissions, Demirgüç-Kunt et al[11] show that the application of NPIs imposed an economic cost. Countries that applied NPIs early saw less of an impact over the period of the study as compared to countries that delayed their NPIs. An analogous compliance cost for NPIs were studied by Kantor and Kantor[12] and Seale et al[13]. Cross-sectional surveys were conducted to understand self-reported compliance with NPIs and the perceived complexity or cost in such compliance.

They showed that the perceived complexity had an impact on NPI compliance. For example, hand-washing was perceived to be less onerous than mask-wearing or school closures, hence there was more self-reported acceptance and compliance with hand-washing. Ghaffarzadegan[14] examines a different aspect of the application of NPIs. They show that applying multiple NPIs simultaneously is more beneficial than applying them individually. The nonlinear positive effects of multiple NPIs outweigh the costs incurred in their application and compliance.

NPI	Level 1	Level 3	Level 5
Shut Bars/Restaurants	Remove	Impose	Impose
Shut Non-Essential Businesses	Remove	Impose	Impose
Restrict Movement (Shelter-in-Place)	Remove	Remove	Impose
Close Schools	Impose	Impose	Impose
Emergency Declaration	Impose	Impose	Impose
Restrict Gatherings	Remove	Impose	Impose
Require Masks	Baseline	Baseline	Baseline

Supplementary Table 31 | Selected subset of the NPI levels in US states as defined by[10]. The 'Baseline' setting means that the corresponding NPI is unchanged from its ground truth values

Counterfactual Vaccination Drive	Description
Low vaccination rate	0.1% of the population is vaccinated daily (95% effectiveness[15, 16])
Medium vaccination rate	0.5% of the population is vaccinated daily (95% effectiveness[15, 16])
High vaccination rate	1% of the population is vaccinated daily (95% effectiveness[15, 16])
Very high vaccination rate	2% of the population is vaccinated daily (95% effectiveness[15, 16])

Supplementary Table 32 | Counterfactual Vaccination Drive levels in Japan and US states. The 'Baseline' setting means that the corresponding NPI is unchanged from its ground truth values

We apply these scenarios both individually, and in combinations considering the combinatorial space of 15 scenarios for Japan, and 16 scenarios for the US (Supplementary Figure 19). One additional, 'heterogeneous' scenario[17] is considered where 4 selected prefectures or states receive very high rate vaccinations, and the remaining prefectures or states receive medium rate vaccinations. The selected prefectures in Japan are Tokyo, Kanagawa, Aichi and Osaka. The selected states in the US are California, Florida, New York and Texas.

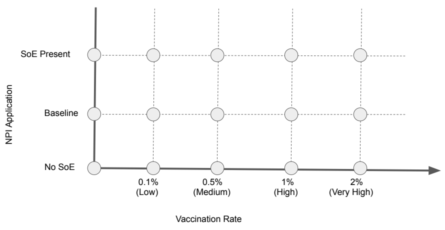

Japan

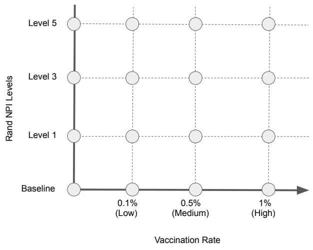

US States

Supplementary Figure 19 | Considered potential scenarios for Japan and the US.

Here, our baseline scenario is the scenario where no counterfactuals are applied, and the forecasted features are used in the model to make predictions. For example, Supplementary Figure 20 shows the forecasted State of Emergency feature for selected prefectures. In a prefecture, say Tokyo, where the State of Emergency is in force as of the training horizon of Feb. 4 2021, the forecasted feature could change value in the forecasting horizon.

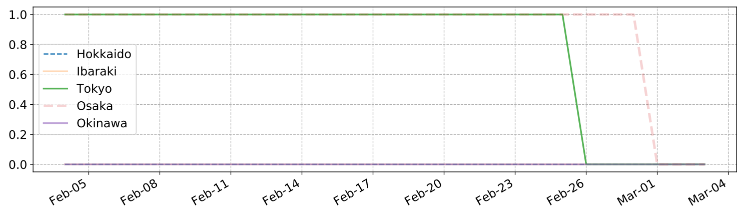

Supplementary Figure 20 | Forecasted State of Emergency feature for selected prefectures. Observe that for those prefectures where the current value is 1.0, the forecasted value does not remain at 1.0 in the forecasting horizon (Feb. 4 2021 through Mar. 3 2021).

Counterfactual Analysis Results for Japan

Supplementary Table 33 shows that vaccination drives by themselves may not be able to drive the disease to the disease-free equilibrium (all compartments tend towards 0 except for susceptible cases) after 28 days. The reduction in predicted exposed count ranges up to 28.4% in Ibaraki. However, in combination with NPIs, vaccination drives have bigger predicted impact, ranging from 75% to 76.5% in Ibaraki. The heterogeneous scenario, where very high vaccination rates are applied to Tokyo, Kanagawa, Aichi and Osaka, with the remaining prefectures receiving medium rates, show an overall national benefit of 2.1% fewer predicted exposed count. Tables 34 and 35 show this for confirmed cases and deaths in Japan respectively.

Counterfactual Analysis Results for the US

For the US model, the NPIs imposed by state governments are more granular and act on different aspects of public life, unlike the Japan model, which only has the binary-valued State of Emergency series. As described previously, we use 3 different levels of NPIs. We present counterfactual analysis on the effect of each scenario on predicted exposed count in Supplementary Table 36, and for susceptible in Supplementary Table 37. As an example, in California, the reduction benefit in the number of predicted exposed count can be as much as 33% reductions when high-rate vaccination drives are applied in tandem with imposing Level 5 NPIs universally. Of this total benefit, 23% is due to the vaccination drive (the reduction in exposed count in California between the 'Consistent' scenario and the 'High rate/Consistent NPI' scenario). A further 10% reduction in predicted exposed counts occurs from the 'High rate/Consistent NPI' scenario to the 'High rate/Level 5 NPI' scenario.

Vaccination Drives	Baseline	State of Emergency OFF	State of Emergency ON
Baseline (same 28-day trend)	Osaka: 5779<br>Hyogo: 4894<br>Okinawa: 3838<br>Hokkaido: 3253<br>Ibaraki: 3210	Osaka: 9853 (70.5%)<br>Hyogo: 11840 (141.95%)<br>Okinawa: 3838 (0.0%)<br>Hokkaido: 3253 (0.0%)<br>Ibaraki: 3210 (0.0%)	Osaka: 5652 (-2.2%)<br>Hyogo: 4453 (-9.01%)<br>Okinawa: 2262 (-41.06%)<br>Hokkaido: 2203 (-32.28%)<br>Ibaraki: 791 (-75.36%)
Low (0.1%)	Osaka: 5772 (-0.12%)<br>Hyogo: 4828 (-1.35%)<br>Okinawa: 3814 (-0.63%)<br>Hokkaido: 3237 (-0.49%)<br>Ibaraki: 3161 (-1.53%)	Osaka: 9781 (69.27%)<br>Hyogo: 11618 (137.39%)<br>Okinawa: 3814 (-0.63%)<br>Hokkaido: 3237 (-0.49%)<br>Ibaraki: 3161 (-1.53%)	Osaka: 5648 (-2.27%)<br>Hyogo: 4399 (-10.11%)<br>Okinawa: 2261 (-41.09%)<br>Hokkaido: 2202 (-32.31%)<br>Ibaraki: 789 (-75.42%)
Medium (0.5% population/day)	Osaka: 5744 (-0.59%)<br>Hyogo: 4568 (-6.66%)<br>Okinawa: 3717 (-3.15%)<br>Hokkaido: 3174 (-2.43%)<br>Ibaraki: 2969 (-7.51%)	Osaka: 9498 (64.37%)<br>Hyogo: 10748 (119.62%)<br>Okinawa: 3717 (-3.15%)<br>Hokkaido: 3174 (-2.43%)<br>Ibaraki: 2969 (-7.51%)	Osaka: 5632 (-2.53%)<br>Hyogo: 4185 (-14.47%)<br>Okinawa: 2256 (-41.22%)<br>Hokkaido: 2197 (-32.43%)<br>Ibaraki: 782 (-75.64%)
High (1.0%)	Osaka: 5710 (-1.19%)<br>Hyogo: 4249 (-13.16%)<br>Okinawa: 3598 (-6.25%)<br>Hokkaido: 3096 (-4.83%)<br>Ibaraki: 2737 (-14.74%)	Osaka: 9150 (58.33%)<br>Hyogo: 9705 (98.3%)<br>Okinawa: 3598 (-6.25%)<br>Hokkaido: 3096 (-4.83%)<br>Ibaraki: 2737 (-14.74%)	Osaka: 5613 (-2.87%)<br>Hyogo: 3923 (-19.84%)<br>Okinawa: 2249 (-41.4%)<br>Hokkaido: 2192 (-32.62%)<br>Ibaraki: 773 (-75.92%)
Very high (2.0%)	Osaka: 5641 (-2.37%)<br>Hyogo: 3634 (-25.75%)<br>Okinawa: 3365 (-12.32%)<br>Hokkaido: 2941 (-9.59%)<br>Ibaraki: 2299 (-28.38%)	Osaka: 8474 (46.63%)<br>Hyogo: 7763 (58.62%)<br>Okinawa: 3365 (-12.32%)<br>Hokkaido: 2941 (-9.59%)<br>Ibaraki: 2299 (-28.38%)	Osaka: 5574 (-3.55%)<br>Hyogo: 3414 (-30.24%)<br>Okinawa: 2236 (-41.74%)<br>Hokkaido: 2181 (-32.92%)<br>Ibaraki: 755 (-76.48%)
Heterogeneous scenario	Overall Benefit: -11436 vs -11203 (Medium rate vaccination and State of Emergency)<br>Osaka: -3.55%<br>Hyogo: -14.47%<br>Okinawa: -41.22%<br>Hokaido: -32.44%<br>Ibaraki: -75.65%		

Supplementary Table 33 | Counterfactual number of predicted exposed count on the 28th-day of the forecasting horizon for the top 5 prefectures by confirmed cases. Lower numbers are better outcomes. Negative percentages imply a reduction in counts, positive percentages imply an increase.

Key observations

From examining results from counterfactual simulations in US states and Japanese prefectures, we can draw certain initial observations:

1. Performing vaccinations, even at high rates, does not appreciably reduce the predicted number of predicted confirmed cases over the forecasting horizon of 28 days. The change in predicted deaths is even smaller after 28 days. However, reductions are observed in the predicted exposed and susceptible counts.

2. Keeping NPIs seems to play a crucial role during vaccination drives. The reduction in predicted confirmed cases is significant when the NPIs are kept along with vaccination efforts. The reductions in the predicted exposed or susceptible counts is larger.

3. The heterogeneous application of very high rate vaccinations applied to the more populated

Supplementary Table 34 | Counterfactual analysis on predicted cases while applying mobility restrictions alongside vaccinations in Japan. The change in predicted confirmed cases for five prefectures across different scenarios. The scenario with no mobility restrictions is normalized to a baseline where no overrides are applied. The scenarios with mobility restriction are referenced to a baseline where just a vaccination drive is applied. Low and high vaccination rate scenarios are modelled, representing 0.1% and 1% of the population vaccinated daily respectively and in both scenarios assuming a 95% effectiveness.

Vaccination Scenario	NPI Scenario	Tokyo	Kanagawa	Osaka	Saitama	Chiba
Forecasted Features Baseline	Forecasted Features Baseline	281847	79820	74888	43358	38199
0.1% pop. vaccinated/day	Forecasted Features Baseline	280481 (-0.49%)	79461 (-0.45%)	74862 (-0.03%)	43273 (-0.2%)	38108 (-0.[illegible]
0.1% pop. vaccinated/day	Weak Mobility Restrictions	263068 (-6.66%)	74791 (-6.3%)	74482 (-0.54%)	42098 (-2.91%)	36900 (-3.[illegible]
0.1% pop. vaccinated/day	Medium Mobility Restrictions	258057 (-8.44%)	73465 (-7.96%)	74366 (-0.7%)	41752 (-3.7%)	36552 (-4.[illegible]
0.1% pop. vaccinated/day	Strong Mobility Restrictions	255473 (-9.36%)	72777 (-8.82%)	74301 (-0.78%)	41568 (-4.13%)	36370 (-4.[illegible]
1% pop. vaccinated/day	Forecasted Features Baseline	276428 (-1.92%)	78400 (-1.78%)	74785 (-0.14%)	43021 (-0.78%)	37837 (-0.[illegible]
1% pop. vaccinated/day	Weak Mobility Restrictions	261728 (-7.14%)	74457 (-6.72%)	74455 (-0.58%)	42015 (-3.1%)	36812 (-3.[illegible]
1% pop. vaccinated/day	Medium Mobility Restrictions	257416 (-8.67%)	73308 (-8.16%)	74352 (-0.71%)	41712 (-3.8%)	36510 (-4.[illegible]
1% pop. vaccinated/day	Strong Mobility Restrictions	255160 (-9.47%)	72701 (-8.92%)	74294 (-0.79%)	41548 (-4.17%)	36350 (-4.[illegible]

Supplementary Table 35 | Counterfactual analysis on predicted deaths while applying mobility restrictions alongside vaccinations in Japan. The change in predicted COVID-19 associated deaths for five Japanese prefectures across different scenarios. The scenario with no mobility restrictions is normalized to a baseline where no overrides are applied. The scenarios with mobility restriction are referenced to a baseline where just a vaccination drive is applied. Low and high vaccination rate scenarios are modelled, representing 0.1% and 1% of the population vaccinated daily respectively and in both scenarios assuming a 95% effectiveness.

Vaccination Scenario	NPI Scenario	Tokyo	Kanagawa	Osaka	Saitama	Chiba
Forecasted Features Baseline	Forecasted Features Baseline	2920	837	1689	582	417
0.1% pop. vaccinated/day	Forecasted Features Baseline	2908 (-0.41%)	835 (-0.24%)	1689 (-0.0%)	581 (-0.0%)	417 (-0.0%)
0.1% pop. vaccinated/day	Weak Mobility Restrictions	2737 (-6.27%)	810 (-3.23%)	1683 (-0.3%)	575 (-1.2%)	412 (-1.2%)
0.1% pop. vaccinated/day	Medium Mobility Restrictions	2687 (-7.98%)	802 (-4.18%)	1682 (-0.41%)	573 (-1.55%)	411 (-1.68%)
0.1% pop. vaccinated/day	Strong Mobility Restrictions	2661 (-8.87%)	798 (-4.66%)	1681 (-0.47%)	571 (-1.72%)	410 (-1.92%)
1% pop. vaccinated/day	Forecasted Features Baseline	2874 (-1.58%)	830 (-0.84%)	1688 (-0.06%)	580 (-0.34%)	416 (-0.24%)
1% pop. vaccinated/day	Weak Mobility Restrictions	2726 (-6.64%)	808 (-3.46%)	1683 (-0.36%)	574 (-1.2%)	412 (-1.44%)
1% pop. vaccinated/day	Medium Mobility Restrictions	2682 (-8.15%)	801 (-4.18%)	1682 (-0.41%)	572 (-1.55%)	410 (-1.68%)
1% pop. vaccinated/day	Strong Mobility Restrictions	2658 (-8.94%)	798 (-4.66%)	1681 (-0.47%)	571 (-1.72%)	410 (-1.92%)

states, with medium rate vaccinations in the remaining states can result in fewer predicted cases and deaths.

Uncertainty Analysis for Counterfactual Predictions

As our models make predictions further out into the forecasting horizon, the outcomes become increasingly uncertain. Besides this aleatoric data uncertainty inherent in the forecasts, it is also important to be able to quantify the epistemic model uncertainty present for both baseline and counterfactual forecasts [18, 19, 20]. Such uncertainty arises when multiple models fit the data equally well yet yield potentially differing predictions. By measuring this model uncertainty, one can use it to build more trust in the impact of a counterfactual change – e.g. if the difference between the baseline and counterfactual predictions is much larger than the credible intervals due

Vaccination Drives	Baseline	Rand Level 1	Rand Level 3	Rand Level 5
Baseline (same 28-day trend)	Florida: 7085317	Florida: 5503106 (-22.33%)	Florida: 4295254 (-39.38%)	Florida: 3953503 (-44.2%)
	Texas: 3644234	Texas: 3379252 (-7.27%)	Texas: 3090946 (-15.18%)	Texas: 2923793 (-19.77%)
	California: 3320749	California: 3939997 (18.65%)	California: 2555856 (-23.03%)	California: 2226157 (-32.96%)
	New York: 2383868	New York: 2430069 (1.94%)	New York: 2290595 (-3.91%)	New York: 2070954 (-13.13%)
	Georgia: 2365059	Georgia: 1823232 (-22.91%)	Georgia: 1361138 (-42.45%)	Georgia: 1271457 (-46.24%)
Low (0.1%)	Florida: 6955601 (-1.83%)	Florida: 5448184 (-23.11%)	Florida: 4283798 (-39.54%)	Florida: 3951766 (-44.23%)
	Texas: 3442140 (-5.55%)	Texas: 3260573 (-10.53%)	Texas: 3042372 (-16.52%)	Texas: 2911744 (-20.1%)
	California: 3234428 (-2.6%)	California: 3804434 (14.57%)	California: 2532294 (-23.74%)	California: 2222985 (-33.06%)
	New York: 2198528 (-7.77%)	New York: 2225056 (-6.66%)	New York: 2157782 (-9.48%)	New York: 2040171 (-14.42%)
	Georgia: 2329769 (-1.49%)	Georgia: 1807612 (-23.57%)	Georgia: 1358821 (-42.55%)	Georgia: 1271135 (-46.25%)
Medium (0.5%)	Florida: 6444873 (-9.04%)	Florida: 5229632 (-26.19%)	Florida: 4238021 (-40.19%)	Florida: 3944822 (-44.32%)
	Texas: 3022900 (-17.05%)	Texas: 2983522 (-18.13%)	Texas: 2918479 (-19.92%)	Texas: 2879254 (-20.99%)
	California: 2897022 (-12.76%)	California: 3277258 (-1.31%)	California: 2438532 (-26.57%)	California: 2210308 (-33.44%)
	New York: 2037002 (-14.55%)	New York: 2050938 (-13.97%)	New York: 2022620 (-15.15%)	New York: 1996826 (-16.24%)
	Georgia: 2190393 (-7.39%)	Georgia: 1745359 (-26.2%)	Georgia: 1349559 (-42.94%)	Georgia: 1269847 (-46.31%)
High (1.0%)	Florida: 5824653 (-17.79%)	Florida: 4958994 (-30.01%)	Florida: 4180890 (-40.99%)	Florida: 3936144 (-44.45%)
	Texas: 2935710 (-19.44%)	Texas: 2924629 (-19.75%)	Texas: 2892860 (-20.62%)	Texas: 2870274 (-21.24%)
	California: 2556876 (-23.0%)	California: 2780332 (-16.27%)	California: 2335454 (-29.67%)	California: 2195779 (-33.88%)
	New York: 2004098 (-15.93%)	New York: 2010821 (-15.65%)	New York: 1997826 (-16.19%)	New York: 1985910 (-16.69%)
	Georgia: 2020171 (-14.58%)	Georgia: 1668052 (-29.47%)	Georgia: 1337992 (-43.43%)	Georgia: 1268238 (-46.38%)
Heterogeneous scenario	Overall Benefit: 29685292 vs 29776868 (Medium rate vaccination and Level 5 NPIs)			
	Florida: 3919800 (-44.68%)			
	Texas: 2859707 (-21.53%)			
	California: 2183781 (-34.24%)			
	New York: 1976346 (-17.09%)			
	Georgia: 1269847 (-46.31%)			

Supplementary Table 36 | Counterfactual analysis for the predicted exposed count on the 28th-day of the forecasting horizon for the top 5 states. Negative percentages imply a reduction in counts, positive percentages imply an increase.

Supplementary Table 37 | Counterfactual analysis for applying NPIs in tandem with vaccinations drives in USA states. The change is the predicted number of Susceptible Individuals on the 28th day of the forecasting horizon for the Top-5 states by baseline susceptible individuals. NPI levels are from Rand Corporation[10]. Negative percentages imply a reduction in counts, positive percentages imply an increase.

Vaccination Drives	Predicted features Baseline	Rand Level 1	Rand Level 3	Rand Level 5
Baseline	Illinois: 6122387	Illinois: 5033620 (-17.78%)	Illinois: 6038620 (-1.37%)	Illinois: 6319586 (3.22%)
	California: 6073129	California: 5260227 (-13.39%)	California: 7031898 (15.79%)	California: 7467760 (22.96%)
	Florida: 4732335	Florida: 6635598 (40.22%)	Florida: 8119648 (71.58%)	Florida: 8552248 (80.72%)
	Pennsylvania: 4520196	Pennsylvania: 4785996 (5.88%)	Pennsylvania: 5303852 (17.34%)	Pennsylvania: 5392968 (19.31%)
	Georgia: 3796765	Georgia: 4452852 (17.28%)	Georgia: 5027352 (32.41%)	Georgia: 5144156 (35.49%)
0.5% pop. vaccinated/day	Illinois: 4593573 (-24.97%)	Illinois: 3657703 (-40.26%)	Illinois: 4520088 (-26.17%)	Illinois: 4766381 (-22.15%)
	California: 1710801 (-71.83%)	California: 1181140 (-80.55%)	California: 2311234 (-61.94%)	California: 2626504 (-56.75%)
	Florida: 2814891 (-40.52%)	Florida: 4303318 (-9.07%)	Florida: 5541122 (17.09%)	Florida: 5918076 (25.06%)
	Pennsylvania: 3083091 (-31.79%)	Pennsylvania: 3300274 (-26.99%)	Pennsylvania: 3740164 (-17.26%)	Pennsylvania: 3819714 (-15.5%)
	Georgia: 2688499 (-29.19%)	Georgia: 3234374 (-14.81%)	Georgia: 3732658 (-1.69%)	Georgia: 3837885 (1.08%)

to model uncertainty, then the impact of the counterfactual simulation can be considered to be relatively more reliable. Notably, the amount of variance in predictions due to model uncertainty, and thus the relative amount of trust that can be placed on a counterfactual prediction, can vary dynamically across locations, time, and NPI values. This in turn provides further capability from a policy-making perspective, e.g. when comparing the effectiveness of different closures across locations.

In the following, we discuss methods and results of our uncertainty analysis for counterfactual predictions. In Section 7, we discuss uncertainty more broadly and examine its use in identifying and withholding unreliable predictions, which could also be extended to counterfactual predictions.

State Name	Scenario	Counts
Texas	Forecasted Features Baseline	2582026
Virginia	Forecasted Features Baseline	667035
California	Forecasted Features Baseline	598596
Ohio	Forecasted Features Baseline	563736
New York	Forecasted Features Baseline	469758
Texas	Decrease Mobility by 90%	2571370 (-0.41)
Virginia	Decrease Mobility by 90%	665642 (-0.21)
California	Decrease Mobility by 90%	597829 (-0.13)
Ohio	Decrease Mobility by 90%	562366 (-0.24)
New York	Decrease Mobility by 90%	469361 (-0.08)
Texas	Decrease Mobility by 90% after 7 days	2574306 (-0.3)
Virginia	Decrease Mobility by 90% after 7 days	666060 (-0.15)
California	Decrease Mobility by 90% after 7 days	598094 (-0.08)
Ohio	Decrease Mobility by 90% after 7 days	562744 (-0.18)
New York	Decrease Mobility by 90% after 7 days	469526 (-0.05)
Texas	All NPIs in force	1844165 (-28.58)
Virginia	All NPIs in force	544914 (-18.31)
California	All NPIs in force	525464 (-12.22)
Ohio	All NPIs in force	447292 (-20.66)
New York	All NPIs in force	441189 (-6.08)
Texas	All NPIs in force after 7 days	2018082 (-21.84)
Virginia	All NPIs in force after 7 days	581784 (-12.78)
California	All NPIs in force after 7 days	549385 (-8.22)
Ohio	All NPIs in force after 7 days	477844 (-15.24)
New York	All NPIs in force after 7 days	452624 (-3.65)

Supplementary Table 38 | Effect of delaying the application of mobility restrictions and NPIs in the US. Shown are the counterfactual outcomes for the predicted exposed count after the 28-day forecasting horizon for the top 5 US states ordered by baseline confirmed cases. Percent change from the forecasted features baseline are shown in parentheses. Negative percentages imply a reduction in counts, whereas positive percentages imply an increase.

We apply the following method for estimating the model uncertainty in the baseline and counterfactual predictions. First, we select a random seed to be used for model initialization, and then perform our full hyperparameter tuning run for 300 trials. As usual, we select the best trial based on the validation loss. Then, we repeat the above process using different random seeds for initializing the model. This produces an ensemble of best model trials equal in size to the number of different random seeds. Notably, since we employ Bayesian optimization for hyperparameter tuning, stochasticity in the model training is introduced via both the random initialization of the model, and in the stochasticity of the Bayesian optimization procedure itself. These sources of stochasticity yield an ensemble of high-performing models that may differ in their predictions. In order to limit the difference in metric performance of the final models, we order the set of best model trials in decreasing order by the validation loss, and select the top-5 trials as our final set of models. These form our baseline, and the spread in their performance is reported in Supplementary Table 40. Lastly, for each of these baseline models, we perform a counterfactual inference, and then record the variance in the predictions over the forecast window.

The selected counterfactual for this study was the application of all 7 of the NPIs enumerated in Supplementary Table 31.

The baseline and counterfactual confirmed case counts are shown in Supplementary Fig-

location_name	Scenario	Counts
California	Forecasted Features Baseline	14173271
Texas	Forecasted Features Baseline	8749567
Pennsylvania	Forecasted Features Baseline	7342288
Michigan	Forecasted Features Baseline	6656776
Georgia	Forecasted Features Baseline	6170734
California	Decrease Mobility by 90%	14174349 (0.01)
Texas	Decrease Mobility by 90%	8763605 (0.16)
Pennsylvania	Decrease Mobility by 90%	7343064 (0.01)
Michigan	Decrease Mobility by 90%	6657352 (0.01)
Georgia	Decrease Mobility by 90%	6171784 (0.02)
California	Decrease Mobility by 90% after 7 days	14173908 (0.0)
Texas	Decrease Mobility by 90% after 7 days	8758933 (0.11)
Pennsylvania	Decrease Mobility by 90% after 7 days	7342741 (0.01)
Michigan	Decrease Mobility by 90% after 7 days	6657123 (0.01)
Georgia	Decrease Mobility by 90% after 7 days	6171300 (0.01)
California	All NPIs in force	14278178 (0.74)
Texas	All NPIs in force	9742591 (11.35)
Pennsylvania	All NPIs in force	7397400 (0.75)
Michigan	All NPIs in force	6692778 (0.54)
Georgia	All NPIs in force	6229372 (0.95)
California	All NPIs in force after 7 days	14236135 (0.44)
Texas	All NPIs in force after 7 days	9447979 (7.98)
Pennsylvania	All NPIs in force after 7 days	7373938 (0.43)
Michigan	All NPIs in force after 7 days	6679216 (0.34)
Georgia	All NPIs in force after 7 days	6203820 (0.54)

Supplementary Table 39 | Effect of delaying the application of mobility restrictions and NPIs in the US. Shown are the counterfactual number of predicted susceptible counts after the 28-day forecasting horizon for the top 5 US states ordered by baseline confirmed cases. Percent change from the forecasted features baseline are shown in parentheses. Negative percentages imply a reduction in counts, whereas positive percentages imply an increase.

Metric	Mean across Top-5 Models	Standard Deviation across Top-5 Models
MAE of Deaths	19.1	0.13
MAE of Confirmed Cases	281.8	21.7

Supplementary Table 40 | Mean and standard deviation of the Mean Absolute Error metric on death and confirmed cases over the top-5 models selected by validation loss.

ures 22 through 28. The baseline and counterfactual exposed individuals counts are shown in Supplementary Figures 29 through 35.

From Supplementary Figure 1, we observe that the NPIs influence the contact rates that govern the transition from the susceptible to the exposed compartment. As a result, the predicted exposed compartment shows the largest change with imposing the NPIs (Supplementary Figures 29 through 35). We estimate less certainty about the counterfactual predictions in certain states such as Illinois, as compared to other states like Iowa (both presented in Supplementary Figure 30). This is evident from much wider uncertainty bands ($\pm$ 1 standard deviation band) for both baseline and counterfactual predictions for Illinois, and the significant overlap between them. On the other hand, we estimate more certainty about the predictions for Iowa, with narrower bands

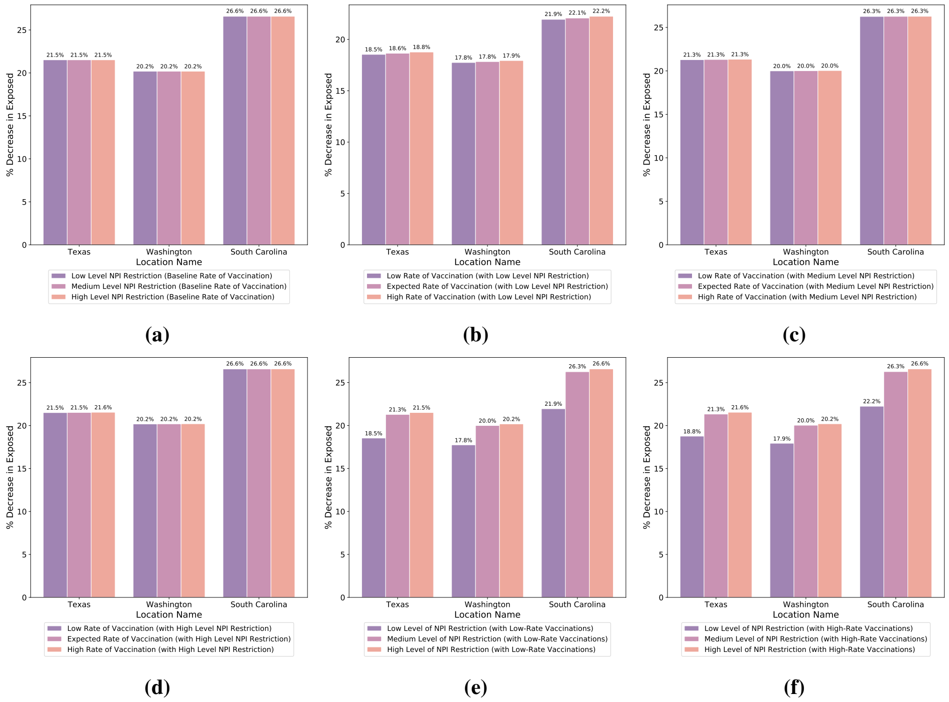

Supplementary Figure 21 | Counterfactual analysis on the predicted exposed counts with different vaccination rates for 3 selected US states (Supplementary Table 32) in tandem with NPIs (Supplementary Table 31), for the prediction date of March 1, 2021. Here, the baseline exposed counts are 69700, 67600 and 63700 for Texas, Washington and South Carolina, respectively. The percent decrease in predicted exposed counts compared to the baseline, are shown for the counterfactual cases that (a) the NPI Levels are changed over 1-Low, 3-Medium and 5-High, while the vaccination rates are the expected forecasted rates; (b) the NPI Levels are at 1-Low, while the vaccination rates are varied; (c) the NPI Levels are at 3-Medium, while the vaccination rates are varied; (d) the NPI Levels are at 5-High, while the vaccination rates are varied; (e) the NPI Levels are changed over 1-Low, 3-Medium and 5-High, while the vaccination rates are low; (f) the NPI Levels are changed over 1-Low, 3-Medium and 5-High, while the vaccination rates are high. *Note that in all cases, because of the uncertainty in counterfactual outcome is high - the 95% confidence intervals for baseline and counterfactual outcomes often overlap. This suggests that although the statistical significance on the directionality of the change would be high, the statistical significance on the exact amount of change would not be as high. Thus, it is important to stress that if used, the forecasts should be used alongside other information and with the support of epidemiology experts.*

and a relatively large separation between the uncertainty bands for the baseline forecasts versus the counterfactual forecasts.

Overall, by analyzing model uncertainty, we are able to provide additional information on the reliability of the counterfactual predictions. In general uncertainty is high, and for confirmed case predictions there is often overlap between the uncertainty bands for the baseline and counterfactual forecasts. This may be expected however, as the predictions are made only 28 days into the future. The results for the exposed compartment are better, and the uncertainty bands help those using the model better understand and weigh up the analyses obtained from our framework.

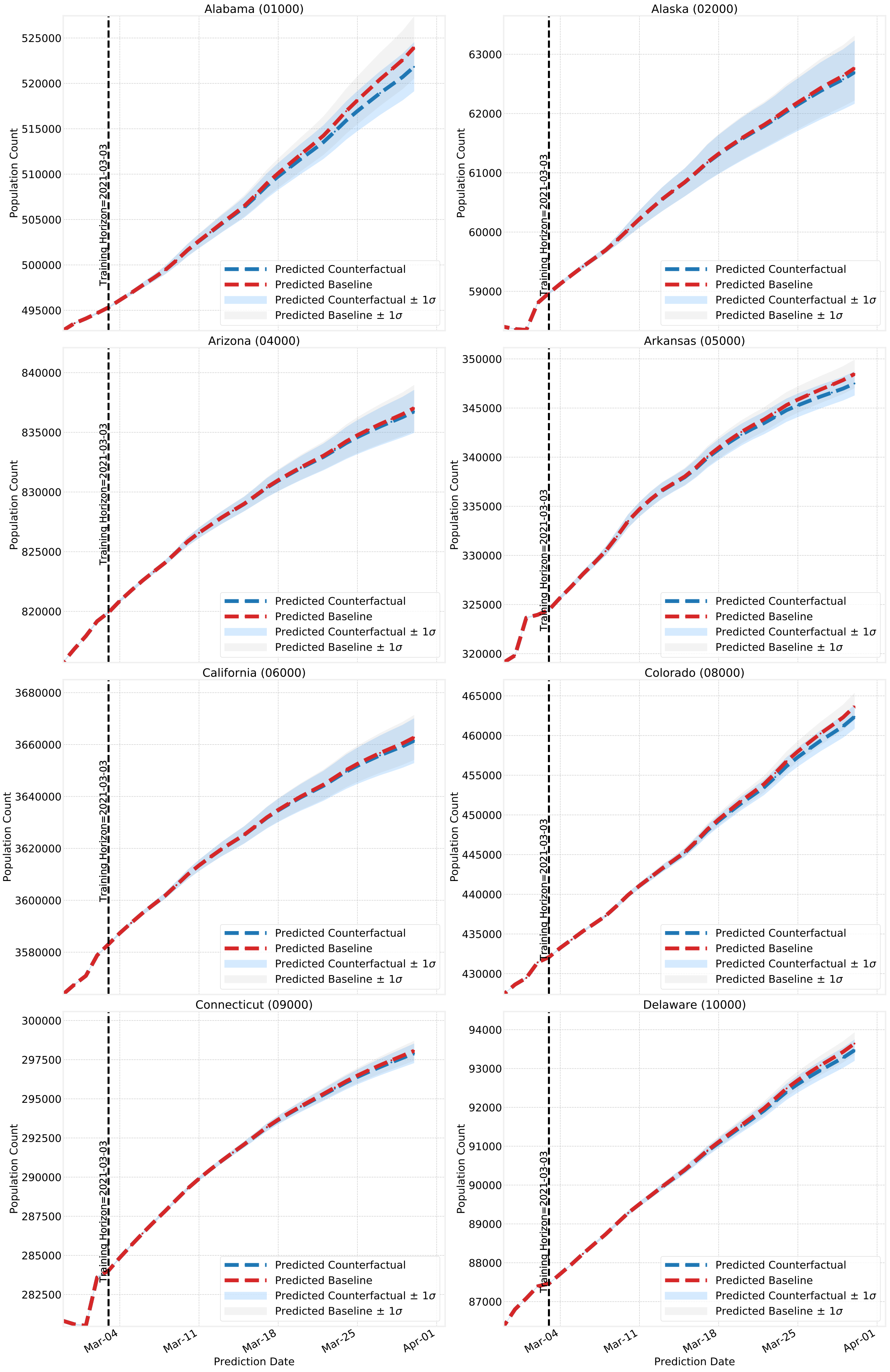

Supplementary Figure 22 | Baseline versus counterfactual prediction of confirmed cases for US states Alabama through Delaware. All the NPIs from Supplementary Table 31 are applied as a single counterfactual scenario. The shaded regions correspond to one standard deviation uncertainty bands.

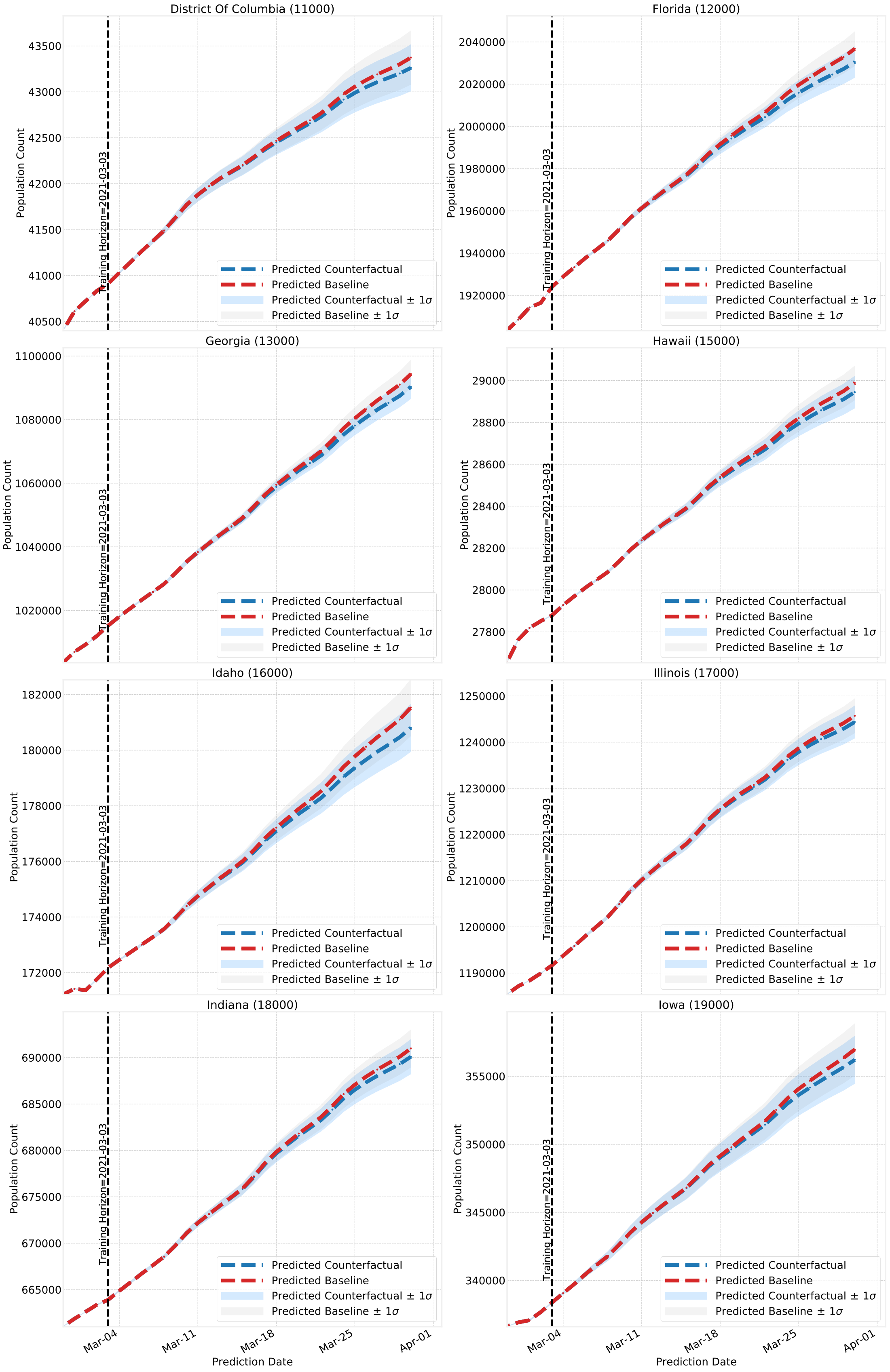

Supplementary Figure 23 | Baseline versus counterfactual prediction of confirmed cases for US states District of Columbia through Iowa. All the NPIs from Supplementary Table 31 are applied as a single counterfactual scenario. The shaded regions correspond to one standard deviation uncertainty bands.

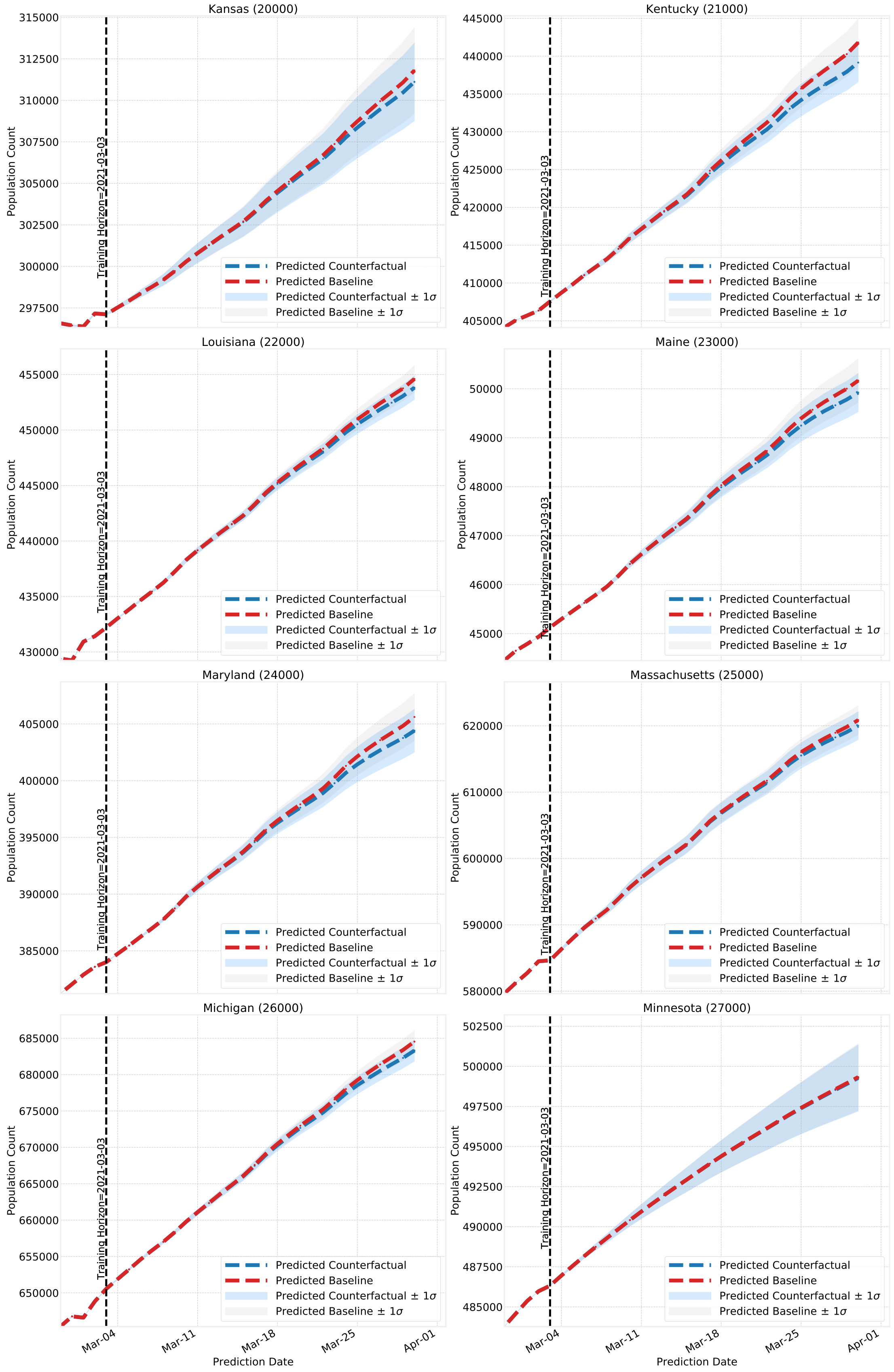

Supplementary Figure 24 | Baseline versus counterfactual prediction of confirmed cases for US states Kansas through Minnesota. All the NPIs from Supplementary Table 31 are applied as a single counterfactual scenario. The shaded regions correspond to one standard deviation uncertainty bands.

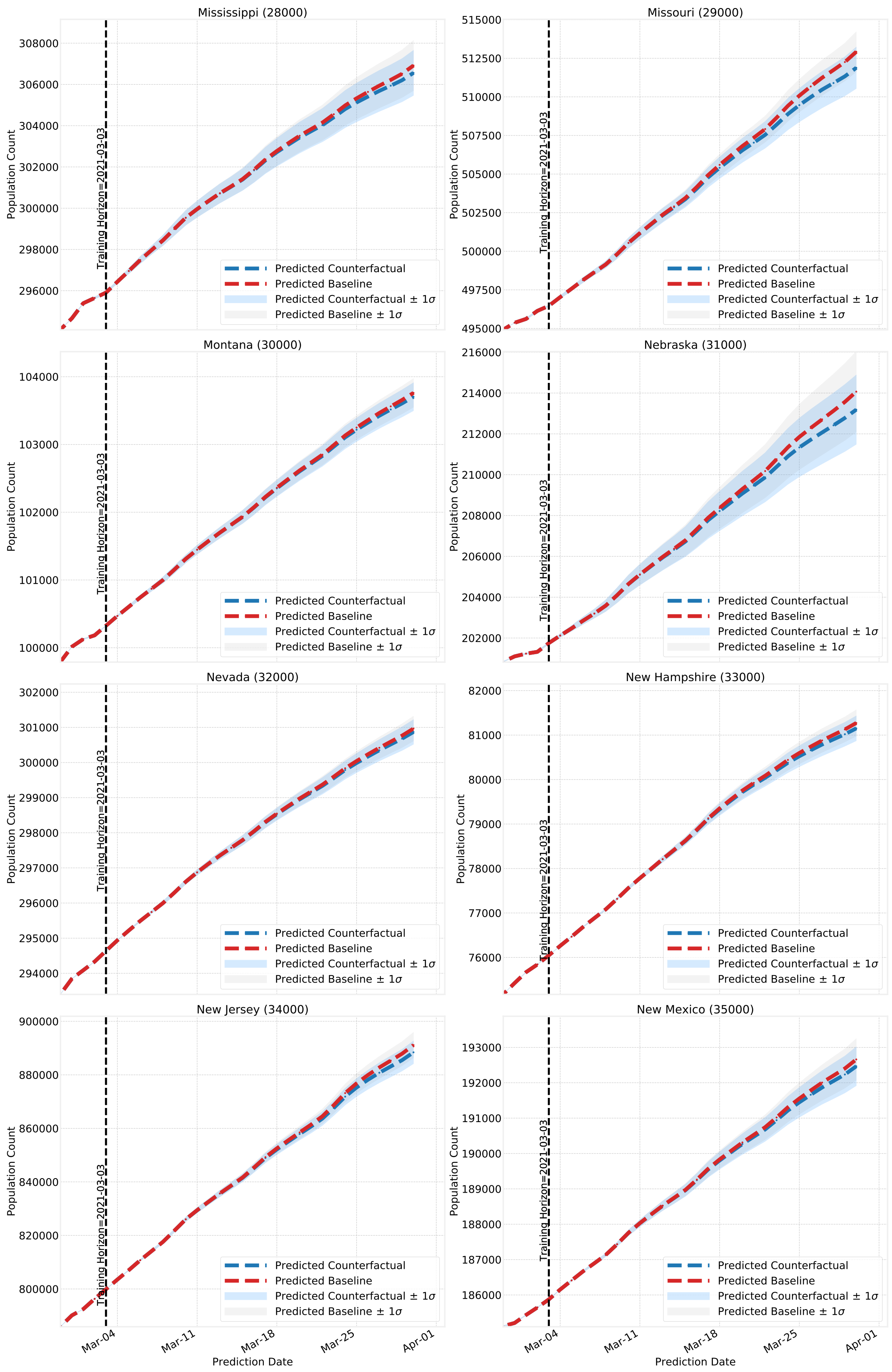

**Supplementary Figure 25 | ** Baseline versus counterfactual prediction of confirmed cases for US states Mississippi through New Mexico. All the NPIs from Supplementary Table 31 are applied as a single counterfactual scenario. The shaded regions correspond to one standard deviation uncertainty bands.

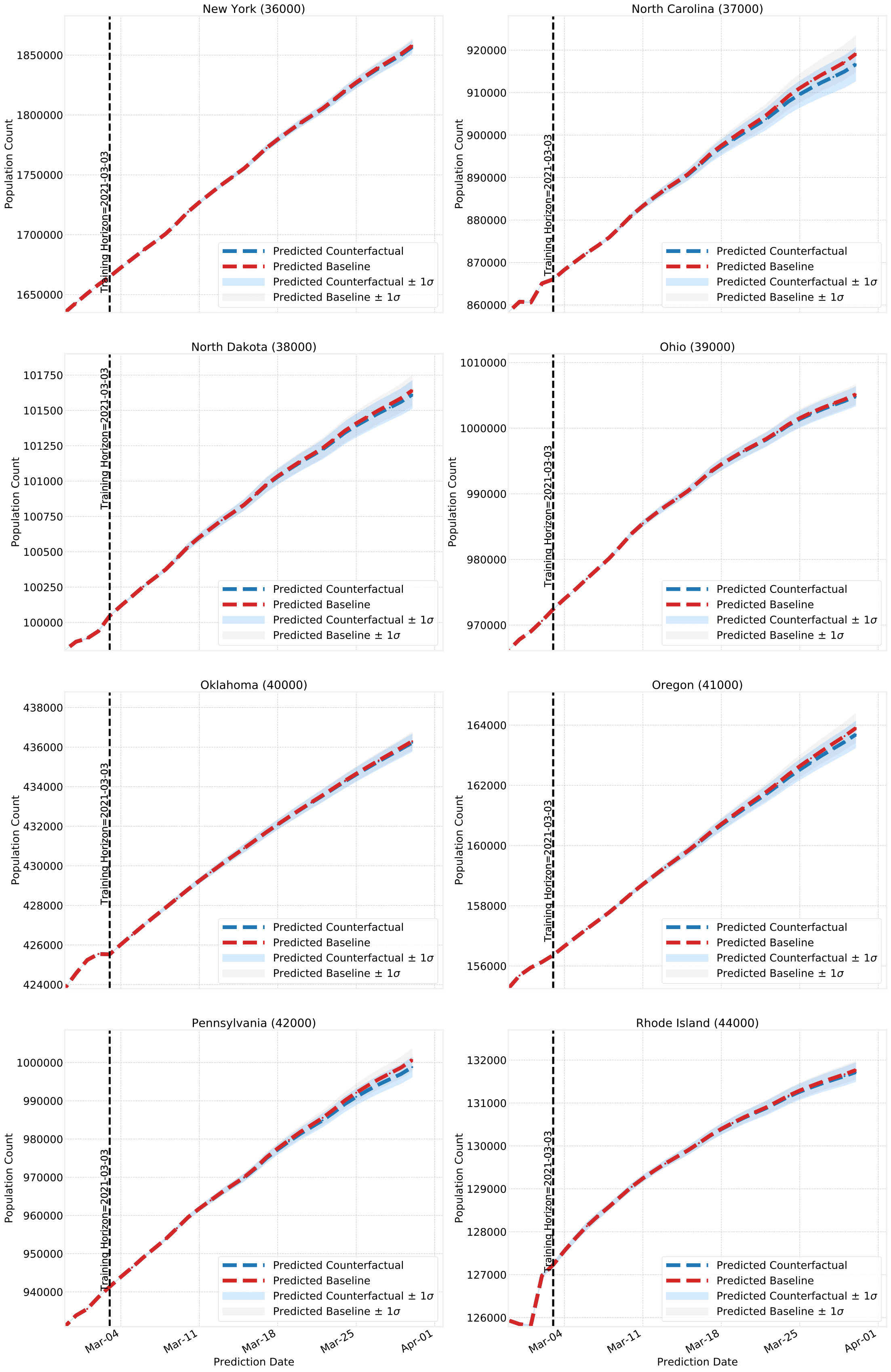

Supplementary Figure 26 | Baseline versus counterfactual prediction of confirmed cases for US states New York through Rhode Island. All the NPIs from Supplementary Table 31 are applied as a single counterfactual scenario. The shaded regions correspond to one standard deviation uncertainty bands.

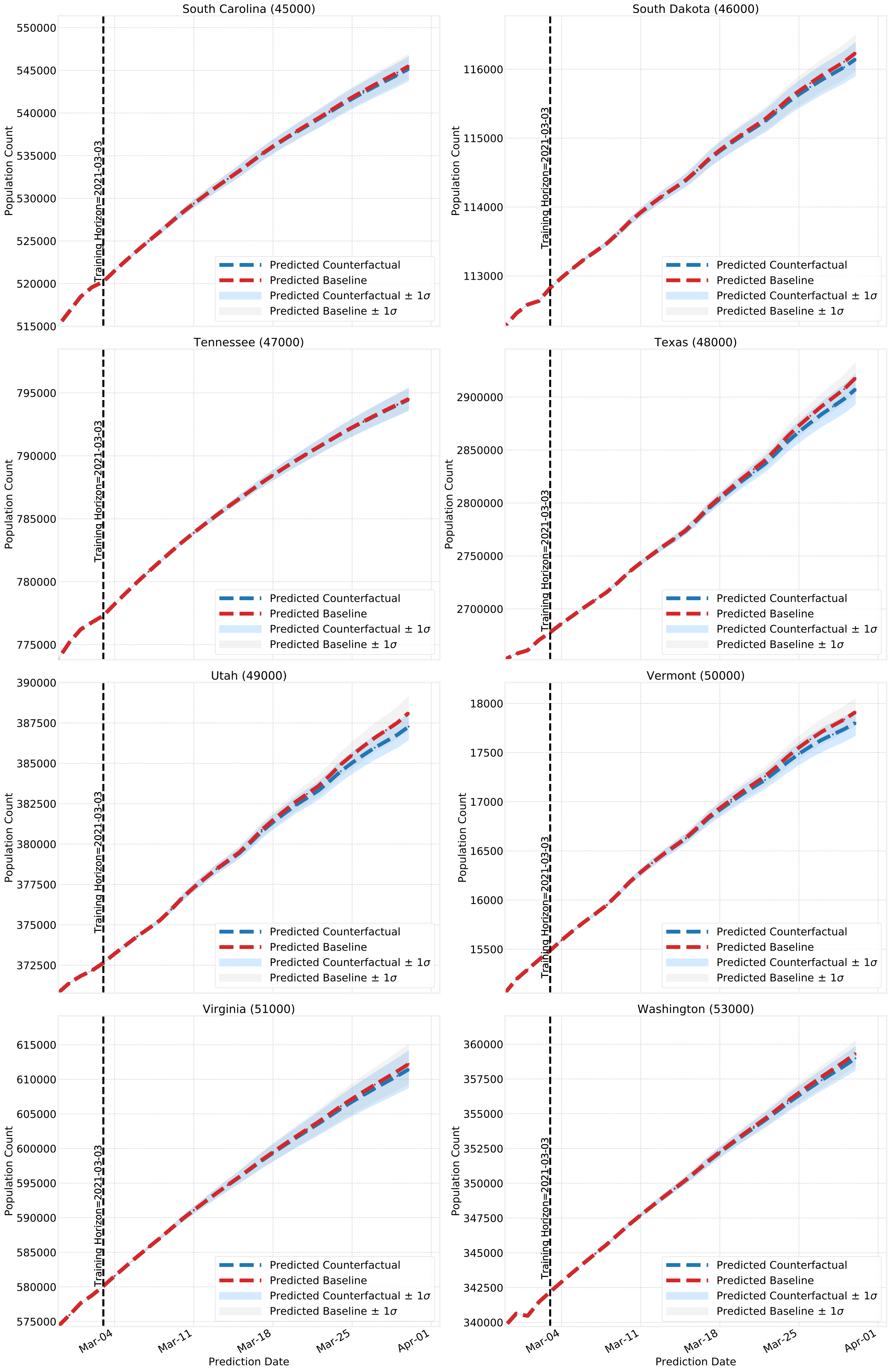

Supplementary Figure 27 | Baseline versus counterfactual prediction of confirmed cases for US states South Carolina through Washington. All the NPIs from Supplementary Table 31 are applied as a single counterfactual scenario. The shaded regions correspond to one standard deviation uncertainty bands.

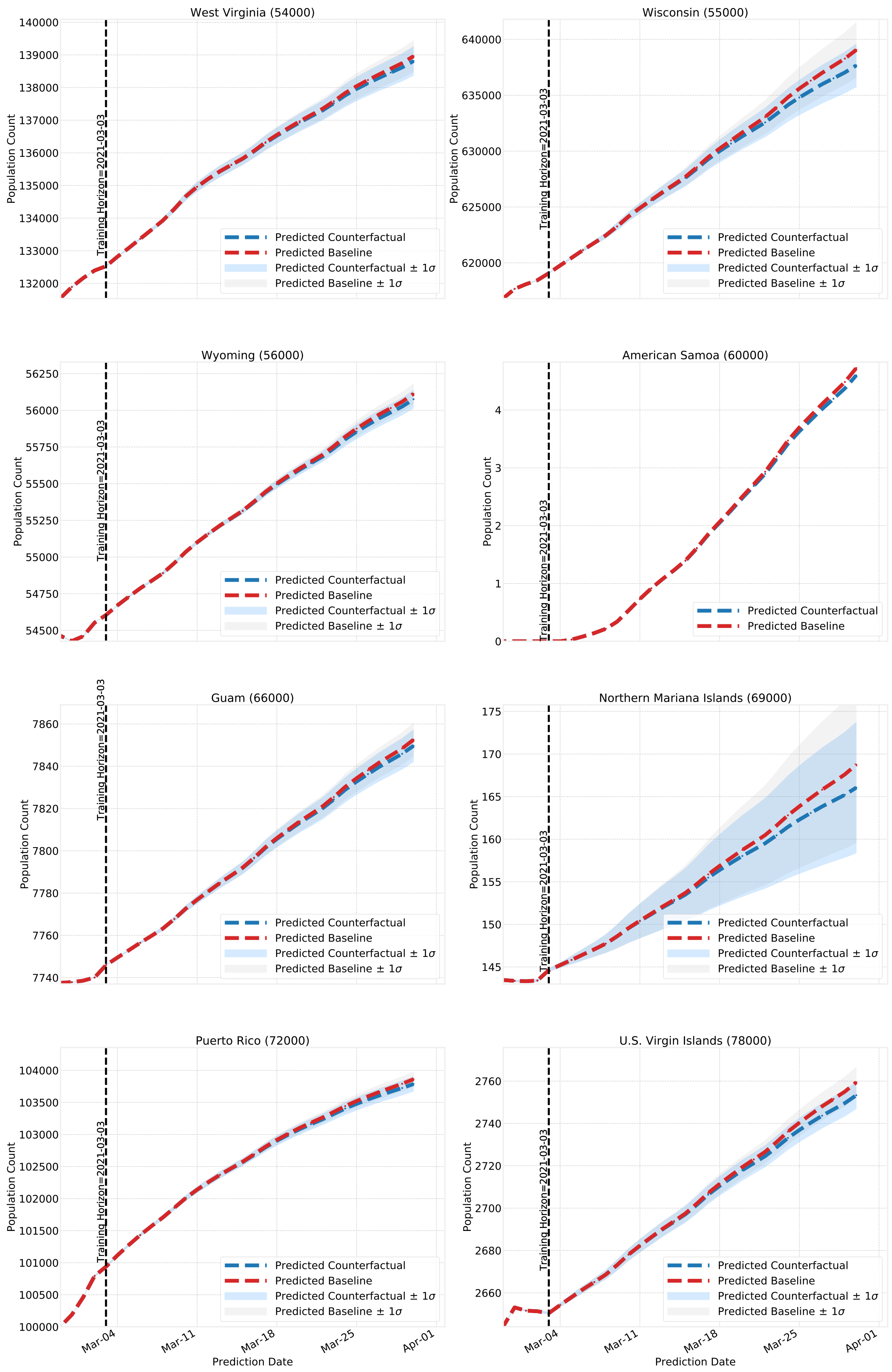

Supplementary Figure 28 | Baseline versus counterfactual prediction of confirmed cases for US states West Virginia through US Virgin Islands. All the NPIs from Supplementary Table 31 are applied as a single counterfactual scenario. The shaded regions correspond to one standard deviation uncertainty bands.

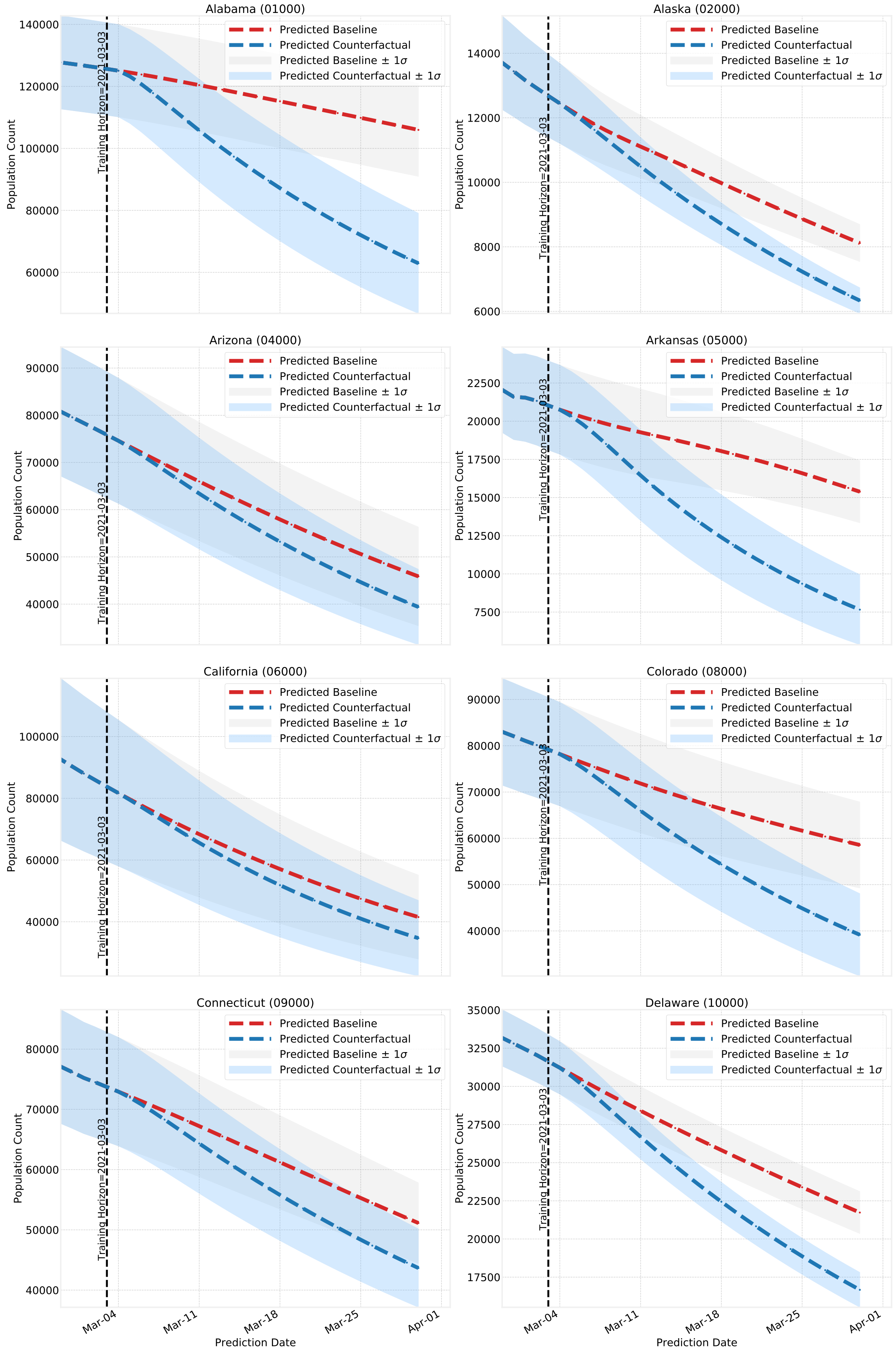

Supplementary Figure 29 | Baseline versus counterfactual prediction of exposed individuals for US states Alabama through Delaware. All the NPIs from Supplementary Table 31 are applied as a single counterfactual scenario. The shaded regions correspond to one standard deviation uncertainty bands.

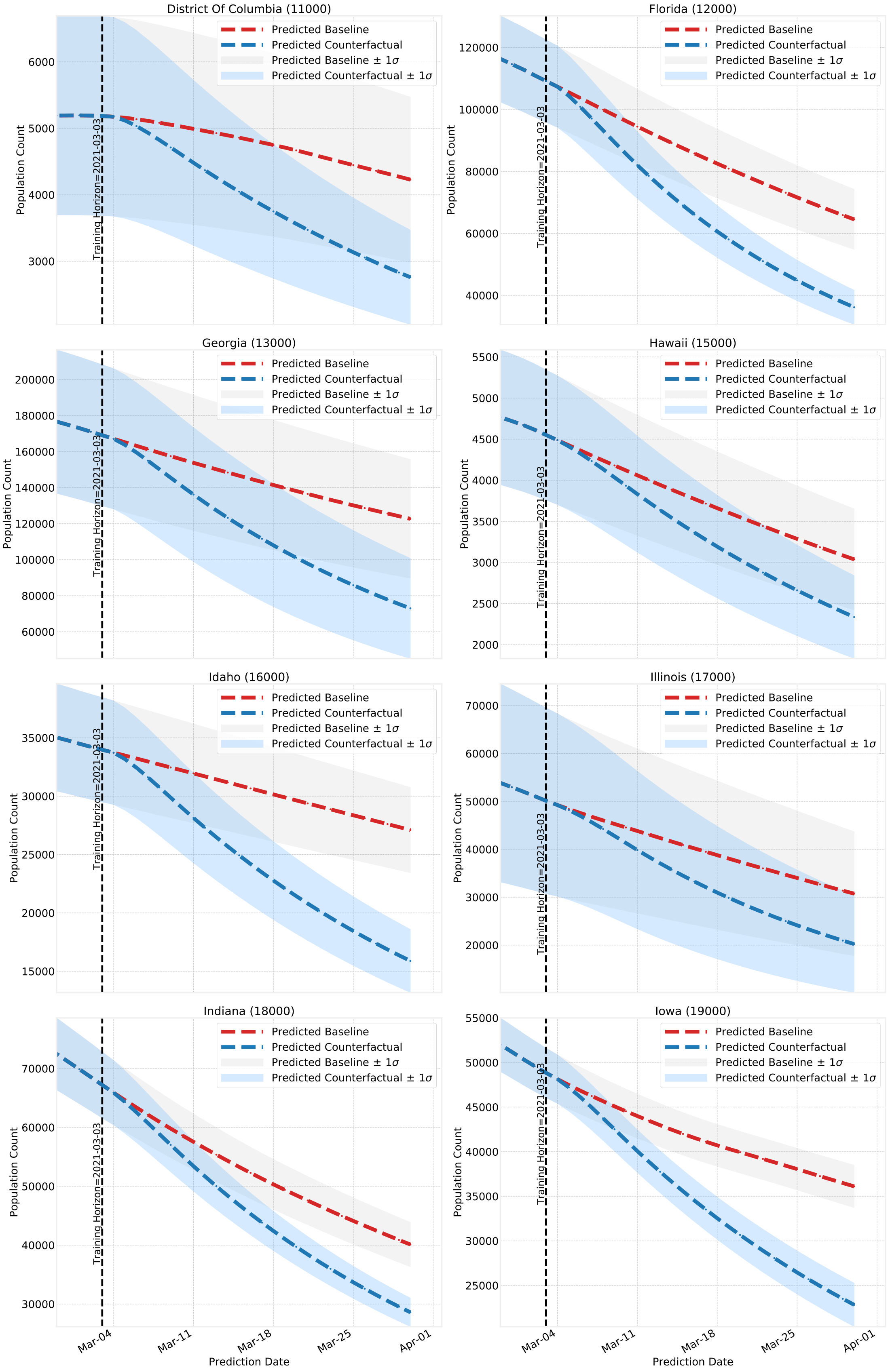

Supplementary Figure 30 | Baseline versus counterfactual prediction of exposed individuals for US states District of Columbia through Iowa. All the NPIs from Supplementary Table 31 are applied as a single counterfactual scenario. The shaded regions correspond to one standard deviation uncertainty bands.

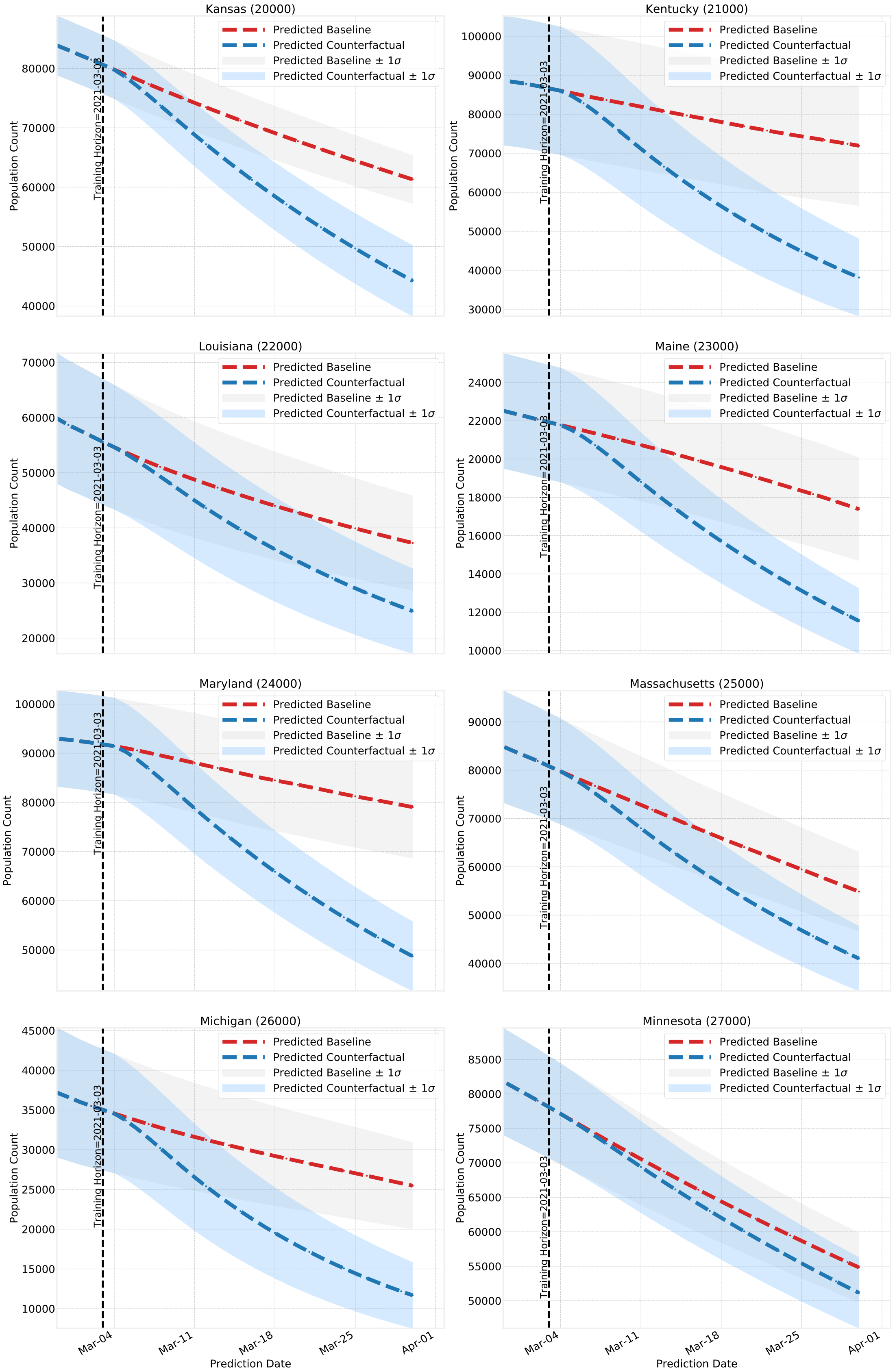

Supplementary Figure 31 | Baseline versus counterfactual prediction of exposed individuals for US states Kansas through Minnesota. All the NPIs from Supplementary Table 31 are applied as a single counterfactual scenario. The shaded regions correspond to one standard deviation uncertainty bands.

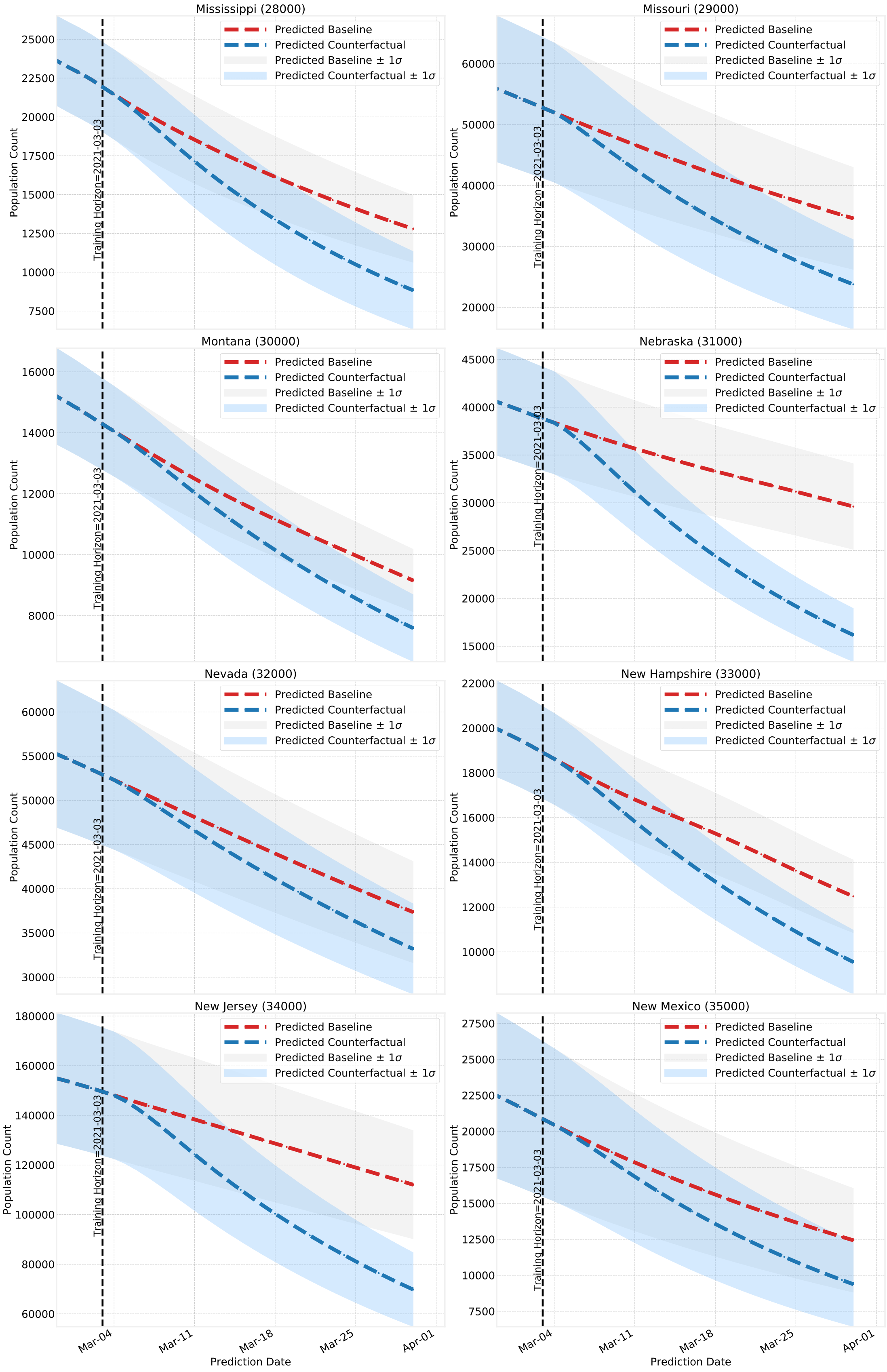

Supplementary Figure 32 | Baseline versus counterfactual prediction of exposed individuals for US states Mississippi through New Mexico. All the NPIs from Supplementary Table 31 are applied as a single counterfactual scenario. The shaded regions correspond to one standard deviation uncertainty bands.

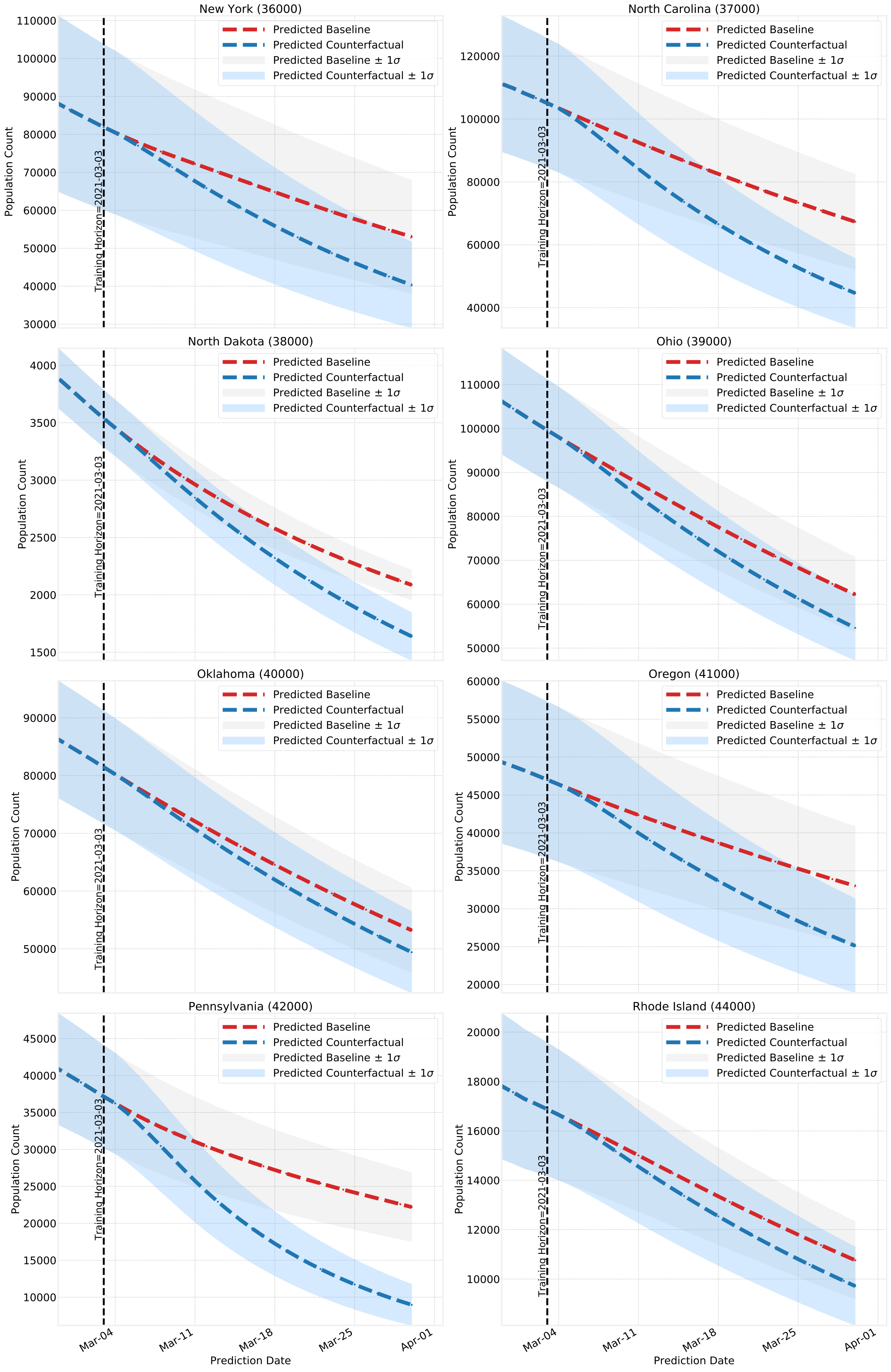

Supplementary Figure 33 | Baseline versus counterfactual prediction of exposed individuals for US states New York through Rhode Island. All the NPIs from Supplementary Table 31 are applied as a single counterfactual scenario. The shaded regions correspond to one standard deviation uncertainty bands.

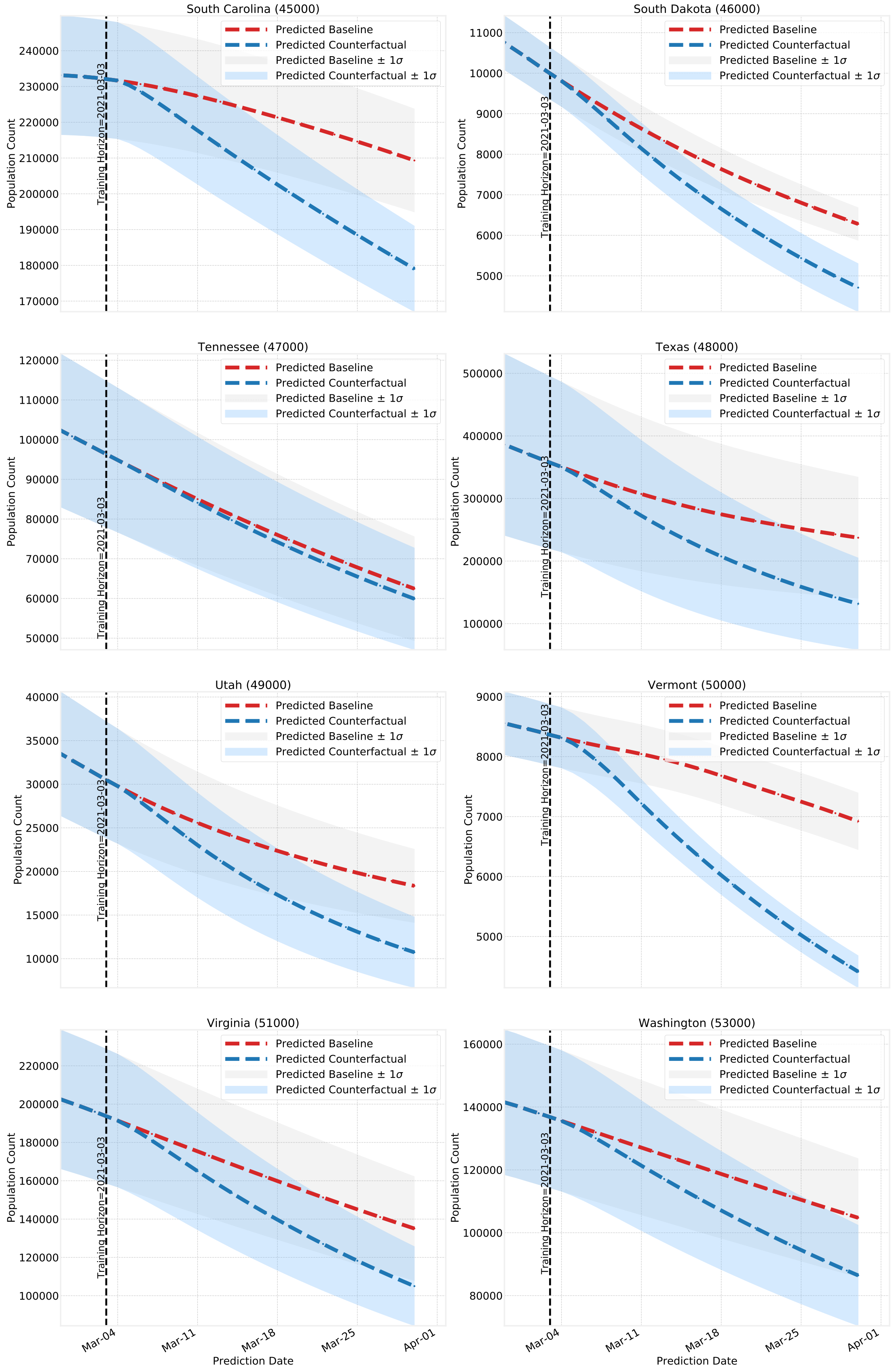

Supplementary Figure 34 | Baseline versus counterfactual prediction of exposed individuals for US states South Carolina through Washington. All the NPIs from Supplementary Table 31 are applied as a single counterfactual scenario. The shaded regions correspond to one standard deviation uncertainty bands.

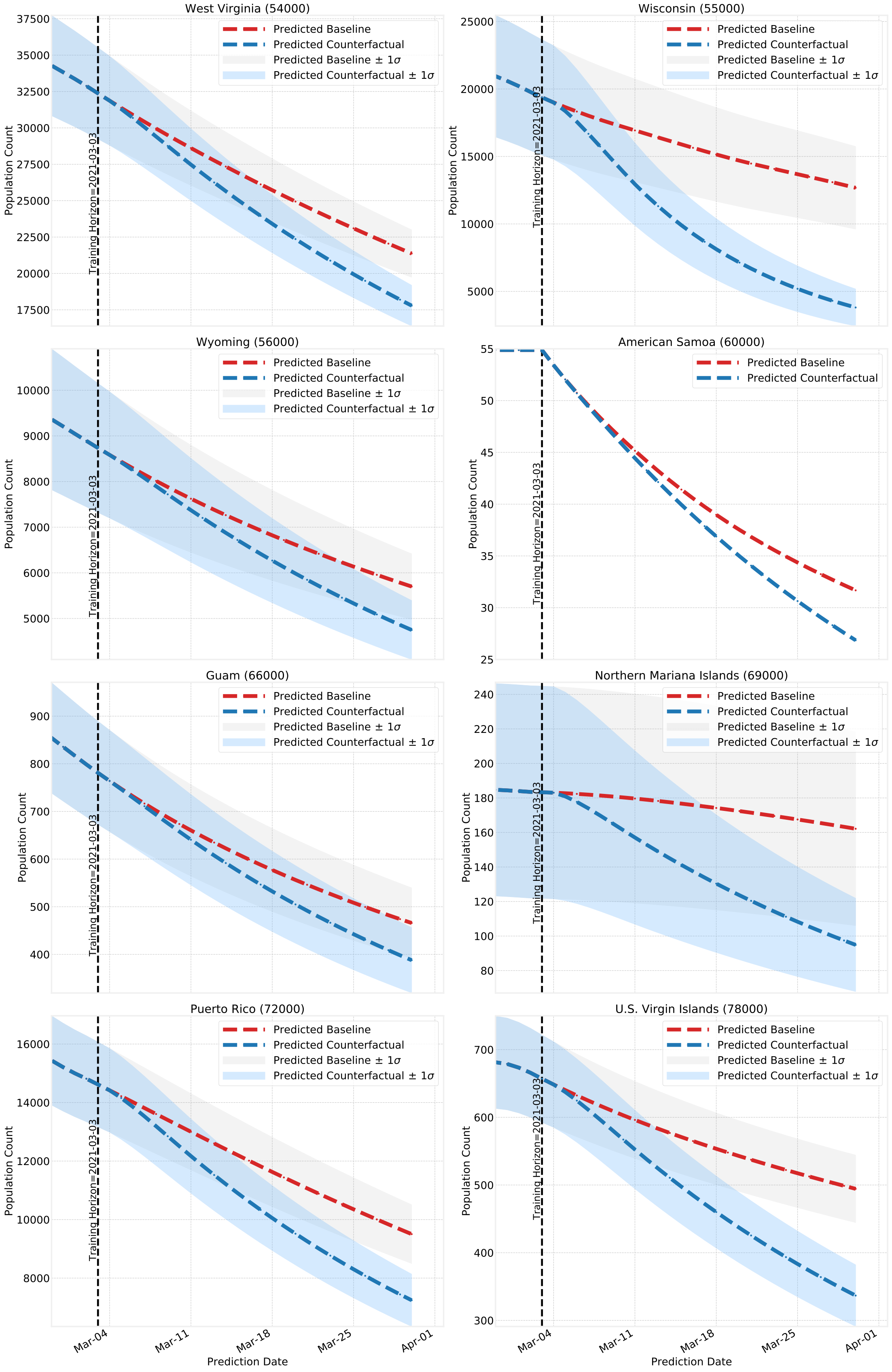

Supplementary Figure 35 | Baseline versus counterfactual prediction of exposed individuals for US states West Virginia through US Virgin Islands. All the NPIs from Supplementary Table 31 are applied as a single counterfactual scenario. The shaded regions correspond to one standard deviation uncertainty bands.

4 Supplementary Note 4: Additional explainability results

Figures 36 through 40 show the progression of the disease in Japanese prefectures, along with the decrease in the Force of Infection F_u and R_{eff} when NPIs are applied. The Force of Infection spikes on certain dates in a handful of prefectures: Akita (JP-05), Fukui (JP-18), Tottori (JP-31) and Shimane (JP-32). Those dates also correspond to a sharp spike in the predicted confirmed cases. This could indicate data restatements in those prefectures.

Figures 41 through 46 show the progression of the disease in US states and territories, along with the decrease in the Force of Infection F_u and R_{eff} when NPIs are applied.

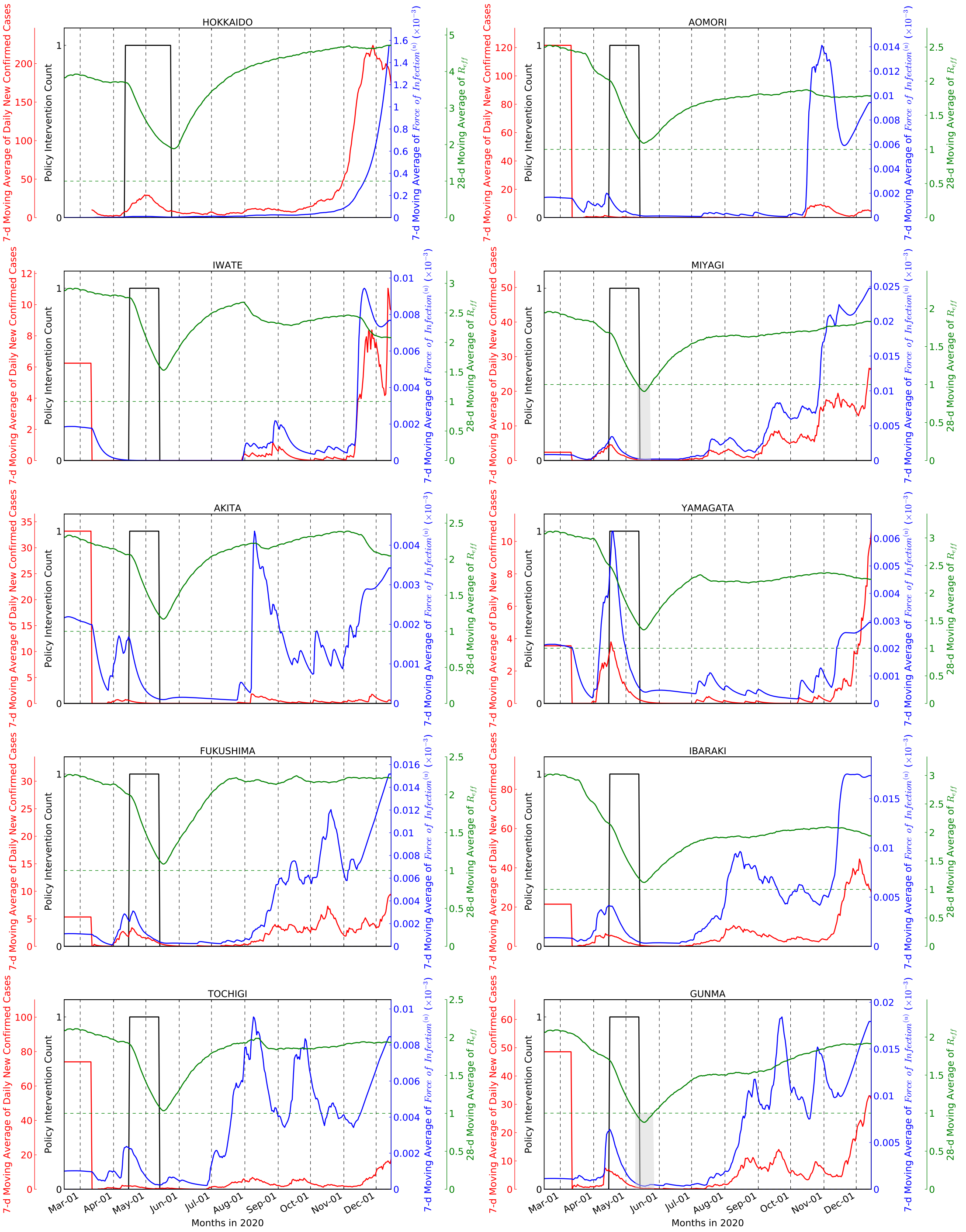

Supplementary Figure 36 | Progression of the disease in Japaneses Prefectures (JP-01 through JP-10)

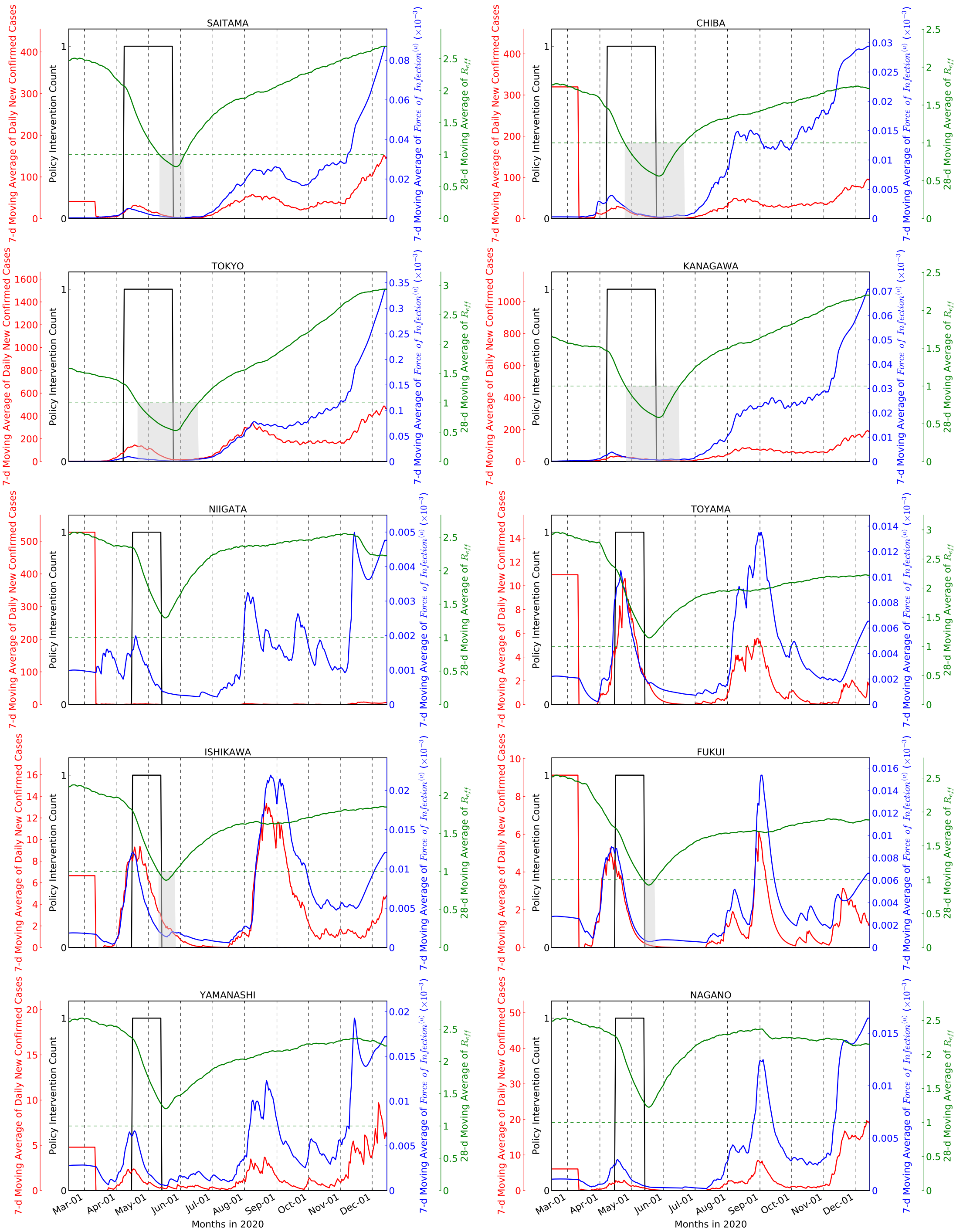

Supplementary Figure 37 | Progression of the disease in Japaneses Prefectures (JP-11 through JP-20)

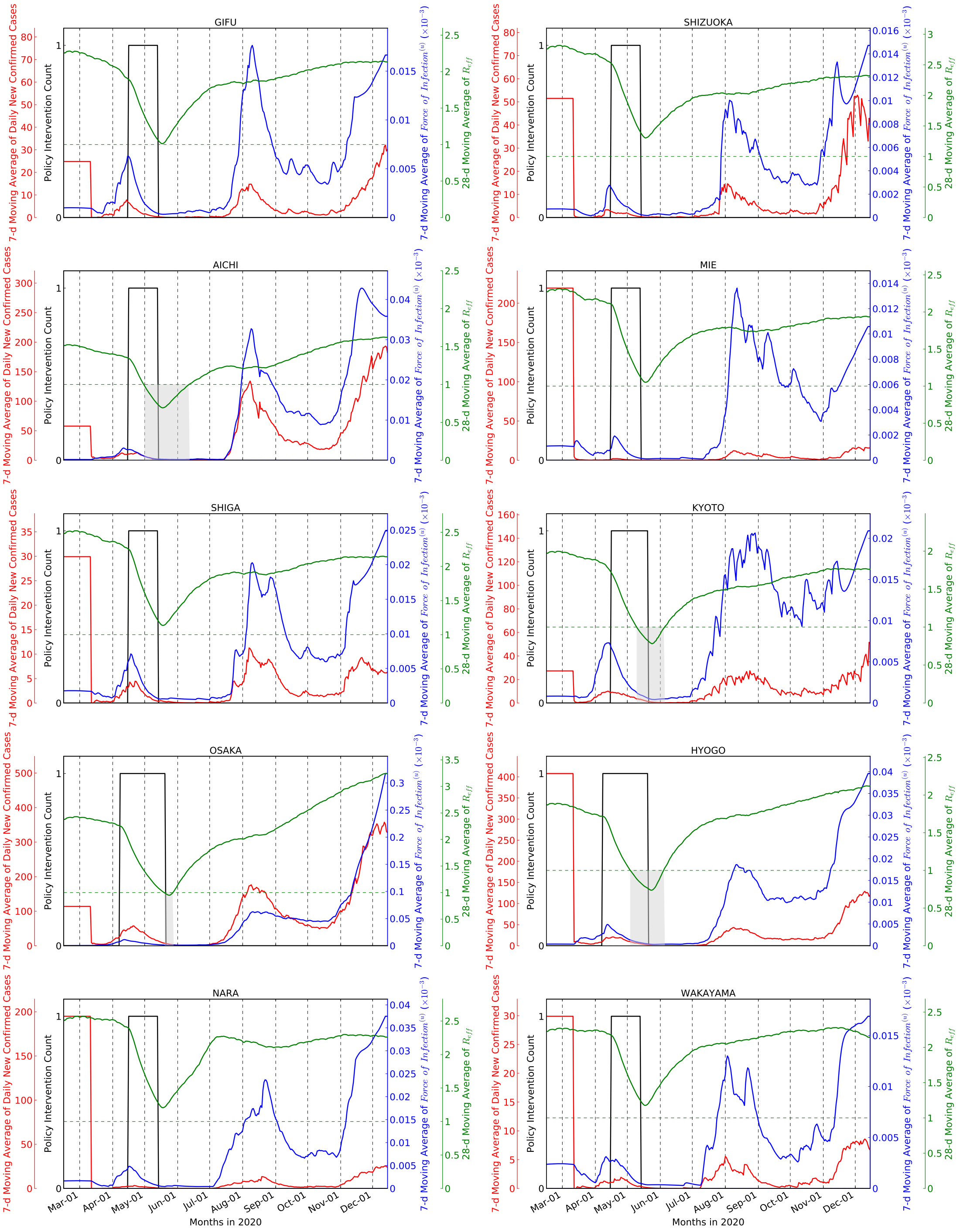

Supplementary Figure 38 | Progression of the disease in Japaneses Prefectures (JP-21 through JP-30)

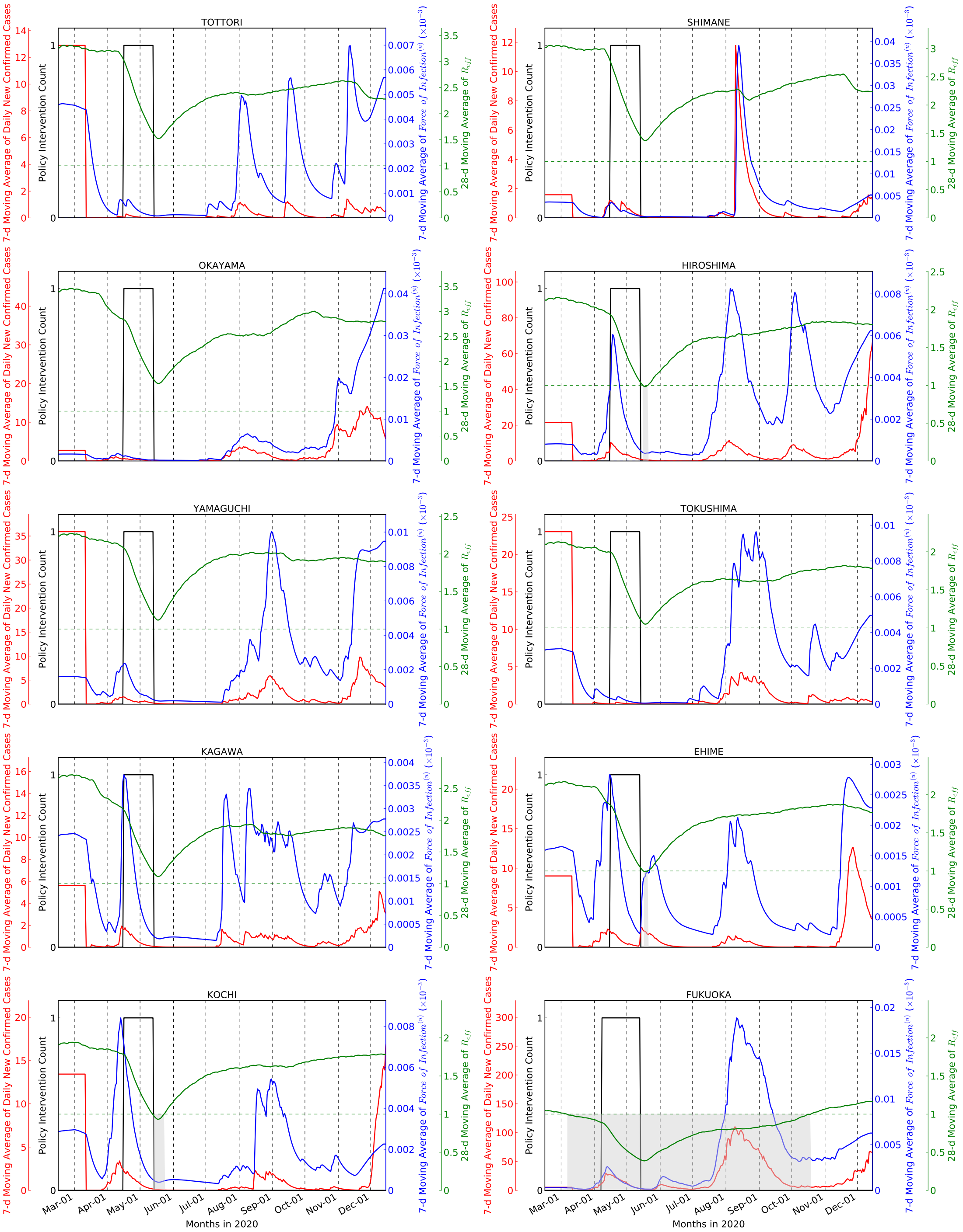

Supplementary Figure 39 | Progression of the disease in Japaneses Prefectures (JP-31 through JP-40)

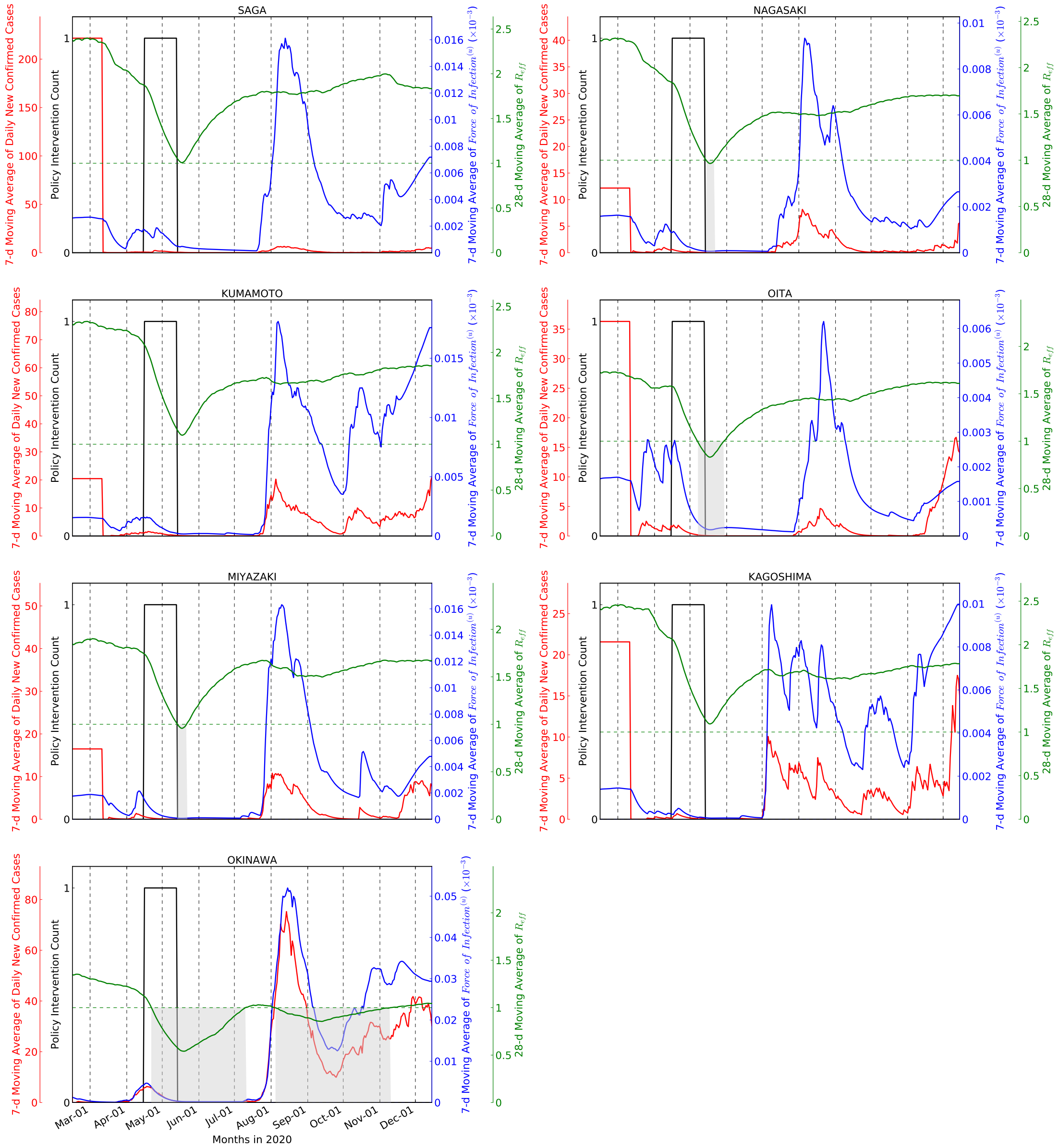

Supplementary Figure 40 | Progression of the disease in Japaneses Prefectures (JP-41 through JP-47)

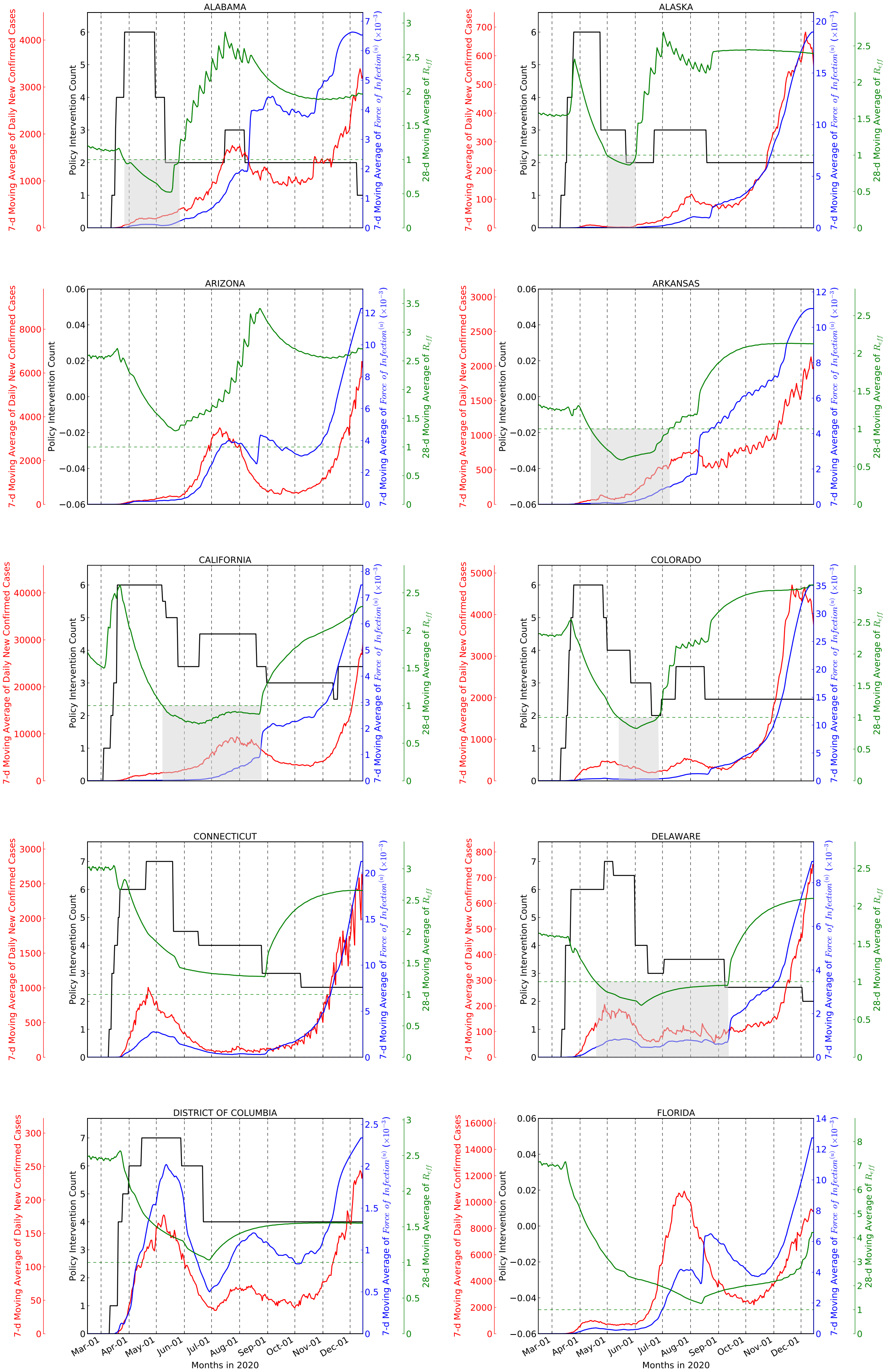

Supplementary Figure 41 | Progression of the disease in US States (Alabama through Florida)

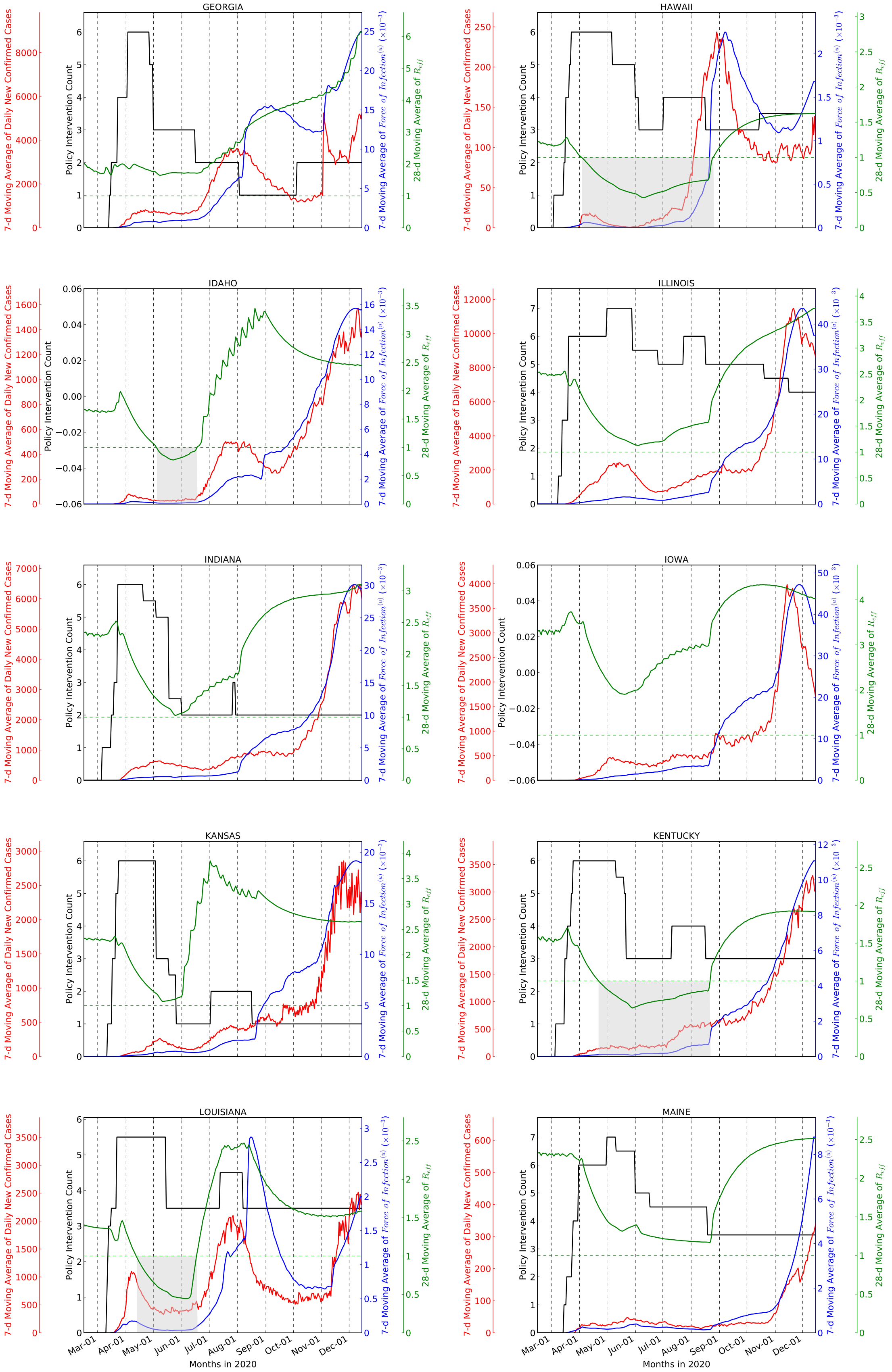

Supplementary Figure 42 | Progression of the disease in US States (Georgia through Maine)

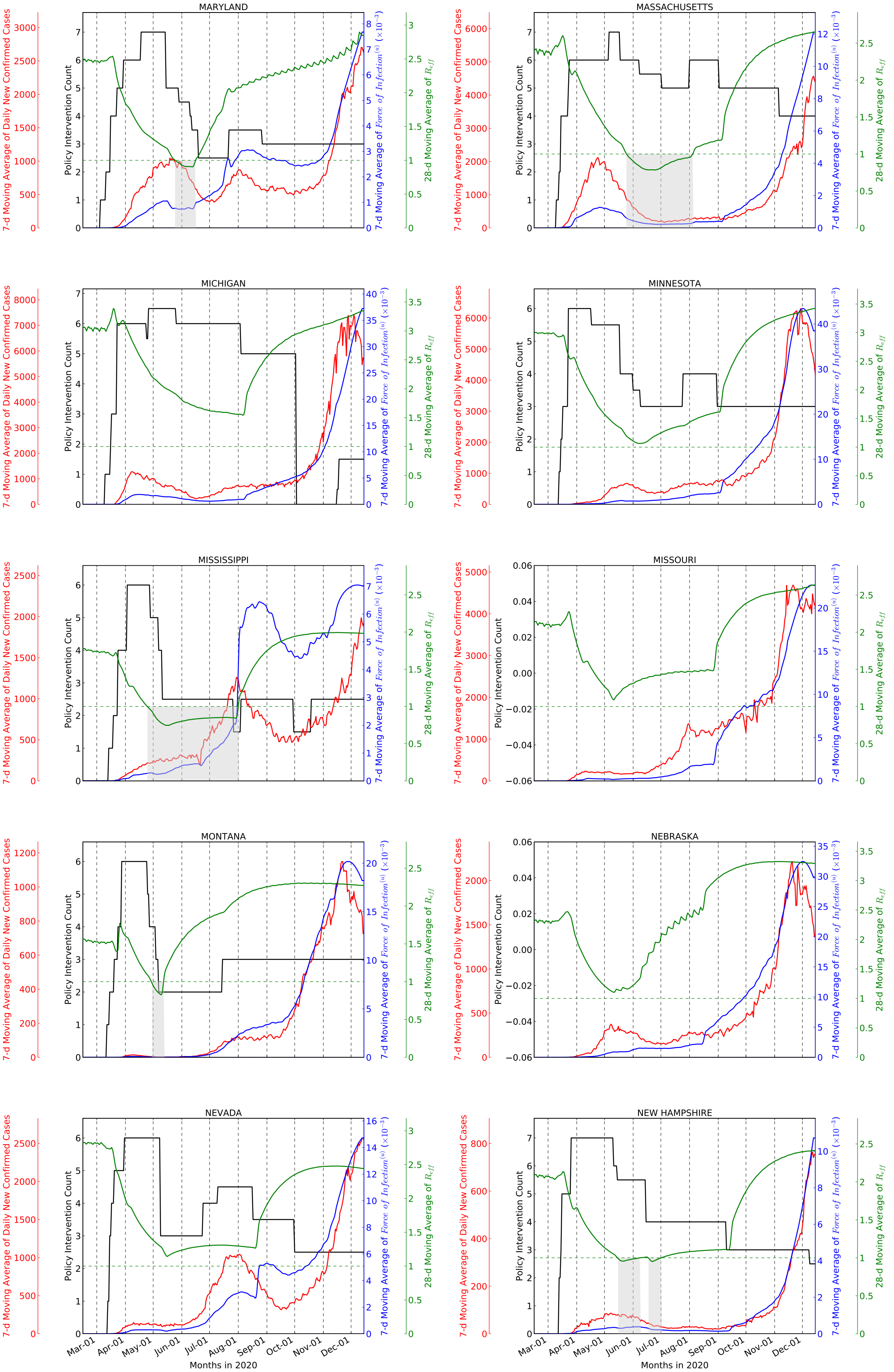

Supplementary Figure 43 | Progression of the disease in US States (Maryland through New Hamsphire)

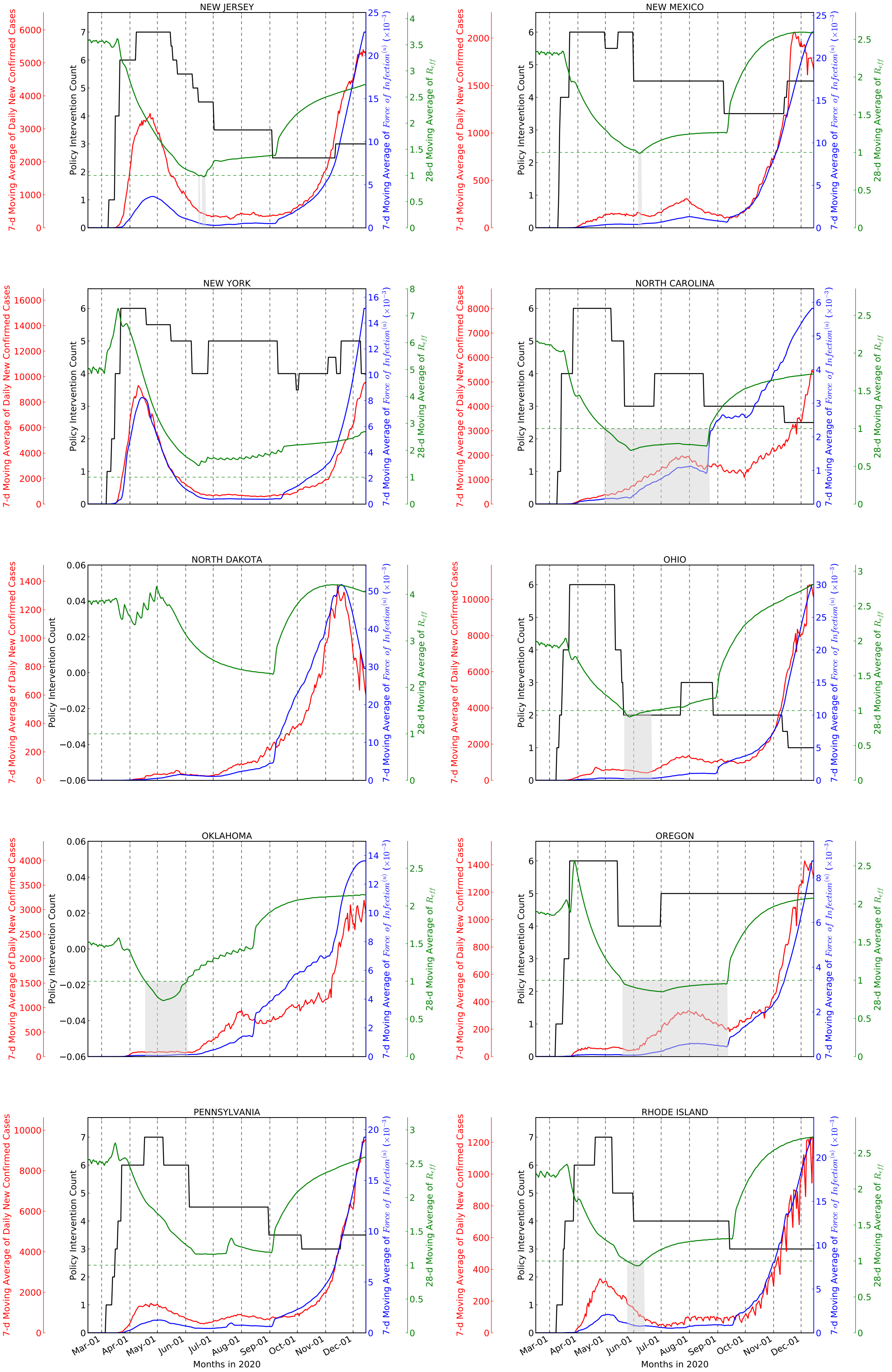

Supplementary Figure 44 | Progression of the disease in US States (New Jersey through Rhode Island)

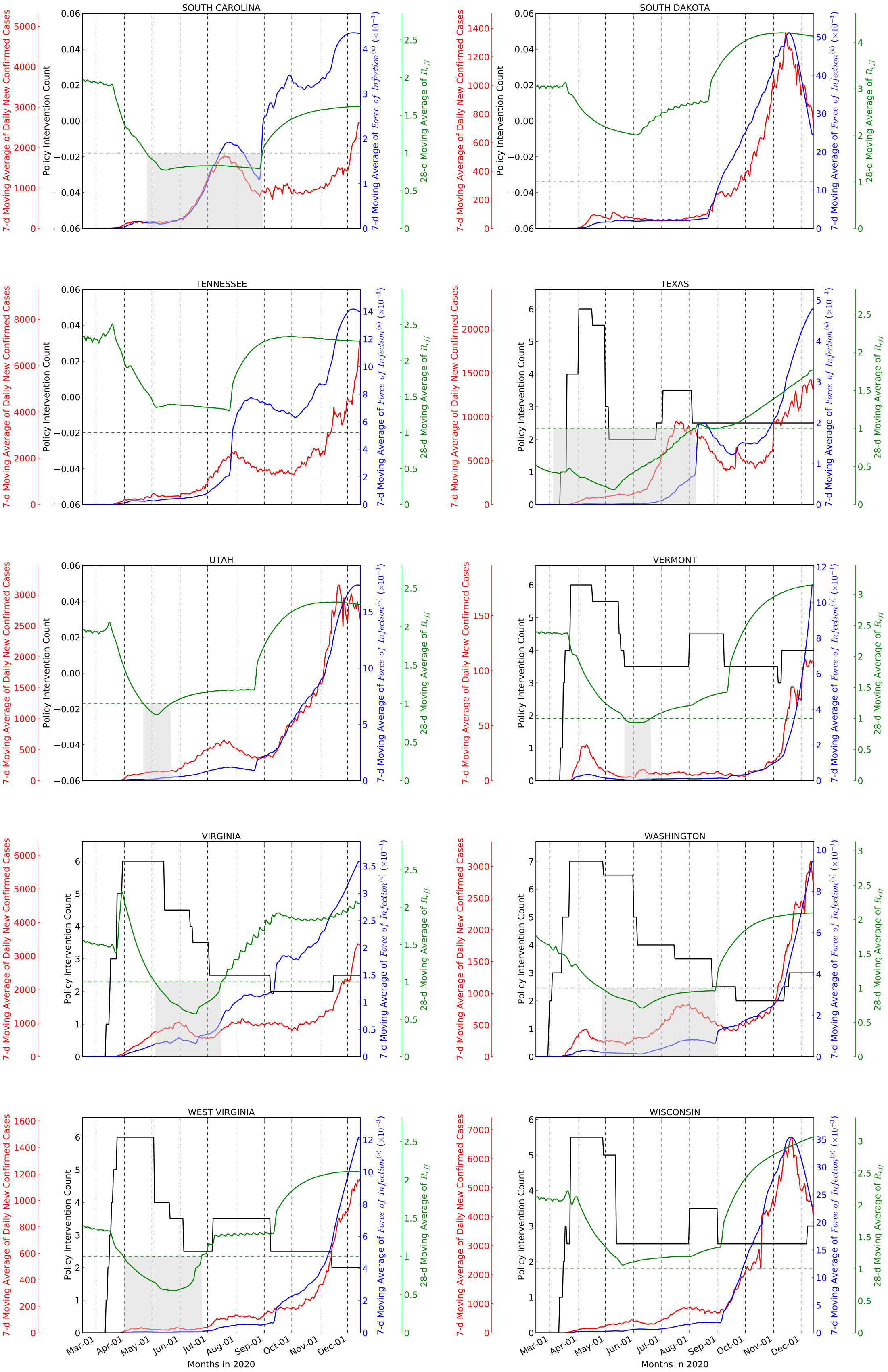

Supplementary Figure 45 | Progression of the disease in US States (South Carolina through Wisconsin)

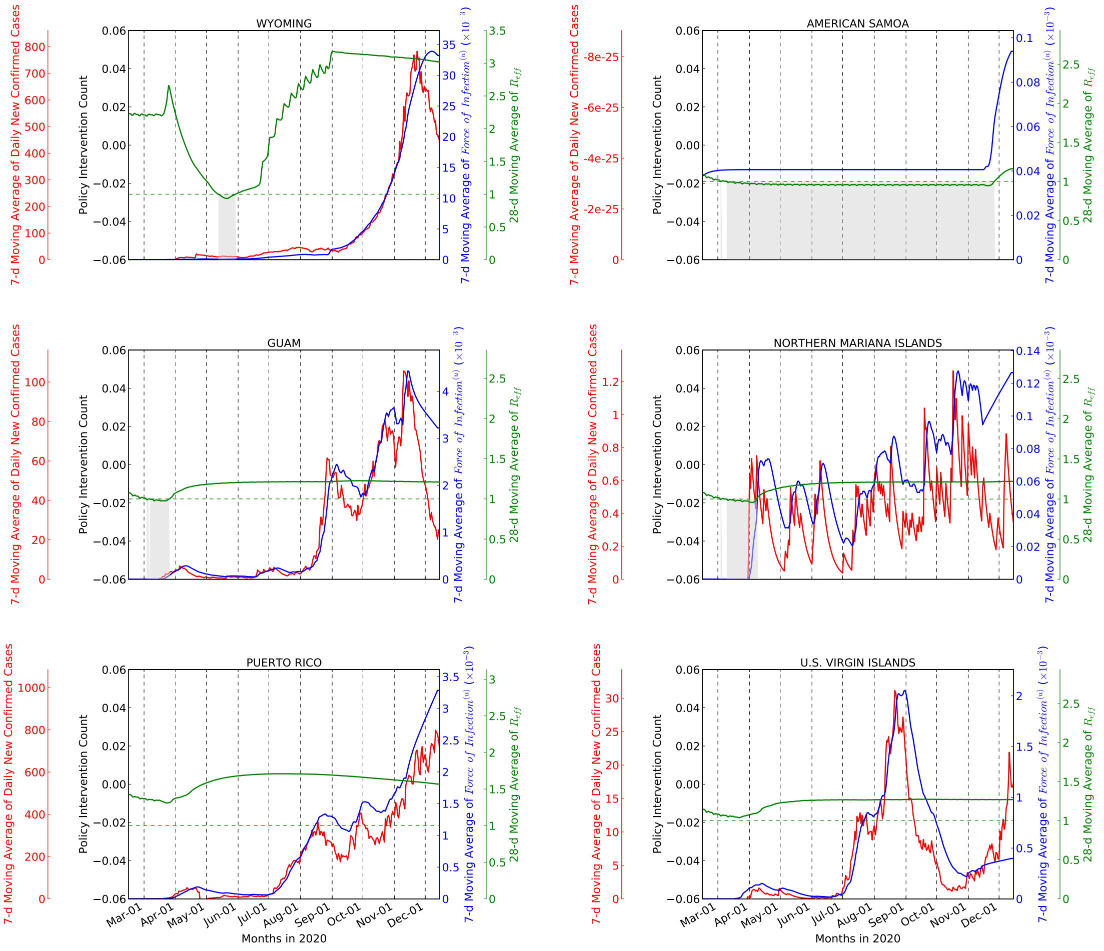

Supplementary Figure 46 | Progression of the disease in US States (Wyoming through U.S. Virgin Islands)

Feature Name	Median Rank
Number of Households	1
Per Capita Income	2
Mean Air Quality Index	3
Ratio of Population over 60	4
Population Density	5

For Average Documented Contact Rate

Feature Name	Median Rank
Ratio of Population over 60	1
Per Capita Income	2
Mean Air Quality Index	3
Population Density	4
Number of Households	5

For Average Undocumented Contact Rate

Feature Name	Median Rank
Average Rating	1
Per Capita Income	2
Mean Air Quality Index	3
Number of Critical Access Hospitals	4
Ratio of Population over 60	5
Households on Public Assistance	6
Population Density	7
Average Patient Experience	8
Emergency Services Supported	9
Number of ICU Beds	10
Number of Acute Care Hospitals	11
Non-Emergency Services Supported	12
Number of Households	13
Above Average Patient Experience	14
Below Average Patient Experience	15

For Hospitalization Rate

Feature Name	Median Rank
Number of Critical Access Hospitals	1
Average Rating	2
Ratio of Population over 60	3
Non-Emergency Services Supported	4
Below Average Patient Experience	5
Households on Public Assistance	6
Number of Households	7
Number of Acute Care Hospitals	8
Average Patient Experience	9
Above Average Patient Experience	10
Population Density	11
Mean Air Quality Index	12
Emergency Services Supported	13
Per Capita Income	14

For Reinfection Rate

Feature Name	Median Rank
Ratio of Population over 60	1
Number of Critical Access Hospitals	2
Per Capita Income	3
Below Average Patient Experience	4
Above Average Patient Experience	5
Non-Emergency Services Supported	6
Average Patient Experience	7
Mean Air Quality Index	8
Emergency Services Supported	9
Households on Public Assistance	10
Population Density	11
Average Rating	12
Number of Acute Care Hospitals	13
Number of Households	14
Number of ICU Beds	15

For ICU Admission Rate

Feature Name	Median Rank
Population Density	1
Number of Critical Access Hospitals	2
Households on Public Assistance	3
Per Capita Income	4
Emergency Services Supported	5
Average Patient Experience	6
Number of Acute Care Hospitals	7
Above Average Patient Experience	8
Average Rating	9
Number of ICU Beds	10
Number of Households	11
Non-Emergency Services Supported	12
Below Average Patient Experience	13
Ratio of Population over 60	14
Mean Air Quality Index	15

For Ventilator Rate

Feature Name	Median Rank
Above Average Patient Experience	1
Emergency Services Supported	2
Per Capita Income	3
Number of Acute Care Hospitals	4
Ratio of Population over 60	5
Number of Critical Access Hospitals	6
Number of Households	7
Below Average Patient Experience	8
Average Patient Experience	9
Non-Emergency Services Supported	10
Average Rating	11
Households on Public Assistance	12
Population Density	13

For Documented Recovery Rate

Feature Name	Median Rank
Below Average Patient Experience	1
Non-Emergency Services Supported	2
Number of Households	3
Number of Acute Care Hospitals	4
Average Patient Experience	5
Above Average Patient Experience	6
Emergency Services Supported	7
Population Density	8
Number of Critical Access Hospitals	9
Average Rating	10
Per Capita Income	11
Ratio of Population over 60	12
Households on Public Assistance	13

For Undocumented Recovery Rate

Feature Name	Median Rank
Non-Emergency Services Supported	1
Average Patient Experience	2
Above Average Patient Experience	3
Number of Acute Care Hospitals	4
Emergency Services Supported	5
Ratio of Population over 60	6
Number of Households	7
Population Density	8
Below Average Patient Experience	9
Number of Critical Access Hospitals	10
Average Rating	11
Households on Public Assistance	12
Per Capita Income	13

For Recovery Rate from Hospitals

Feature Name	Median Rank
Population Density	1
Households on Public Assistance	2
Average Patient Experience	3
Average Rating	4
Non-Emergency Services Supported	5
Number of Acute Care Hospitals	6
Per Capita Income	7
Ratio of Population over 60	8
Emergency Services Supported	9
Number of Critical Access Hospitals	10
Number of Households	11
Below Average Patient Experience	12
Above Average Patient Experience	13

For Recovery Rate from ICUs

Supplementary Figure 47 | Ranking of static features for encoders in the US Model of January 20, 2021 (Part 1).

Feature Name	Median Rank
Population Density	1
Number of Critical Access Hospitals	2
Households on Public Assistance	3
Per Capita Income	4
Average Rating	5
Average Patient Experience	6
Ratio of Population over 60	7
Non-Emergency Services Supported	8
Emergency Services Supported	9
Number of Acute Care Hospitals	10
Number of Households	11
Above Average Patient Experience	12
Below Average Patient Experience	13

For Recovery Rate from Ventilators

Feature Name	Median Rank
Population Density	1
Per Capita Income	2
Average Rating	3
Ratio of Population over 60	4
Households on Public Assistance	5
Mean Air Quality Index	6
Average Patient Experience	7
Number of Acute Care Hospitals	8
Non-Emergency Services Supported	9
Number of Households	10
Below Average Patient Experience	11
Above Average Patient Experience	12
Emergency Services Supported	13
Number of Critical Access Hospitals	14
Number of ICU Beds	15

For Documented Death Rate

Feature Name	Median Rank
Non-Emergency Services Supported	1
Number of ICU Beds	2
Number of Acute Care Hospitals	3
Average Patient Experience	4
Emergency Services Supported	5
Number of Households	6
Below Average Patient Experience	7
Number of Critical Access Hospitals	8
Ratio of Population over 60	9
Above Average Patient Experience	10
Mean Air Quality Index	11
Households on Public Assistance	12
Population Density	13
Per Capita Income	14
Average Rating	15

For Death Rate from Hospitals

Feature Name	Median Rank
Number of Critical Access Hospitals	1
Below Average Patient Experience	2
Number of ICU Beds	3
Mean Air Quality Index	4
Emergency Services Supported	5
Number of Households	6
Non-Emergency Services Supported	7
Number of Acute Care Hospitals	8
Population Density	9
Average Patient Experience	10
Ratio of Population over 60	11
Households on Public Assistance	12
Above Average Patient Experience	13
Per Capita Income	14
Average Rating	15

For Death Rate from ICUs

Feature Name	Median Rank
Population Density	1
Number of Critical Access Hospitals	2
Average Rating	3
Per Capita Income	4
Above Average Patient Experience	5
Below Average Patient Experience	6
Households on Public Assistance	7
Mean Air Quality Index	8
Average Patient Experience	9
Ratio of Population over 60	10
Emergency Services Supported	11
Number of ICU Beds	12
Number of Households	13
Number of Acute Care Hospitals	14
Non-Emergency Services Supported	15

For Death Rate from Ventilators

Feature Name	Median Rank
Ratio of Population over 60	1
Population Density	2
Per Capita Income	3
Number of Households	4
Average Rating	5

For Diagnosis Rate

Supplementary Figure 48 | Ranking of static features for encoders in the US Model of January 20, 2021 (Part 2).

Feature Name	Median Rank
NPI Schools	1
Deaths	2
Cases/Total Tests	3
Mobility Index	4
Confirmed Cases	5
Snowfall (mm)	6
Negative Antibody Test Ratio	7
NPI Non-Essential Business	8
NPI Bar/Restaurants	9
Positive Antibody Test Ratio	10
Cases Mean to Sum Ratio	11
Rainfall (mm)	12
Deaths Mean to Sum Ratio	13
Mobility Samples	14
NPI Gatherings	15
NPI Mask	16
Average Temperature (C)	17
NPI Movement	18
NPI Emergency	19

For Average Documented Contact Rate

Feature Name	Median Rank
NPI Schools	1
NPI Bar/Restaurants	2
Snowfall (mm)	3
Mobility Index	4
Cases/Total Tests	5
NPI Non-Essential Business	6
Mobility Samples	7
Average Temperature (C)	8
Deaths	9
Cases Mean to Sum Ratio	10
Deaths Mean to Sum Ratio	11
Confirmed Cases	12
NPI Movement	13
Positive Antibody Test Ratio	14
Negative Antibody Test Ratio	15
NPI Gatherings	16
NPI Mask	17
NPI Emergency	18
Rainfall (mm)	19

For Average Undocumented Contact Rate

Feature Name	Median Rank
Symptoms Search for Cough	1
Cases/Total Tests	2
Symptoms Search for Anosmia	3
Symptoms Search for Fever	4
Average Temperature (C)	5
Total Per Capita Tests	6
Snowfall (mm)	7
Positive Antibody Test Ratio	8
Day of Week	10
Symptoms Search for Chest Pain	10
Deaths Mean to Sum Ratio	11
Symptoms Search for Infection	11
Rainfall (mm)	12
Deaths	13
Confirmed Cases	14
Symptoms Search for Chills	16
Negative Antibody Test Ratio	17
Symptoms Search for Shortness of Breath	18
Cases Mean to Sum Ratio	19

For Hospitalization Rate

Feature Name	Median Rank
Snowfall (mm)	1
Symptoms Search for Anosmia	2
Symptoms Search for Chest Pain	3
Rainfall (mm)	4
Symptoms Search for Fever	6
Symptoms Search for Chills	7
Symptoms Search for Infection	8
Symptoms Search for Shortness of Breath	8
Total Per Capita Tests	9
Cases/Total Tests	10
Average Temperature (C)	11
Cases Mean to Sum Ratio	11
Symptoms Search for Cough	11
Deaths	13
Day of Week	15
Positive Antibody Test Ratio	15
Confirmed Cases	17
Deaths Mean to Sum Ratio	18
Negative Antibody Test Ratio	19

For Diagnosis Rate

Feature Name	Median Rank
Deaths	1
Confirmed Cases	2
Deaths Mean to Sum Ratio	3
Day of Week	4
Cases Mean to Sum Ratio	5

For ICU Admission Rate

Feature Name	Median Rank
Deaths Mean to Sum Ratio	1
Confirmed Cases	2
Cases Mean to Sum Ratio	3
Day of Week	4
Deaths	5

For Ventilator Rate

Feature Name	Median Rank
Rainfall (mm)	1
Deaths	2
Average Temperature (C)	3
Snowfall (mm)	4
Confirmed Cases	5
Cases Mean to Sum Ratio	6
Positive Antibody Test Ratio	7
Deaths Mean to Sum Ratio	8
Day of Week	9
Negative Antibody Test Ratio	10

For Documented Recovery Rate

Feature Name	Median Rank
Rainfall (mm)	1
Snowfall (mm)	2
Confirmed Cases	3
Average Temperature (C)	4
Deaths Mean to Sum Ratio	5
Positive Antibody Test Ratio	6
Deaths	7
Cases Mean to Sum Ratio	8
Negative Antibody Test Ratio	9

For Undocumented Recovery Rate

Feature Name	Median Rank
Day of Week	1
Deaths	2
Deaths Mean to Sum Ratio	3
Cases Mean to Sum Ratio	4
Confirmed Cases	5

For Recovery Rate from Hospitals

Feature Name	Median Rank
Confirmed Cases	1
Deaths Mean to Sum Ratio	2
Cases Mean to Sum Ratio	3
Deaths	4
Day of Week	5

For Recovery Rate from ICUs

Supplementary Figure 49 | Ranking of time-varying features for encoders for Encoders in the US Model of January 20, 2021 (Part 1).

Feature Name	Median Rank
Confirmed Cases	1
Deaths	2
Deaths Mean to Sum Ratio	3
Cases Mean to Sum Ratio	4
Day of Week	5

For Recovery Rate from Ventilators

Feature Name	Median Rank
Cases Mean to Sum Ratio	1
Deaths Mean to Sum Ratio	2
Day of Week	3
Deaths	4
Confirmed Cases	5

Feature Ranks for Documented Death Rate

Feature Name	Median Rank
Day of Week	1
Confirmed Cases	2
Deaths	3
Deaths Mean to Sum Ratio	4
Cases Mean to Sum Ratio	5

For Death Rate from Hospitals

Feature Name	Median Rank
Day of Week	1
Deaths	2
Confirmed Cases	3
Deaths Mean to Sum Ratio	4
Cases Mean to Sum Ratio	5

For Death Rate from ICUs

Feature Name	Median Rank
Day of Week	1
Confirmed Cases	2
Deaths Mean to Sum Ratio	3
Deaths	4
Cases Mean to Sum Ratio	5

For Death Rate from Ventilators

Supplementary Figure 50 | Ranking of time-varying features for encoders for Encoders in the US Model of January 20, 2021 (Part 2).

Feature Name	Median Rank
% Population that drink alcohol	1
BMI of Females (lower confidence interval)	2
People with H1N1 in 2010	3
Population over 75	4
Population Density	5
Average BMI of males	6
Per Capita Income	7
Population over 64	8
Population aged 0-14	9
Population aged 15-64	10
% Female Smokers	11
% Male Smokers	11

For Average Documented Contact Rate

Feature Name	Median Rank
People with H1N1 in 2010	1
BMI of Females (lower confidence interval)	2
% Population that drink alcohol	3
Average BMI of males	4
Population Density	5
Population over 64	6
Population aged 15-64	7
Per Capita Income	8
Population aged 0-14	9
Population over 75	10
% Female Smokers	11
% Male Smokers	11

For Average Undocumented Contact Rate

Feature Name	Median Rank
Population over 75	1
% Population that drink alcohol	2
Population Density	3
Population aged 0-14	4
Population over 64	5
Population aged 15-64	6
Per Capita Income	7
Average BMI of males	8
BMI of Females (lower confidence interval)	9
People with H1N1 in 2010	10
% Female Smokers	11
% Male Smokers	11

For Reinfection Rate

Feature Name	Median Rank
Number of clinic beds	1
Number of hospital beds	2
Number of new ICU beds	3
Number of doctors	4
Number of doctors/100k population	5
Number of clinic beds/100k population	6
Number of hospital beds/100k population	7

For Diagnosis Rate

Feature Name	Median Rank
Number of hospital beds	1
Population over 64	2
Population aged 15-64	3
Number of doctors	4
Population aged 0-14	5
Number of new ICU beds	6
Per Capita Income	7
Average BMI of males	8
Number of doctors/100k population	9
% Population that drink alcohol	10
Population over 75	11
Number of clinic beds	12
BMI of Females (lower confidence interval)	13
Number of clinic beds/100k population	14
People with H1N1 in 2010	15
Population Density	16
Number of hospital beds/100k population	17
% Female Smokers	18
% Male Smokers	18

For Documented Recovery Rate

Feature Name	Median Rank
Average BMI of males	1
People with H1N1 in 2010	2
Number of new ICU beds	3
Number of clinic beds/100k population	4
Number of doctors/100k population	5
BMI of Females (lower confidence interval)	6
Number of hospital beds/100k population	7
Population aged 0-14	8
Per Capita Income	9
Population over 64	10
Population aged 15-64	11
Number of doctors	12
Population Density	13
% Population that drink alcohol	14
Population over 75	15
Number of hospital beds	16
Number of clinic beds	17
% Female Smokers	18
% Male Smokers	18

For Undocumented Recovery Rate

Feature Name	Median Rank
Average BMI of males	1
People with H1N1 in 2010	2
Number of hospital beds/100k population	3
BMI of Females (lower confidence interval)	4
Number of doctors/100k population	5
Number of new ICU beds	6
Per Capita Income	7
Population aged 15-64	8
Population over 64	9
Number of clinic beds/100k population	10
Number of clinic beds	11
Population aged 0-14	12
Number of hospital beds	13
Number of doctors	14
Population over 75	15
% Population that drink alcohol	16
Population Density	17
% Female Smokers	18
% Male Smokers	18

For Recovery Rate from Hospitals

Feature Name	Median Rank
Population over 75	1
Number of clinic beds/100k population	2
Number of new ICU beds	3
% Population that drink alcohol	4
People with H1N1 in 2010	5
Number of doctors/100k population	6
BMI of Females (lower confidence interval)	7
Number of clinic beds	8
Population Density	9
Per Capita Income	10
Average BMI of males	11
Number of hospital beds	12
Population aged 15-64	13
Number of hospital beds/100k population	14
Number of doctors	15
Population aged 0-14	16
Population over 64	17
% Female Smokers	18
% Male Smokers	18

For Documented Death Rate

Feature Name	Median Rank
Number of clinic beds/100k population	1
Population Density	2
Population over 75	3
Number of new ICU beds	4
Number of doctors	5
Population aged 0-14	6
Number of doctors/100k population	7
Per Capita Income	8
% Population that drink alcohol	9
Population aged 15-64	10
Population over 64	11
Number of hospital beds/100k population	12
Number of hospital beds	13
Average BMI of males	14
BMI of Females (lower confidence interval)	15
People with H1N1 in 2010	16
Number of clinic beds	17
% Female Smokers	18
% Male Smokers	18

For Death Rate from Hospitals

Feature Name	Median Rank
Population over 75	1
Number of new ICU beds	2
People with H1N1 in 2010	3
Population aged 0-14	4
BMI of Females (lower confidence interval)	5
Number of clinic beds/100k population	6
Population aged 15-64	7
Number of doctors	8
Number of hospital beds	9
Per Capita Income	10
Population over 64	11
% Population that drink alcohol	12
Population Density	13
Number of clinic beds	14
Average BMI of males	15
Number of hospital beds/100k population	16
Number of doctors/100k population	17
% Female Smokers	18
% Male Smokers	18

For Hospitalization Rate

Supplementary Figure 51 | Ranking of static features for encoders in the Japan Model of January 20, 2021.

Feature Name	Median Rank
Mobility Changes: Residences	1
R_{eff}	2
Cases Mean to Sum Ratio	3
Deaths Mean to Sum Ratio	4
Weighted % of respondents reporting CLI	5
State of Emergency	6
Deaths	7
Std error of weighted % of respondents reporting CLI	8
Confirmed Cases	9
Std error of unweighted % of respondents reporting CLI	10
Mobility Changes: Parks	11
Mobility Changes: Transit	12
Mobility Changes: Retail/Recreation	13
Mobility Changes: Workplaces	14
Mobility Changes: Grocery Stores/Pharmacies	15

For Average Documented Contact Rate

Feature Name	Median Rank
Mobility Changes: Residences	1
Std error of unweighted % of respondents reporting CLI	2
R_{eff}	3
Confirmed Cases	4
Cases Mean to Sum Ratio	5
State of Emergency	6
Mobility Changes: Transit	7
Std error of weighted % of respondents reporting CLI	8
Weighted % of respondents reporting CLI	9
Mobility Changes: Workplaces	10
Deaths Mean to Sum Ratio	11
Mobility Changes: Retail/Recreation	12
Deaths	13
Mobility Changes: Parks	14
Mobility Changes: Grocery Stores/Pharmacies	15

For Average Undocumented Contact Rate

Feature Name	Median Rank
Confirmed Cases	1
Cases Mean to Sum Ratio	2
Deaths	3
Number discharged from hospitals	4
Day of Week	5
Deaths Mean to Sum Ratio	6

For Hospitalization Rate

Feature Name	Median Rank
Std error of unweighted % of respondents reporting CLI	1
Confirmed Cases	2
Std error of weighted % of respondents reporting CLI	4
Cases Mean to Sum Ratio	4
Deaths	5
Number of people tested	5
Weighted % of respondents reporting CLI	6
Deaths Mean to Sum Ratio	8
Day of Week	9

For Diagnosis Rate

Feature Name	Median Rank
Std error of weighted % of respondents reporting CLI	1
Std error of unweighted % of respondents reporting CLI	2
Weighted % of respondents reporting CLI	3
Deaths Mean to Sum Ratio	4
Confirmed Cases	5
Cases Mean to Sum Ratio	6
Deaths	7
Day of Week	8

For Documented Recovery Rate

Feature Name	Median Rank
Weighted % of respondents reporting CLI	1
Std error of weighted % of respondents reporting CLI	2
Std error of unweighted % of respondents reporting CLI	3
Confirmed Cases	4
Deaths	5
Deaths Mean to Sum Ratio	6
Cases Mean to Sum Ratio	7

For Undocumented Recovery Rate

Feature Name	Median Rank
Deaths Mean to Sum Ratio	1
Number discharged from hospitals	2
Cases Mean to Sum Ratio	3
Confirmed Cases	4
Day of Week	5
Deaths	6

For Recovery Rate from Hospitals

Feature Name	Median Rank
Confirmed Cases	1
Deaths	2
Deaths Mean to Sum Ratio	3
Cases Mean to Sum Ratio	4
Day of Week	5

For Documented Death Rate

Feature Name	Median Rank
Cases Mean to Sum Ratio	1
Deaths	2
Deaths Mean to Sum Ratio	3
Day of Week	4
Number discharged from hospitals	5
Confirmed Cases	6

For Death Rate from Hospitals

Supplementary Figure 52 | Ranking of time-varying features for encoders for Encoders in the Japan Model of January 20, 2021.

5 Supplementary Note 5: Ablation Studies

Table 41 shows the MAE and MAPE difference in cumulative deaths and confirmed cases with the removal of major constituents of our model.

Supplementary Table 41 | Ablation studies, showing the mean and 95 % confidence intervals of the MAE or MAPE gains aggregated along the prediction dates. Rows correspond to the baseline model performance, and ablation of (i) removal of using teacher forcing during training, (ii) using static rate variables for compartmental transitions (i.e. not utilizing covariates), (iii) removal of information sharing across locations (i.e. fitting different weights and biases to different locations) and (iv) removal of hospitalized, ICU, and ventilator compartments, and supervision from corresponding data sources.

	Death		Confirmed	
	MAPE (%)	MAE	MAPE (%)	MAE
Baseline	5.08 ± 1.22	82.76 ± 13.00	5.85 ± 1.06	8363.75 ± 2178.59
Without teacher forcing	+34.87 ± 9.36	+951.4 ± 238.2	+9.57 ± 6.23	+13367.87 ± 6226.95
With constant rates	+1.57 ± 2.86	+46.25 ± 30.78	+2.17 ± 3.66	+2215.74 ± 4009.00
Without information sharing across locations	+0.01 ± 0.20	+15.48 ± 9.19	+0.74 ± 0.79	+1389.68 ± 829.72
Without hospitalization	+1.00 ± 1.06	+16.04 ± 7.52	+0.13 ± 0.50	+1002.12 ± 1115.33

Robustness of sequential learning is highly important, especially learning from noisy data corresponding to highly nonlinear dynamics. To this end, we employ partial teacher-forcing (see the Section “Machine learning methods") and we demonstrate that partial teacher-forcing is highly important – without teacher-forcing, death forecasts would get much worse. This can be attributed to the rapidly-varying nature of time dynamics, and the robustness benefit of learning how to mitigate error propagation during training.

Our model integrates learning from static and time-varying features (see the Section “Data sources and preprocessing" for the full list), as opposed to using constant rates as standard compartmental models do. Using features improves forecasts for both death and confirmed cases, as the models can flexibly fit to different locations and timesteps as opposed to using constant rates, as in standard compartmental modeling.

Our mechanisms enable cross learning between the locations and we observe this information sharing to be beneficial, especially for confirmed forecasts. We attribute this mainly to the pandemic having different phases at different locations, especially in terms of the infection spreading. E.g. the impact of mask mandates for a particular state can be learned from other states who have introduced the mask mandates although that particular state has not before.

Unlike most compartmental models, our model utilizes hospitalization compartments (including those who are in ICU and on ventilators – see the Section “Proposed compartmental model"). We observe that these compartments improve the death forecasts noticeably as they carry highly informative signals, while being less important for confirmed as expected.

6 Supplementary Note 6: Model Fairness

COVID-19 has observed to have disproportionally large impact on communities that have larger proportions of minority groups such as African-American and Hispanic subpopulations in the

US[21, 22, 23, 24, 25] and it is important that our models' forecasts are accurate across demographic groups so that and does not exacerbate these discrepancies.

When the mean performance of the model across variations in a demographic feature of interest was calculated using mean values, the counties were sorted by the feature of interest and then grouped into bins that have approximately equal numbers of geographic regions (i.e. the first bin has the quarter of the areas with the lowest proportion of the population, the second bin contains the quarter of the areas whose proportions were between the 25th and 50th quantiles, etc.). It should be noted that while this methodology compares equal numbers of counties it can lead to large disparities in the number of people in each bin which, in turn, can lead to skewed distributions in ground truth data. The demographic data for the investigation into US counties was taken from 2018 US census data and the data for the Japanese investigation used the 2015 census for sex and ethnicity [26], Ministry of Health, Labour and Welfare statistics for age [27], and the Japanese Cabinet Office statistics for median income [28].

US County Analysis

To assess the relationship of the error to each of the features of interest, the MAPE of cumulative deaths of the prospective forecasts was calculated and the mean values were calculated for each of the groups of counties (as described above). Bootstrapping was used to estimate the confidence intervals in each group by sampling with replacement across both the forecast date and the counties 1,000 times. The resulting mean values and confidence intervals can be seen in Tables 42 - 46.

Supplementary Table 42 | The MAPE of cumulative deaths for quartiles of US counties grouped by the race and ethnicity subgroup percentages.

Race/Ethnicity	Group Percentage	MAPE [95% CI]
Black	<0.9%	0.294 [0.240,0.364]
	0.9%-2.6%	0.237 [0.197,0.280]
	2.6%-11.0%	0.192 [0.149,0.248]
	>11.0%	0.083 [0.073,0.096]
White	<79.6%	0.102 [0.087,0.122]
	79.6%-91.4%	0.189 [0.148,0.248]
	91.4%-95.5%	0.219 [0.185,0.259]
	>95.5%	0.293 [0.239,0.360]
Hispanic	<2.4%	0.241 [0.200,0.291]
	2.4%-4.4%	0.189 [0.155,0.227]
	4.4%-10.0%	0.190 [0.154,0.235]
	>10.0%	0.181 [0.143,0.231]

An alternative methodology for quantifying the relationship between the demographic features and the models' accuracy that takes into account the continuous nature of both features is to examine the correlation of the feature of interest with the error. Kendall's Tau was used to assess the relationship between the feature of interest and the MAPE of cumulative deaths or for each county because the MAPE values were not normally distributed and non-linear, but monotonic, relationships were also of interest [29, 30]. Significant correlations were found for all of the features other than the median income (Table 47). However, it should be noted that the most

Supplementary Table 43 | The MAPE of cumulative deaths for quartiles of US counties grouped by the percentage of the population that is female.

Percentage Female	MAPE [95% CI]
<49.4%	0.278 [0.222,0.344]
49.4%-50.3%	0.241 [0.195,0.308]
50.3%-51.0%	0.165 [0.140,0.195]
>51.0%	0.120 [0.100,0.145]

Supplementary Table 44 | The MAPE of cumulative deaths for quartiles of US counties grouped by median income.

Median Income	MAPE [95% CI]
<$42.4k	0.181 [0.155,0.212]
42.5k-49.8k	0.215 [0.177,0.266]
49.9k-57.6k	0.190 [0.162,0.222]
>$57.6k	0.216 [0.164,0.286]

Supplementary Table 45 | The MAPE of cumulative deaths for quartiles of US counties grouped by median age.

Median Age	MAPE [95% CI]
<38.4yrs	0.134 [0.110,0.167]
38.4yrs-41.5yrs	0.179 [0.141,0.232]
41.5yrs-44.7yrs	0.214 [0.175,0.260]
>44.7yrs	0.279 [0.233,0.337]

Supplementary Table 46 | The MAPE of cumulative deaths for quartiles of US counties grouped by population density.

Population Density	MAPE [95% CI]
<6.6 ppl./sq. mi.	0.324 [0.266,0.390]
6.6-17.5 ppl./sq. mi.	0.217 [0.179,0.265]
17.5-45.9 ppl./sq. mi.	0.148 [0.127,0.173]
>46.0 ppl./sq. mi.	0.119 [0.087,0.170]

significant correlation is with the total population of the county, rather than a demographic feature, which suggests that the impact of confounding features may be significant and simply looking at changes in the mean without simultaneously accounting for the influence of other features may not be sufficient.

To mitigate the impact of confounding features on the correlations, we calculate the partial correlation [31] between each demographic feature of interest and the average MAPE of cumulative deaths for each county during the prospective period. To take into account the interdependency between features when considering conditional outcomes, all of the other features were included

Supplementary Table 47 | Kendall's Tau between features of interest and the MAPE for a county.

feature	Tau	p-value	Adjusted p-value
Actual Deaths	-0.481	< 0.001	< 0.001
Total Population	-0.325	< 0.001	< 0.001
Fraction Black	-0.314	< 0.001	< 0.001
Population Density	-0.312	< 0.001	< 0.001
Fraction White	0.297	< 0.001	< 0.001
Increase in Deaths	-0.294	< 0.001	< 0.001
Fraction Female	-0.211	< 0.001	< 0.001
Median Age	0.196	< 0.001	< 0.001
Fraction Hispanic	-0.123	< 0.001	< 0.001
Median Income	-0.014	0.255	0.255

as confounding variables for partial correlation estimations. As was done above Kendall's Tau was used to quantify the remaining correlation between the MAPE and the feature of interest. We note that while this methodology helps control for the impact of confounding variables, it may also mask the impact of a single demographic variable if strong linear correlations between the factors exist. Table 48 demonstrates that while some of the demographic features of interest were found to be statistically significant, the only feature with a correlation that had a magnitude larger than 0.1 was the population density of the county and none of the correlations with the fractions of racial or ethnic groups were found to be statistically significant at the 0.025 level.

Supplementary Table 48 | The partial correlations between features of interest and the MAPE of cumulative deaths for US counties after conditioning on the other demographic features.

feature	Tau	p-value	Adjusted p-value
Population Density	-0.152	< 0.001	< 0.001
Median Age	0.072	< 0.001	< 0.001
Median Income	-0.059	< 0.001	< 0.001
Fraction Female	-0.041	0.001	0.003
Fraction White	-0.025	0.037	0.112
Fraction Hispanic	-0.024	0.047	0.112
Fraction Black	-0.010	0.397	0.397

Japan Prefecture Analysis

As was performed for the US county-level analysis, the MAPE of cumulative deaths for the prospective 28-day forecasts of deaths was calculated for Japan, and the aggregate statistics were calculated for each of the groups of the prefectures. The resulting mean values and confidence intervals can be seen in Tables 49 - 53.

When Kendall's Tau was used to quantify the correlation between the feature of interest and the MAPE of cumulative deaths for each Japanese prefecture. Significant correlations at the 95% confidence level were found for the total number of actual deaths in each prefecture, the percentage of the population that was in the middle age group, the percentage of the population in the older group, the population density, and the percentage of the population that was Chinese

Supplementary Table 49 | The MAPE of cumulative deaths for quartiles of Japanese prefectures grouped by the race percentages.

Race	Percentage	MAPE [95% CI]
Japanese	<98.6%	0.086 [0.066,0.109]
	98.6%-99.2%	0.169 [0.094,0.264]
	99.2%-99.5%	0.215 [0.130,0.326]
	>99.5%	0.223 [0.167,0.285]
Korean	<0.1%	0.239 [0.155,0.329]
	0.1%-0.1%	0.130 [0.083,0.180]
	0.1%-0.2%	0.172 [0.111,0.241]
	>0.2%	0.140 [0.079,0.230]
Chinese	<0.2%	0.203 [0.142,0.268]
	0.2%-0.3%	0.253 [0.154,0.372]
	0.3%-0.3%	0.137 [0.083,0.200]
	>0.4%	0.096 [0.063,0.139]

Supplementary Table 50 | The MAPE of cumulative deaths for quartiles of Japanese prefectures grouped by the percentage of the population that is female.

Percentage Female	MAPE [95% CI]
<50.8%	0.104 [0.065,0.156]
51.0%-51.8%	0.170 [0.088,0.277]
51.8%-52.7%	0.213 [0.125,0.308]
>52.7%	0.201 [0.148,0.264]

Supplementary Table 51 | The MAPE of cumulative deaths for quartiles of Japanese prefectures grouped by median income.

Median Income	MAPE [95% CI]
<¥257X	0.212 [0.142,0.284]
¥258X-¥285X	0.217 [0.144,0.306]
¥285X-¥301X	0.102 [0.058,0.153]
>¥303X	0.151 [0.093,0.242]

(Table 54) after p-values were adjusted using the Holm–Bonferroni method. However if the partial correlations are computed using the other demographic features as features none of the features are statistically significant (Table 55).

Supplementary Table 52 | The MAPE of cumulative deaths for Japanese prefectures grouped by the percentage of each age group.

Age Group	Range	MAPE [95% CI]
Younger	<11.7%	0.216 [0.134,0.323]
	11.7%-12.2%	0.128 [0.076,0.222]
	12.3%-12.7%	0.182 [0.129,0.250]
	>12.7%	0.160 [0.101,0.224]
Middle	<55.7%	0.243 [0.167,0.329]
	55.9%-57.3%	0.235 [0.156,0.331]
	57.5%-59.0%	0.109 [0.081,0.136]
	>59.3%	0.098 [0.062,0.149]
Older	<28.8%	0.100 [0.063,0.153]
	28.9%-30.4%	0.125 [0.087,0.171]
	30.6%-32.0%	0.246 [0.164,0.345]
	>32.0%	0.225 [0.137,0.326]

Supplementary Table 53 | The MAPE of cumulative deaths for quartiles of Japanese prefectures grouped by population density.

Density	MAPE [95% CI]
<179.0 ppl./sq. km	0.252 [0.187,0.317]
182.0-270.0 ppl./sq. km	0.200 [0.112,0.326]
275.0-475.0 ppl./sq. km	0.155 [0.106,0.205]
>478.0 ppl./sq. km	0.084 [0.057,0.117]

Supplementary Table 54 | Kendall's Tau between features of interest and the MAPE in the Japanese prefecture.

Feature	Tau	p-value	Adjusted p-value
Actual Deaths	-0.479	< 0.001	< 0.001
Middle	-0.447	< 0.001	< 0.001
Older	0.413	< 0.001	0.001
Population Density	-0.345	0.001	0.007
Chinese	-0.337	0.001	0.008
Japanese	0.297	0.004	0.026
Percentage Female	0.256	0.012	0.073
Total Population	-0.252	0.013	0.073
Median Income	-0.229	0.025	0.099
Increase in Deaths	-0.223	0.029	0.099
Korean	-0.159	0.118	0.236
Younger	-0.069	0.501	0.501

Supplementary Table 55 | The partial correlations between features of interest and the MAPE for Japanese prefectures after conditioning on the other demographic features.

Feature	Tau	p-value	Adjusted p-value
Percentage Female	-0.244	0.017	0.149
Population Density	0.235	0.021	0.171
Korean	0.154	0.132	0.925
Japanese	0.123	0.229	1.000
Chinese	-0.074	0.466	1.000
Older	-0.063	0.538	1.000
Median Income	0.061	0.551	1.000
Middle	-0.061	0.551	1.000
Younger	-0.057	0.576	1.000

7 Supplementary Note 7: Uncertainty Analysis

The ability for a model to distinguish what it does and doesn't know is an important characteristic for being able to make more reliable predictions [18, 19, 32, 20]. More formally, we can analyze this under an uncertainty quantification framework by measuring both data uncertainty and model uncertainty, where, under a fixed dataset, these are also referred to as aleatoric (data) and epistemic (model) uncertainties, respectively. Aleatoric uncertainty is irreducible uncertainty inherent in the data due to incomplete and unknown information, and is represented as a conditional distribution of the outputs conditioned on the observed inputs. Epistemic uncertainty is reducible uncertainty over the correct model, and is represented as a distribution over model functions. For our analysis of the reliability of our COVID-19 forecasts, we look into the calibration of our predicted uncertainties, specifically looking at the ability for our model uncertainty to be used for improving robustness by identifying and withholding unreliable forecasts.

Model uncertainty for unreliable prediction withholding

In order to identify predictions that are likely to be erroneous or otherwise unreliable, we look into into the relationship between model disagreement and metric performance. Here, model disagreement is measured as the variance in predictions from the top-k best models, averaged over a 28-day forecast. More specifically, the procedure is as follows. For each day in the retrospective period, we start with our standard setup for training models and releasing a 28-day forecast using the best model identified during hyperparameter tuning. We then make one modification: instead of using the single best model, we instead keep the top-k best models and generate a 28-day forecast with each, where we set $k = 5$. At evaluation time, we record two values for each location: (1) the metric performance of the single best model over the 28-day forecast (MAE or MAPE), and (2) the variance in predictions across the k models for each day, averaged over the 28-day period. The variance in predictions can be interpreted as a measure of model disagreement due to model uncertainty. We repeat this for all days in the retrospective period, yielding a tuple of metric performance and average prediction variance for each 28-day forecast release date for each location.

We then analyze this data in two ways. In Figure 53, we plot the average prediction variance versus the MAE metric performance on predicted confirmed cases, for all release dates and all prefectures in Japan. We can see that there is a positive correlation between the average prediction variance and the final metric performance. That is, higher model disagreement correlates with worse metric performance. We note though that the relationship is weaker when measured with MAPE instead of MAE, and that the correlation is noisier for some prefectures. Overall, since the prediction variance can be measured when we release a 28-day forecast, and thus before any ground truth can be observed, this correlation suggests that we could consider taking an action to mark 28-day forecasts with higher model disagreement as likely to have worse metric performance, i.e., to likely be less reliable.

To test this, we simulate the scenario of deciding, for each day, whether or not to withhold the 28-day forecast based on its associated model disagreement. We start with the data on metric performance and prediction variance from the retrospective period for Japan, as described above. For each location, we collect the set of average predicted variances for all release dates

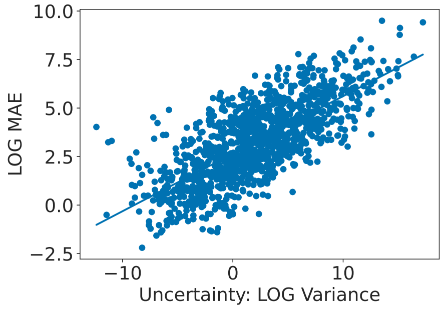

Supplementary Figure 53 | Model disagreement due to model uncertainty, measured as average prediction variance across the top $k = 5$ models, versus the MAE performance, both plotted in log space. From this, we see that higher model disagreement correlates with worse metric performance. For the best fit line, $R^2 = 0.539, 4.39x + 3.37$.

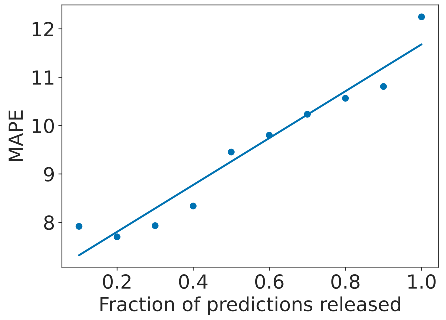

Supplementary Figure 54 | A rejection diagram showing the percentage of dates on which a prediction is made, after thresholding on model disagreement due to model uncertainty, versus the MAPE performance on those dates. From this, we can see that better average metric performance (on the days for which a forecast is released) can be achieved by withholding forecasts on days with higher model disagreement. Thus, we find the reliability of the forecasting system can be improved through model uncertainty thresholding. For the best fit line, $R^2 = 0.941, f(x) = 2.18x + 9.50$.

and compute ten quantiles at the $[10\%, 20\%, \ldots, 90\%, 100\%]$ levels. We then decide on which forecast dates to withhold predictions by thresholding the average predicted variance based on the value at a given quantile. More specifically, for each quantile q, we threshold the set of forecast dates, and release predictions on only those dates for which the associated uncertainty value is less than or equal to q. This yields ten groups of dates per location representing the bottom $[10\%, 20\%, \ldots, 90\%, 100\%]$, respectively, based on average predicted variance. We then compute the average metric performance for each group, and average over locations. Overall, this yields average metric performance at ten quantiles of uncertainty values, which correspond to making predictions for ten percentages of release dates.

In Figure 54, we plot this in the form of a rejection diagram [33, 34], which measures the percentage of dates on which a prediction is made (after thresholding on uncertainty) versus the average metric performance for those dates. From this, we can see that withholding forecasts on days with higher average predicted variance can lead to better average metric performance over the remaining forecasts. We note that this overall relationship holds when measured with

either MAPE or MAE. When examining single prefectures, the relationship holds when measured with MAE, but is noisier when measured with MAPE. Regardless, these findings suggest that on average, the reliability of our forecasting system can be improved through model uncertainty thresholding, at the cost of only releasing new 28-day forecasts on a lower percentage of days.

Overall, this analysis shows that prediction error is correlated with model disagreement (which is due to model uncertainty), and therefore, the more erroneous predictions can be identified at prediction time without access to ground truth values. Once identified, the system can take an action, such as withholding those predictions as described above. Other actions could include bringing an expert human into the loop to further analyze the predictions for that day, or simply adding a note to the released predictions to indicate that they may be less reliable. The uncertainty threshold used for the withholding decision can then be set adaptively based on the desired metric performance, as measured on a validation dataset. To reiterate, the goal here is to increase the reliability of the system by only releasing high-quality predictions for which the system is relatively certain.

8 Supplementary Note 8: Model applicability and case studies

We conducted a structured survey to understand how organisations were using our model forecasts. In total seven organisations responded. Their responses are summarised in Supplementary Table 56.

Supplementary Table 56 | Results of a structured survey on the applicability of the model

Organisation type	Academia (3), Government (2), Private industry (2)
Main user job role	Analyst/Scientist (3), Healthcare Professional (1), Statistician (2), Managerial (1)
Location	USA (4), Japan (3)
Predictions used	Confirmed cases (7), Death (4), Hospitalizations (4)<br>ICU (3), Ventilator (2), Infected (2)
Model use case	Resource allocation (2), business planning (2), scenario planning (1), general understanding of COVID spread (1), confirm existing forecasts (1)
Frequency of use	Daily (1), Weekly (1), Monthly (1)
Was the model helpful	Yes (7)

Case studies

Estimating state and national COVID-19 testing targets

"We have an international dashboard at HGHI and Brown School of Public Health that combines a seven-day moving average of cases, hospitalizations, and deaths with forecasts from the Google-Harvard CPF. This dashboard has been used for scientific communication efforts by Dr. Thomas Tsai and Dr. Ashish Jha in the media.

"The HSPH and Brown School of Public Health team have used the Google-Harvard CPF to create estimates of state and national testing targets. To create state and national COVID-19 testing targets, we began by obtaining projected cases on January 1 from the public Google Cloud Forecasting model, and these projections formed the backbone of our entire estimate.

We converted these projected cases to projected infections using a case detection rate (ratio of infections to cases) that was averaged from the Gu and CovidEstim forecast models, and then extrapolated the number of symptomatic infections and the number of contacts who would emerge from this infected population. Lastly, we incorporated testing for individuals with flu-like symptoms using CDC ILI surveillance and added allocation for surveillance testing based on the size of student, prison, nursing home, and healthcare worker populations.

"Example news coverage:
https://www.npr.org/sections/health-shots/2020/12/22/948085513/vaccines-are-coming-but-the-u-s-still-needs-more-testing-to-stop-the-surge

"Testing Target Dashboard:
https://globalepidemics.org/testing-targets/january-1-2021-state-testing-targets/"

Informing state-wide social distancing measures

"We have also used the Google-Harvard CPF estimates to inform our guidance to state public health leaders. We were recently asked by Department of Public Health officials from State A to assess tradeoffs in the timing of issuing a stay-at-home order. Using the Google-Harvard CPF projections as our starting point, we were able to estimate the expected rate of exponential growth and show how a lag of a few days in implementing social distancing measures could lead to a much larger number of deaths in the ensuing weeks. After reviewing this data over the weekend, the governor of State A implemented substantial social distancing measures on Monday morning to help curb the spread of COVID-19 in the state."

Allocating Department of Defence resources

"We used the forecasts to help the Command determine where DoD resources could be requested, and at what point requests for DoD support should be terminated based on individual state capabilities and capacity. We would compare the Google Public Forecast to others available and find the delta between them. When there were large variances, we dug into the data and local events to identify potential causes for the deviations. We used a total of 6-8 predictive models at any given time, open source research of the local areas, HHS reporting on hospital capacity, and DHS reporting on case spread/emerging hotspots.

"Google allowed us to submit specific events, such as the Sturgis Motorcycle Rally, and evaluate the potential impact on the spread of the disease through lower use of NPI, increased travel, and other factors. These “What-if Analysis” events helped us provide COVID threat assessments for multiple events that could increase disease spread. We also used the Google model to support disease threat assessments and mitigation planning for events where DoD had participation."

Making business decisions during the pandemic

"Based on the predicted number of infections by prefecture, we use the forecasts to anticipate in advance areas where infections are likely to spread, and where requests from local governments are likely to be issued. Most recently, we use the forecasts to anticipate when the Japanese Governments emergency declaration may be canceled. Based on the predicted number of confirmed cases from the infection prediction we can check whether the weekly average will meet the government's emergency declaration issuance and cancellation criteria are met.

"We want to know advance the possibility that a request from the local government will be issued, so we can better plan our sales forecasting and cost reduction. If we have the actual results of the floating population by region and day of the week, and the forecast, we think that we can take business decisions that are more effective. Our company is in service industry with about 160 stores in more than 20 prefectures. Government requests for self-restraint from each business are being made based on the spread of infection. Where requests are issued we check the available prior information at different timings, periods, contents, and transmission methods depending on each local government. By making a prediction that a local government request will be issued in advance from the infection prediction, information can be obtained almost without omission. We were able to anticipate the request and respond according to the request in each prefecture.

"In addition, because there is an infection prediction, the management can discuss whether or not we should respond according to the request at an earlier timing in response to the request of each local government. Furthermore, we can allow each store manager to make decisions based the future spread of infection. For example, if the infection forecast increases and there is a possibility that the store may have to close or reduce it's hours (or vise versa, if infections decrease and hours can be extended), adjustments can be made to worker shift patterns in advance. Even if we haven't decided on the above measures, we were able to convey the possibility with a certain degree of certainty, so we believe that the on-site adjustments were smooth."

Supplementary References

[1] Fda briefing document moderna covid-19 vaccine. https://www.fda.gov/media/144434/download/. Accessed: 2021-02-15.

[2] David Harvey, Stephen Leybourne, and Paul Newbold. Testing the equality of prediction mean squared errors. *International Journal of forecasting*, 13(2):281–291, 1997.

[3] Roberto S Mariano and Francis X Diebold. Comparing predictive accuracy. *J. Bus. Econ. Stat.*, 13:253, 1995.

[4] Sture Holm. A simple sequentially rejective multiple test procedure. *Scandinavian Journal of Statistics*, pages 65–70, 1979.

[5] Denis Kwiatkowski, Peter CB Phillips, Peter Schmidt, and Yongcheol Shin. Testing the null hypothesis of stationarity against the alternative of a unit root: How sure are we that economic time series have a unit root? *Journal of Econometrics*, 54(1-3):159–178, 1992.

[6] Elizabeth A. Stuart. Matching Methods for Causal Inference: A Review and a Look Forward. *Statistical Science*, 25(1):1 – 21, 2010.

[7] JM Oakes and Pamela Jo Johnson. *Propensity Score Matching for Social Epidemiology*, volume 1, pages 364–386. 05 2006.

[8] Ariel Linden and Paul Yarnold. Combining machine learning and matching techniques to improve causal inference in program evaluation. *Journal of Evaluation in Clinical Practice*, 22:868–874, 12 2016.

[9] Anthony Scotina and Roee Gutman. Matching algorithms for causal inference with multiple treatments. 09 2018.

[10] Raffaele Vardavas, Aaron Strong, Jennifer Bouey, Jonathan William Welburn, Pedro Nascimento de Lima, Lawrence Baker, Keren Zhu, Michelle Priest, Lynn Hu, and Jeanne S. Ringel. *The Health and Economic Impacts of Nonpharmaceutical Interventions to Address COVID-19: A Decision Support Tool for State and Local Policymakers*. RAND Corporation, Santa Monica, CA, 2020.

[11] Asli Demirguc-Kunt, Michael Lokshin, and Ivan Torre. The sooner, the better: The early economic impact of non-pharmaceutical interventions during the COVID-19 pandemic. *World Bank Policy Research Working Paper*, (9257), May 2020.

[12] Bella Nichole Kantor and Jonathan Kantor. Non-pharmaceutical interventions for pandemic COVID-19: A cross-sectional investigation of us general public beliefs, attitudes, and actions. *Frontiers in Medicine*, 7:384, 2020.

[13] Holly Seale, Clare E. F. Dyer, Ikram Abdi, Kazi M. Rahman, Yanni Sun, Mohammed O. Qureshi, Alexander Dowell-Day, Jonathon Sward, and M. Saiful Islam. Improving the impact of non-pharmaceutical interventions during COVID-19: examining the factors that influence engagement and the impact on individuals. *BMC Infectious Diseases*, 20(607), 2020.

[14] Navid Ghaffarzadegan. Simulation-based what-if analysis for controlling the spread of COVID-19 in universities. *PLoS ONE*, 16(2), 2021.

[15] Pfizer and biontech conclude phase 3 study of COVID-19 vaccine candidate, meeting all primary efficacy endpoints - pfizer press release, November 2020.

[16] Moderna says its COVID-19 vaccine is nearly 95 percent effective, November 2020.

[17] J. Grauer, H. Löwen, and B. Liebchen. Strategic spatiotemporal vaccine distribution increases the survival rate in an infectious disease like COVID-19. *Nature Scientific Reports*, 10(21594), 2020.

[18] Alex Kendall and Yarin Gal. What Uncertainties Do We Need in Bayesian Deep Learning for Computer Vision? In *Advances in Neural Information Processing Systems*, 2017.

[19] Andrey Malinin and Mark Gales. Predictive Uncertainty Estimation via Prior Networks. In *Advances in Neural Information Processing Systems*, February 2018.

[20] Michael W. Dusenberry, Dustin Tran, Edward Choi, Jonas Kemp, Jeremy Nixon, Ghassen Jerfel, Katherine Heller, and Andrew M. Dai. Analyzing the role of model uncertainty for electronic health records. In *Proc. of the ACM Conference on Health, Inference, and Learning (ACM CHIL)*, pages 204–213, Toronto Ontario Canada, April 2020. ACM.

[21] Clyde W. Yancy. COVID-19 and African Americans. *JAMA*, 323(19):1891–1892, 05 2020.

[22] Monica Webb Hooper, Anna María Nápoles, and Eliseo J. Pérez-Stable. COVID-19 and Racial/Ethnic Disparities. *JAMA*, 323(24):2466–2467, 06 2020.

[23] Merlin Chowkwanyun and Adolph L. Reed. Racial health disparities and COVID-19 — caution and context. *New England Journal of Medicine*, 383(3):201–203, 2020.

[24] Neeraj Bhala, Gwenetta Curry, Adrian R Martineau, Charles Agyemang, and Raj Bhopal. Sharpening the global focus on ethnicity and race in the time of COVID-19. *The Lancet*, 395(10238):1673–1676, 2020/07/15 2020.

[25] Carrie Henning-Smith, Mariana Tuttle, and Katy B Kozhimannil. Unequal distribution of COVID-19 risk among rural residents by race and ethnicity. *The Journal of rural health*, page 10.1111/jrh.12463, 05 2020.

[26] The Statistics Bureau of Japan. National census, 2015.

[27] The Statistics Bureau of Japan. The 66th Japan statistical yearbook 2017, 2015.

[28] Organisation for Economic Cooperation and Development. OECD.stat, 2000.

[29] Marie-Therese Puth, Markus Neuhäuser, and Graeme D Ruxton. Effective use of spearman's and kendall's correlation coefficients for association between two measured traits. *Animal Behaviour*, 102:77–84, 2015.

[30] Haldun Akoglu. User's guide to correlation coefficients. *Turkish Journal of Emergency Medicine*, 18(3):91–93, 2018.

[31] Ronald Aylmer Fisher et al. 035: The distribution of the partial correlation coefficient. 1924.

[32] Yaniv Ovadia, Emily Fertig, Jie Ren, Zachary Nado, D. Sculley, Sebastian Nowozin, Joshua V. Dillon, Balaji Lakshminarayanan, and Jasper Snoek. Can You Trust Your Model's Uncertainty? Evaluating Predictive Uncertainty Under Dataset Shift. *arXiv:1906.02530*, June 2019.

[33] Angelos Filos, Sebastian Farquhar, Aidan N. Gomez, Tim G. J. Rudner, Zachary Kenton, Lewis Smith, Milad Alizadeh, Arnoud de Kroon, and Yarin Gal. Benchmarking Bayesian Deep Learning with Diabetic Retinopathy Diagnosis. *arXiv:1912.10481*, 2019.

[34] Joost van Amersfoort, Lewis Smith, Yee Whye Teh, and Yarin Gal. Uncertainty Estimation Using a Single Deep Deterministic Neural Network. *arXiv:2003.02037*, June 2020.
